# Genome changes due to artificial selection in U.S. Holstein cattle

**DOI:** 10.1186/s12864-019-5459-x

**Published:** 2019-02-11

**Authors:** Li Ma, Tad S. Sonstegard, John B. Cole, Curtis P. VanTassell, George R. Wiggans, Brian A. Crooker, Cheng Tan, Dzianis Prakapenka, George E. Liu, Yang Da

**Affiliations:** 1 Department of Animal and Avian Sciences, University of Maryland, College Park, MD USA; 2 Recombinetics, Saint Paul, MN USA; 3 Animal Genomics and Improvement Laboratory, USDA-ARS, Beltsville, MD USA; 4 Department of Animal Science, University of Minnesota, Saint Paul, MN USA; 5 College of Animal Science and National Engineering Research Center for Breeding Swine Industry, South China Agricultural University, Guangzhou, People’s Republic of China

**Keywords:** Genome landscape, Genetic selection, Milk production, Fertility, Immunity

## Abstract

**Background:**

The availability of a unique unselected Holstein line since 1964 provided a direct comparison between selected and unselected Holstein genomes whereas large Holstein samples provided unprecedented statistical power for identifying high-confidence SNP effects. Utilizing these unique resources, we aimed to identify genome changes affected by selection since 1964.

**Results:**

Direct comparison of genome-wide SNP markers between a Holstein line unselected since 1964 and contemporary Holsteins showed that the 40 years of artificial selection since 1964 resulted in genome landscape changes. Among the regions affected by selection, the regions containing 198 genes with fertility functions had a larger negative correlation than that of all SNPs between the SNP effects on milk yield and daughter pregnancy rate. These results supported the hypothesis that hitchhiking of genetic selection for milk production by negative effects of fertility genes contributed to the unintended declines in fertility since 1964. The genome regions subjected to selection also contained 67 immunity genes, the bovine MHC region of Chr23 with significantly decreased heterozygosity in contemporary Holsteins, and large gene clusters including T-cell receptor and immunoglobulin genes.

**Conclusions:**

This study for the first time provided direct evidence that genetic selection for milk production affected fertility and immunity genes and that the hitchhiking of genetic selection for milk production by negative fertility effects contributed to the fertility declines since 1964, and identified a large number of candidate fertility and immunity genes affected by selection. The results provided novel understanding about genome changes due to artificial selection and their impact on fertility and immunity genes and could facilitate developing genetic methods to reverse the declines in fertility and immunity in Holstein cattle.

**Electronic supplementary material:**

The online version of this article (10.1186/s12864-019-5459-x) contains supplementary material, which is available to authorized users.

## Background

Genetic selection of U.S. Holstein cattle during the 40 years of 1964–2004 resulted in a virtually straight-line increase in milk yield and straight-line decrease in fertility measured by daughter pregnancy rate (Additional file 1). During that period, annual milk yield had a 79% increase from 6309 kg to 11,324 kg, but the 2004 Holstein cows required about 30 days longer than the 1964 cows for a successful conception. A group of selected Holsteins (selection line) and a group of unselected Holsteins (control line) since 1964 in this study were maintained under the same management and environment conditions at the University of Minnesota [1]. The control line was maintained without any genetic selection, whereas the selection line was continuously selected for high milk yield. Because of these two different breeding goals, the control line remained unchanged for milk yield and daughter pregnancy rate, whereas the selection line had the same trend as the national average for milk yield and fertility: virtually a straight-line increase in milk yield and a straight-line decrease in daughter pregnancy rate (Additional file 1). Since the control and selection lines were maintained under the same management and environment conditions, the increases in milk production during the 40 years of selection were primarily direct selection response to genetic selection for milk production because increased milk production was the primary selection target. Similarly, management and environment conditions could not be the primary reason for the fertility declines of the selected Holstein cows because the two groups of selected and unselected Holstein cows were maintained under the same management and environment conditions. Therefore, the declines in daughter pregnancy rate could only be negative indirect selection responses caused by focused selection for milk production, because no systematic selection for decreased fertility was known to exist and the genetic evaluation of daughter pregnancy rate as a fertility trait did not exist until 2003 [2]. Genetic selection for increased milk production was also accompanied by increased veterinary expenses in selected Holstein cattle [3] that we hypothesize was due to decreased immunity, and the reason for the increased veterinary expenses could be negative indirect selection responses of immunity caused by selection for milk production, similar to the reason for decreased fertility. The fertility and immunity declines were unintended consequences of genetic selection for increased milk production. The primary goal of this research was to identify genome regions affected by selection and to find genes with fertility and immunity functions that could explain the unintended consequences of decreased fertility and immunity due to selection.

Selection signature analysis can detect a genomic region affected by genetic selection because genetic selection leaves its signature on the genome. Allele frequency change is the most fundamental change due to selection [4, 5], and a genomic region subjected to selection typically has a long-range pattern of linkage disequilibrium (LD) due to hitchhiking between the selection target and the neighboring variants [6–8]. Selection signature analysis based on allele frequency differences and long-range LD patterns of genome regions have identified a large number of genomic regions affected by selection from within-breed analysis of Holsteins [9–14] or from comparative analysis between Holsteins and other cattle breeds [15–19]. However, the relationship between genome changes and the decreased fertility and immunity in Holstein cattle remained unknown. The phenotype of daughter pregnancy rate had a low heritability [20] and could be a manifest of many component traits that do not have phenotypic measures. Consequently, each genetic variant associated with daughter pregnancy rate would tend to have a small effect and many of these small effects would be difficult to detect reliably. In contrast, many fertility genes had experimental evidence of fertility as shown by a large number of articles cited by this study, and the known fertility genes present in genome regions affected by selection could explain the unintended declines in fertility. Similarly, the known immunity genes present in genome regions affected by selection could explain the unintended declines in immunity. The availability of a group of Holstein cattle unselected since 1964 provided a unique opportunity of selection signature analysis for identifying genomic regions affected by selection using direct comparison of the selected and unselected Holstein genomes. Utilizing these unique resources, we investigated genome changes due to selection since 1964, the relationship between the genome changes and the unintended declines in fertility and immunity, and the relationship between genetic selection and the SNP effects for milk production.

## Results

### Time trend of the Holstein genome landscape change

**Fig. 1:**
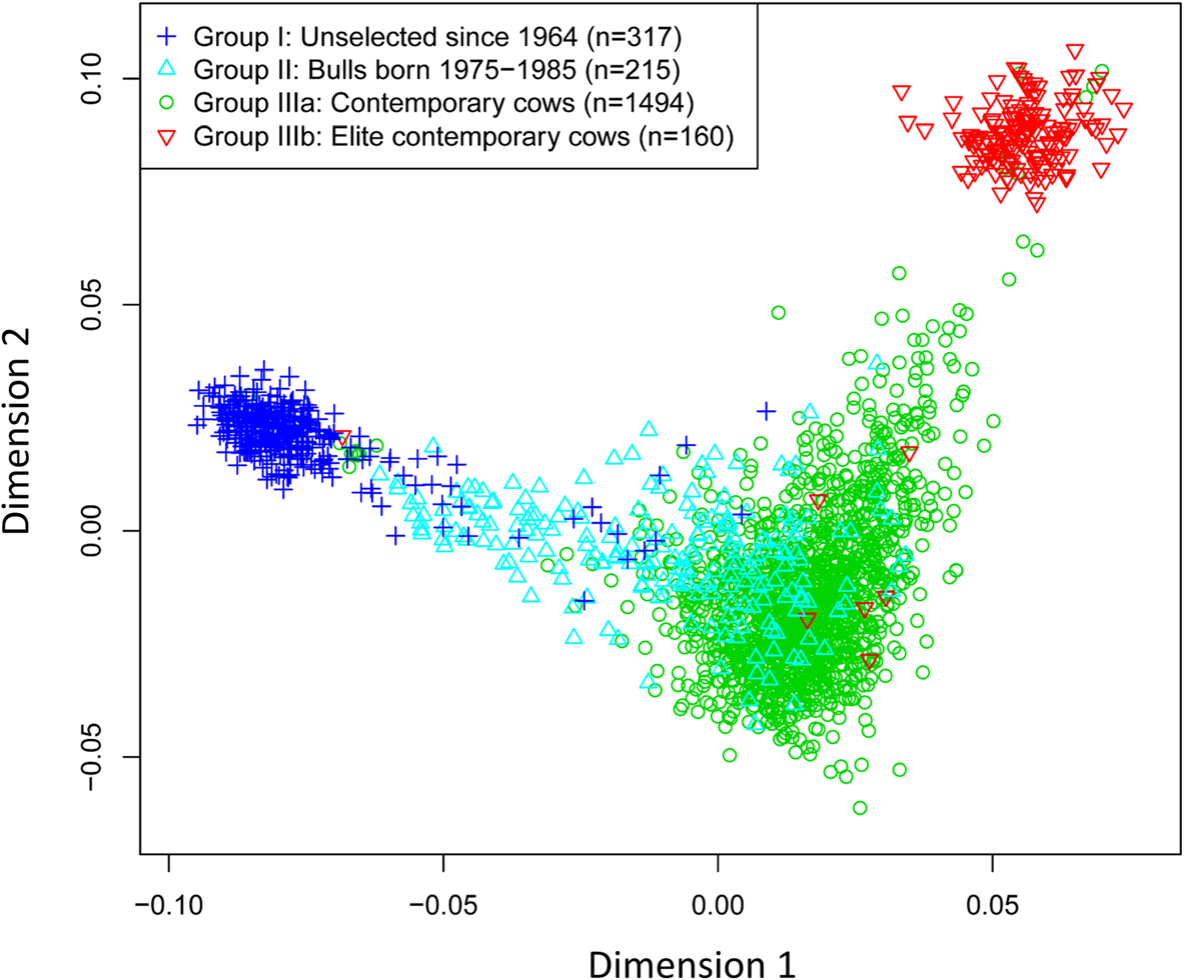
Multidimensional scaling (MDS) plot of the first dimension versus the second dimension of the SNP identity by state (IBS) distances. The figure shows a genome landscape shift from the unselected Holsteins at the lower left to the elite Holsteins at the upper right

The Holstein genome had a clear time trend of landscape change from the unselected to the contemporary Holstein genomes since 1964. The multidimensional scaling (MDS) plot of the first dimension versus the second dimension of the SNP identity by state distances [21] showed a shift from lower-left for the unselected cattle (Group I) to the upper-right for the selected cattle (Groups II and III) (Fig. 1). Dimension 1 was at the lower left corner for the unselected Holsteins (Group I), shifted to the middle in the upper-right direction for Holsteins subjected to 20 years of selection (Group II), and reached the upper-right of the figure for Holsteins subjected to 40 years of selection (Groups IIIa and IIIb, two sub-groups of Group III). Group IIIb at the extreme upper-right of the figure was a group of elite cows of 160 half sibs, and Group IIIa was the rest of Group III after removing cows of Group IIIb. The time trend of Holstein genome landscape change was further confirmed by the relationship between birth year and the MDS dimensions (Additional file 2). Dimension 1 had a clear time trend and explained the time trend of the Holstein genome landscape change. Dimensions 3 and 4 had no time trend, and Dimension 2 only had differences between the unselected group (Group I) and the elite group (Group IIIb).

### Genome-wide allele frequency changes

**Fig. 2:**
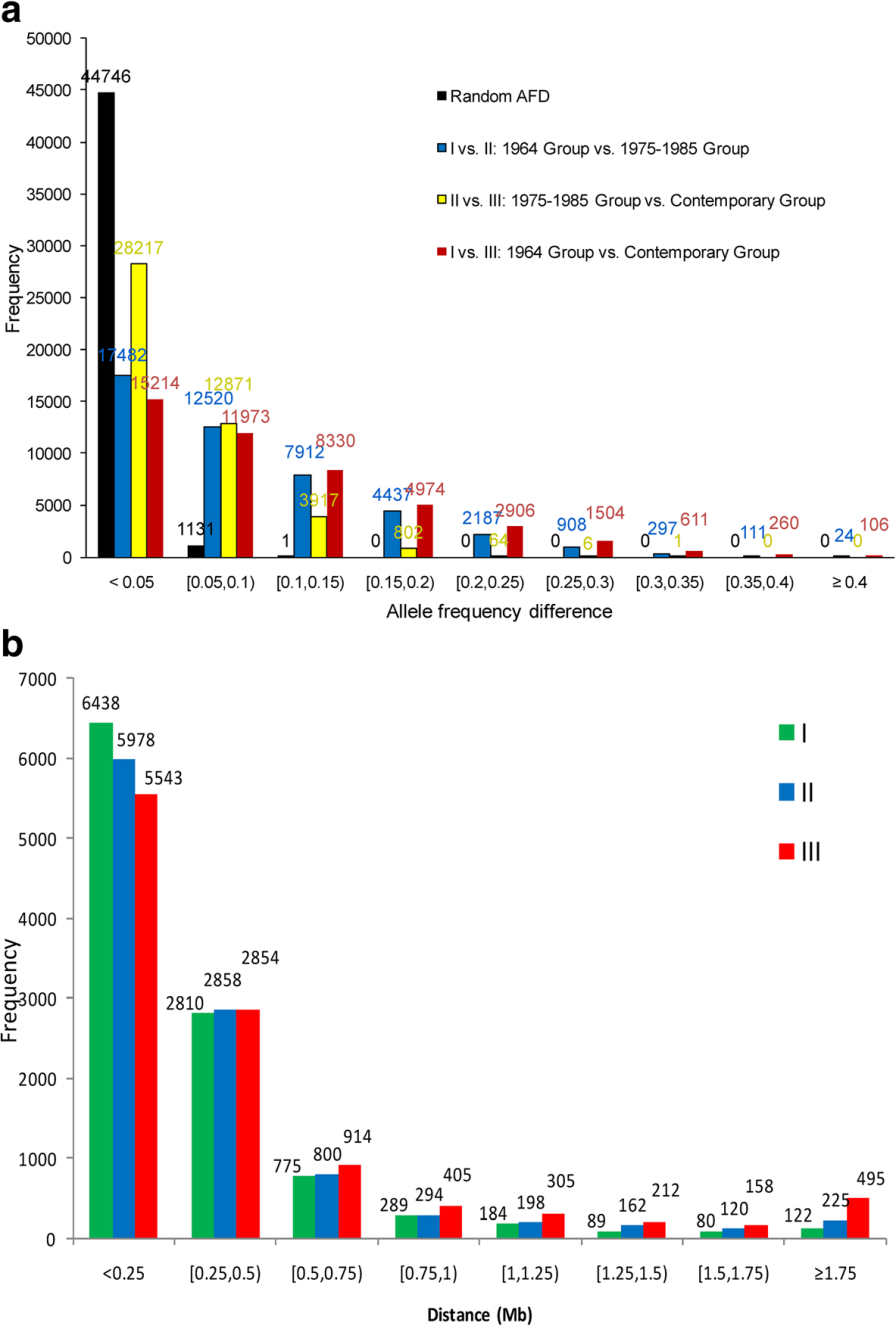
Allele frequency difference (AFD) and haplotypes with long distances of extended haplotype homozygosity (EHH). **a**. The 40 years of selection had the largest numbers of large AFD between Groups I and III, followed by the first 20 years of selection between Groups I and II, and the second 20 years of selection between Groups II and III. Random AFD is the AFD between the selected cows at the University of Minnesota and the rest of the contemporary Holsteins in Group III. **b**. The 40 years of selection had the largest numbers of long-distance EHH between Groups I and III, followed by the first 20 years of selection between Groups I and II, and the second 20 years of selection between Groups II and III. The EHH distances were distances of haplotypes with minimal EHH of 0.6

The 40 years of genetic selection resulted in genome-wide allele frequency differences (AFD) between the selected and unselected cattle (Fig. 2a, Additional file 3: Figure S3a). Several genes known to be associated with fertility and immunity had large AFD between the 1964 Holsteins and the 294,079 cows mostly born in 2006–2015 (Table 1). The largest AFD among all SNPs was 0.60 in the *ADGRG2* gene, which was associated with fluid dysregulation and male infertility [22] and decidualization of endometrial stromal cells [23]. The major allele of this gene in the 1964 Holsteins had a high frequency of 0.99 but became a minor allele in the contemporary cows with a frequency of 0.39. The *AVEN* gene associated with defective spermatogenesis [24], *SPATA6* associated with normal assembly of the sperm connecting piece and tight head–tail conjunction [25], and *ERBB4* associated with embryonic lethality in mice [26] also had large AFD, 0.56, 0.51 and 0.48 respectively. The *BOLA-DRB2* gene, a class II gene of the bovine major histocompatibility complex (MHC), had AFD of 0.40, one of the largest AFD among the immunity genes identified in this study. Of the 21 genes in Table 1, 19 genes were associated with fertility or immunity or both. These results provided initial indication that the genetic selection since 1964 affected fertility and immunity genes. The allele frequencies of *DGAT1* that was widely confirmed to have the most significant effects on milk production surprisingly did not have much allele frequency change since the mid-1980’s, apparently due to the antagonism between fat yield and milk and protein yields [27–29]. The selection for high fat yield would decrease milk and protein yields or vice versa, leaving *DGAT1* relatively unchanged since the mid-1980’s.

**Table 1:** Allele frequency changes since 1964 in selected genes. (‘Allele 1’ is the lower-ranked letter of any pair of *A*, *C*, *G*, *T*)

SNP^a^			frequency of ‘allele 1’ in population				
chr	position (bp)	gene	I ^b^	II ^b^	III ^b^	Current ^c^	AFD ^d^
X	114,995,979	*ADGRG2* ^e^	0.99	0.62	0.45	0.39	0.60
10	28,703,125	*AVEN* ^e^	0.02	0.30	0.51	0.59	0.56
X	116,153,810	*DMD* ^f^	0.05	0.46	0.53	0.58	0.53
3	98,450,919	*SPATA6* ^e^	0.08	0.53	0.57	0.59	0.51
2	100,376,419	*ERBB4* ^e^	0.08	0.29	0.47	0.56	0.48
23	27,215,001	*SKIV2L* ^e^	0.37	0.58	0.78	0.84	0.47
1	88,050,696	*USP13* ^g^	0.22	0.57	0.63	0.64	0.42
11	6,724,678	*IL1R2* ^g^	0.68	0.33	0.29	0.26	0.42
13	76,932,968	*SULF2* ^e^	0.19	0.38	0.51	0.61	0.41
26	17,465,791	*BLNK* ^g^	0.41	0.74	0.79	0.83	0.41
19	24,812,617	*SPATA22* ^e^	0.80	0.49	0.42	0.39	0.40
23	25,552,635	*BOLA-DRB2* ^g^	0.22	0.49	0.60	0.62	0.40
1	65,433,706	*GSK3B* ^g^	0.54	0.21	0.17	0.16	0.38
12	35,934,370	*LATS2* ^e, g^	0.42	0.23	0.14	0.08	0.34
9	90,155,533	*ESR1* ^e^	0.45	0.24	0.16	0.13	0.32
20	32,030,332	*GHR* ^e^	0.42	0.61	0.70	0.73	0.31
11	28,602,889	*EPAS1* ^e, g^	0.41	0.60	0.64	0.71	0.30
1	95,258,770	*SPATA16* ^e^	0.92	0.65	0.69	0.62	0.30
11	31,296,603	*FSHR* ^e^	0.03	0.23	0.25	0.26	0.23
20	39,017,985	*PRLR* ^e^	0.70	0.61	0.50	0.49	0.21
14	1,801,116	*DGAT1* ^h^	0.93	0.71	0.82	0.77	0.16

^a^ These SNPs were selected based on three criteria: AFD is large among all SNPs, the SNP is in a gene with known fertility or immunity function, or other well established functions (*DGAT1* and *DMD*)

^b^ I, II, III represent Groups I, II and III respectively, where Group I was unselected, Group II was subjected to 20 years of selection, and Group III was subjected to 40 years of selection since 1964

^c^ The current population had 294,079 cows with 98% of the cows born in 2006–2015

^d^ This is the absolute allele frequency difference between Group I and the current population approximately due to 50 years of selection

^e^ Associated with fertility

^f^ Associated with muscle diseases

^g^ Associated with immunity

^h^ Widely confirmed to have the most significant effects on milk production traits in dairy cattle

To estimate the total genome changes due to selection using the AFD results (Fig. 2a), a threshold value for random AFD is needed. We defined the AFD between the 149 contemporary cows (part of group III) maintained at the University of Minnesota and the rest of the contemporary Holsteins in Group III as random AFD because contemporary Holsteins had the same selection goals. The largest random AFD was below 0.10 except one value of 0.103 (Fig. 2a). Therefore, we used AFD ≥ 0.10 as a conservative and approximate threshold for non-random AFD due to selection. Some SNPs with AFD ≥ 0.10 could be random AFD and some SNPs with AFD < 0.10 could be due to selection. To reduce random AFD among SNPs with AFD ≥ 0.10, we further identified AFD that changed in the same direction during the first and second 20 years of selection, because such AFD should have less chance to be random. The 40 years of selection had the largest number of SNPs with AFD ≥ 0.10 (18,693 SNPs or 40.74%), followed by the first 20 years of selection (15,876 SNPs or 34.60%), and the second 20 years of selection (4790 SNPs or 10.44%) (Fig. 2a). The result that the first 20 years had more allele frequency changes than the second 20 years was consistent with the fact that only a small number of traits were included in the USDA’s genetic evaluation during the first 20 years of selection before 1985 and more traits were added to the genetic evaluation after 1985. This is because multi-trait selection may select multiple genomic regions and hence reduce the speed of change for regions associated with fewer traits. Before 1985, the USDA genetic evaluation only included milk and fat yields until protein yield was added for bull evaluations in 1977, type evaluation was added in 1978, and more traits were added since 1994 [30]. Of the 45,878 SNPs, 18,229 (39.7%) had allele frequency changes in the same direction during the first and second 20 years and had AFD ≥ 0.10. Therefore, the 40 years of genetic selection since 1964 approximately affected 40% (39.7–40.74%) of the Holstein genome. Although the 40 years of genetic selection since 1964 resulted in genome-wide allele frequency changes, allele fixation due to selection was not observed.

### Long-range haplotype and frequency changes

**Fig. 3:**
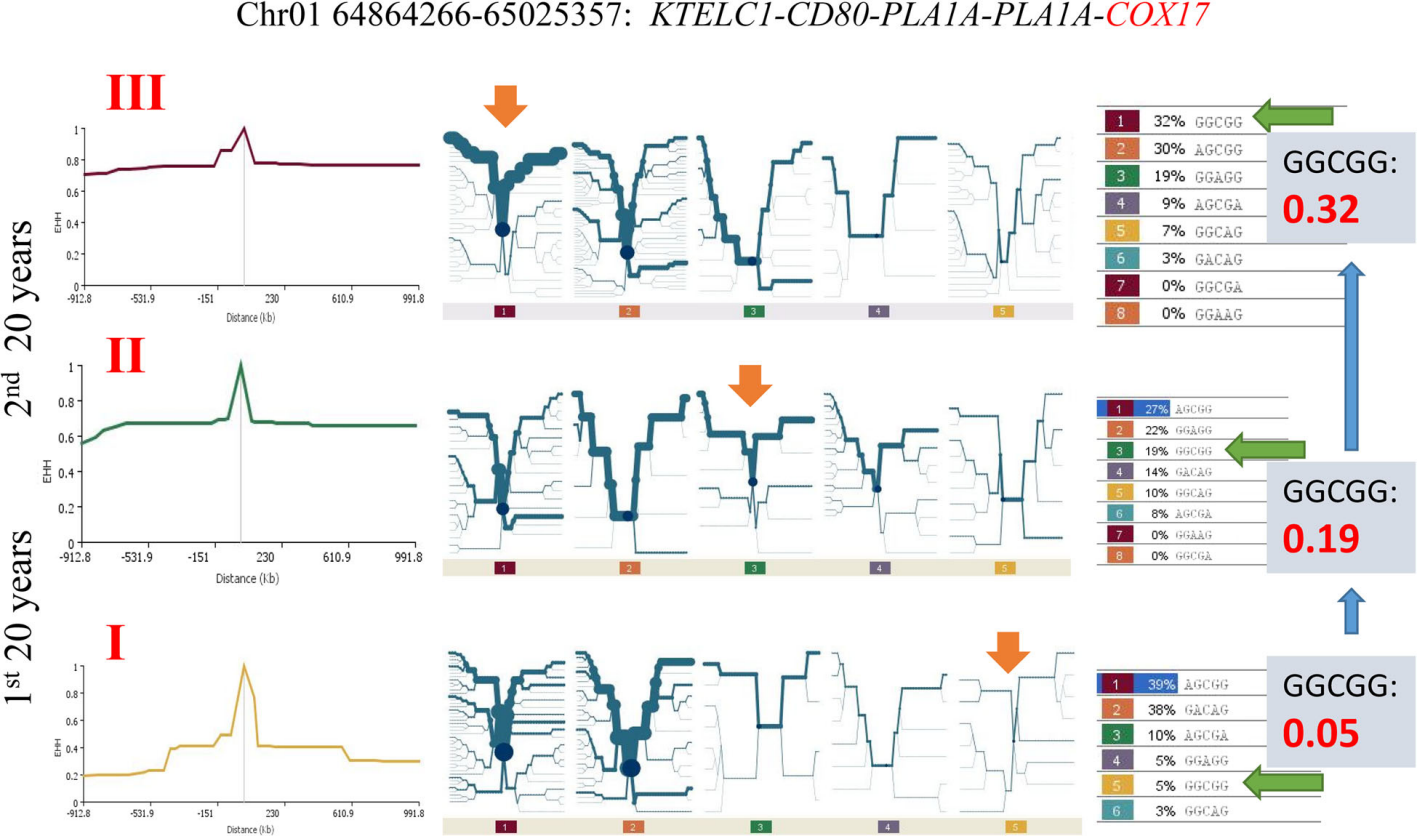
The 64.86–65.05 Mb region of Chr01 had increased EHH continuously during the 40 years of selection. The Holstein cows subjected to 40 years of selection had the highest EHH values (first column). The frequency of the *GGCGG* haplotype was 0.05 in the unselected Holsteins (Group I), increased to 0.19 in Group II after 20 years of selection, and increased to 0.32 in Group III after 40 years of selection (second and third columns).

To identify specific chromosome regions affected by selection, we conducted two types of long-range analysis: extended haplotype homozygosity (EHH) and long-range AFD. These long-range analyses have the advantage to average out random allele frequency changes such as genetic drift [7] or minimize the influence of genetic drift in identifying genomic regions with increased LD due to selection [31]. Genetic selection increases the EHH value, frequencies and distances of haplotypes being selected due to increased LD between loci in the region subjected to selection [6, 8]. The Holstein cows subjected to selection (Groups II and III) had more regions with long EHH distances (haplotype distances with EHH ≥ 0.60) than the unselected Holsteins (Group I). Group III with 40 years of selection had 1170 chromosome regions with EHH distances ≥1 Mb, Group II with 20 years of selection had 705, whereas Group I without selection since 1964 had 475 such chromosome regions (Fig. 2b). Haplotypes with increased EHH distances generally had increased frequencies and became major haplotypes in the selected Holsteins (Groups II and III). The 64.86–65.05 Mb region of Chr01 was an example of continued changes in haplotype frequencies with the *GGCGG* haplotype being selected continuously for the entire 40 years of selection since 1964. The *GGCGG* frequency increased from 0.05 in the unelected Holsteins (Group I), to 0.19 in Holsteins subjected to 20 years of selection (Group II), and to 0.32 in Holsteins subjected to 40 years of selection (Group III). The gain in the *GGCGG* haplotype was at the expense of the *GACAG* haplotype, which had a high frequency of 0.38 in the unselected Holsteins, 0.14 in the Holsteins subjected to 20 years of selection, and a low frequency of 0.03 in the Holsteins subjected to 40 years of selection (Fig. 3). Chr20 had the strongest EHH evidence of genetic selection. The 21–49 Mb region in the center of Chr20, approximately 28 Mb in size, was covered by long haplotypes with high frequencies and EHH values, and the *GHR*-*PRLR* region at 32–39 Mb had the highest concentration of long haplotypes with high EHH values in contemporary Holsteins (Group III) (Additional file 3: Figure S3b, Additional file 4, Additional file 5). These results showed that changes in haplotype frequencies and EHH distances were important consequences of genetic selection.

### Long-range frequency changes

Because of the virtually complete coverage of the 28 Mb region of Chr20 by long haplotypes with high frequencies and EHH values, we mostly used long-range frequency differences to identify chromosome regions affected by selection. The comparison of long-range frequency differences between the selected and unselected cattle used long-range standardized AFD (*Z*_*AFD*_) [32] and long-range standardized heterozygosity differences (*Z*_*HD*_) [7, 32]. A genome region with *Z*_*AFD*_ ≥3 or |*Z*_*HD*_| ≥ 3 in sliding windows of 0.5–3.0 Mb was considered a selection signature, and the size of the selection signature was defined as the region with *Z*_*AFD*_≥2 on both sides of the peak *Z*_*AFD*_ value or |*Z*_*HD*_| ≥ 2 on both sides of the peak |*Z*_*HD*_| value. The results of these long-range analyses are shown in Additional file 6 for the 40 years of selection, and the first and second 20 years of selection; and are shown in Additional file 7 for the comparison between an elite group (Group IIIb) and the other groups (Groups I, II and IIIa).

**Fig. 4:**
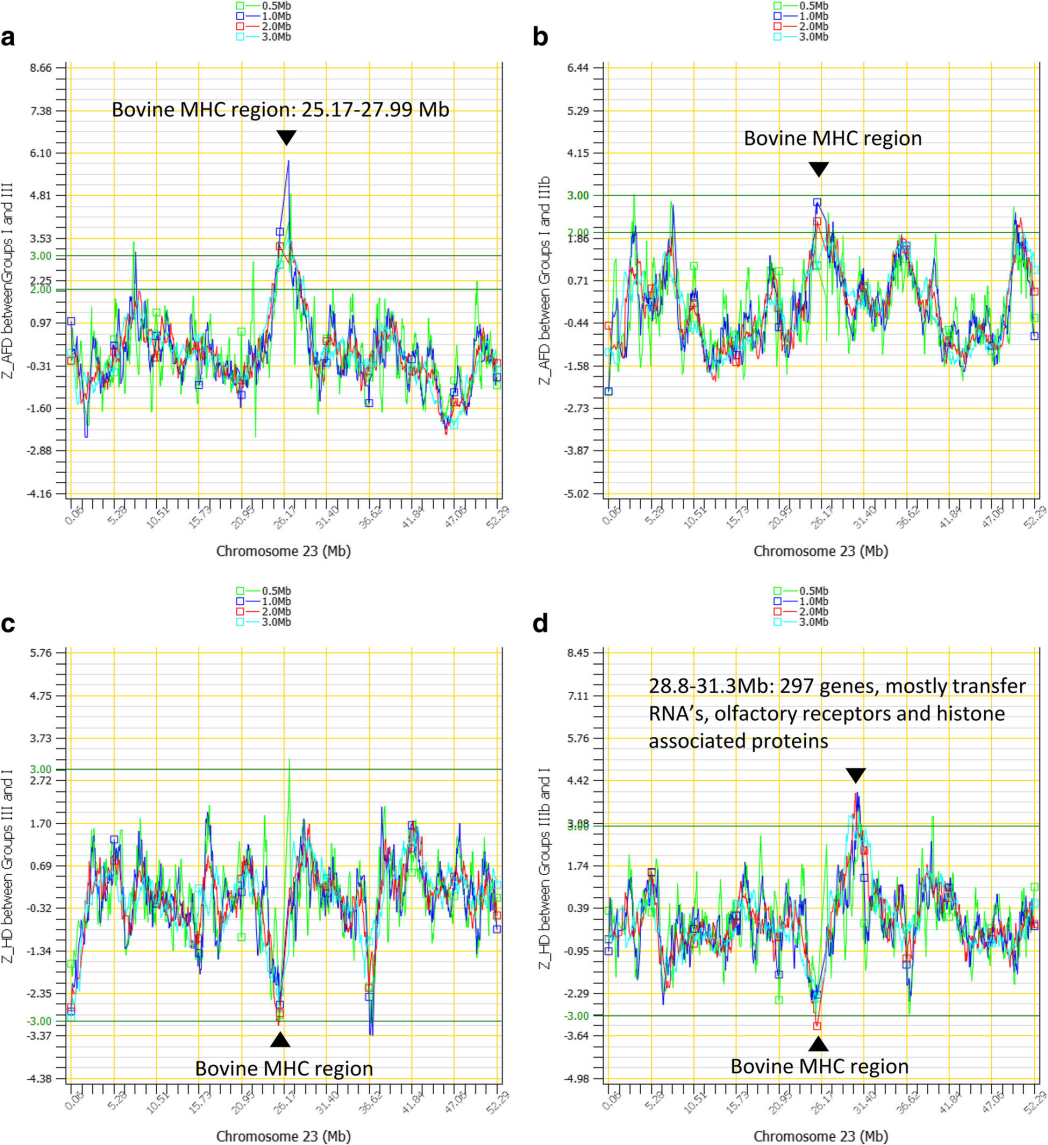
Chromosome 23 had strong selection signatures identified by long-range frequency changes. **a**. The bovine MHC region had the largest long-range AFD between Groups I and III due to the 40 years of selection. **b**. The bovine MHC region also had large long-range AFD between Groups I and IIIb (the elite group) due to the 40 years of selection. **c**. Contemporary Holsteins (Group III) had long-range heterozygosity decreases in the MHC region relative to Holsteins unselected since 1964 (Group I). **d**. The elite group (Group IIIb) also had long-range heterozygosity decreases in the MHC region but had heterozygosity increases in the 28.8–31.3 Mb region with a large cluster of 297 genes

Chr23 had one of the strongest selection signatures with long-range frequency changes in the 25.17–27.99 Mb region or the *ELOVL5-DDR1* region, which was slightly smaller than the bovine MHC region from *BOLA-DQA1* to *BOLA-A* (25.16–28.51 Mb). The *Z*_*AFD*_ in the MHC region was one of the largest long-range AFD between Groups I and III (Fig. 4a-b). The *Z*_*HD*_ analysis showed that the contemporary Holstein cattle (Group III) had a significant decrease in heterozygosity in the MHC region relative to the Holstein cattle unselected since 1964 (Group I) (Fig. 4c). The elite group (Group IIIb) also had heterozygosity decrease in the MHC region but had heterozygosity increase in a region with a large cluster of 297 genes (Fig. 4d). The comparison between Groups I and III as well as between groups II and III (Additional file 7) showed that the MHC region had significant long-range AFD for the first 20 years and the second 20 years of selection. These results showed that the MHC region was subjected to selection during the 40-year period since 1964. We previously reported that the center regions of the bovine chromosomes had lower recombination rates than the other regions, and the center regions of the Chr23 including the MHC region had the lowest recombination rates in both males and females among the center regions of all chromosomes [33]. Consequently, selection at a few locations in or near the MHC region could result in strong hitchhiking by neighboring loci and a highly visible selection signature for the entire MHC region due to the lack of recombination.

### Fertility genes affected by genetic selection

We identified 234 genome regions with signature of selection mostly based on the results of long-range frequency differences (Additional file 8). For each of the 234 chromosome regions affected by selection, we searched for genes with documented fertility functions that could explain the unintended declines in fertility during the 40 years of selection for milk production since 1964. Of these 234 genome regions, 125 regions either contained or were in the proximity of 198 genes with documented fertility functions (Additional file 9), including 76 genes for male fertility, 99 genes for female fertility, and 23 genes for fertility in both males and females. These fertility genes included all the fertility genes in Table 1 except *ADGRG2*, *AVEN* and *ERBB4*. The *ADGRG2* gene was about 1.0 Mb upstream of a selection signature, *AVEN* was 1.4–2.5 Mb between two selection signatures, and *ERBB4* was 1.5 Mb upstream of a selection signature. The male and female fertility genes affected by selection had an approximately 3:4 male-female ratio (Additional file 10), indicating that today’s dairy fertility problems were due to declined fertility in both males and females. The 198 genes affected many aspects of male and female fertility, including completion of meiosis; testis development; spermatogenesis and spermiogenesis; semen mobility and morphology; follicle, oocyte and embryo development; embryo implantation and survival; placenta development; uterine receptivity and environment; miscarriage and premature ovarian failure; pregnancy rate; and the joint male-female fertility function of fertilization and sperm-egg fusion (Additional file 9). These many functions of the fertility genes provided many possible genetic reasons for the decline in fertility in contemporary Holsteins and pointed to the complex and polygenic nature of the fertility traits.

To evaluate the potential association between the decreased fertility and the fertility genes affected by selection, we calculated the correlation between the SNP effects on milk yield and daughter pregnancy rate from the April 2017 CDCB/USDA genomic evaluation. At the whole genome level, the correlation coefficient between the SNP effects of milk yield and daughter pregnancy rate was r = − 0.27, indicating that selection for increased milk yield had a tendency to decrease daughter pregnancy rate and that antagonistic pleiotropy effects between milk yield and daughter pregnancy rate existed. The SNP effects of milk yield and daughter pregnancy rate in the 234 selection signatures had a negative correlation of *r* = − 0.27. The 109 selection signatures without known fertility genes had a correlation coefficient of *r* = − 0.21, and the 125 selection signatures with known fertility genes had a correlation coefficient of *r* = − 0.30 between SNP effects of milk yield and daughter pregnancy rate (Table 2). These results supported the hypothesis that the hitchhiking of genetic selection for milk yield since 1964 by negative effects of fertility genes contributed to the unintended decline in fertility of contemporary Holstein cattle, along with the antagonistic pleiotropy between milk yield and daughter pregnancy rate.

**Table 2:** Correlation between SNP effects of milk yield and daughter pregnancy rate (SNP effects were from the April 2017 CDCB/USDA genomic evaluation)

	Whole-genome^a^	234 selection signatures^a^	109 signatures without known fertility genes^a^	125 signatures with known fertility genes^a^
Number of SNPs	60,671	3893	1555	2338
Correlation	−0.27	−0.27	−0.21	−0.30

^a^ The 1,463,676–2,138,926 region of Chr14 containing *DGAT1* is not included in the calculation of correlation between milk yield and daughter pregnancy rate, because the extremely negative milk effect of *DGAT1* drastically reduced the correlations. With this region, the correlation between milk yield and daughter pregnancy rate reduced to − 0.17 from − 0.27 for the 234 regions with selection signature, to − 0.09 from − 0.21 for the 109 regions without known fertility genes, to − 0.17 from − 0.30 for the 125 regions with known fertility genes, and to − 0.26 from − 0.27 for the whole-genome correlation. All correlations were statistically significant with *p* < 0.0001

### Immunity genes affected by genetic selection

We identified 67 immunity genes (including all immunity genes in Table 1) and several large clusters of genes with documented immunity functions in or near chromosome regions affected by selection (Additional file 11**)**. These results indicated that the 40 years of genetic selection affected immunity genes due to hitchhiking of genetic selection. The bovine MHC region from *BOLA-DQA1* (Class-II) to *BOLA-A* (Class-I) was within the strongest selection signature at 25.16–27.86 Mb of Chr23 identified by long-range frequency changes (Fig. 4). The human MHC region is known to be associated with many diseases [34, 35]. Some of the genes with immunity functions also had fertility functions (Additional file 9, Additional file 11). Large gene clusters with immunity functions affected by selection included a cluster of 135 T-cell receptor genes and 33 immunoglobulin genes at the 22.1–25.5 Mb region of Chr10, and a cluster of 33 cationic amino acid transporter genes and nine sialic acid binding immunoglobulin like lectin genes at the 58.70–61.42 Mb region of Chr18.

### Selection in the 1.46–2.14 Mb region containing *DGAT1*

**Fig. 5:**
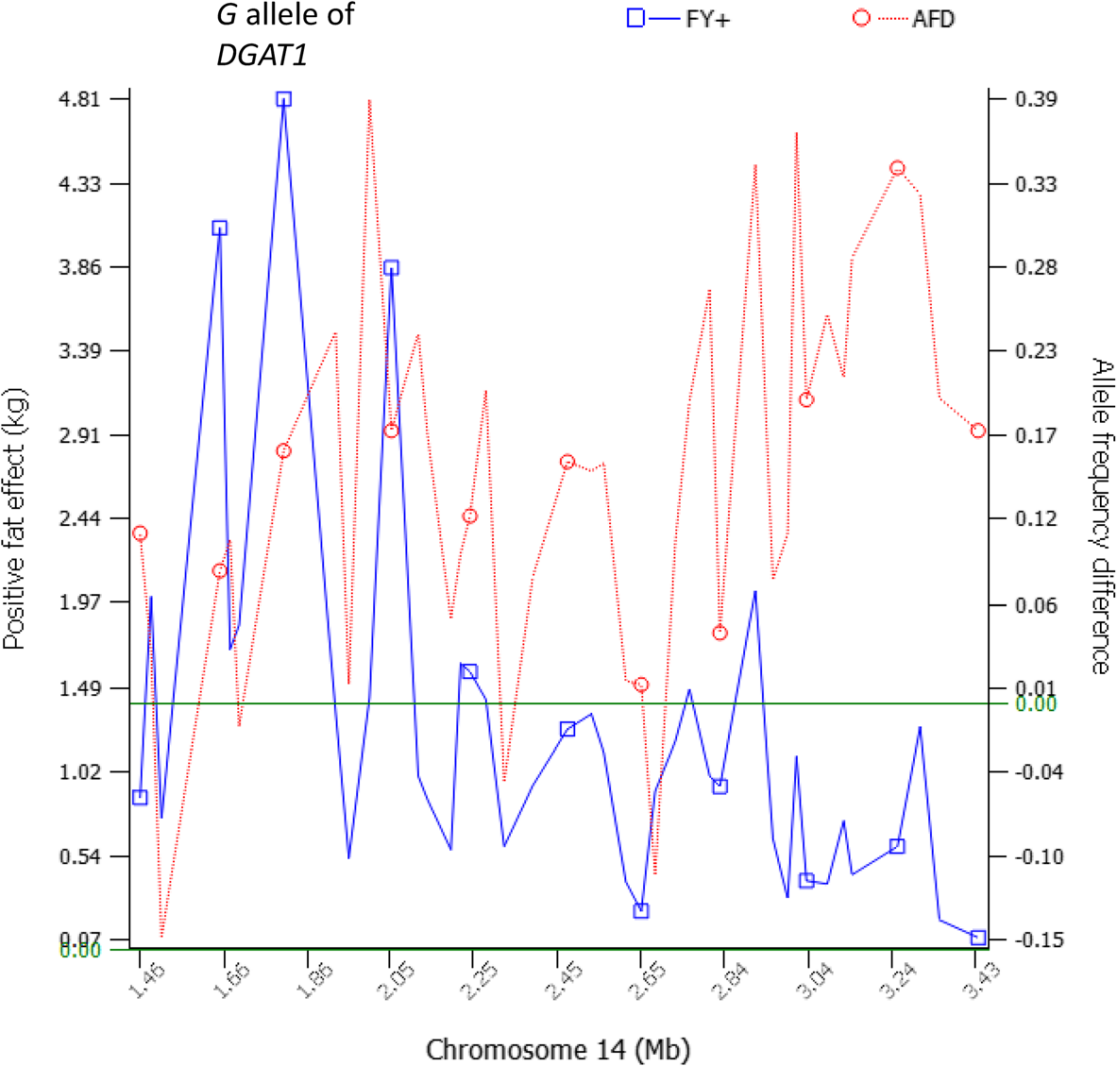
The positive fat effects of *DGAT1* and neighboring SNPs in the 2.08 Mb region of 1,379,063-3,464,083 bp were subjected to positive selection. Of the 41 SNPs present in both the 294,079 cows of the current Holstein population and the unselected Holsteins (Group I), 37 SNPs had higher frequencies (AFD > 0) in the 294,079 cows of the current Holstein population than in the unselected Holsteins for the alleles with positive effects on fat yield and negative effects on milk and protein yields. FY+ = positive fat effect. AFD = allele frequency difference between the unselected Holsteins and the 294,079 cows in the current Holstein population

The widely confirmed *DGAT1* effects [27, 36–39] provided an ideal model to study the relationship between selection and QTL effects. The 2.08 Mb region containing *DGAT1* had the best consistency between positive allelic effects and allele frequency increases among all regions with selection signature and QTL effects. The only SNP in *DGAT1* (*rs109421300*) in our SNP data has *A* and *G* alleles. The *G* allele had extreme antagonistic pleiotropic effects among all SNPs with the highest fat yield and the lowest milk and protein yields, whereas the *A* allele had antagonistic pleiotropy between negative fat yield and positive milk and protein yields but this antagonistic pleiotropy was not nearly as strong as the antagonistic pleiotropy of the *G* allele [29]. Long-range frequency analysis showed that the 1.46–2.14 Mb region containing *DGAT1* was subjected to selection during the first 20 years of selection (*Z*_*AFD*_ = 3.36 between Groups I and II, Additional file 6). The allele frequencies of *DGAT1* remained relatively unchanged since the mid-1980’s (Table 1), consistent with the result of long-range AFD that the *DGAT1* region was subjected to selection during the first 20 years only. During the first 20 years, selection of *DGAT1* was for the positive fat effect of *DGAT1*, because the *G* allele had the most significant and largest positive effect on fat yield and had a higher frequency (0.29) among the Holsteins subjected to 20 years of selection (Group II) than in the 1964 Holsteins (0.07 in Group I). The selection for positive fat effects extended in the surrounding regions of *DGAT1*. In the 2.08 Mb region of 1,379,063-3,464,083 bp, 42 SNPs were present in a large sample of Holstein cows [29] and the unselected Holsteins (Group I). Of the 42 SNPs, 38 SNPs had higher frequencies in the 294,079 cows of the current Holstein population than in the unselected Holsteins for the alleles with positive effects on fat yield and negative effects on milk and protein yields (Fig. 5). The AFD results of *DGAT1* in Table 1 and the long-range frequency results (*Z*_*AFD*_ = 3.36 between Groups I and II, Additional file 6) identified the time of selection in and around *DGAT1* for positive fat effects to be 1964–1985. Genetic selection in this period was mainly on milk and fat yields, which were the only traits for genetic evaluation during 1936–1976, noting that protein yield was added for bull evaluation and type traits added in 1977, and protein yield was evaluated for both bulls and cows since 1985 [30].

## Discussion

The results in this study identified genomic regions affected by genetic selection since 1964 and revealed that negative effects of fertility genes hitchhiking genetic selection for milk production was a contributing factor to the unintended decline in fertility that accompanied genetic selection for milk production since 1964 as shown by the genetic trends since 1964. This result was consistent with a previous analysis about possible links between selection and decreased fertility [40]. The unintended consequences of genetic selection was due to genome landscape changes and due to the mixtures of genes affecting production, fertility and immunity in chromosome regions affected by selection. The genome landscape changes due to the 40 years of genetic selection in Holstein cattle included genome-wide allele frequency changes and associated heterozygosity changes, haplotype changes, and localized frequency changes that created ‘peaks’, ‘hills’ and ‘valleys’ of frequency differences between the selected and unselected Holsteins. As a consequence of genome landscape change, the selection for a specific chromosome location also caused unintended changes of the neighboring chromosome regions. Many of the chromosome regions targeted by genetic selection for milk production also contained or were in the proximity of genes affecting fertility and immunity. The stronger negative correlation between milk yield and daughter pregnancy rate in selection signatures with fertility genes offered a direct measure supporting the hypothesis that the hitchhiking of genetic selection for increased milk production by negative fertility effects contributed to the fertility declines since 1964. Given that immunity genes also were present in some selection signatures due to selection for increased milk production, we also hypothesize that the hitchhiking of genetic selection for increased milk production by negative immunity effects contributed to the presumed immunity declines indicated by increased veterinary expenses in contemporary Holsteins. The genome landscape changes involved different chromosome regions in different time periods with new and old ‘peaks’ of allele frequency changes. Examples of new peaks emerged in the second 20 years included Chr06 selection signatures at 88.74–88.82 Mb downstream of *GC* and at 37.63–38.41 Mb or the *HERC5-IBSP* region. Examples of old peaks created by the first 20 years of selection included the 55.5–56.2 Mb of Chr08 with *TLE4*, the 43.6–44.4 Mb of Chr16 with 18 genes, the 34.0–34.4 Mb of Chr22 with *SUCLG2*, the 32.4–33.7 Mb of chr27 with 34 genes, and the 1.46–2.14 Mb region of Chr14 containing *DGAT1* (Additional files 5 and 6).

The direct comparison of the unselected and selected Holstein genomes revealed complex genetic factors affecting dairy phenotypes, including the many aspects of fertility affected by the fertility genes, large gene clusters affected by selection, and strong selection signatures in the center regions of some chromosomes. The many aspects of fertility affected by the fertility genes provided a potential reason for the low heritability of the three fertility traits of daughter pregnancy rate, cow conception rate and heifer conception rate (0.01–0.08) [20]: the decreased fertility of different cows could be due to different genetic factors that each accounted for a small fraction of the phenotypic variation. The genome landscape change involved large gene clusters. These large clusters included olfactory receptors at 58.07–60.54 Mb of Chr05 and at 79.0–82.0 Mb of Chr15; T-cell receptors at 21.5–22.0 Mb of Chr10; microRNA genes at 67.3–67.8 Mb of Chr21; transfer RNA’s, olfactory receptors and histone associated proteins at 28.8–31.3 Mb of Chr23; and zinc fingers, cationic amino acid transporters, sialic acid-binding Ig-like genes, and vomeronasal receptors at 58.7–61.4 Mb of Chr18 (Additional file 8). Several autosomes had selection signatures in the center of those chromosomes, including Chr23, Chr07, Chr08, Chr12, Chr16, and Chr20. The bovine MHC region of Chr23 with the strongest selection signature identified by long-range frequency analysis was in the center region. The center regions of cattle autosomes generally had lower recombination rate than in other regions, with Chr23 having the lowest recombination rate in the center of the chromosome among all center regions [33]. The decreased recombination rates in those center regions should have enhanced the hitchhiking effects of fertility and immunity genes in those center regions. Consequently, the same selection pressure should have resulted in more severe unintended selection responses in those center regions than in other regions. These results of genome landscape changes involving dense genes and reduced recombination rate indicated that the unintended consequences of genetic selection could have been more severe in these regions than in gene-sparse regions.

The elite group of 160 contemporary cows (Group IIIb) was from a half-sib family sired by a single bull. Of these 160 cows, 153 were classified into the upper-right cluster in Fig. 1. This group of cows had high milk production, low somatic cells in the milk, high daughter pregnancy rate and least calving problems relative to the remaining contemporary cows (Group IIIa) [41]. A striking difference between these two sub-groups was the increased heterozygosity in the elite group. The elite group was more heterozygous than the remaining contemporary cows for all 29 regions with significant differences in long-range heterozygosity between these two groups (*Z*_*HD*_≥3, blue triangle in right column of Additional file 7). The highest heterozygosity of the elite group over all the other groups was in a region containing *BMP15* on the X chromosome. The *LPPR1-CYLC2-SMC2* region of Chr08 had one of the strongest heterozygosity increases, noting that *SMC2* was shown to be the causal gene of Holstein lethal haplotype 3 [42]. Several of the highly heterozygous regions in the elite group involved large gene clusters, including olfactory receptors at 58.07–60.54 Mb of Chr05; olfactory receptors and *EMR2* receptors at 9.2–12.2 Mb of Chr07; zinc fingers, cationic amino acid transporters and sialic acid-binding Ig-like genes at 57.58–62.53 Mb of Chr18; and transfer RNAs, olfactory receptors and histone associated proteins at 28.8–31.3 Mb of Chr23 (Additional file 8). The most likely reason for the higher heterozygosity in the elite group is that the sire of the half-sib cows in the elite group carried rare alleles associated with low heterozygosity in the other populations. The daughters received half of their genomes from the sire, and the frequencies of the rare alleles among those daughters would be at least 50% if the sire was homozygous for the rate allele and at least 25% if the sire was heterozygous, resulting in higher heterozygosity than in populations with rare allele frequencies.

This study focused on genetic selection since 1964, the earliest time period for which we have DNA samples for the 1964 Holstein line. Apparently, genetic selection had been going on before 1964 because the USDA dairy genetic evaluation for milk and fat yields started in 1936 [30]. Without direct genome comparison between the 1964 Holsteins and those before 1964, LD based analysis such as the EHH analysis in the 1964 Holstein line becomes the primary analysis to identify selection signature due to selection before 1964. The EHH analysis showed that the 1964 Holstein line had 202 long-range haplotypes ≥1.50 Mb with high EHH values (≥0.60), in comparison with 653 such haplotypes in contemporary Holsteins (Group III) (Fig. 2b). Therefore, some of the 202 long haplotypes with high EHH values could be due to selection before 1964.

## Conclusions

Genetic selection in Holstein cattle since 1964 resulted in genome-wide changes in allele frequencies, heterozygosity, and haplotype frequencies and distances with localized selection signatures in the bovine MHC region and chromosome regions containing genes with known fertility and immunity functions. The selection signature results and the correlation between SNP effects of milk yield and daughter pregnancy rate supported the hypothesis that the hitchhiking of genetic selection for milk yield by negative fertility effects contributed to the decline in Holstein fertility. The selection signature analysis revealed genetic selection for alleles with positive effects on fat yield in and around *DGAT1*, showing that the integration of selection signature analysis with association analysis may help understand the genetic mechanism of genome variants associated with phenotypes. The selection signature analysis also revealed potential involvement of complex genetic mechanisms in the artificial selection since 1964 as indicated by the presence of large clusters of microRNA genes, olfactory receptors, zinc fingers, cationic amino acid transporters, sialic acid-binding Ig-like genes, vomeronasal receptors, keratin genes, *EMR2* receptors, transfer RNA’s and histone associated proteins in genome regions affected by selection.

## Methods

### Holstein populations and genotyping data

Three groups of Holstein cattle representing three periods of artificial selection were analyzed for signatures of selection. Group I with 317 cattle represented Holstein genomes of the 1950’s and was used as a sample of the unselected Holstein genomes since 1964. This group included 228 cows, 10 of the 20 founder bulls born in 1951–1959, 63 sons of the 20 founder bulls of the University of Minnesota Holstein control line unselected since 1964, and 16 bulls born in 1954–1959 unrelated to the control line. Group II consisted of 215 bulls born between 1975 and 1985 representing 20 years of selection since 1964, and Group III consisted of 1654 contemporary cows representing 40 years of artificial selection for increased milk production, including 149 contemporary Holstein cows maintained at the University of Minnesota. These 149 contemporary cows and the 228 control line cows were maintained at the Southern Research and Outreach Center of the University of Minnesota in Waseca, Minnesota. Other contemporary cows were maintained at several industry and university facilities. Group III had an elite group consisting of 160 half-sibs from a single sire [41] that were defined as Group IIIb, while the rest of Group III were defined as Group IIIa. The differences between Groups I and II, II and III, and I and III reflected genome changes during the first 20 years, the second 20 years, and the entire 40 years of selection since 1964, respectively. The difference between Group IIIb and the other groups reflected potential genomic features unique to the elite group. Although the sample size of the unselected Holsteins since 1964 (Group I, *n* = 317) was smaller than the sample size of the contemporary Holsteins subjected to 40 years of selection (Group III, *n* = 1654), the unselected Holsteins had lower inbreeding coefficients than contemporary Holsteins and had insignificant genetic drift [12]. The SNP genotypes were obtained from the Illumina BovineSNP50™ BeadChip. A total of 45,878 SNPs (50 K) on the 29 autosomes and the X chromosome with a minimal allele frequency difference of 0.02 between the contemporary and unselected groups (Groups I and III) were used in the analysis of single-SNP allele frequencies. The long-range analysis of selection signature used 45,451 SNPs after removing SNPs with unknown chromosome positions. The sample for calculating allele frequencies in the current Holstein population contained 294,079 first lactation cows with 98.4% of the cows born in 2006–2015, and 60,671 SNPs was used for calculating allele frequencies for comparison with the unselected Holsteins. Genome differences between the current Holstein population and the unselected Holsteins since 1964 approximately reflected genome changes due to 50 years of artificial selection. The 294,079 cows in the current Holstein population and the three groups for selection signature analysis had 40,593 overlapping SNPs. The SNP data for groups I, II and III were generated by this study. The SNP data for the 294,079 cows in the current Holstein population were from the collaborator’s database at Council for Dairy Cattle Breeding (https://www.uscdcb.com/). The SNP data of the 301 cows and bulls in the University of Minnesota control line as well as the historical pedigree of the control line are available from (Additional file 12).

### Data analysis

**Fig. 6:**
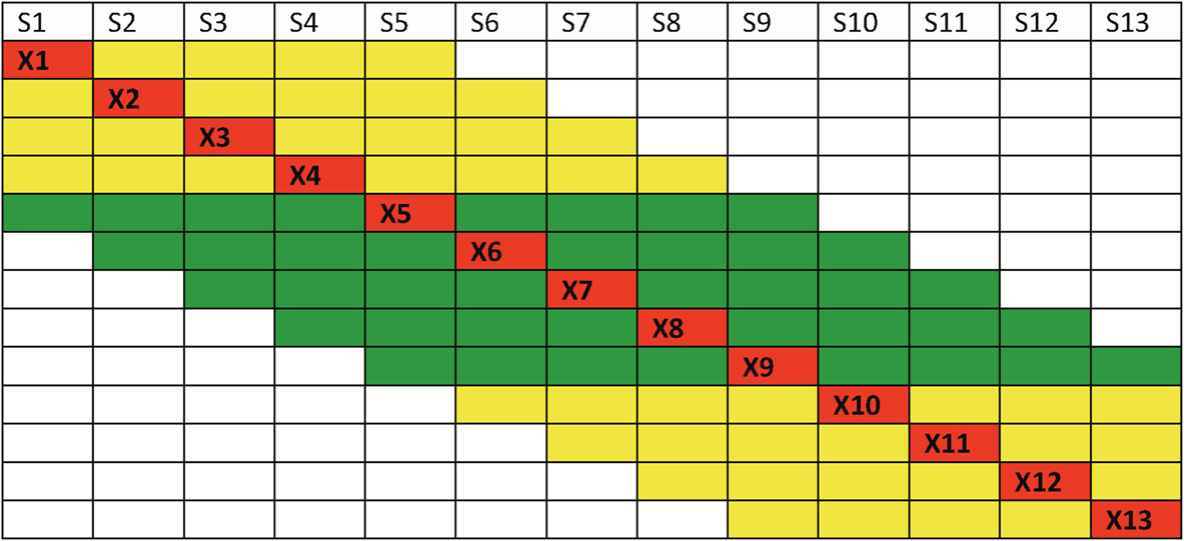
Calculation of long-range frequencies. For a sliding window with a fixed number of SNPs, a red cell flanked by green cells utilized equal numbers of single-SNP frequencies (allele frequency or heterozygosity of each SNP) on both sides of the red cell, whereas a red cell flanked by yellow cells utilized unequal numbers of single-SNP frequencies on the two sides of the red cell towards the two ends of the chromosome. For sliding windows with fixed chromosome distance, a red cell flanked by green cells utilized single-SNP frequencies in half the window size on both sides of the red cell whereas a red cell flanked by yellow cells utilized single-SNP frequencies of unequal chromosome distances on the two sides of the red cell towards the two ends of the chromosome

Selection signature analysis used long-range frequency analysis and extended haplotype homozygosity (EHH) analysis. The EHH distances and probabilities as well as bifurcation figures showing haplotype changes away from the core region were produced by Sweep 1.1 [6] for the unselected Holsteins (Groups I) and selected Holsteins (Groups II and III) as evidence of selection signature. A haplotype with a higher haplotype frequency and EHH probabilities for longer distances in the selected population than in the unselected population was considered to have been subjected to positive selection. The long-range frequency analysis included standardized long-range heterozygosity difference (|*Z*_*HD*_|) [7, 32] and standardized long-range allele frequency difference (*Z*_*AFD*_) [32] in sliding windows of SNP markers for genome-wide scan of selection signatures. The standardized frequency (*Z*_*AFD*_ or *Z*_*HD*_) was calculated based on each long-range frequency (X_j_) as shown in Fig. 6, where each red cell is a long-range frequency. For a sliding window with a fixed number of SNPs, a red cell flanked by green cells utilized equal numbers of single-SNP frequencies on both sides of the red cell, whereas a red cell flanked by yellow cells utilized unequal numbers of single-SNP frequencies on the two sides of the red cell towards the two ends of the chromosome. For sliding windows with fixed chromosome distance, a red cell flanked by green cells utilized single-SNP frequencies in half the window size on both sides of the red cell whereas a red cell flanked by yellow cells utilized single-SNP frequencies of unequal chromosome distances on the two sides of the red cell towards the two ends of the chromosome. The long-range heterozygosity difference (HD) or AFD for SNP j was calculated as the average of HD or AFD of all SNPs in the sliding window for SNP j using the following formula:

1 $$ {\mathrm{X}}_{\mathrm{j}}=\frac{\sum_{\mathrm{i}={\mathrm{l}}_{\mathrm{j}}}^{{\mathrm{u}}_{\mathrm{j}}}{\mathrm{S}}_{\mathrm{i}}}{{\mathrm{u}}_{\mathrm{j}}-{\mathrm{l}}_{\mathrm{j}}+1} $$

where S_i_ = HD or AFD of marker i in the sliding window, i = l_j_, …, u_j_; l_j_ = marker index at the lower bound of the sliding window for marker j; u_j_ = marker index at the upper bound of the window for marker j. Standardized HD and AFD were calculated using the formula of standardized normal variable, i.e.,

2 $$ {\mathrm{Z}}_{\mathrm{j}}=\left[{\mathrm{X}}_{\mathrm{j}}-\left(\mathrm{mean}\kern0.17em \mathrm{of}\;{\mathrm{X}}_{\mathrm{j}}\right)\right]/\left(\mathrm{standard}\kern0.17em \mathrm{deviation}\kern0.17em \mathrm{of}\;{\mathrm{X}}_{\mathrm{j}}\right),\mathrm{j}=\mathrm{HD}\;\mathrm{or}\;\mathrm{AFD} $$

where the mean and standard deviation of X_j_ were calculated for each chromosome to account for different chromosomal averages and variations. A genome region with *Z*_*AFD*_ ≥3 or |*Z*_*HD*_| ≥ 3 in sliding windows of 0.5–3.0 Mb was considered a selection signature, and the size of the selection signature was defined as the region with *Z*_*AFD*_ ≥2 on both sides of the peak *Z*_*AFD*_ value or |*Z*_*HD*_| ≥ 2 on both size of the peak |*Z*_*HD*_| value. The long-range frequency analysis was implemented using the AGDP computer package [43, 44].

## Additional files

Additional file 1: **Figure S1.** Phenotypic changes due to selection since 1964. **a**. Milk yield. **b**. Daughter pregnancy rate. The genetic merit of milk yield increased but daughter pregnancy rate decreased steadily for the U.S. Holstein cows and the University of Minnesota (UMN) selected cows. The UMN cows unselected since 1964 remained relatively unchanged for milk yield and daughter pregnancy rate. (PDF 491 kb)

Additional file 2: **Figure S2.** The time trend of the first four MDS dimensions. Dimension 1 had a clear time trend, Dimensions 3 and 4 had no time trend, and Dimension 2 only had differences between unselected group (Group I) and the elite group (Group IIIb). (PDF 307 kb)

Additional file 3: **Figure S3.** Overview of allele frequency differences (AFD) and extended haplotype homozygosity (EHH). **a**. The Chr23 example of single-SNP AFD between selected and unselected Holsteins, showing that genetic selection resulted in genome-wide allele frequency changes but single-SNP AFD lacked interpretable patterns. Other chromosomes had similar single-SNP AFD patterns. **b**. Chr20 had the strongest EHH evidence of selection signature spanning center region 21–49 Mb. Most of the long-distance EHH values were concentrated in the *GHR-PRLR* region. The EHH distances were distances of haplotypes with minimal EHH of 0.6. (PDF 371 kb)

Additional file 4: **Figure S4.** Selection signature of the 21–49 Mb region of Chr20 by the analysis of extended haplotype homozygosity (EHH). Most selection signatures had high frequency haplotypes (≥0.30) and high EHH values (≥0.60) for long distances (≥1.8 Mb) in the Holsteins subjected to 40 years of selection (Group III), and these long haplotypes virtually covered the entire 21–49 Mb region. (PDF 1701 kb)

Additional file 5: **Figure S5**. Extended haplotype homozygosity (EHH) evidence of selection in three Holstein groups for all 30 bovine chromosomes. All autosomes had long-range EHH values indicating selection, but the center region of Chr20 had the highest concentration of long haplotypes with high EHH values. ‘I’ is Group I unselected since 1964. ‘II’ is Group II subjected to 20 years of selection, and ‘III’ is Group III subjected to 40 years of selection since 1964. The EHH distances were haplotype distances with minimal EHH value of 0.6. (PDF 611 kb)

Additional file 6: **Figure S6.** Long-range differences of allele frequencies and heterozygosity between unselected and selected Holsteins since 1964. Left column: 40 years of selection between Groups I and III. Middle column: the first 20 years of selection between Groups I and II. Right column: the second 20 years of selection between Groups II and III. Chr30 is the X chromosome. (PDF 19496 kb)

Additional file 7: **Figure S7.** Long-range differences of allele frequencies and heterozygosity between an elite group (Group IIIb) and the other groups in the selection signature analysis. Left column: the 40 years of selection between Groups IIIb and I. Middle column: the second 20 years of selection between Groups IIIb and II. Right column: the difference between the elite group and their contemporaries (Groups IIIb and IIIa). Chr30 is the X chromosome. (PDF 19946 kb)

Additional file 8: **Table S1.** Genome regions with signature of selection detected by long-range frequency differences in 0.5 Mb, 1 Mb, 2 Mb and 3 Mb sliding windows of SNP markers. (PDF 280 kb)

Additional file 9: **Table S2.** Fertility genes in or near chromosome regions subjected to genetic selection since 1964. (PDF 456 kb)

Additional file 10: **Table S3.** Genes with documented fertility functions in or near genome regions with signature of selection. (Summarized from Additional file 9: Table S2). (PDF 93 kb)

Additional file 11: **Table S4.** Immunity genes in or near chromosome regions subjected to genetic selection since 1964. (PDF 255 kb)

Additional file 12: **Data Set1.** SNP and pedigree data of the University of Minnesota Holstein control line unselected since 1964. (ZIP 5309 kb)

## Abbreviations

AFD: Allele frequency difference
bp: Base pair
Chr: Chromosome
EHH: Extended haplotype homozygosity
HD: Heterozygosity difference
Kb: Kilo bases
MAF: Minor allele frequency
Mb: Mega bases
MHC: Major histocompatibility complex
QTL: Quantitative trait loci
SNP: Single nucleotide polymorphism
