# Supplementary material for: Genome changes due to artificial selection in U.S. Holstein cattle
Source: BMC Genomics. 2019 Feb 11;20:128. doi: 10.1186/s12864-019-5459-x (PMC6371544; doi:10.1186/s12864-019-5459-x)
Supplement: Supplementary file 1 — Figure S1. Phenotypic changes due to selection since 1964. a. Milk yield. b. Daughter pregnancy rate. The genetic merit of milk yield increased but daughter pregnancy rate decreased steadily for the U.S. Holstein cows and the University of Minnesota (UMN) selected cows. The UMN cows unselected since 1964 remained relatively unchanged for milk yield and daughter pregnancy rate. (PDF 491 kb) [file 12864_2019_5459_MOESM1_ESM.pdf]

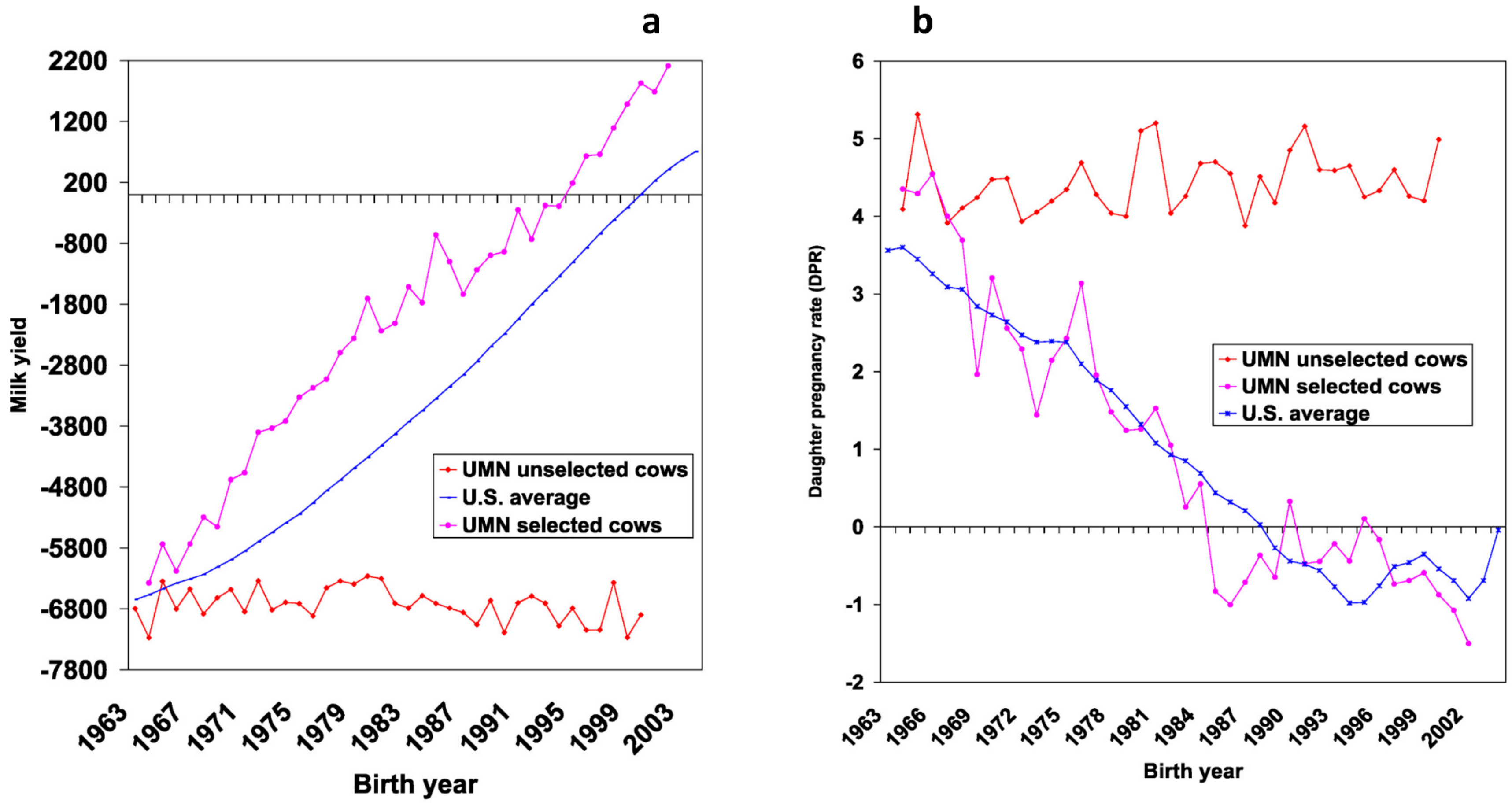

Fig. S1. Phenotypic changes due to selection since 1964. **a.** Milk yield. **b.** Daughter pregnancy rate. The genetic merit of milk yield increased but daughter pregnancy rate decreased steadily for the U.S. Holstein cows and the University of Minnesota (UMN) selected cows. The UMN cows unselected since 1964 remained relatively unchanged for milk yield and daughter pregnancy rate.
