# Supplementary material for: Genome changes due to artificial selection in U.S. Holstein cattle
Source: BMC Genomics. 2019 Feb 11;20:128. doi: 10.1186/s12864-019-5459-x (PMC6371544; doi:10.1186/s12864-019-5459-x)
Supplement: Supplementary file 2 — Figure S2. The time trend of the first four MDS dimensions. Dimension 1 had a clear time trend, Dimensions 3 and 4 had no time trend, and Dimension 2 only had differences between unselected group (Group I) and the elite group (Group IIIb). (PDF 307 kb) [file 12864_2019_5459_MOESM2_ESM.pdf]

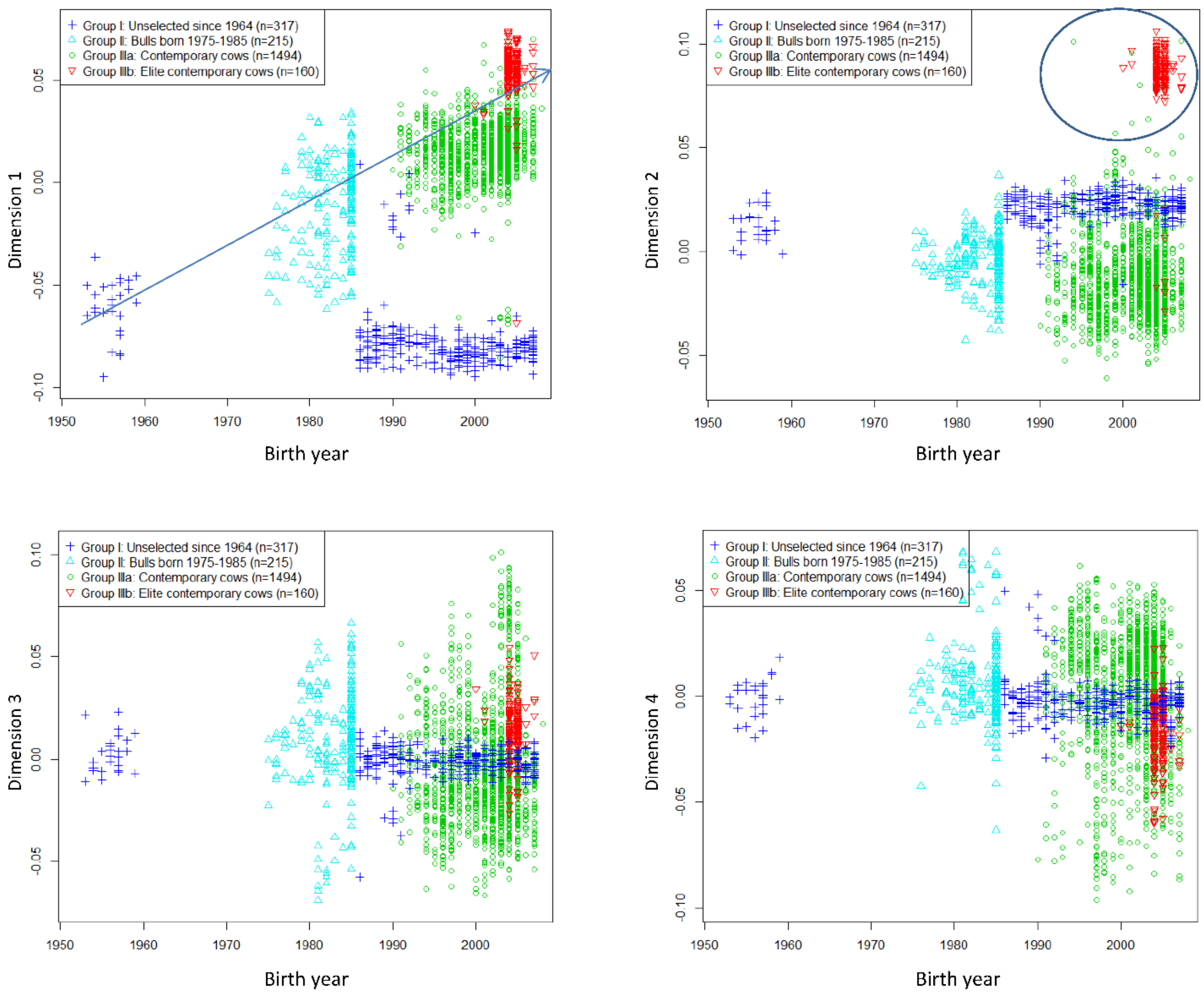

Additional file 2: Fig. S2. The time trend of the first four MDS dimensions. Dimension 1 had a clear time trend, Dimensions 3 and 4 had no time trend, and Dimension 2 only had differences between unselected group (Group I) and the elite group (Group IIIb).
