# Supplementary material for: Genome changes due to artificial selection in U.S. Holstein cattle
Source: BMC Genomics. 2019 Feb 11;20:128. doi: 10.1186/s12864-019-5459-x (PMC6371544; doi:10.1186/s12864-019-5459-x)
Supplement: Supplementary file 3 — Figure S3. Overview of allele frequency differences (AFD) and extended haplotype homozygosity (EHH). a. The Chr23 example of single-SNP AFD between selected and unselected Holsteins, showing that genetic selection resulted in genome-wide allele frequency changes but single-SNP AFD lacked interpretable patterns. Other chromosomes had similar single-SNP AFD patterns. b. Chr20 had the strongest EHH evidence of selection signature spanning center region 21–49 Mb. Most of the long-distance EHH values were concentrated in the GHR-PRLR region. The EHH distances were distances of haplotypes with minimal EHH of 0.6. (PDF 371 kb) [file 12864_2019_5459_MOESM3_ESM.pdf]

a

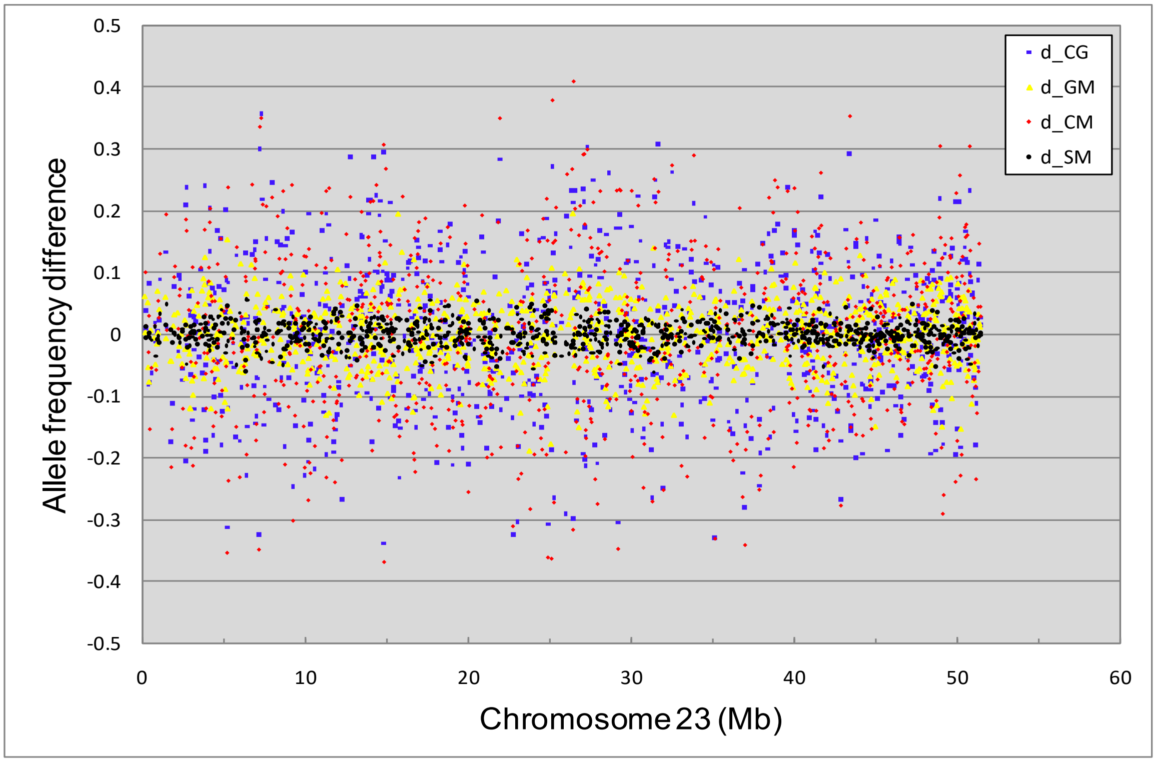
d_CG
d_GM
d_CM
d_SM
Allele frequency difference
0.5
0.4
0.3
0.2
0.1
0
-0.1
-0.2
-0.3
-0.4
-0.5
0
10
20
30
40
50
60
Chromosome 23 (Mb)

b

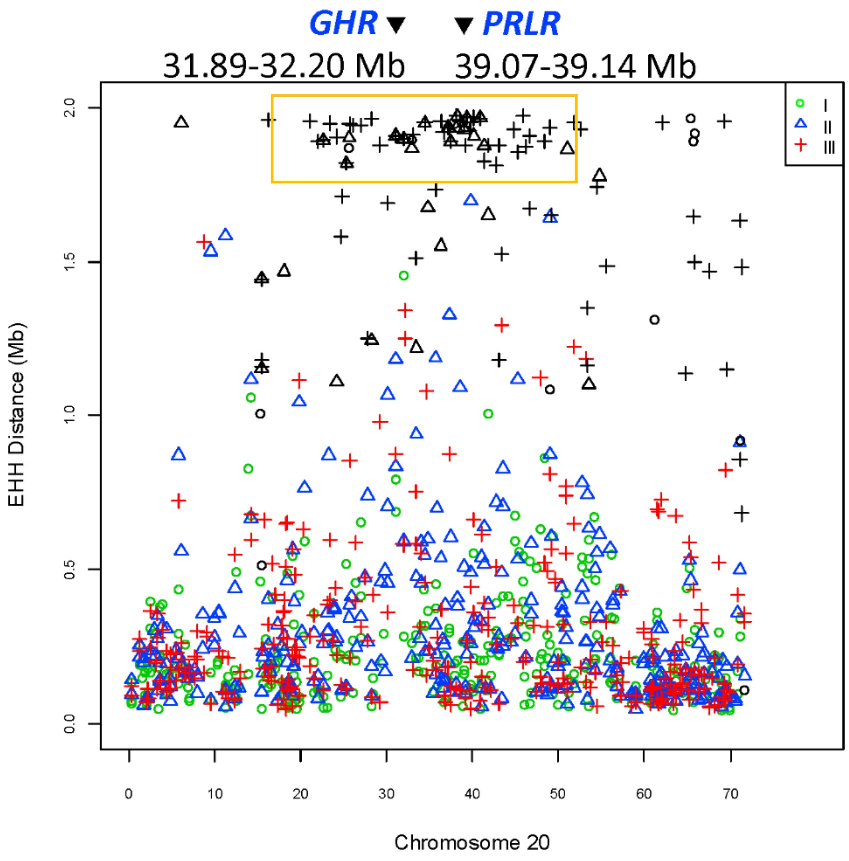

Additional file 3: Fig S3. Overview of allele frequency differences (AFD) and extended haplotype homozygosity (EHH). **a.** The Chr23 example of single-SNP AFD between selected and unselected Holsteins, showing that genetic selection resulted in genome-wide allele frequency changes but single-SNP AFD lacked interpretable patterns. Other chromosomes had similar single-SNP AFD patterns. **b.** Chr20 had the strongest EHH evidence of selection signature spanning center region 21-49 Mb. Most of the long-distance EHH values were concentrated in the *GHR-PRLR* region. The EHH distances were distances of haplotypes with minimal EHH of 0.6.
