# Supplementary material for: Genome changes due to artificial selection in U.S. Holstein cattle
Source: BMC Genomics. 2019 Feb 11;20:128. doi: 10.1186/s12864-019-5459-x (PMC6371544; doi:10.1186/s12864-019-5459-x)
Supplement: Supplementary file 4 — Figure S4. Selection signature of the 21–49 Mb region of Chr20 by the analysis of extended haplotype homozygosity (EHH). Most selection signatures had high frequency haplotypes (≥0.30) and high EHH values (≥0.60) for long distances (≥1.8 Mb) in the Holsteins subjected to 40 years of selection (Group III), and these long haplotypes virtually covered the entire 21–49 Mb region. (PDF 1701 kb) [file 12864_2019_5459_MOESM4_ESM.pdf]

<table>
<tr><th>Population</th><th>Core position & EHH values</th><th>Haplotype bifurcation figure</th><th>Haplotype frequency</th></tr>
<tr><td></td><td>chr20: 21018903-21107750</td><td></td><td></td></tr>
<tr><td>I</td><td>Not defined</td><td></td><td></td></tr>
<tr><td>III</td><td>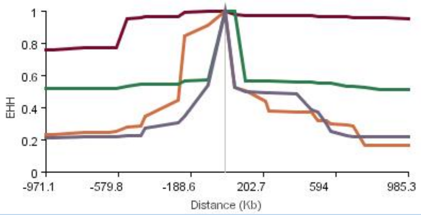
</td><td>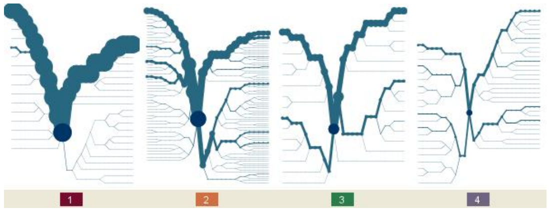
</td><td>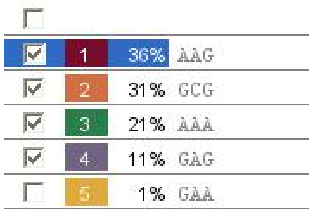
</td></tr>
<tr><td></td><td>chr20:21938158-22114399</td><td>LOC539789-GPBP1</td><td></td></tr>
<tr><td>I</td><td>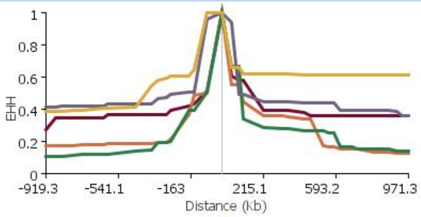
</td><td>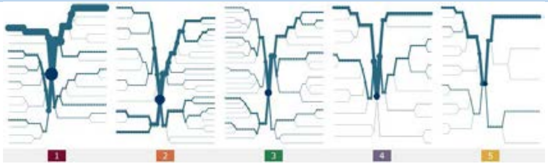
</td><td>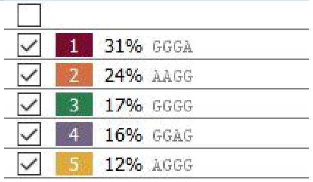
</td></tr>
<tr><td>III</td><td>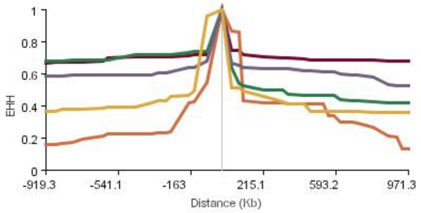
</td><td>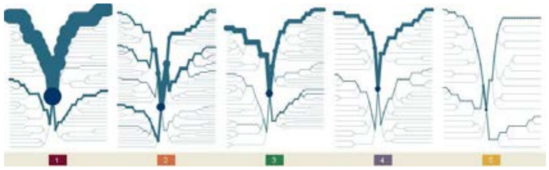
</td><td>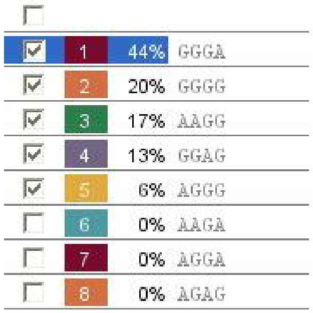
</td></tr>
<tr><td></td><td>chr20:22575532-22670366</td><td></td><td></td></tr>
</table>

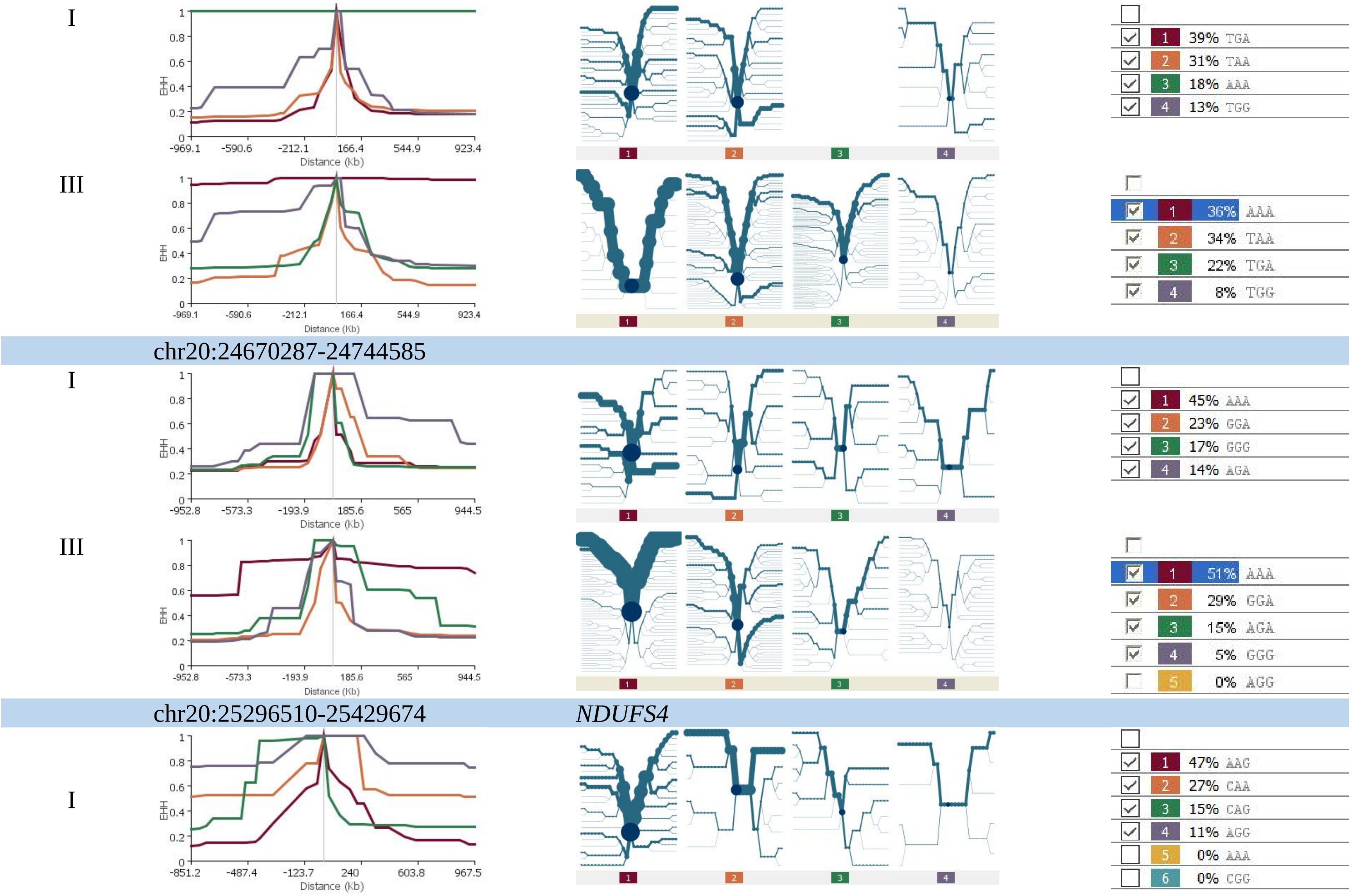
I
III
chr20:24670287-24744585
I
III
chr20:25296510-25429674
NDUFS4
I
EHH
Distance (kb)
-969.1
-590.6
-212.1
166.4
544.9
923.4
-952.8
-573.3
-193.9
185.6
565
944.5
-851.2
-487.4
-123.7
240
603.8
967.5
39% TGA
31% TAA
18% AAA
13% TGG
36% AAA
34% TAA
22% TGA
8% TGG
45% AAA
23% GGA
17% GGG
14% AGA
51% AAA
29% GGA
15% AGA
5% GGG
0% AGG
47% AAG
27% CAA
15% CAG
11% AGG
0% AAA
0% CGG

III

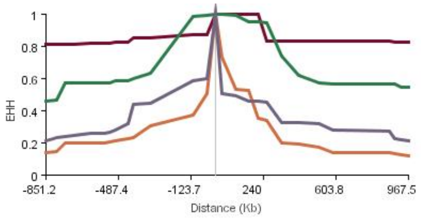

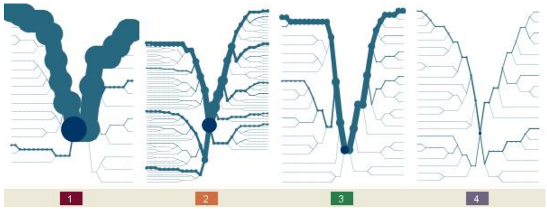

#	Frequency	Haplotype
1	50%	CAA
2	29%	AAG
3	16%	AGG
4	5%	CAG
5	0%	CGG
6	0%	AAA

chr20:26917749-27170720 Deletion: 27049039-27430926

I. chr20:26917749-27100488

I

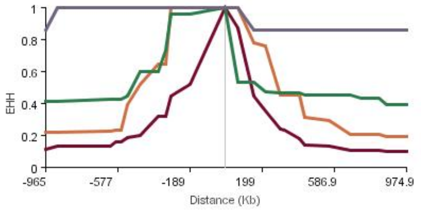

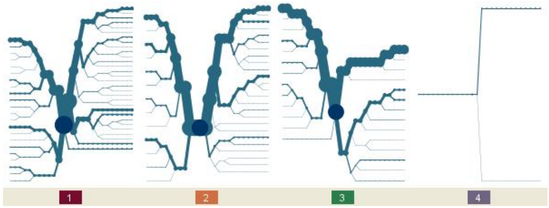

#	Frequency	Haplotype
1	35%	AAC
2	32%	CAC
3	31%	CGA
4	2%	CGC

III. chr20:26917749-27170720

III

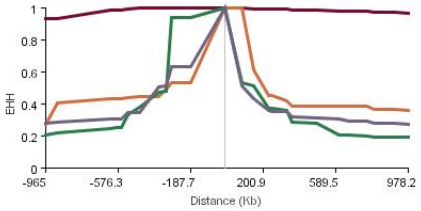

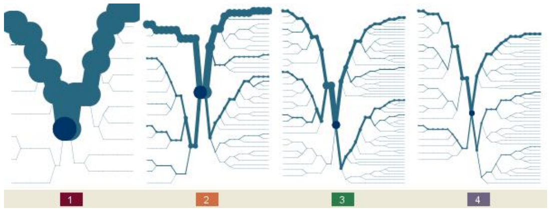

#	Frequency	Haplotype
1	46%	CGAA
2	26%	CGAG
3	15%	CACG
4	11%	AACG
5	1%	CACA
6	1%	AACA
7	0%	CGCG

chr20:28148900-28299434

I. chr20:28186842-28299434

I

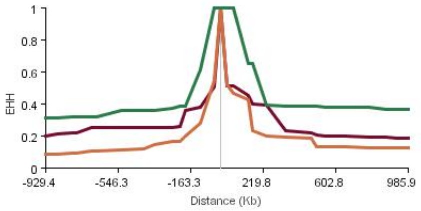

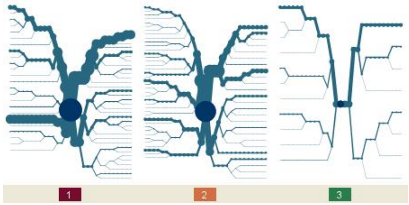

#	Frequency	Haplotype
1	45%	AGG
2	41%	AAA
3	13%	CGG

III. chr20:28148900-28299434

III

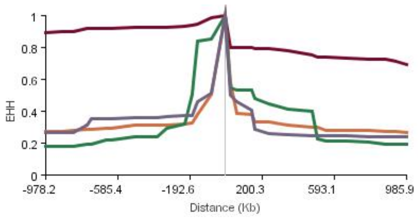

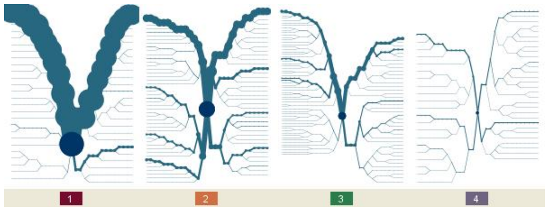

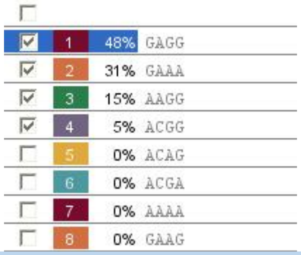

chr20:30928803-31151035 *LOC614206*

I

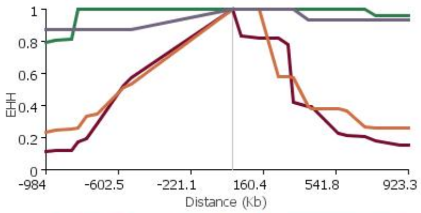

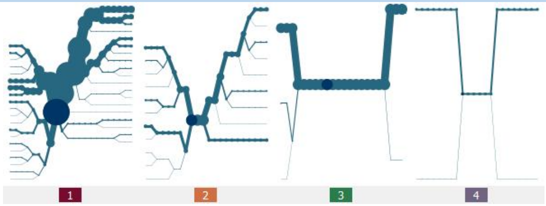

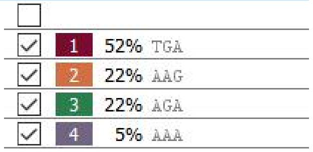

III

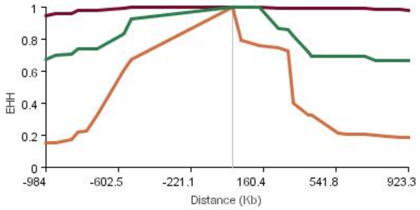

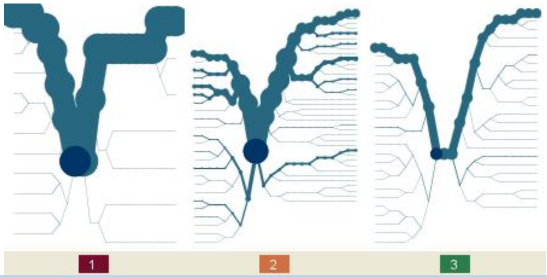

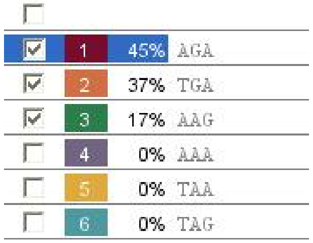

chr20:32074342-32360404 *GHR* (Ensembl)

I Not defined

III

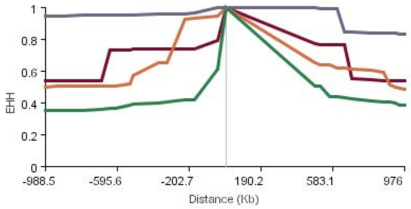

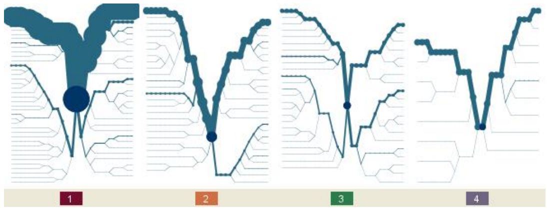

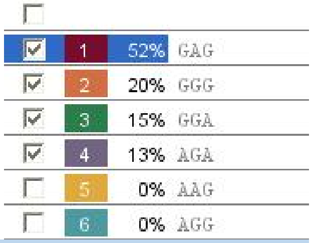

chr20:33007835-33223973 *PLCXD3*

I Not defined

III

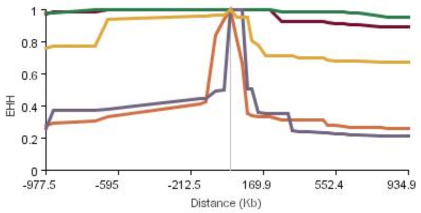

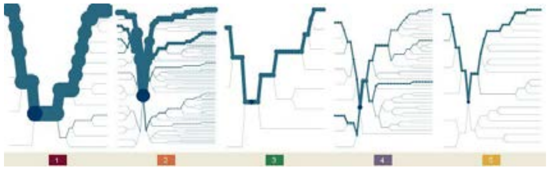

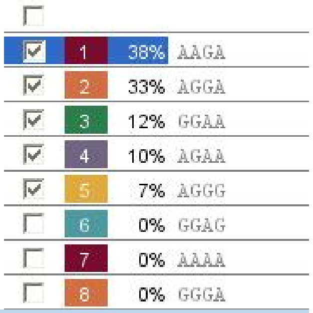

chr20:34416396-34474873

I Not defined

III

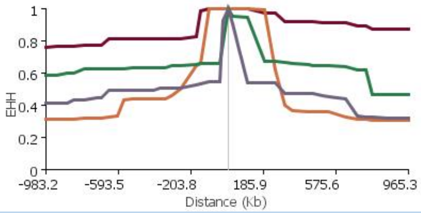

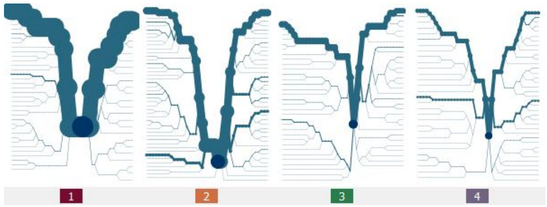

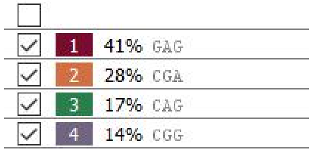

chr20:36561330-36674340 *GDNF*

I. chr20:36570529-36674340

I

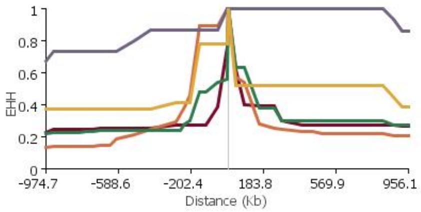

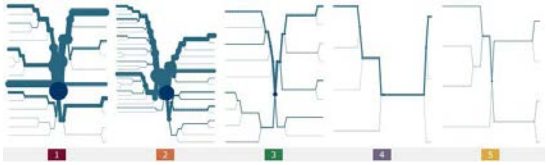

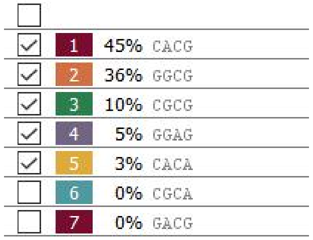

III. chr20:36561330-36674340

III

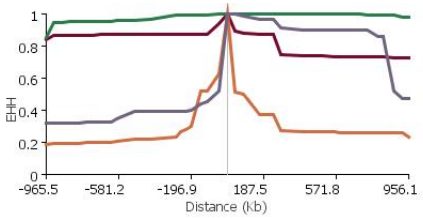

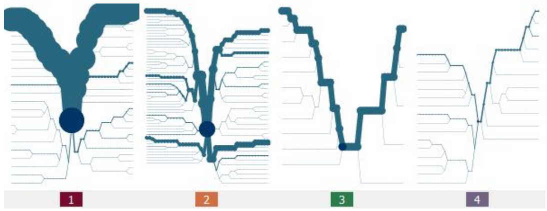

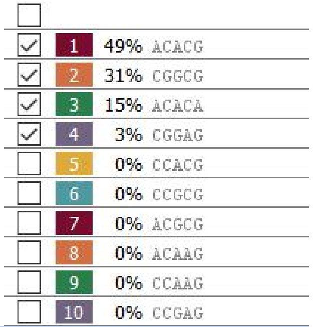

	chr20:37237425-37412305	*NIPBL*
I	Not defined	

III

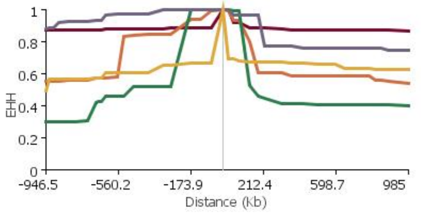

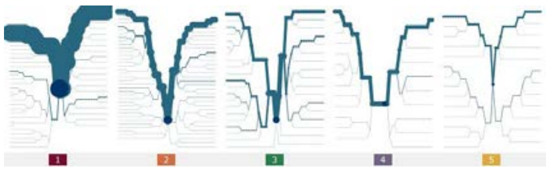

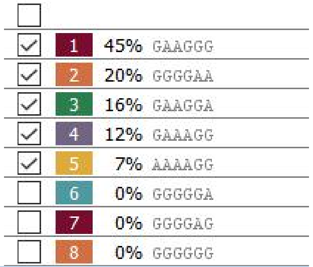

	chr20: 37939597-38252896	*RANBP3L, NADK2, SKP2, LMBRD2, UGT3A2, LOC525484*
I.	Not defined	

III.

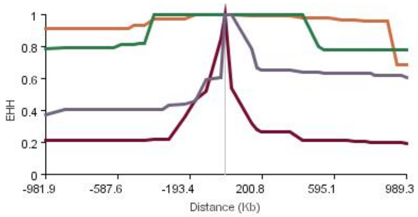

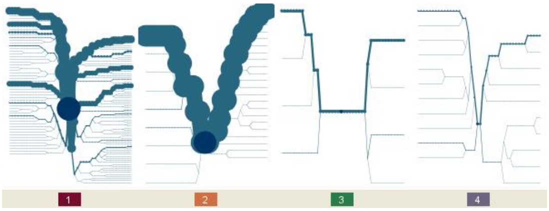

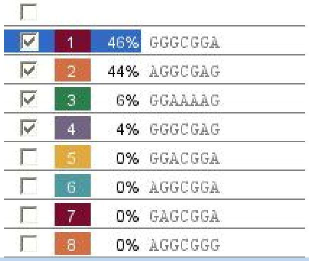

	chr20:38920878-39071965	*PRLR*

I. chr20:38960231-39046015

I

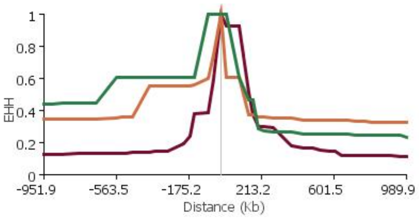

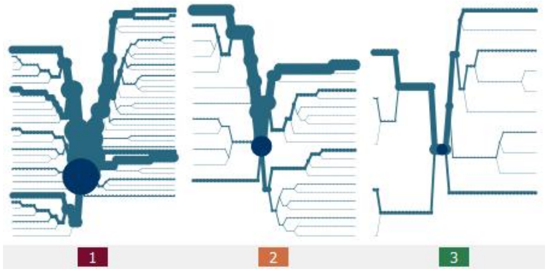

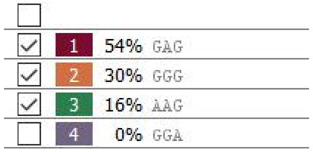

II. chr20:38920878-39071965

II

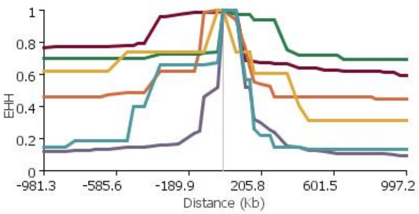

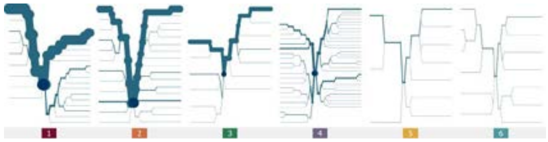

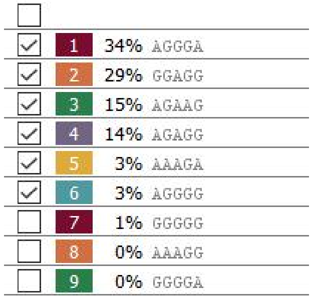

III. chr20:38920878-39071965

III

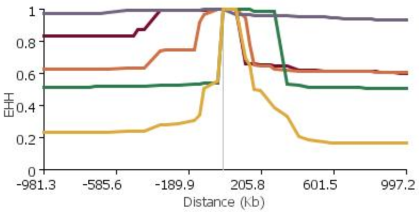

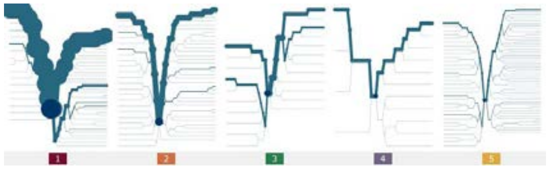

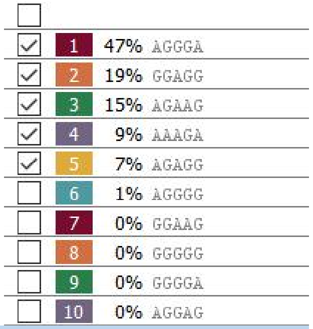

	chr20:39242226-39350230	*DNAJC21,BRIX1,RAD1, TTC23L*
I	Not defined	

III

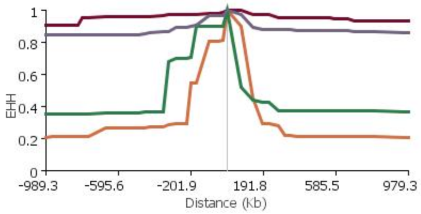

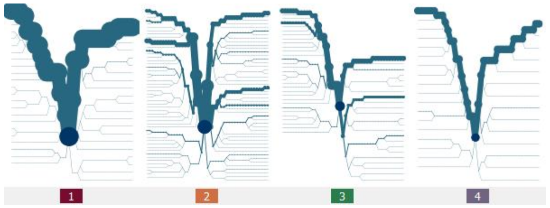

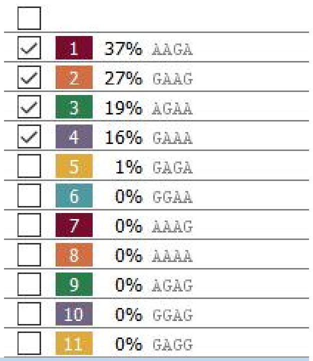

chr20:39658427-39727100

I Not defined

III

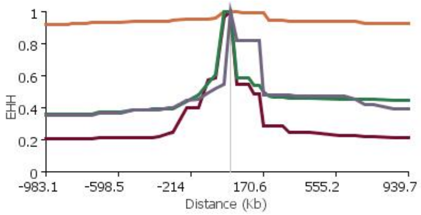

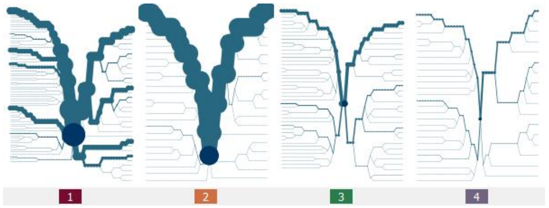

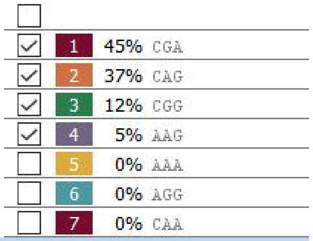

chr20:40004925-40145270 *LOC101902009*, *ADAMTS12*

I Not defined

III

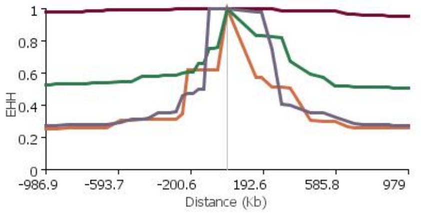

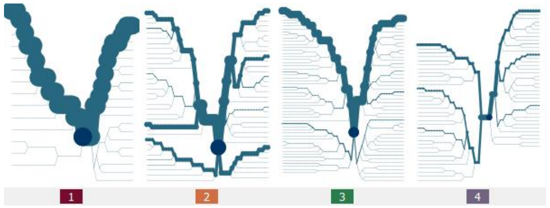

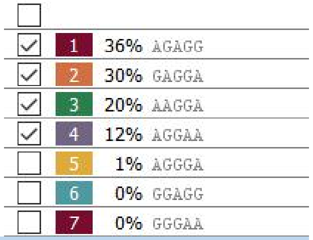

chr20:41742228-41936114 *PDZD2*

I. chr20:41777888-41936114

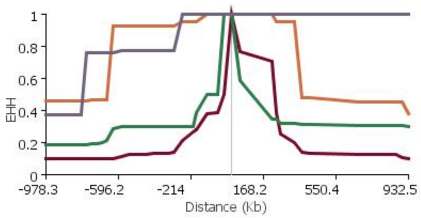

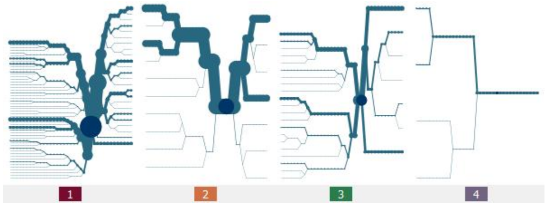

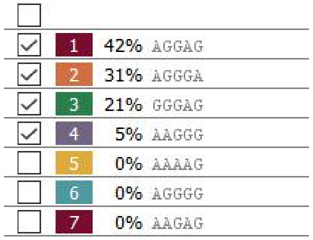

III. chr20:41742228-41936114

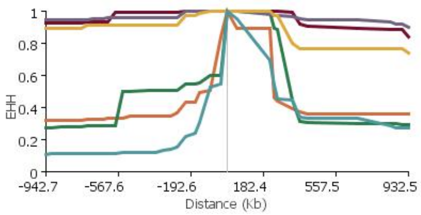

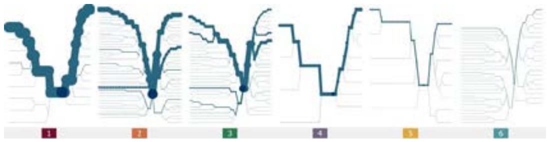

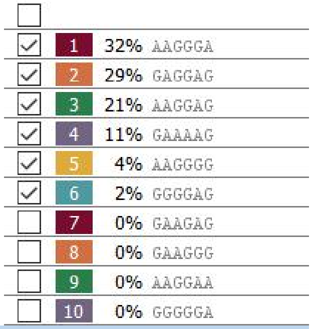

	chr20:42779370-42868638
I	Not defined

III

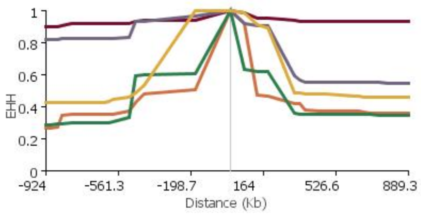

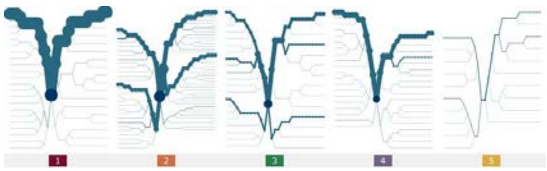

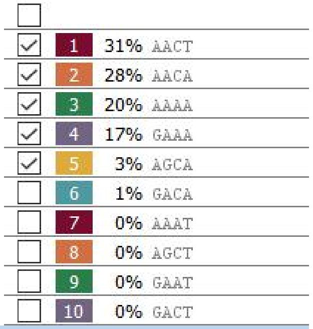

	chr20:44694564-44950378	Deletion: 44938188-44942384
I	Not defined	

III

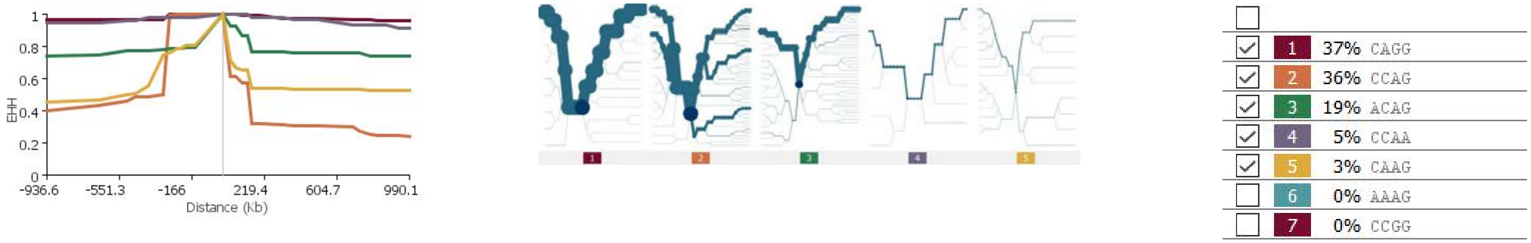

46209952-46296840

46179978-46296840

I

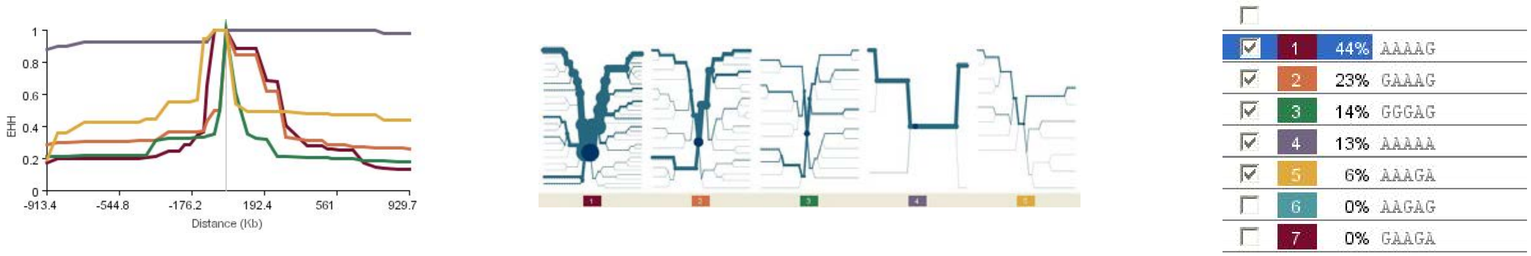

46209952-46296840

III

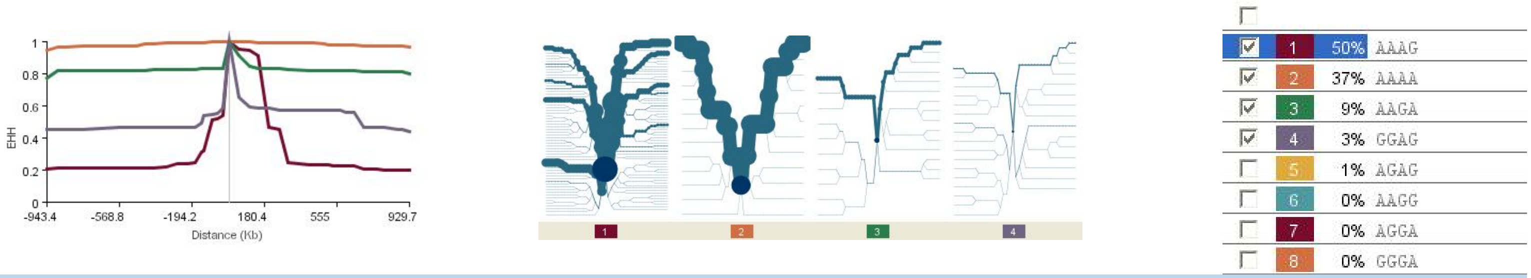

46555323-46900120 *CDH9*

46555323-46787179

I

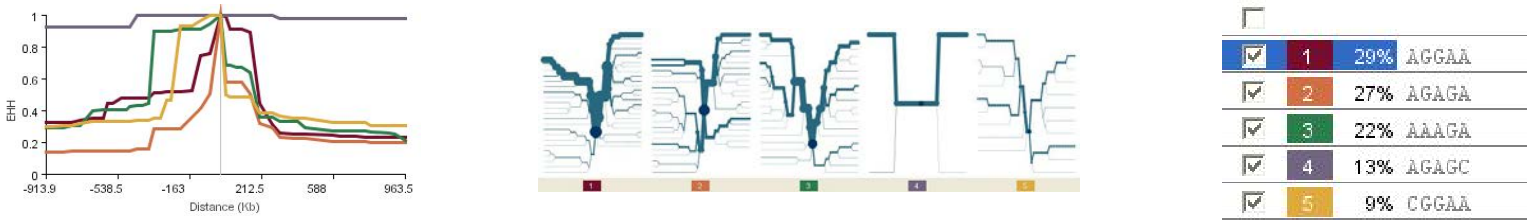

46709345-46900120

III

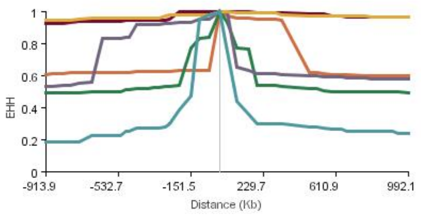

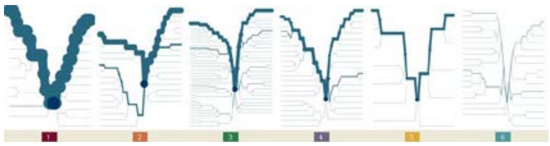

	#	%	Haplotype
☑	1	37%	AGAGCATAA
☑	2	22%	AAAGAATAA
☑	3	15%	AGAGAGTAA
☑	4	12%	AGGAAGTAA
☑	5	9%	AGGAAGACG
☑	6	2%	CGGAAGTAA
☐	7	1%	AGAGAATAA
☐	8	0%	AGGAAGACA
☐	9	0%	AAAGAGTAA
☐	10	0%	AAGAAGTAA
☐	11	0%	AAAGCGTAA
☐	12	0%	AGAGCGTAA
☐	13	0%	AGAGAGACG
☐	14	0%	AGAGAGTAG
☐	15	0%	AGAGAGTCA
☐	16	0%	AGAGCGACG
☐	17	0%	AGGAAATAA
☐	18	0%	AGGGAGTAA
☐	19	0%	CGGAAGTCA
☐	20	0%	AAAGAGACG
☐	21	0%	AGGAAGAAA
☐	22	0%	AGGAAGTCA
☐	23	0%	AGGAAGTCG
☐	24	0%	AGGACGTAA
☐	25	0%	CGAGAGTAA
☐	26	0%	CGGAAATAA

chr20:48318267-48510710 Tandem duplication: 48097291-48389570

I. chr20:48346601-48510710

I

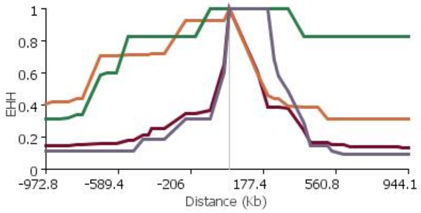

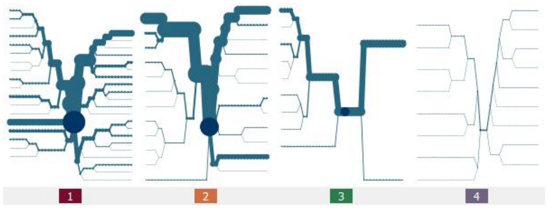

	#	%	Haplotype
☑	1	44%	GAA
☑	2	36%	GAG
☑	3	17%	AGG
☑	4	3%	GGG

III. chr20:48318267-48510710

III

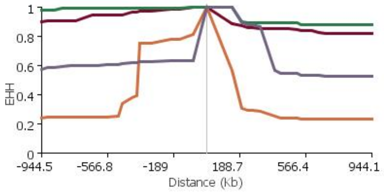

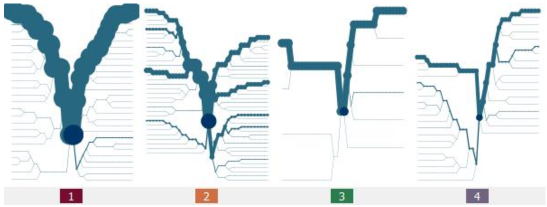

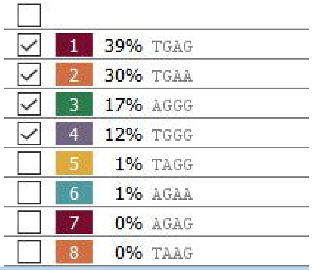

chr20:49111029-49339693

I Not defined

III

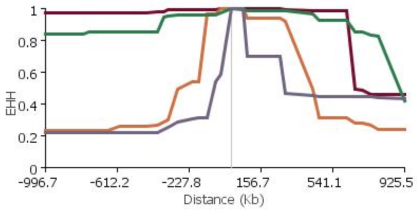

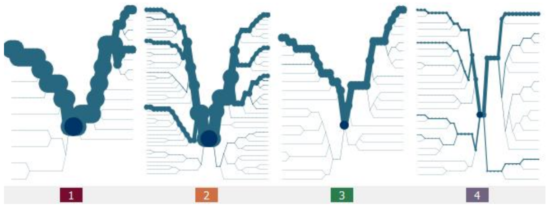

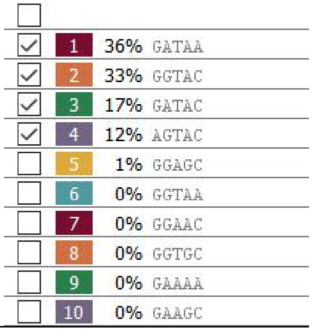

Fig. S4. Selection signature of the 21-49 Mb region of Chr20 by the analysis of extended haplotype homozygosity (EHH). Most selection signatures had high frequency haplotypes (≥0.30) and high EHH values (≥0.60) for long distances (≥1.8 Mb) in the Holsteins subjected to 40 years of selection (Group III), and these long haplotypes virtually covered the entire 21-49 Mb region.
