# Supplementary material for: Genome changes due to artificial selection in U.S. Holstein cattle
Source: BMC Genomics. 2019 Feb 11;20:128. doi: 10.1186/s12864-019-5459-x (PMC6371544; doi:10.1186/s12864-019-5459-x)
Supplement: Supplementary file 5 — Figure S5. Extended haplotype homozygosity (EHH) evidence of selection in three Holstein groups for all 30 bovine chromosomes. All autosomes had long-range EHH values indicating selection, but the center region of Chr20 had the highest concentration of long haplotypes with high EHH values. ‘I’ is Group I unselected since 1964. ‘II’ is Group II subjected to 20 years of selection, and ‘III’ is Group III subjected to 40 years of selection since 1964. The EHH distances were haplotype distances with minimal EHH value of 0.6. (PDF 611 kb) [file 12864_2019_5459_MOESM5_ESM.pdf]

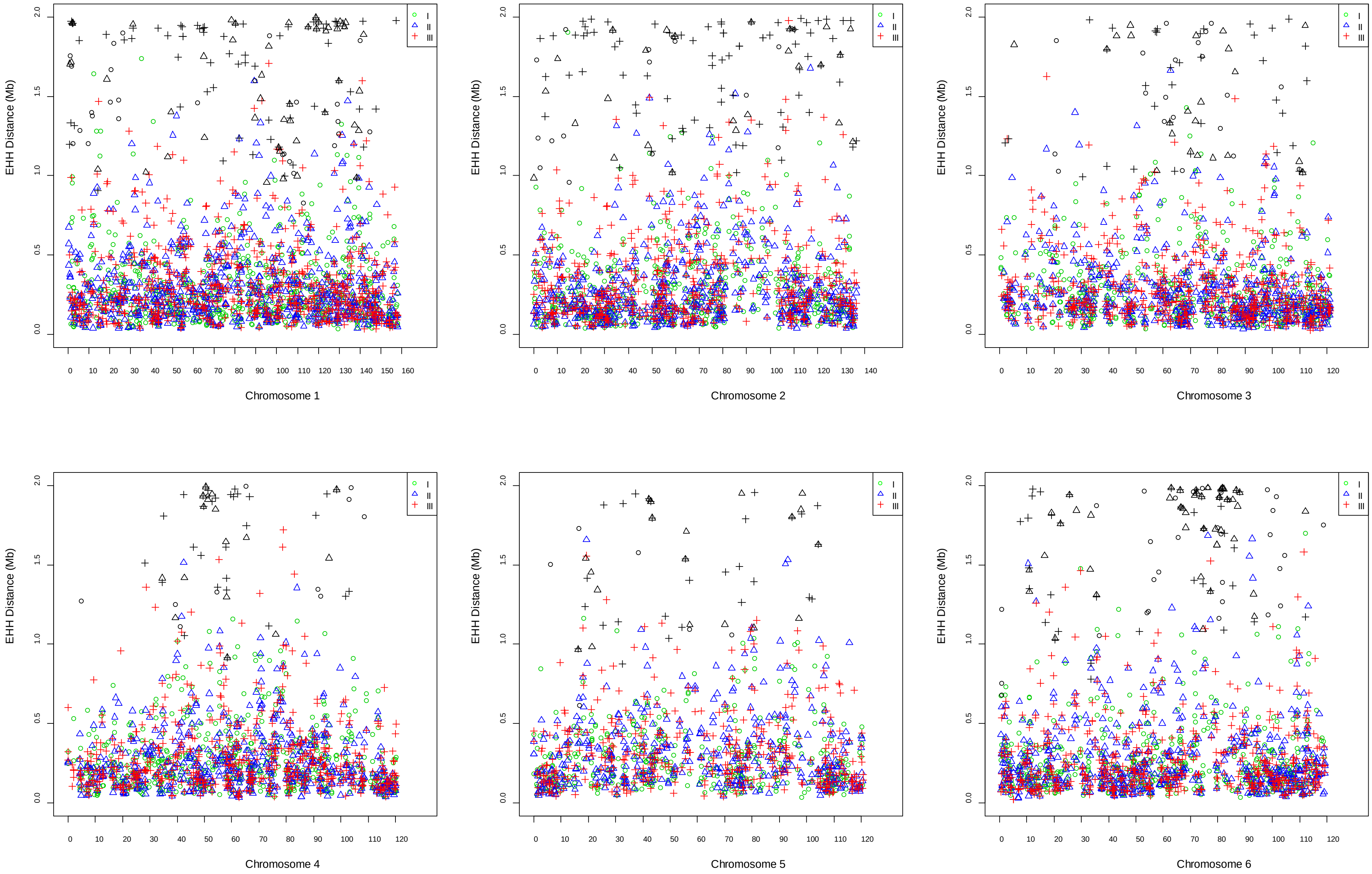
EHH Distance (Mb)
Chromosome 1
Chromosome 2
Chromosome 3
Chromosome 4
Chromosome 5
Chromosome 6
I
II
III

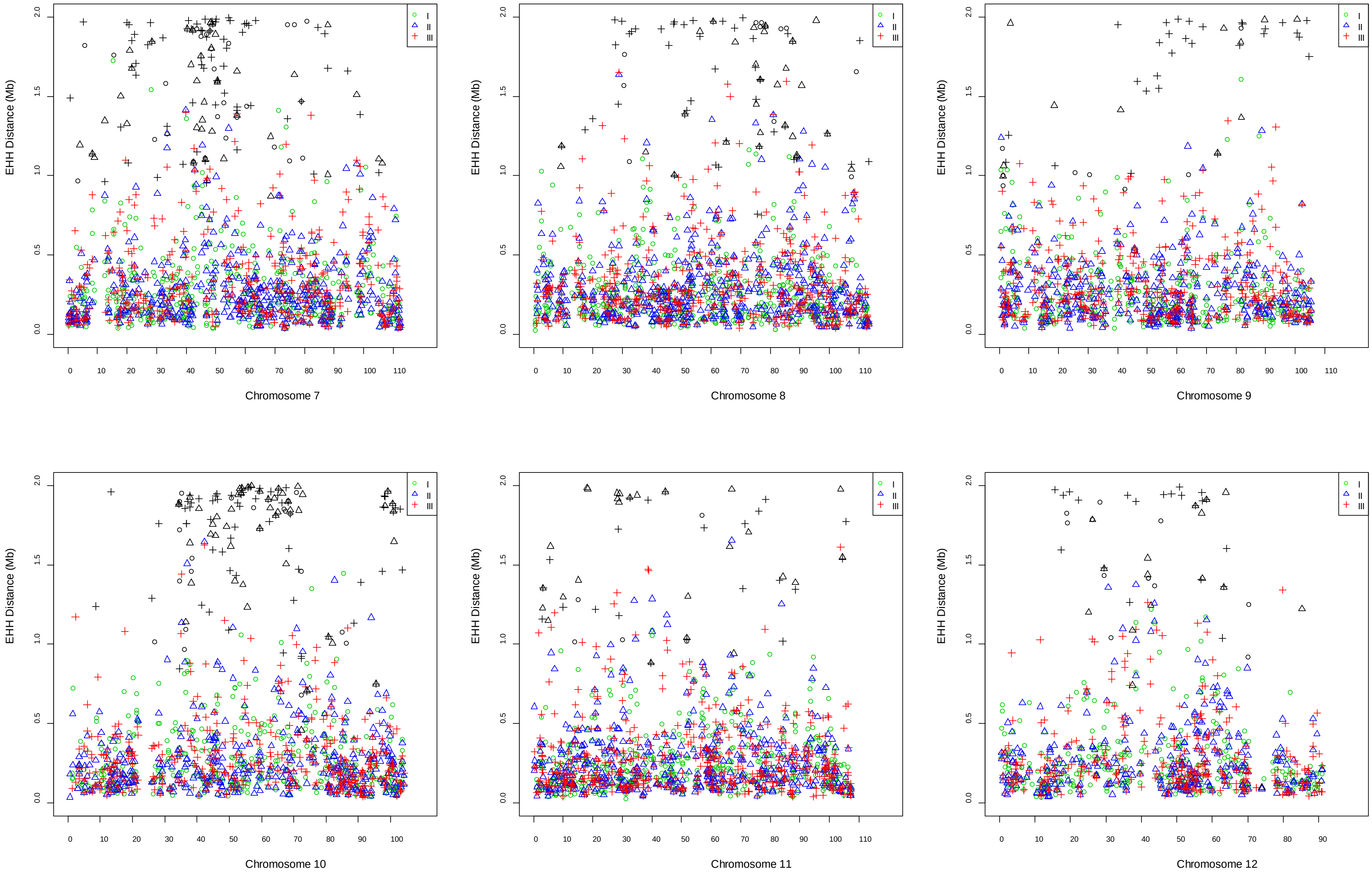
EHH Distance (Mb)
I
II
III
Chromosome 7
Chromosome 8
Chromosome 9
Chromosome 10
Chromosome 11
Chromosome 12

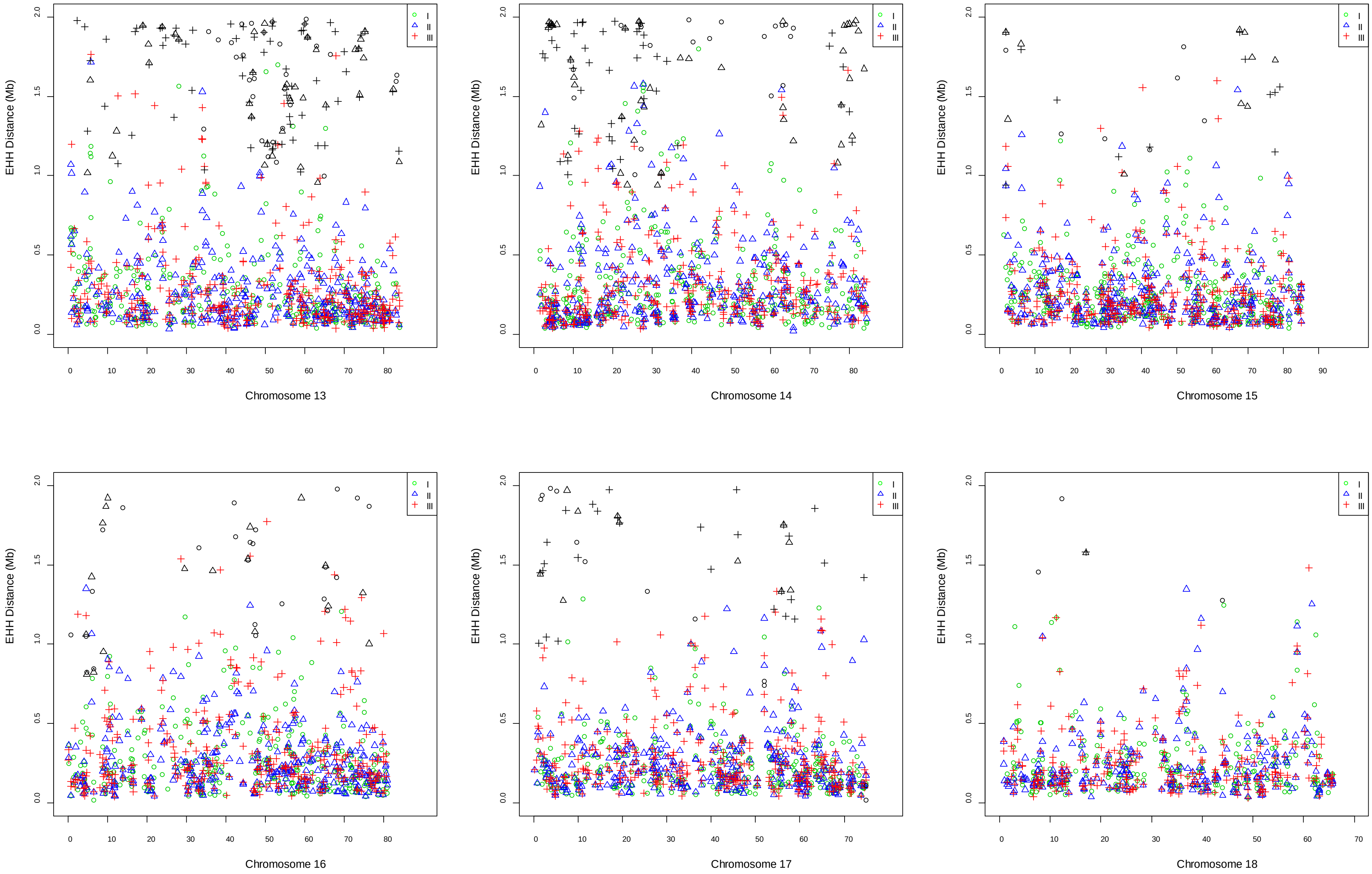
EHH Distance (Mb)
0.0
0.5
1.0
1.5
2.0
I
II
III
0
10
20
30
40
50
60
70
80
90
Chromosome 13
Chromosome 14
Chromosome 15
Chromosome 16
Chromosome 17
Chromosome 18

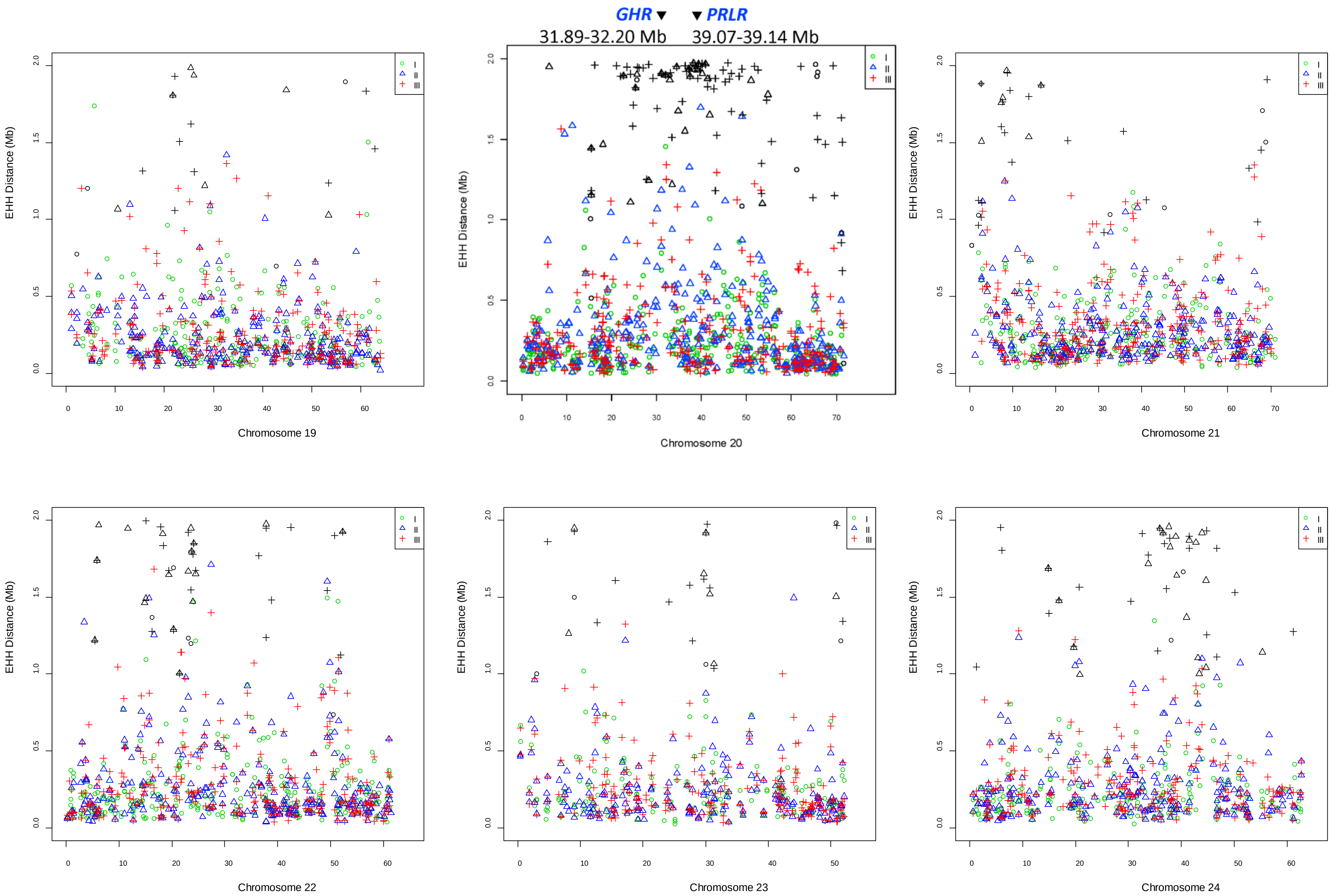
GHR
PRLR
31.89-32.20 Mb
39.07-39.14 Mb
EHH Distance (Mb)
Chromosome 19
Chromosome 20
Chromosome 21
Chromosome 22
Chromosome 23
Chromosome 24
I
II
III

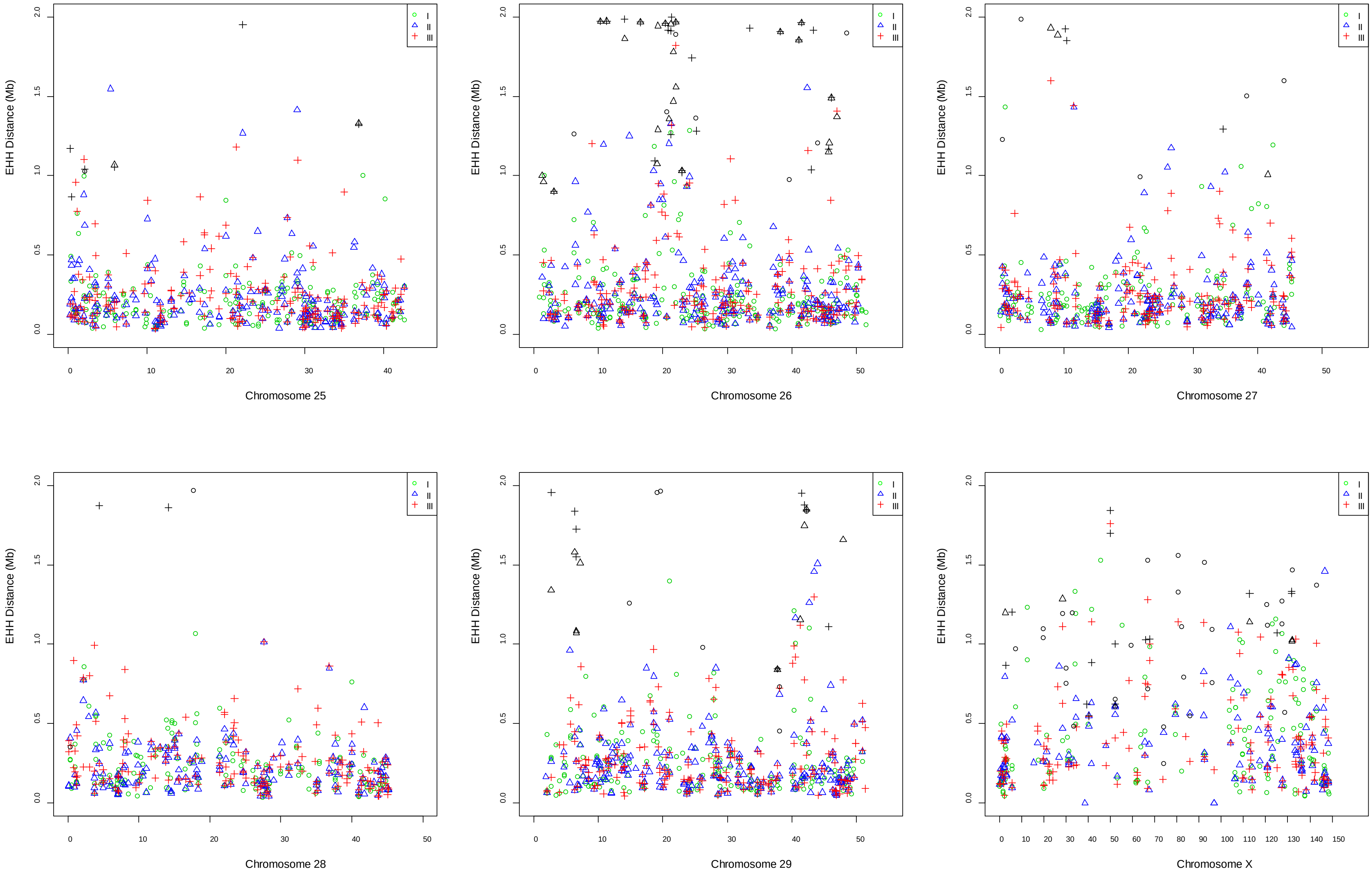

Fig. S5. Extended haplotype homozygosity (EHH) evidence of selection in three Holstein groups for all 30 bovine chromosomes. All autosomes had long-range EHH values indicating selection, but the center region of Chr20 had the highest concentration of long haplotypes with high EHH values. 'I' is Group I unselected since 1964. 'II' is Group II subjected to 20 years of selection, and 'III' is Group III subjected to 40 years of selection since 1964. The EHH distances were haplotype distances with minimal EHH value of 0.6.
