# Supplementary material for: Genome changes due to artificial selection in U.S. Holstein cattle
Source: BMC Genomics. 2019 Feb 11;20:128. doi: 10.1186/s12864-019-5459-x (PMC6371544; doi:10.1186/s12864-019-5459-x)
Supplement: Supplementary file 6 — Figure S6. Long-range differences of allele frequencies and heterozygosity between unselected and selected Holsteins since 1964. Left column: 40 years of selection between Groups I and III. Middle column: the first 20 years of selection between Groups I and II. Right column: the second 20 years of selection between Groups II and III. Chr30 is the X chromosome. (PDF 19496 kb) [file 12864_2019_5459_MOESM6_ESM.pdf]

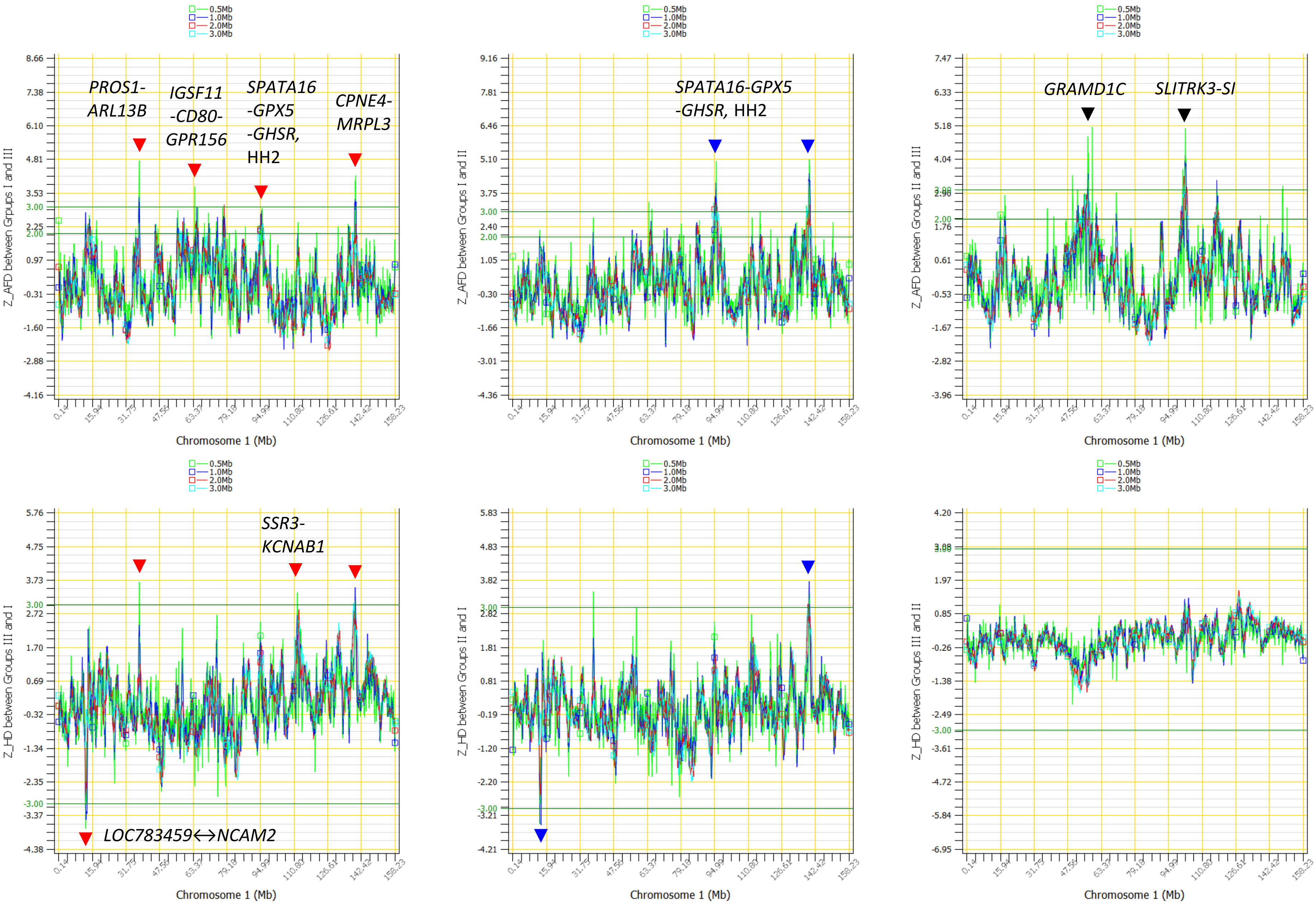
0.5Mb
1.0Mb
2.0Mb
3.0Mb
Z_AFD between Groups I and III
PROS1-ARL13B
IGSF11-CD80-GPR156
SPATA16-GPX5-GHSR, HH2
CPNE4-MRPL3
Chromosome 1 (Mb)
Z_AFD between Groups I and II
SPATA16-GPX5-GHSR, HH2
Chromosome 1 (Mb)
Z_AFD between Groups II and III
GRAMD1C
SLITRK3-SI
Chromosome 1 (Mb)
Z_HD between Groups III and I
SSR3-KCNAB1
LOC783459↔NCAM2
Chromosome 1 (Mb)
Z_HD between Groups II and I
Chromosome 1 (Mb)
Z_HD between Groups III and II
Chromosome 1 (Mb)

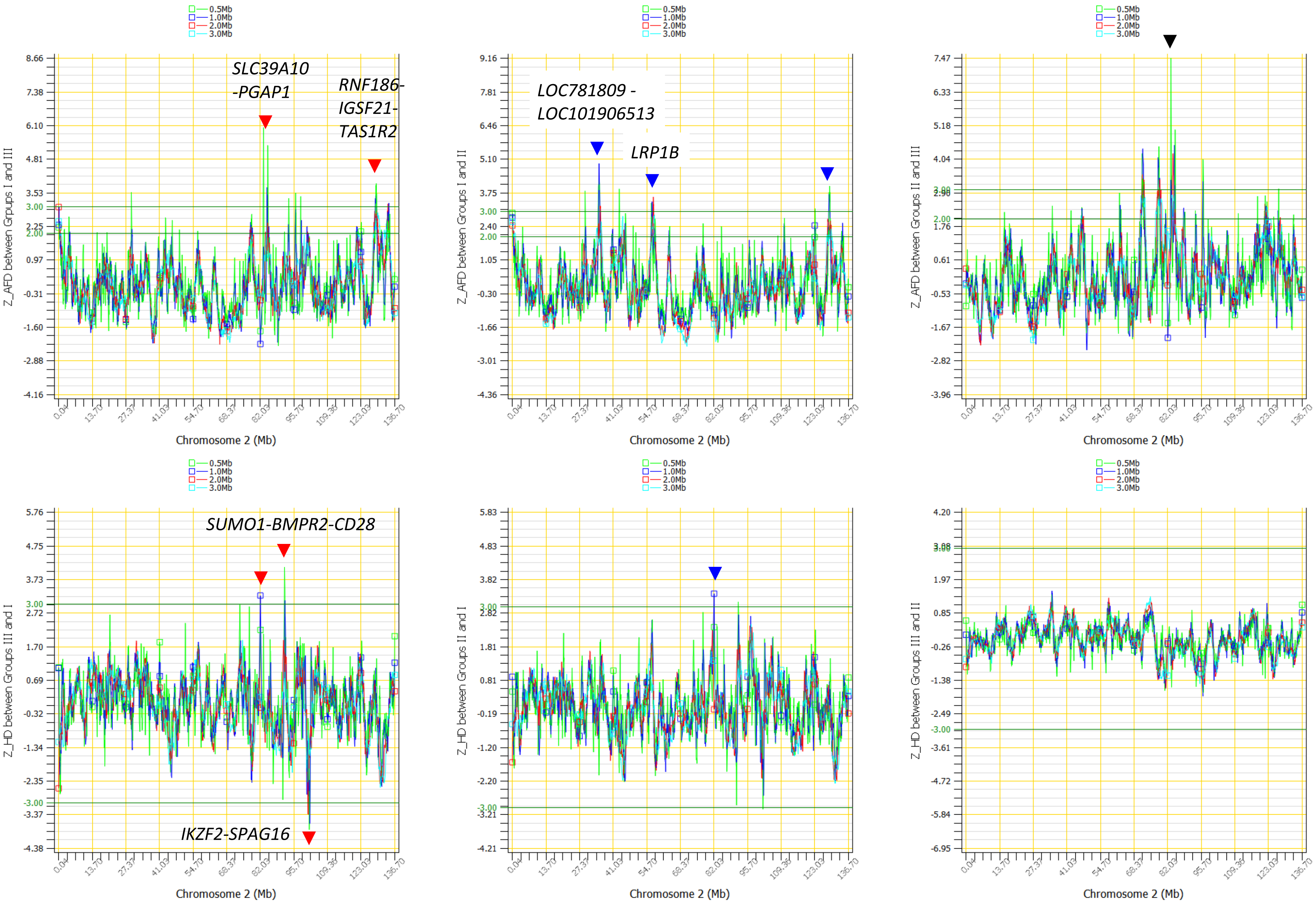

0.5Mb
1.0Mb
2.0Mb
3.0Mb
SLC39A10
-PGAP1
RNF186-
IGSF21-
TAS1R2
Z_AFD between Grpups I and III
Chromosome 2 (Mb)
LOC781809 -
LOC101906513
LRP1B
Z_AFD between Groups I and II
Z_AFD between Groups II and III
SUMO1-BMPR2-CD28
IKZF2-SPAG16
Z_HD between Groups III and I
Z_HD between Groups II and I
Z_HD between Groups III and II

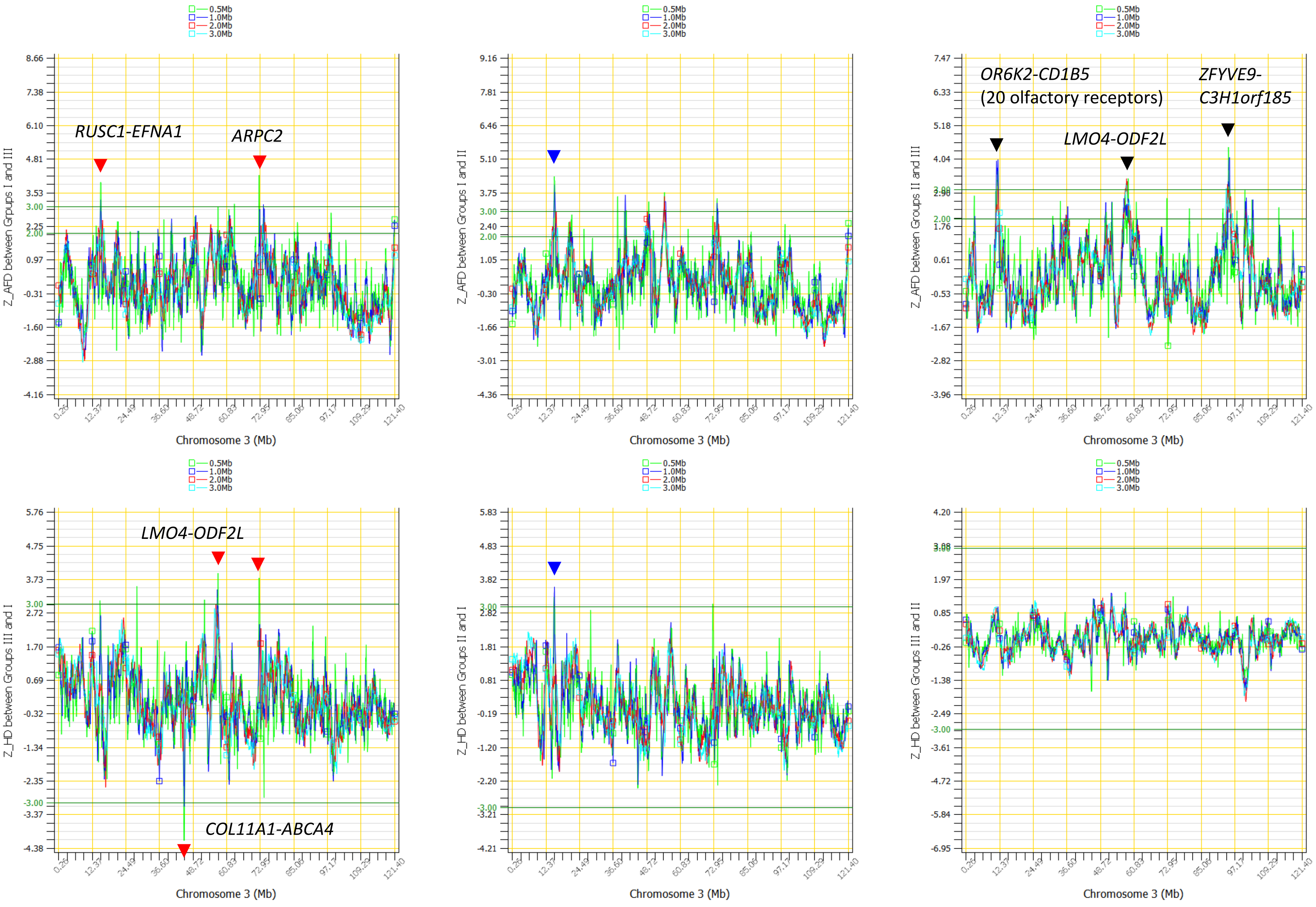

0.5Mb
1.0Mb
2.0Mb
3.0Mb
Z_AFD between Groups I and III
RUSC1-EFNA1
ARPC2
Z_AFD between Groups I and II
Z_AFD between Groups II and III
OR6K2-CD1B5
(20 olfactory receptors)
ZFYVE9-
C3H1orf185
LMO4-ODF2L
Z_HD between Groups III and I
LMO4-ODF2L
COL11A1-ABCA4
Z_HD between Groups II and I
Z_HD between Groups III and II
Chromosome 3 (Mb)

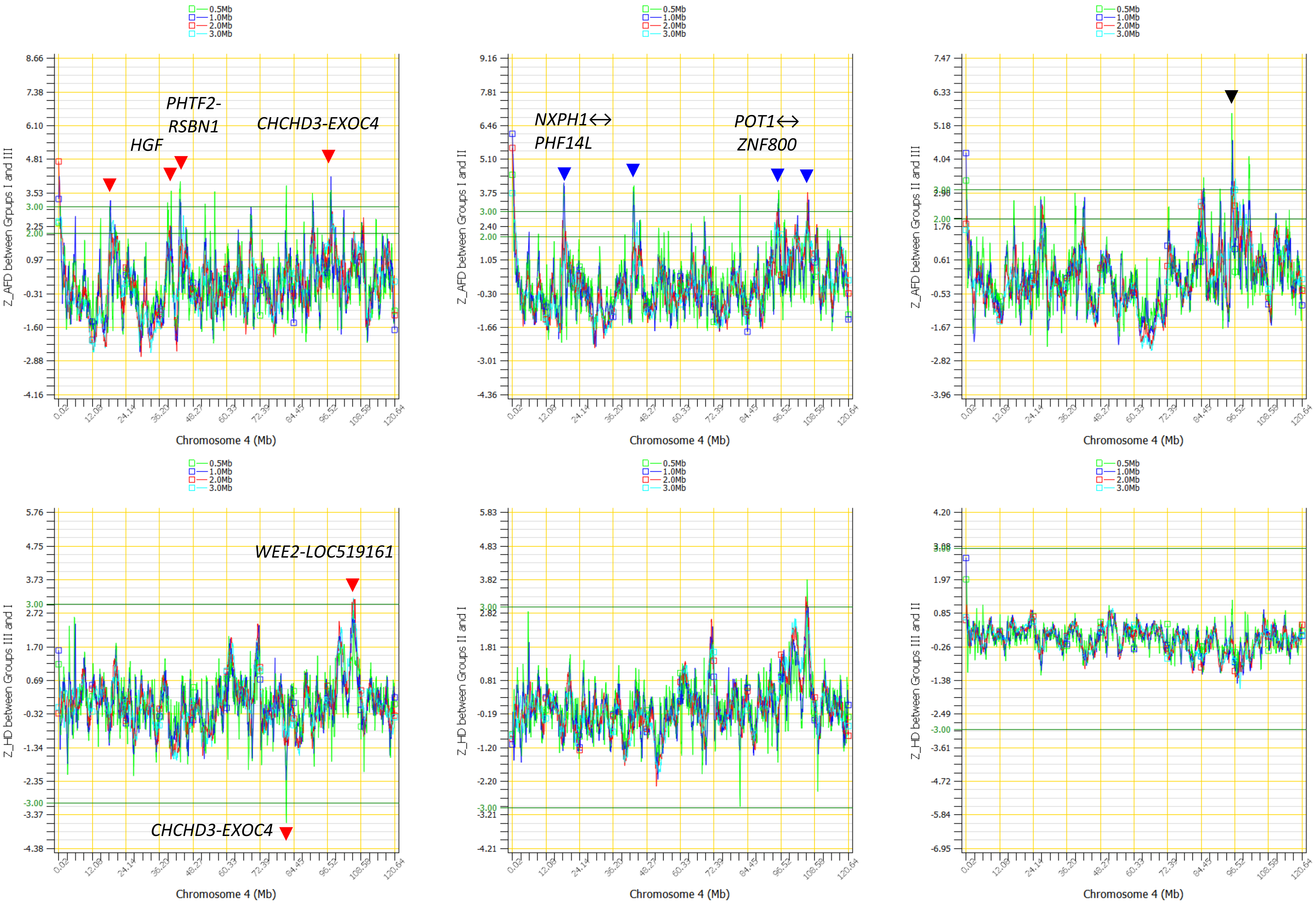
0.5Mb
1.0Mb
2.0Mb
3.0Mb
Z_AFD between Groups I and III
HGF
PHTF2-
RSBN1
CHCHD3-EXOC4
Chromosome 4 (Mb)
Z_AFD between Groups I and II
NXPH1↔
PHF14L
POT1↔
ZNF800
Chromosome 4 (Mb)
Z_AFD between Groups II and III
Chromosome 4 (Mb)
Z_HD between Groups III and I
WEE2-LOC519161
CHCHD3-EXOC4
Chromosome 4 (Mb)
Z_HD between Groups II and I
Chromosome 4 (Mb)
Z_HD between Groups III and II
Chromosome 4 (Mb)

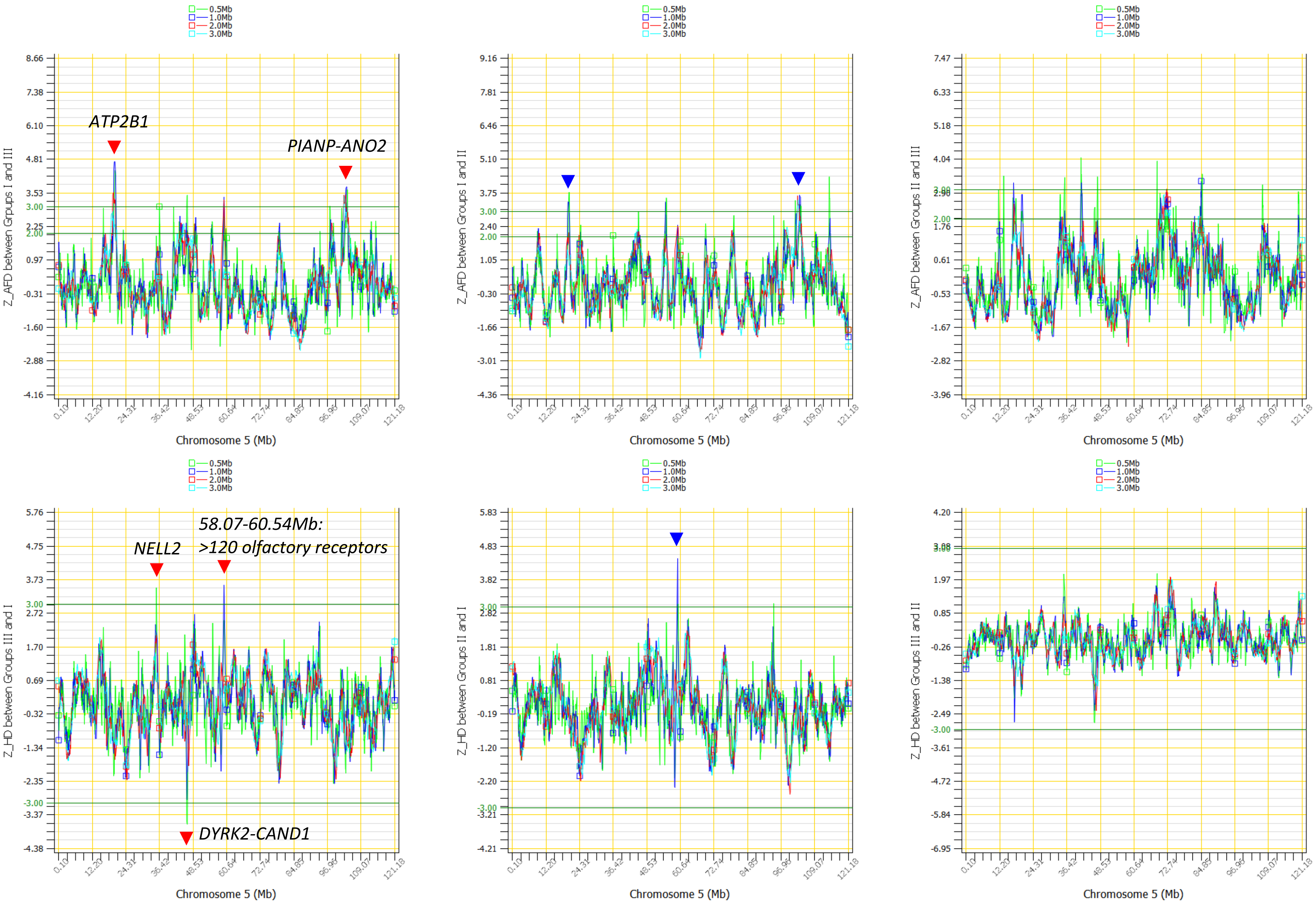

0.5Mb
1.0Mb
2.0Mb
3.0Mb
ATP2B1
PIANP-ANO2
Z_AFD between Grpups I and III
Z_AFD between Groups I and II
Z_AFD between Groups II and III
NELL2
58.07-60.54Mb:
>120 olfactory receptors
DYRK2-CAND1
Z_HD between Groups III and I
Z_HD between Groups II and I
Z_HD between Groups III and II
Chromosome 5 (Mb)

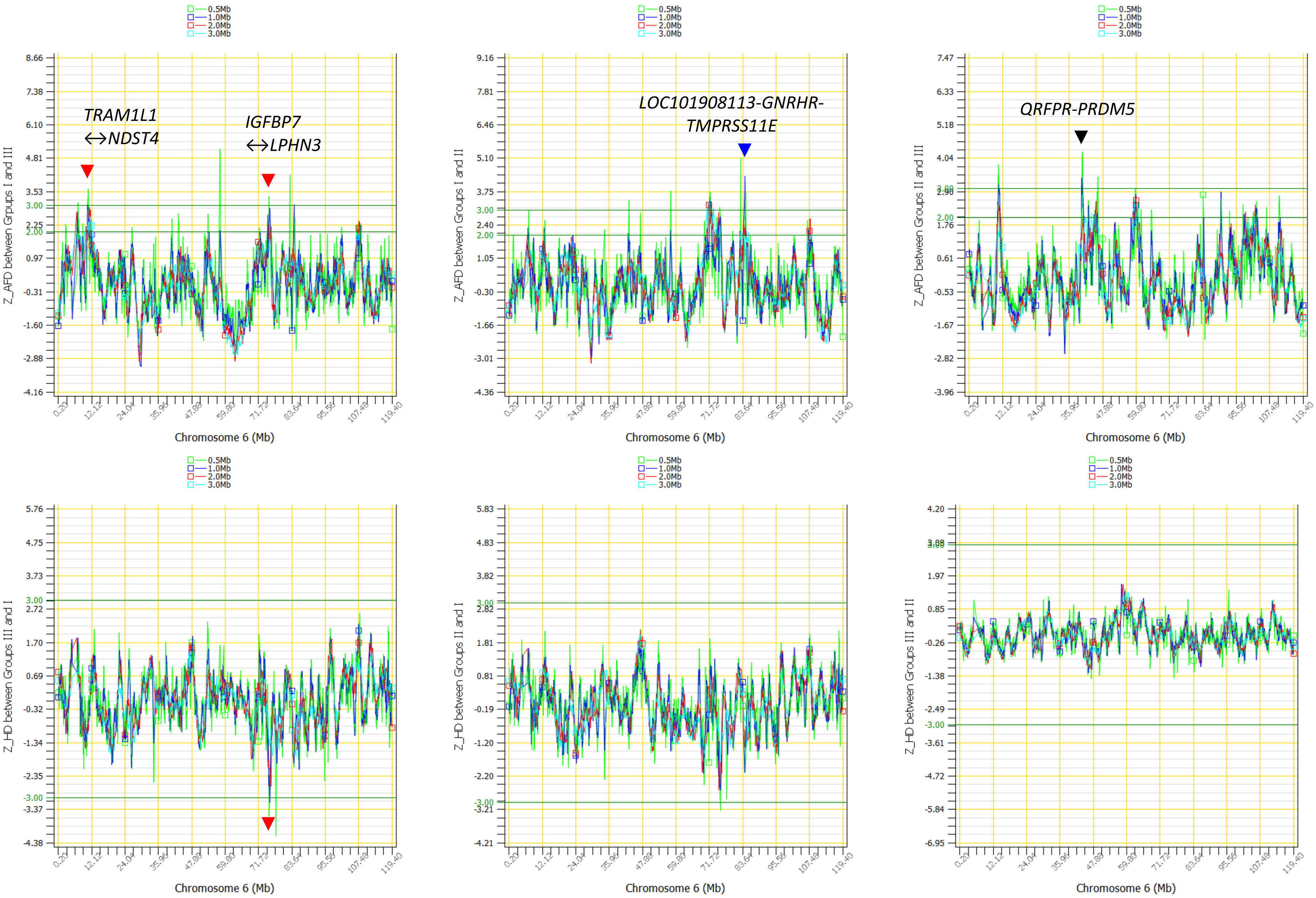
0.5Mb
1.0Mb
2.0Mb
3.0Mb
TRAM1L1
↔NDST4
IGFBP7
↔LPHN3
LOC101908113-GNRHR-
TMPRSS11E
QRFPR-PRDM5
Z_AFD between Groups I and III
Z_AFD between Groups I and II
Z_AFD between Groups II and III
Z_HD between Groups III and I
Z_HD between Groups II and I
Z_HD between Groups III and II
Chromosome 6 (Mb)
Chromosome 6 (Mb)
Chromosome 6 (Mb)
Chromosome 6 (Mb)
Chromosome 6 (Mb)
Chromosome 6 (Mb)

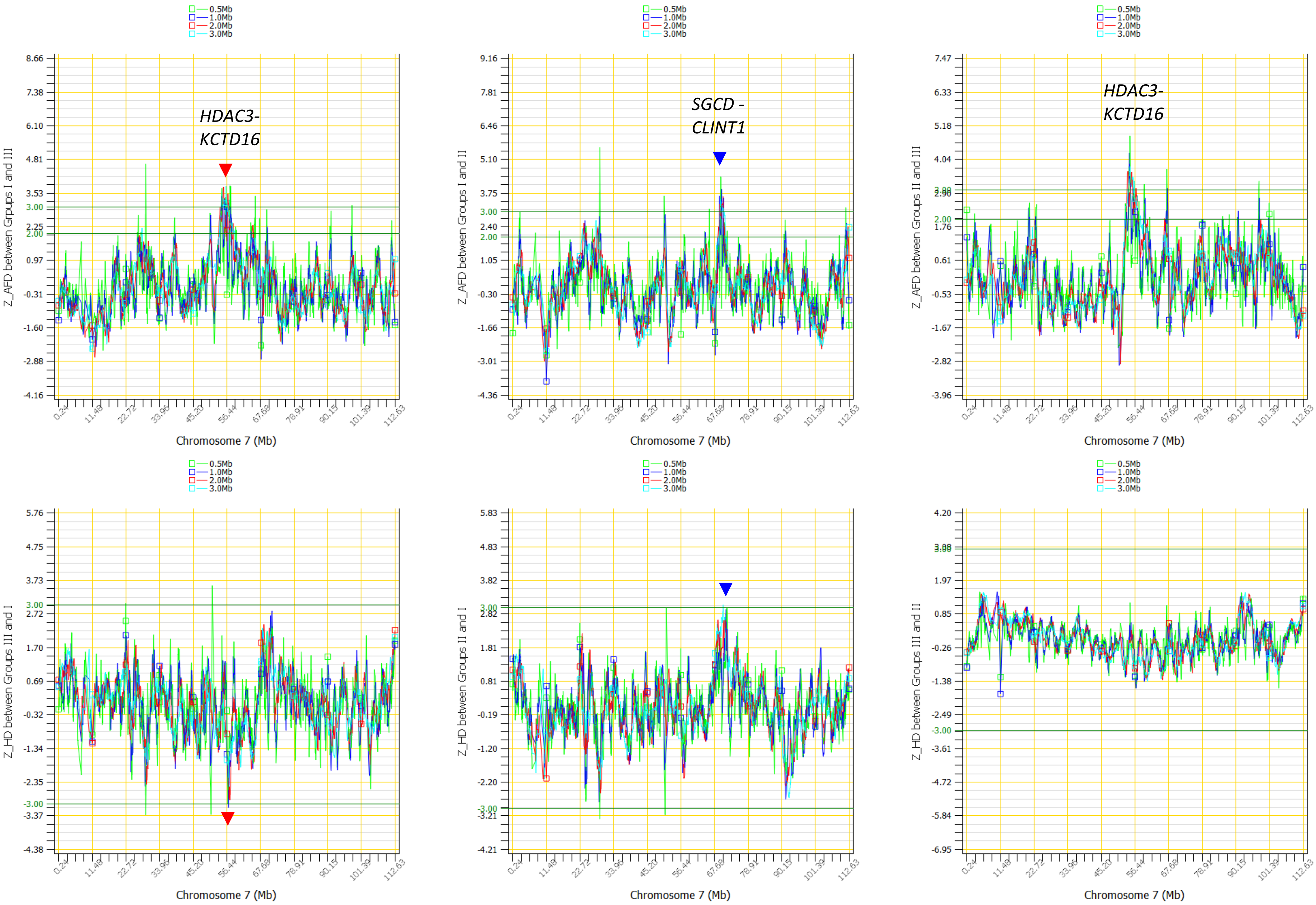
0.5Mb
1.0Mb
2.0Mb
3.0Mb
HDAC3-KCTD16
SGCD - CLINT1
HDAC3-KCTD16
Z_AFD between Grpups I and III
Z_AFD between Groups I and II
Z_AFD between Groups II and III
Z_HD between Groups III and I
Z_HD between Groups II and I
Z_HD between Groups III and II
Chromosome 7 (Mb)
0.24
11.48
22.72
33.96
45.20
56.44
67.68
78.91
90.15
101.39
112.63

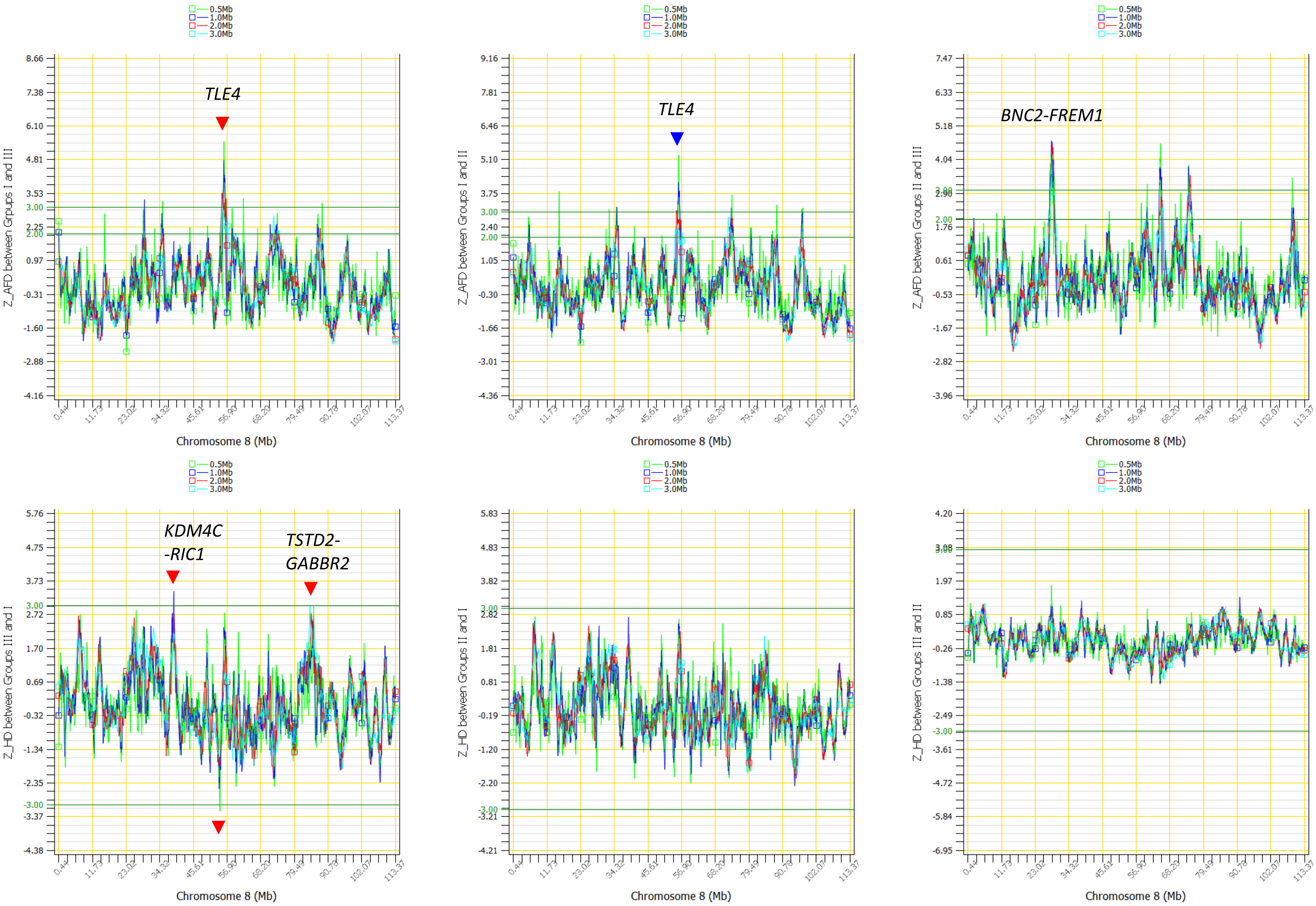
0.5Mb
1.0Mb
2.0Mb
3.0Mb
Z_AFD between Grpups I and III
TLE4
Chromosome 8 (Mb)
Z_AFD between Groups I and II
TLE4
Chromosome 8 (Mb)
Z_AFD between Groups II and III
BNC2-FREM1
Chromosome 8 (Mb)
Z_HD between Groups III and I
KDM4C
-RIC1
TSTD2-
GABBR2
Chromosome 8 (Mb)
Z_HD between Groups II and I
Chromosome 8 (Mb)
Z_HD between Groups III and II
Chromosome 8 (Mb)

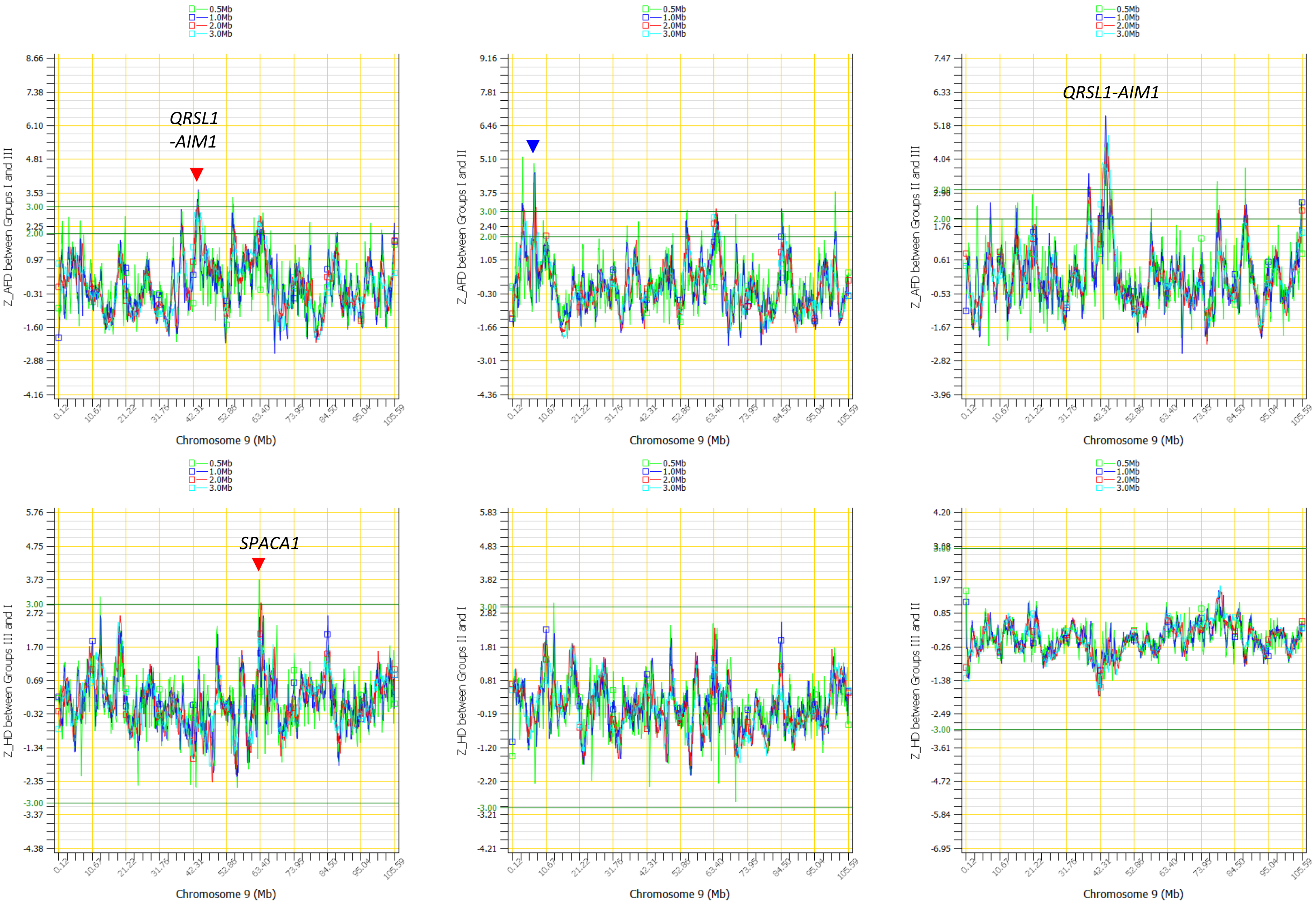
0.5Mb
1.0Mb
2.0Mb
3.0Mb
QRSL1
-AIM1
Z_AFD between Groups I and III
Chromosome 9 (Mb)
Z_AFD between Groups I and II
Chromosome 9 (Mb)
QRSL1-AIM1
Z_AFD between Groups II and III
Chromosome 9 (Mb)
SPACA1
Z_HD between Groups III and I
Chromosome 9 (Mb)
Z_HD between Groups II and I
Chromosome 9 (Mb)
Z_HD between Groups III and II
Chromosome 9 (Mb)

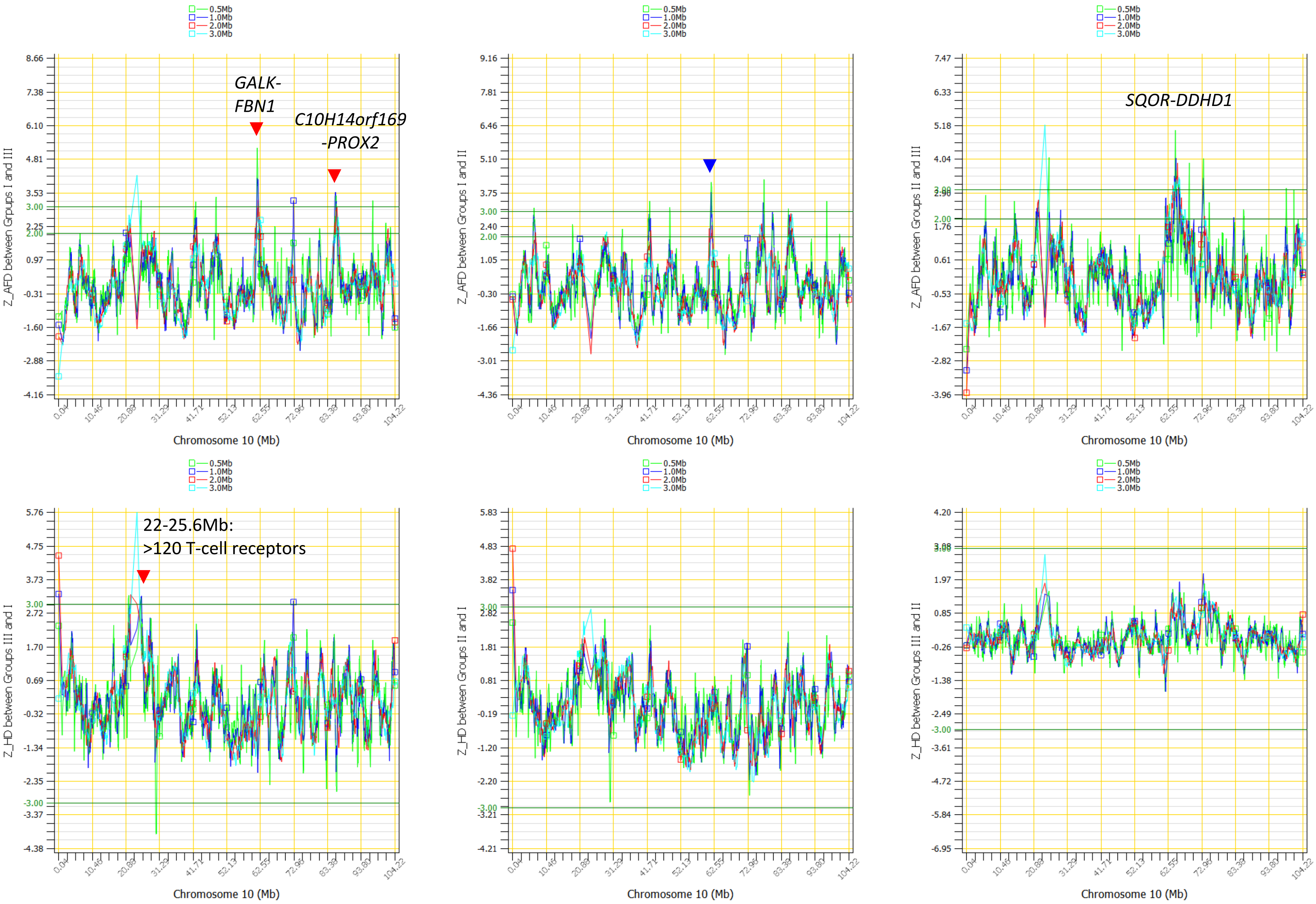
0.5Mb
1.0Mb
2.0Mb
3.0Mb
Z_AFD between Grpups I and III
GALK-FBN1
C10H14orf169 -PROX2
Z_AFD between Groups I and II
Z_AFD between Groups II and III
SQOR-DDHD1
Z_HD between Groups III and I
22-25.6Mb: >120 T-cell receptors
Z_HD between Groups II and I
Z_HD between Groups III and II
Chromosome 10 (Mb)

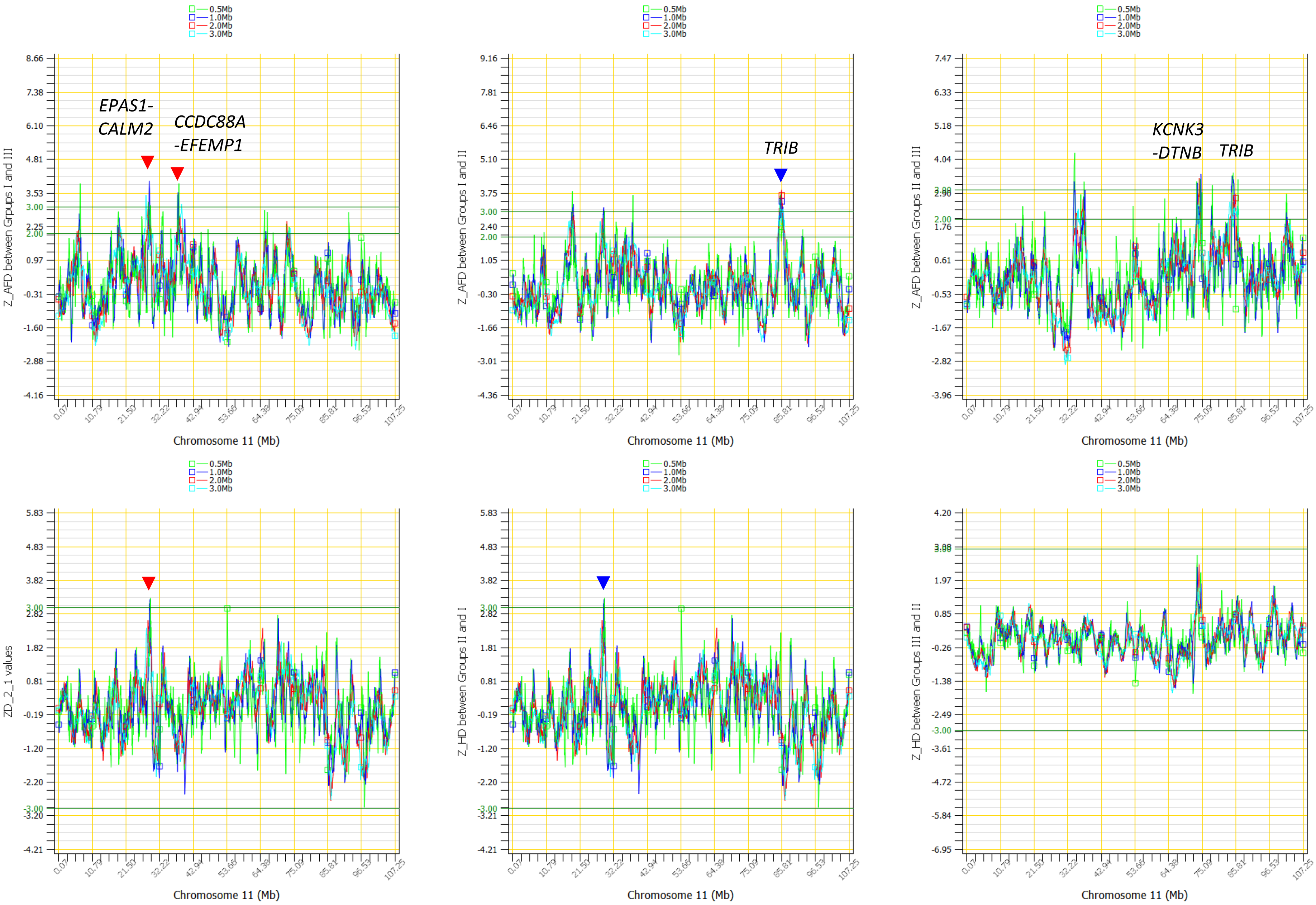
0.5Mb
1.0Mb
2.0Mb
3.0Mb
Z_AFD between Groups I and III
EPAS1-
CALM2
CCDC88A
-EFEMP1
Chromosome 11 (Mb)
Z_AFD between Groups I and II
TRIB
Z_AFD between Groups II and III
KCNK3
-DTNB
TRIB
ZD_2_1 values
Z_HD between Groups II and I
Z_HD between Groups III and II

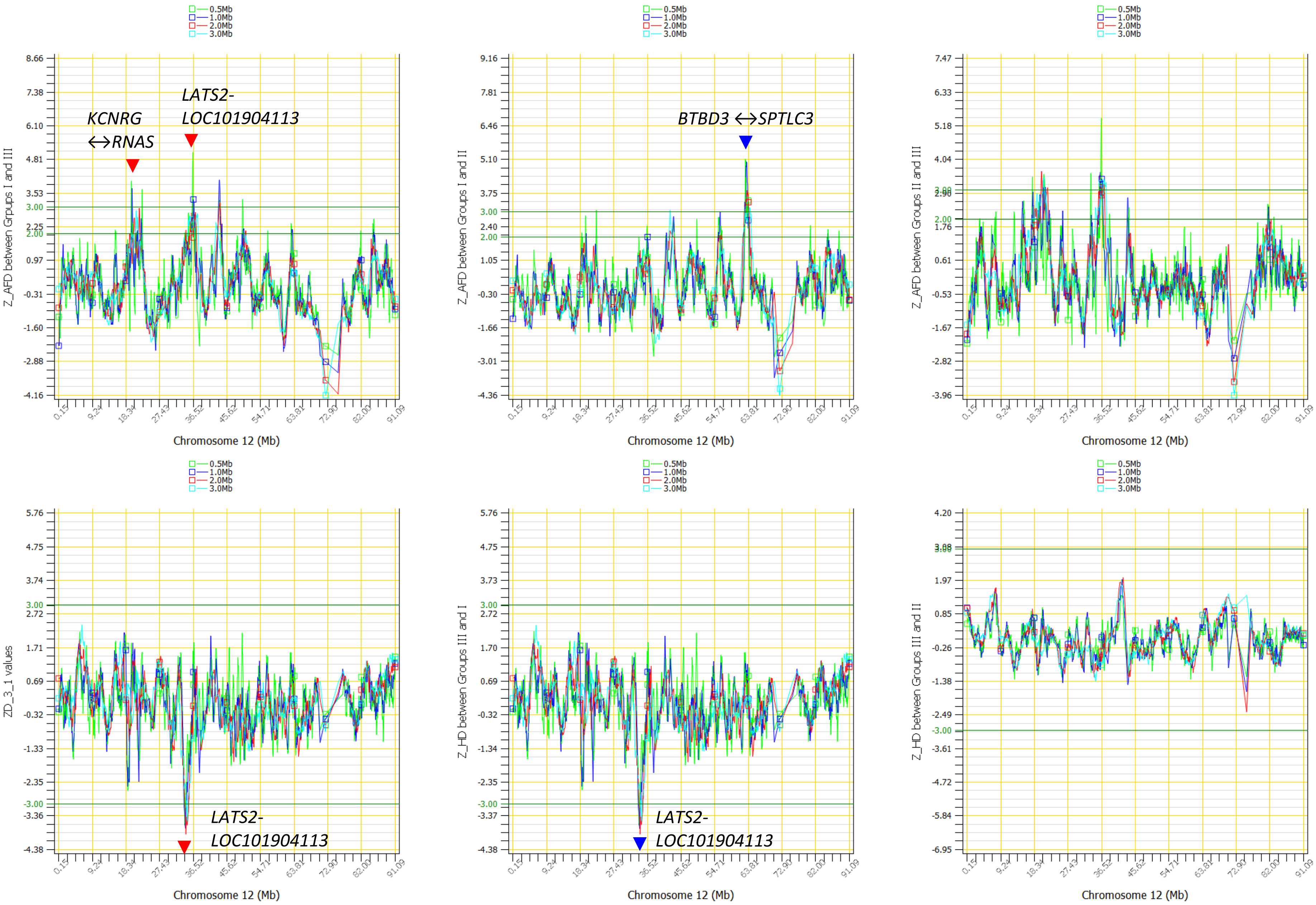

0.5Mb
1.0Mb
2.0Mb
3.0Mb
Z_AFD between Groups I and III
KCNRG
↔RNAS
LATS2-
LOC101904113
Chromosome 12 (Mb)
Z_AFD between Groups I and II
BTBD3 ↔SPTLC3
Chromosome 12 (Mb)
Z_AFD between Groups II and III
Chromosome 12 (Mb)
ZD_3_1 values
LATS2-
LOC101904113
Chromosome 12 (Mb)
Z_HD between Groups III and I
LATS2-
LOC101904113
Chromosome 12 (Mb)
Z_HD between Groups III and II
Chromosome 12 (Mb)

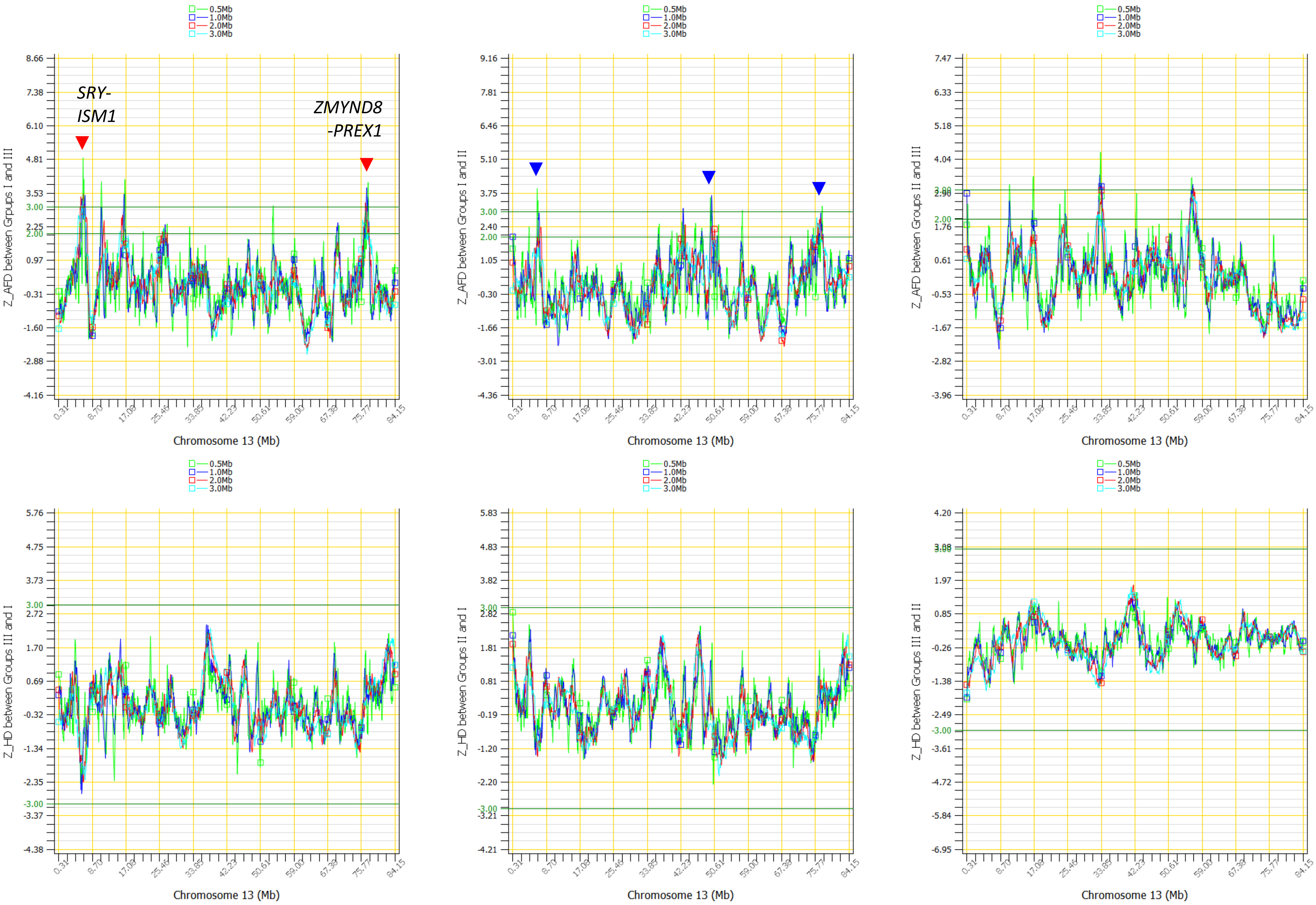

0.5Mb
1.0Mb
2.0Mb
3.0Mb
SRY-ISM1
ZMYND8 -PREX1
Z_AFD between Grpups I and III
Z_AFD between Groups I and II
Z_AFD between Groups II and III
Z_HD between Groups III and I
Z_HD between Groups II and I
Z_HD between Groups III and II
Chromosome 13 (Mb)

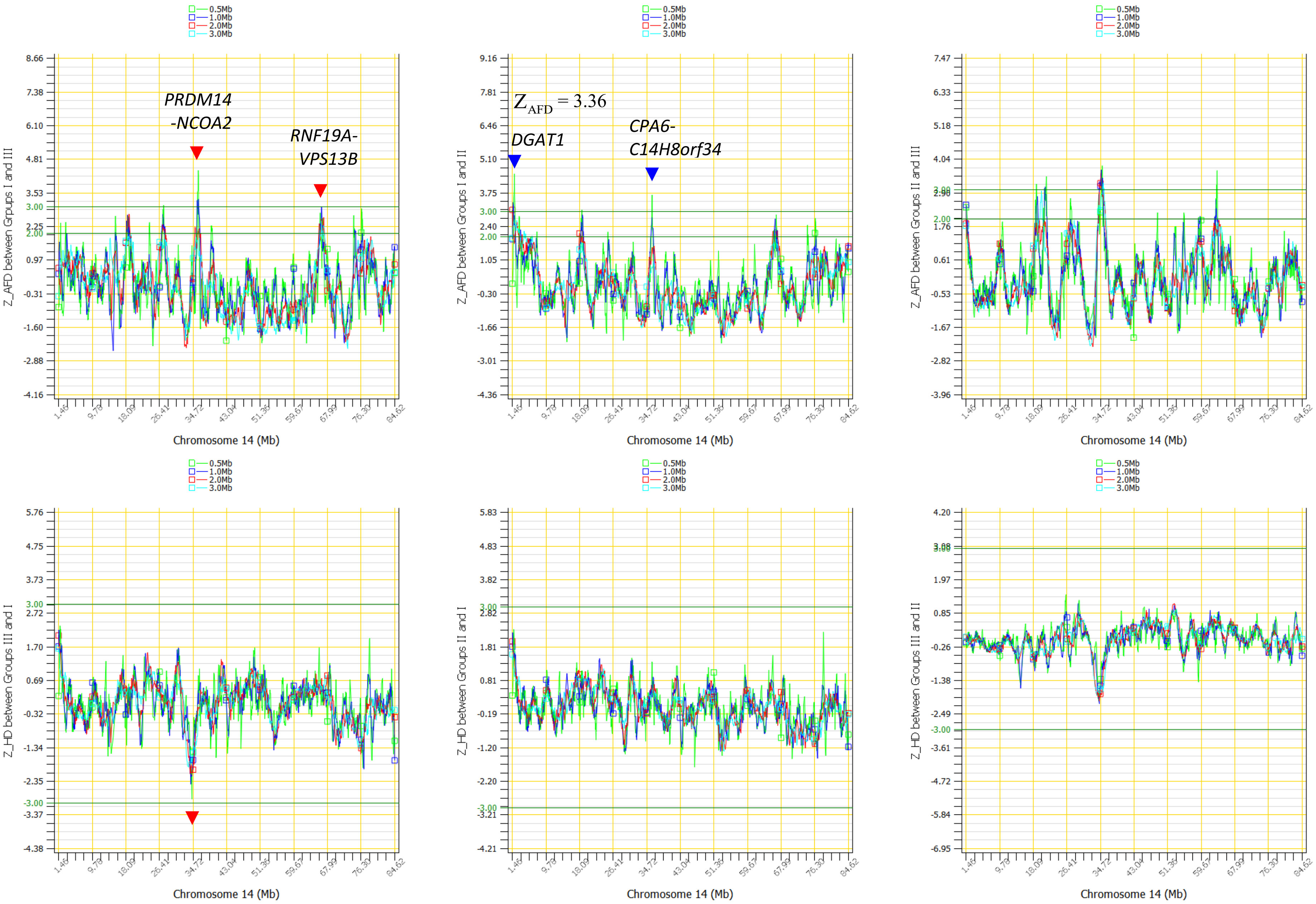
0.5Mb
1.0Mb
2.0Mb
3.0Mb
PRDM14
-NCOA2
RNF19A-
VPS13B
Z_AFD between Groups I and III
Chromosome 14 (Mb)
Z_AFD = 3.36
DGAT1
CPA6-
C14H8orf34
Z_AFD between Groups I and II
Z_AFD between Groups II and III
Z_HD between Groups III and I
Z_HD between Groups II and I
Z_HD between Groups III and II

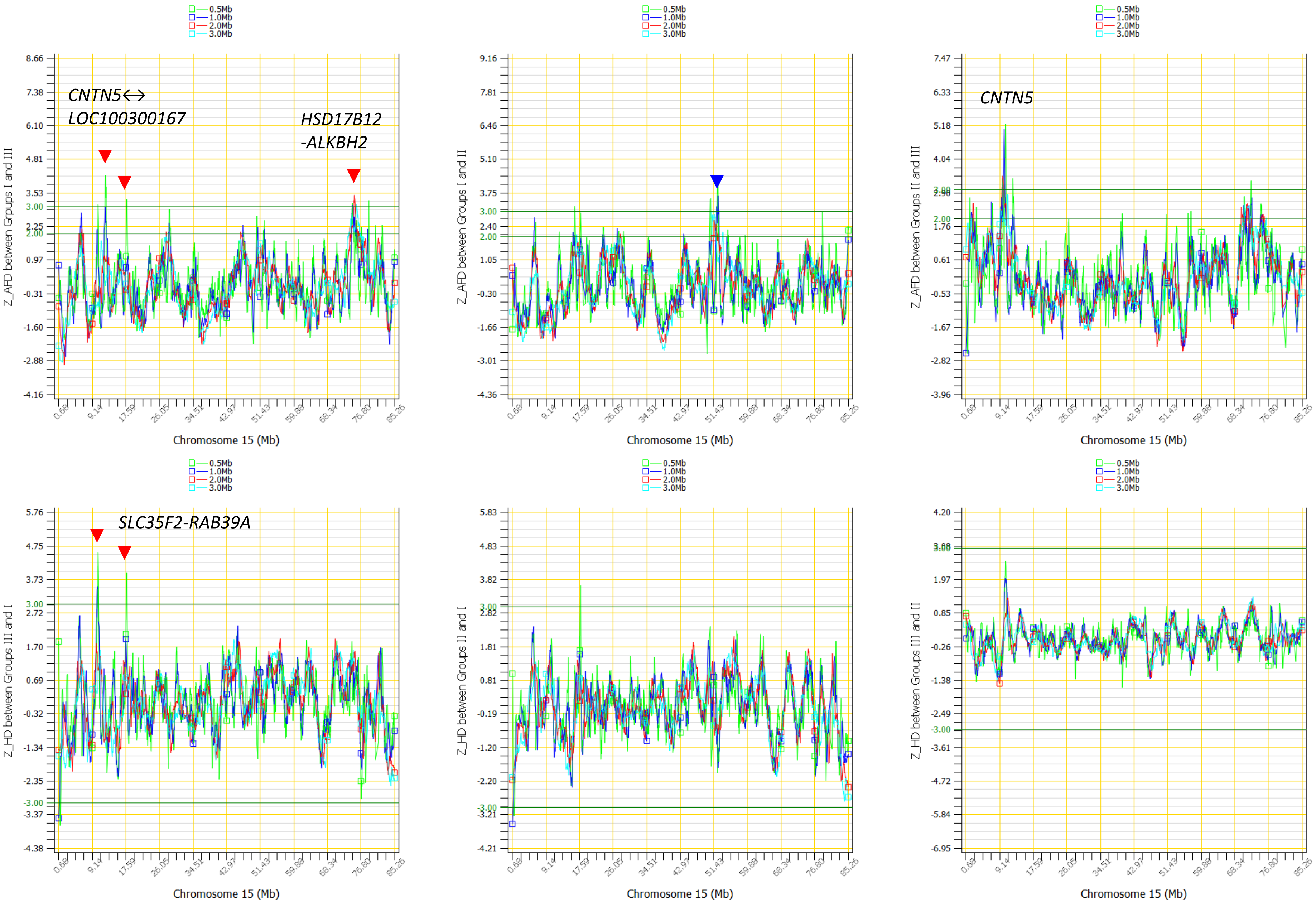
CNTN5↔
LOC100300167
HSD17B12
-ALKBH2
Z_AFD between Groups I and III
Chromosome 15 (Mb)
Z_AFD between Groups I and II
Chromosome 15 (Mb)
CNTN5
Z_AFD between Groups II and III
Chromosome 15 (Mb)
SLC35F2-RAB39A
Z_HD between Groups III and I
Chromosome 15 (Mb)
Z_HD between Groups II and I
Chromosome 15 (Mb)
Z_HD between Groups III and II
Chromosome 15 (Mb)
0.5Mb
1.0Mb
2.0Mb
3.0Mb

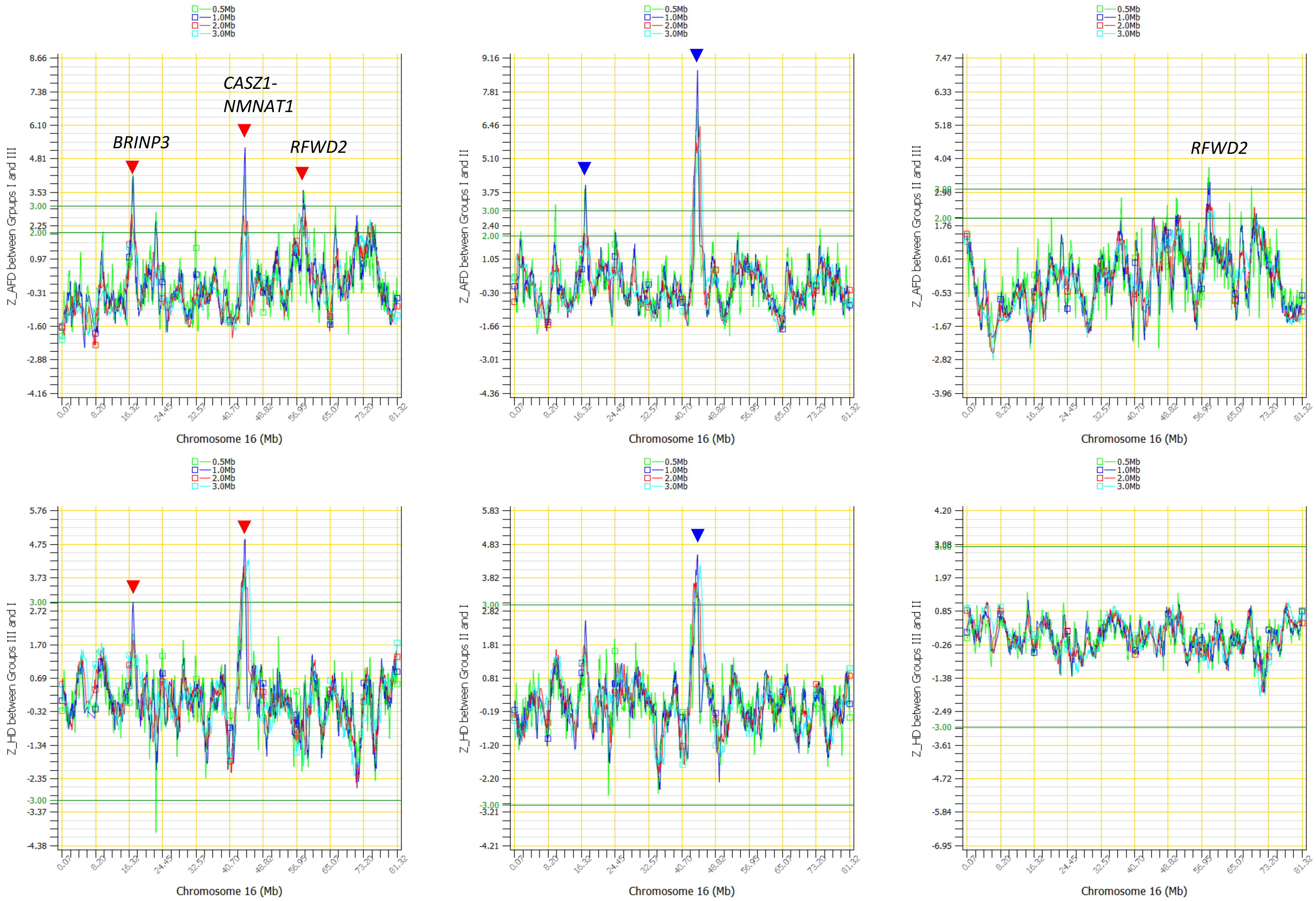
0.5Mb
1.0Mb
2.0Mb
3.0Mb
BRINP3
CASZ1-NMNAT1
RFWD2
Z_AFD between Grpups I and III
Z_AFD between Groups I and II
Z_AFD between Groups II and III
Z_HD between Groups III and I
Z_HD between Groups II and I
Z_HD between Groups III and II
Chromosome 16 (Mb)
0.07
8.20
16.32
24.45
32.57
40.70
48.82
56.95
65.07
73.20
81.32

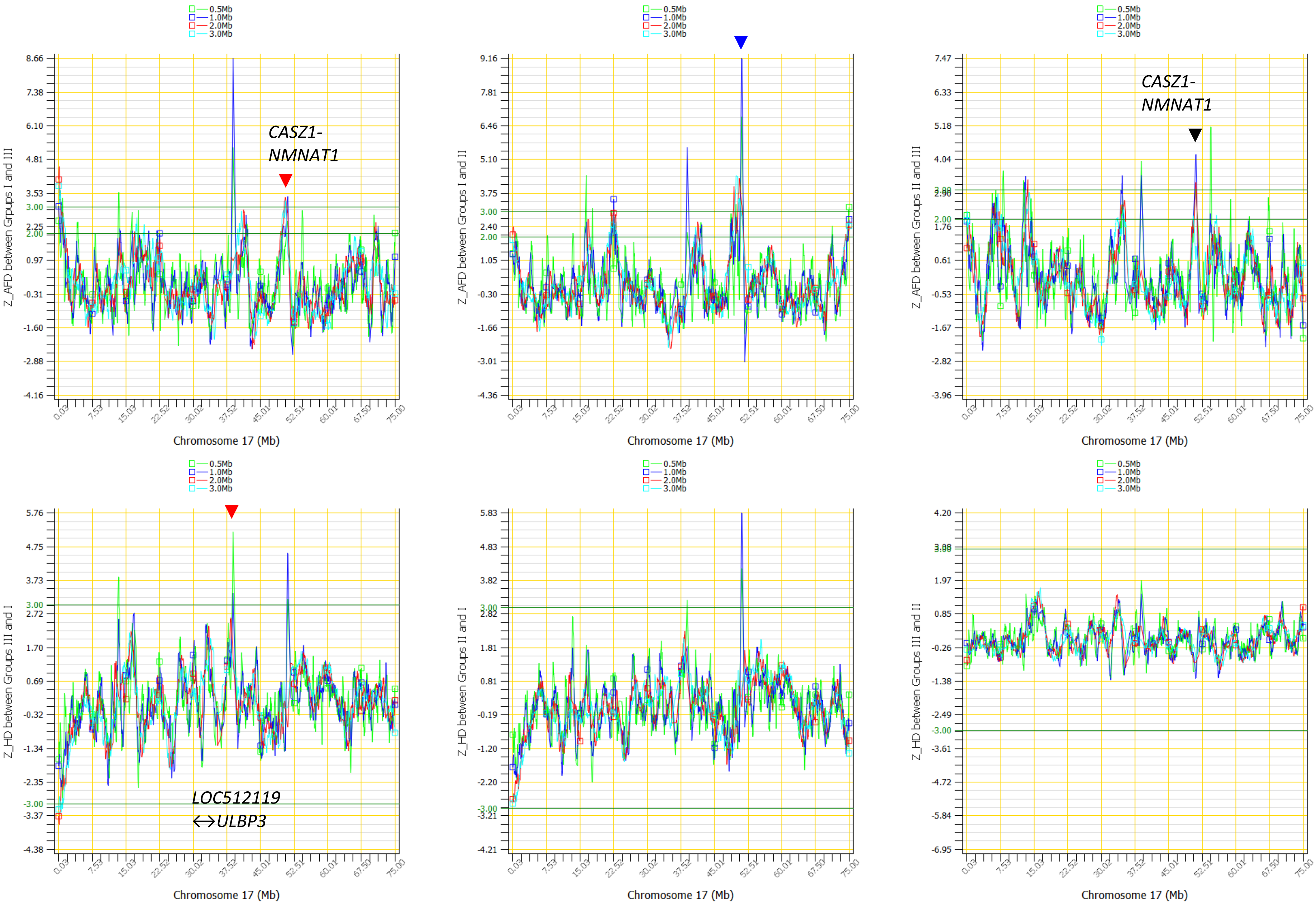
0.5Mb
1.0Mb
2.0Mb
3.0Mb
Z_AFD between Grpups I and III
CASZ1-
NMNAT1
Chromosome 17 (Mb)
Z_AFD between Groups I and II
Z_AFD between Groups II and III
CASZ1-
NMNAT1
Z_HD between Groups III and I
LOC512119
↔ULBP3
Z_HD between Groups II and I
Z_HD between Groups III and II

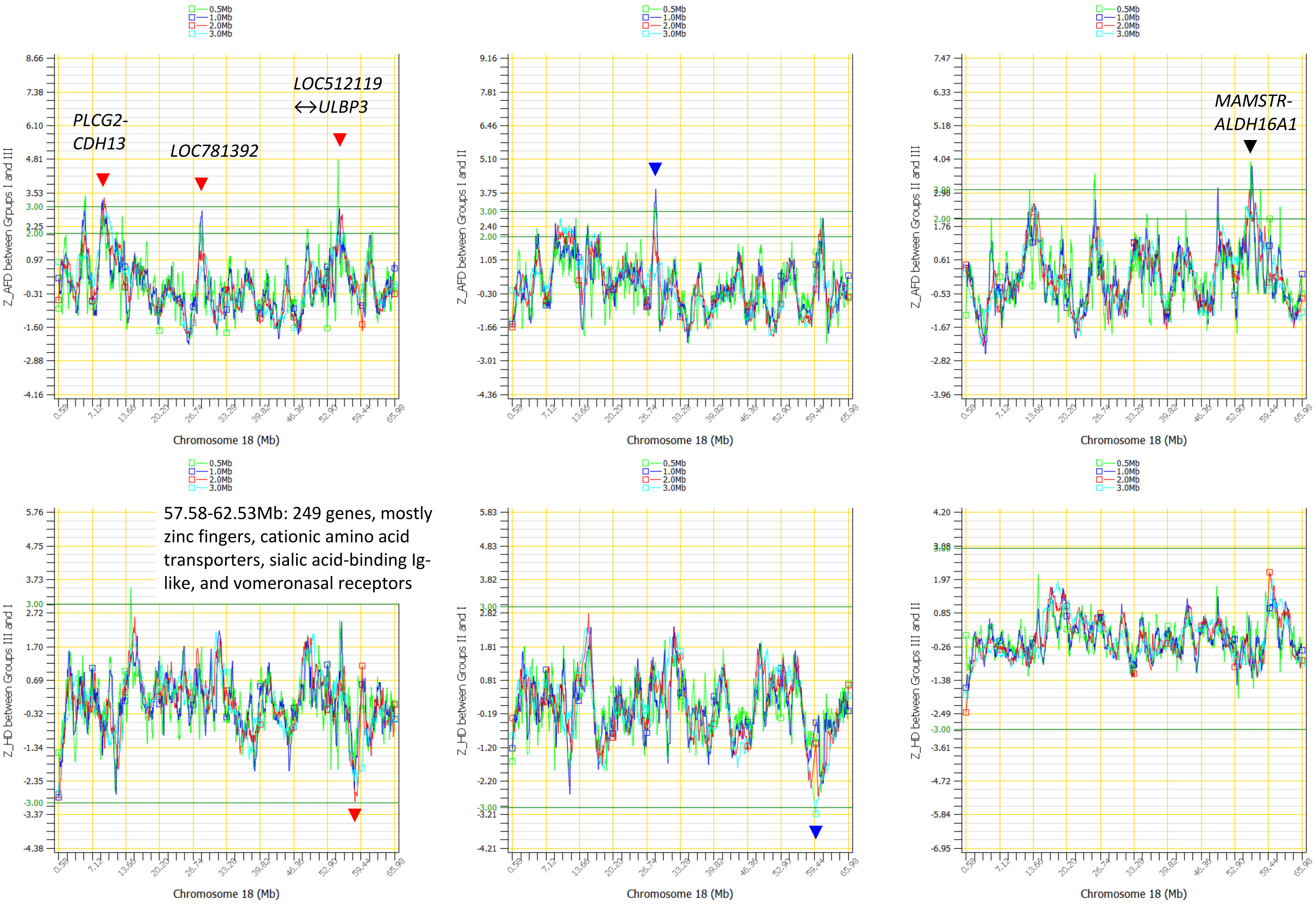

0.5Mb
1.0Mb
2.0Mb
3.0Mb
PLCG2-
CDH13
LOC781392
LOC512119
↔ULBP3
MAMSTR-
ALDH16A1
57.58-62.53Mb: 249 genes, mostly zinc fingers, cationic amino acid transporters, sialic acid-binding Ig-like, and vomeronasal receptors
Z_AFD between Groups I and III
Z_AFD between Groups I and II
Z_AFD between Groups II and III
Z_HD between Groups III and I
Z_HD between Groups II and I
Z_HD between Groups III and II
Chromosome 18 (Mb)

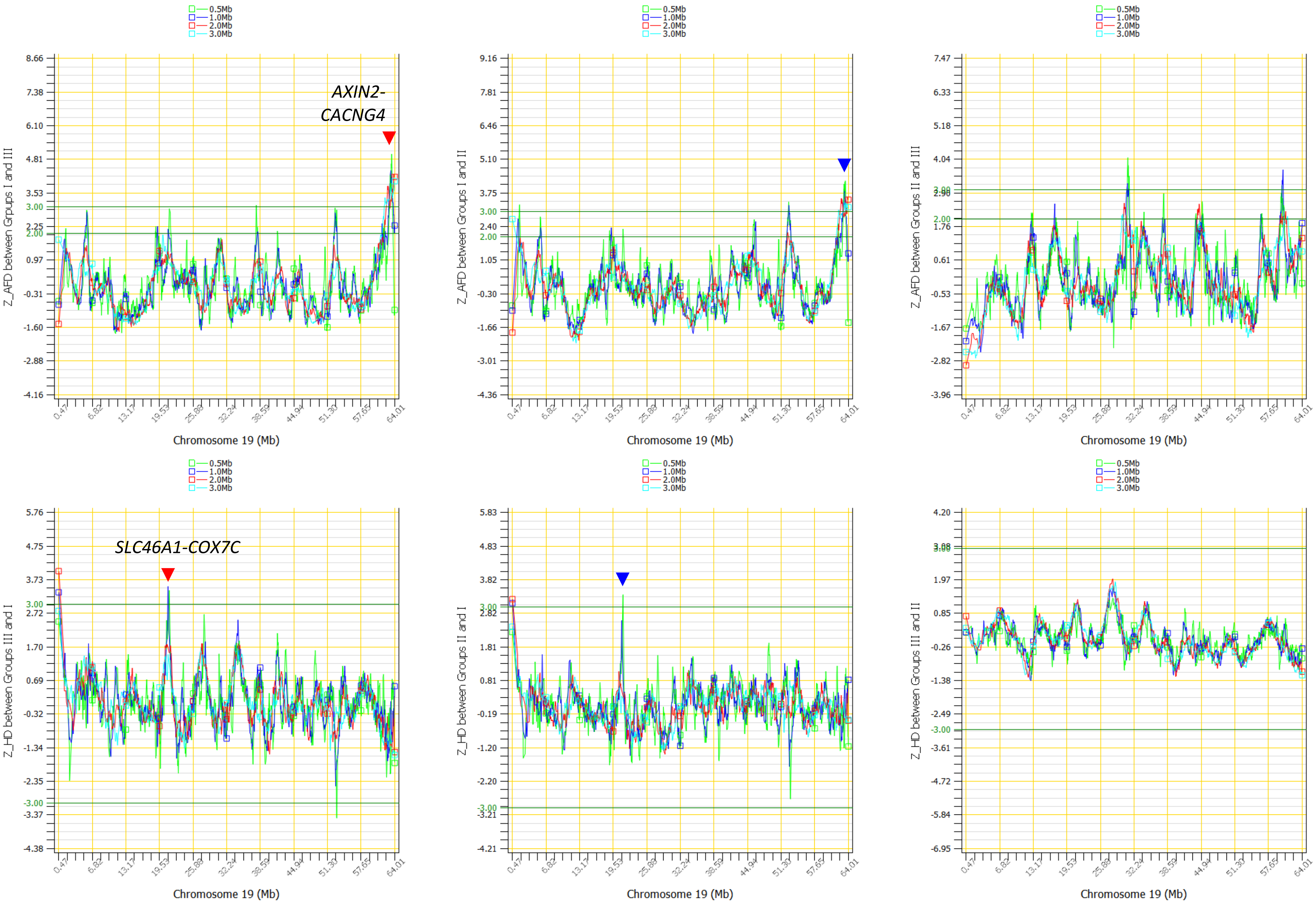

0.5Mb
1.0Mb
2.0Mb
3.0Mb
AXIN2-
CACNG4
Z_AFD between Groups I and III
Chromosome 19 (Mb)
Z_AFD between Groups I and II
Chromosome 19 (Mb)
Z_AFD between Groups II and III
Chromosome 19 (Mb)
SLC46A1-COX7C
Z_HD between Groups III and I
Chromosome 19 (Mb)
Z_HD between Groups II and I
Chromosome 19 (Mb)
Z_HD between Groups III and II
Chromosome 19 (Mb)

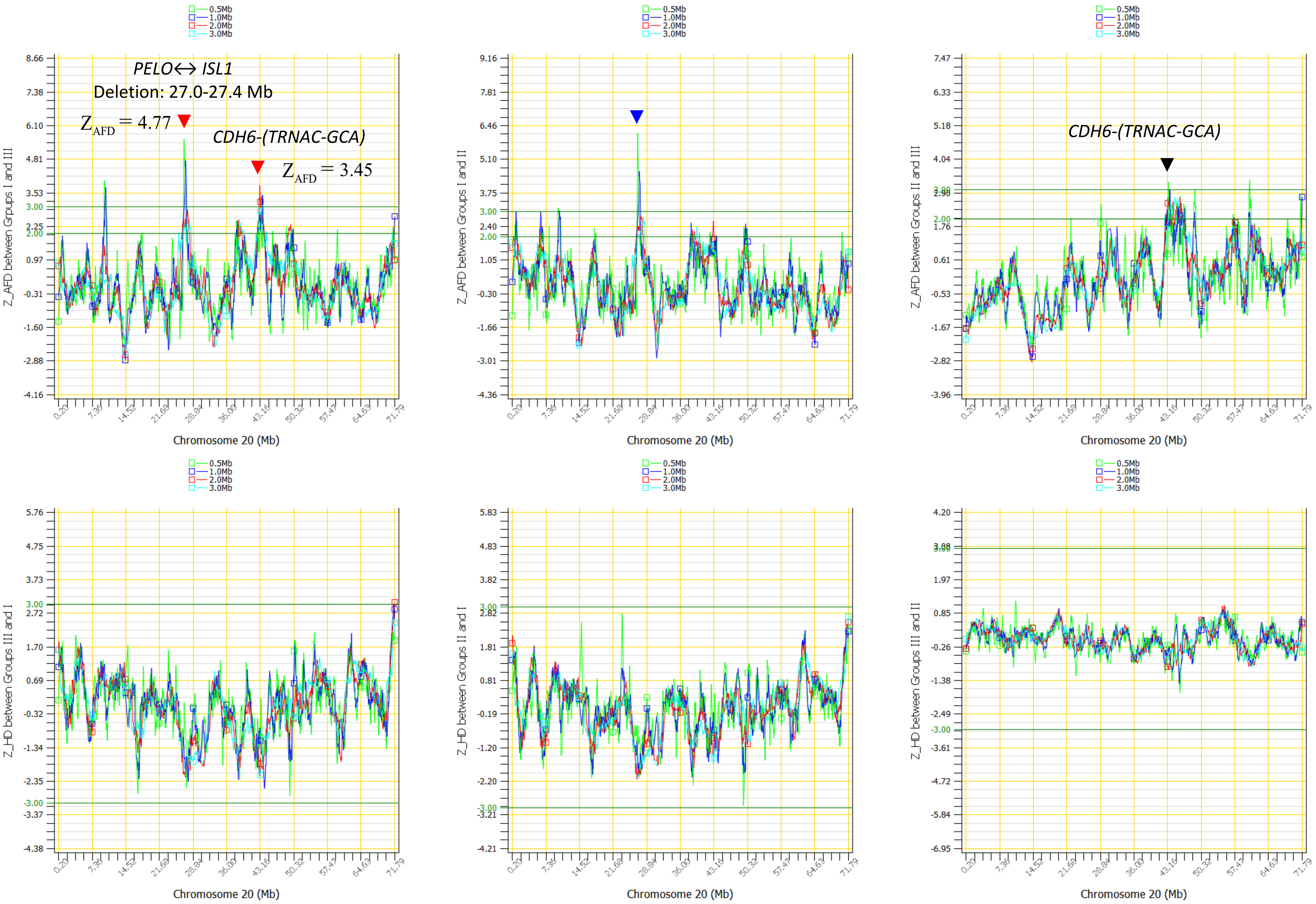
0.5Mb
1.0Mb
2.0Mb
3.0Mb
PELO↔ ISL1
Deletion: 27.0-27.4 Mb
Z_{AFD} = 4.77
CDH6-(TRNAC-GCA)
Z_{AFD} = 3.45
Z_AFD between Grpups I and III
Z_AFD between Groups I and II
Z_AFD between Groups II and III
Z_HD between Groups III and I
Z_HD between Groups II and I
Z_HD between Groups III and II
Chromosome 20 (Mb)
0.20
7.36
14.52
21.68
28.84
36.00
43.16
50.32
57.47
64.63
71.79

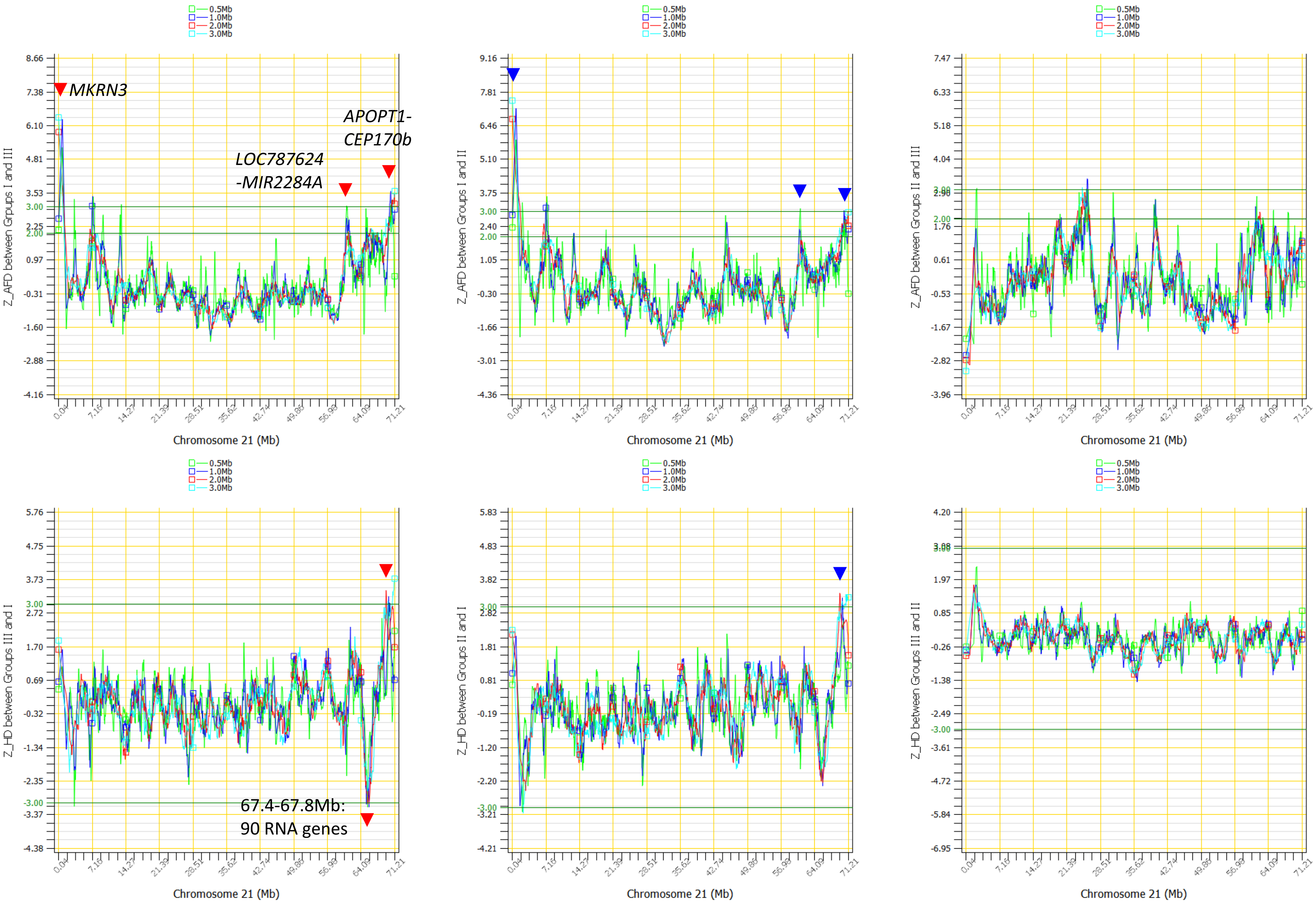
0.5Mb
1.0Mb
2.0Mb
3.0Mb
Z_AFD between Grpups I and III
MKRN3
LOC787624
-MIR2284A
APOPT1-
CEP170b
Chromosome 21 (Mb)
Z_AFD between Groups I and II
Z_AFD between Groups II and III
Z_HD between Groups III and I
67.4-67.8Mb:
90 RNA genes
Z_HD between Groups II and I
Z_HD between Groups III and II

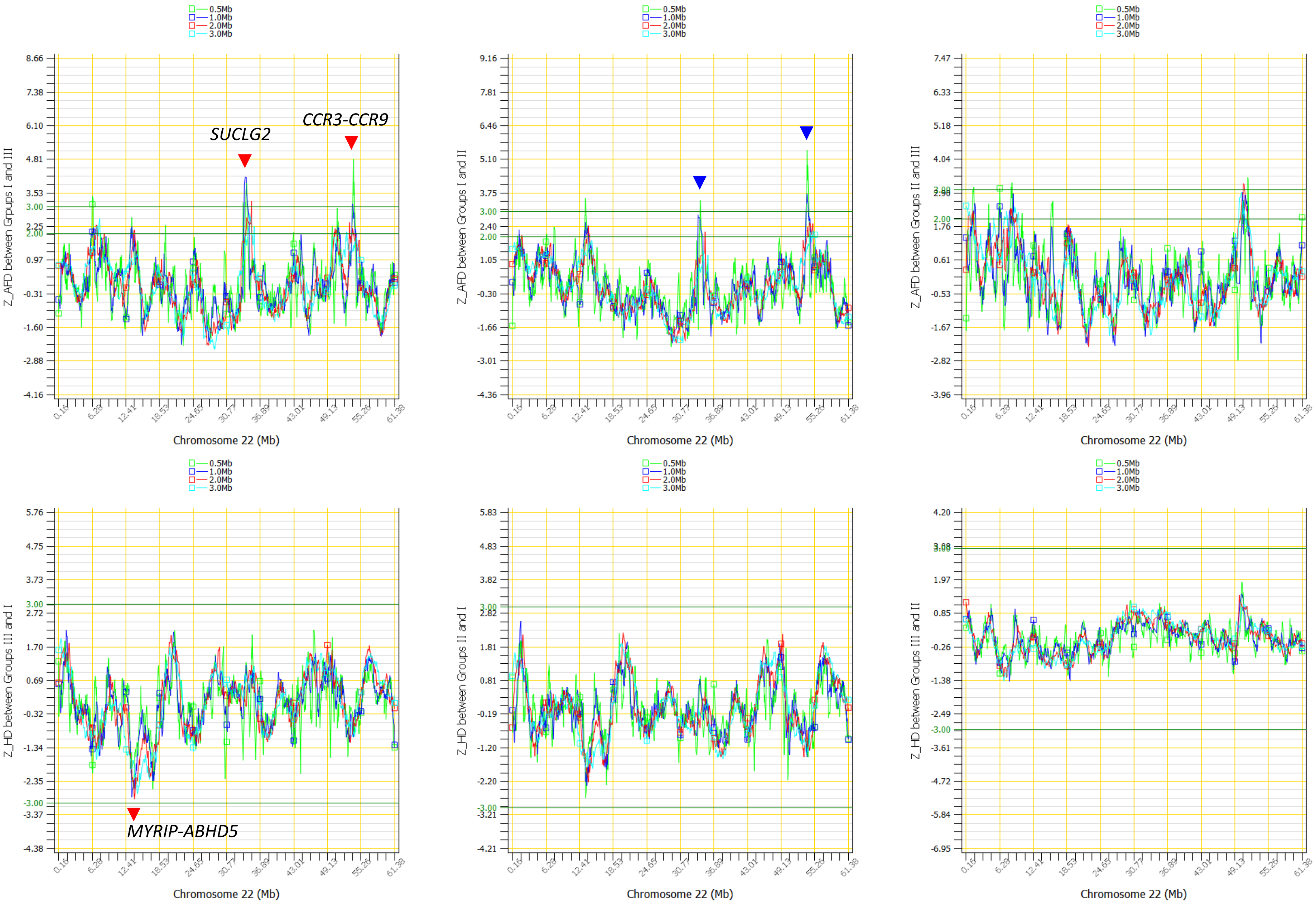
0.5Mb
1.0Mb
2.0Mb
3.0Mb
Z_AFD between Groups I and III
Z_AFD between Groups I and II
Z_AFD between Groups II and III
Z_HD between Groups III and I
Z_HD between Groups II and I
Z_HD between Groups III and II
Chromosome 22 (Mb)
SUCLG2
CCR3-CCR9
MYRIP-ABHD5
0.16
6.28
12.41
18.53
24.65
30.77
36.89
43.01
49.13
55.26
61.38

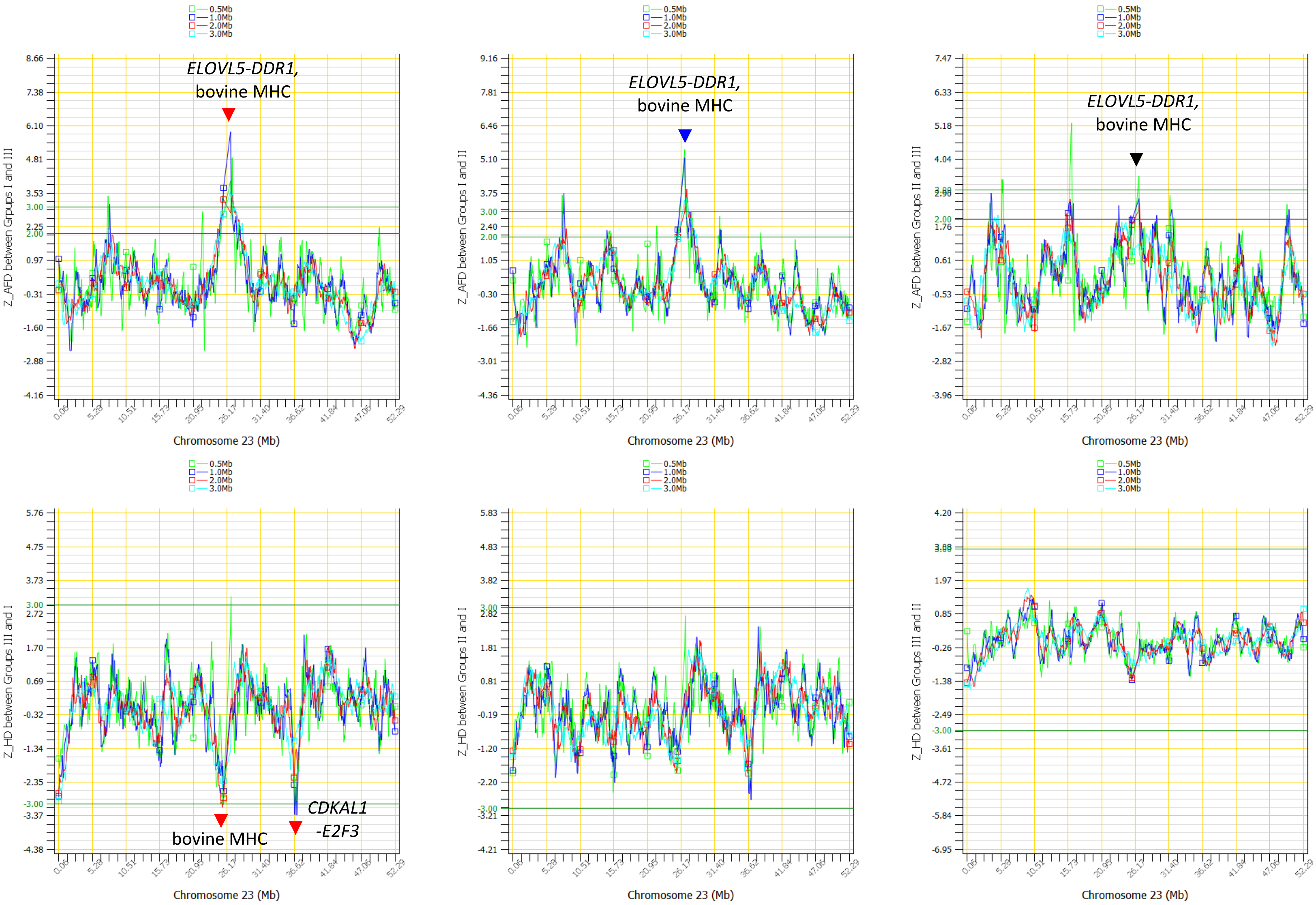

0.5Mb
1.0Mb
2.0Mb
3.0Mb
ELOVL5-DDR1,
bovine MHC
Z_AFD between Grpups I and III
Chromosome 23 (Mb)
ELOVL5-DDR1,
bovine MHC
Z_AFD between Groups I and II
Chromosome 23 (Mb)
ELOVL5-DDR1,
bovine MHC
Z_AFD between Groups II and III
Chromosome 23 (Mb)
Z_HD between Groups III and I
bovine MHC
CDKAL1
-E2F3
Chromosome 23 (Mb)
Z_HD between Groups II and I
Chromosome 23 (Mb)
Z_HD between Groups III and II
Chromosome 23 (Mb)

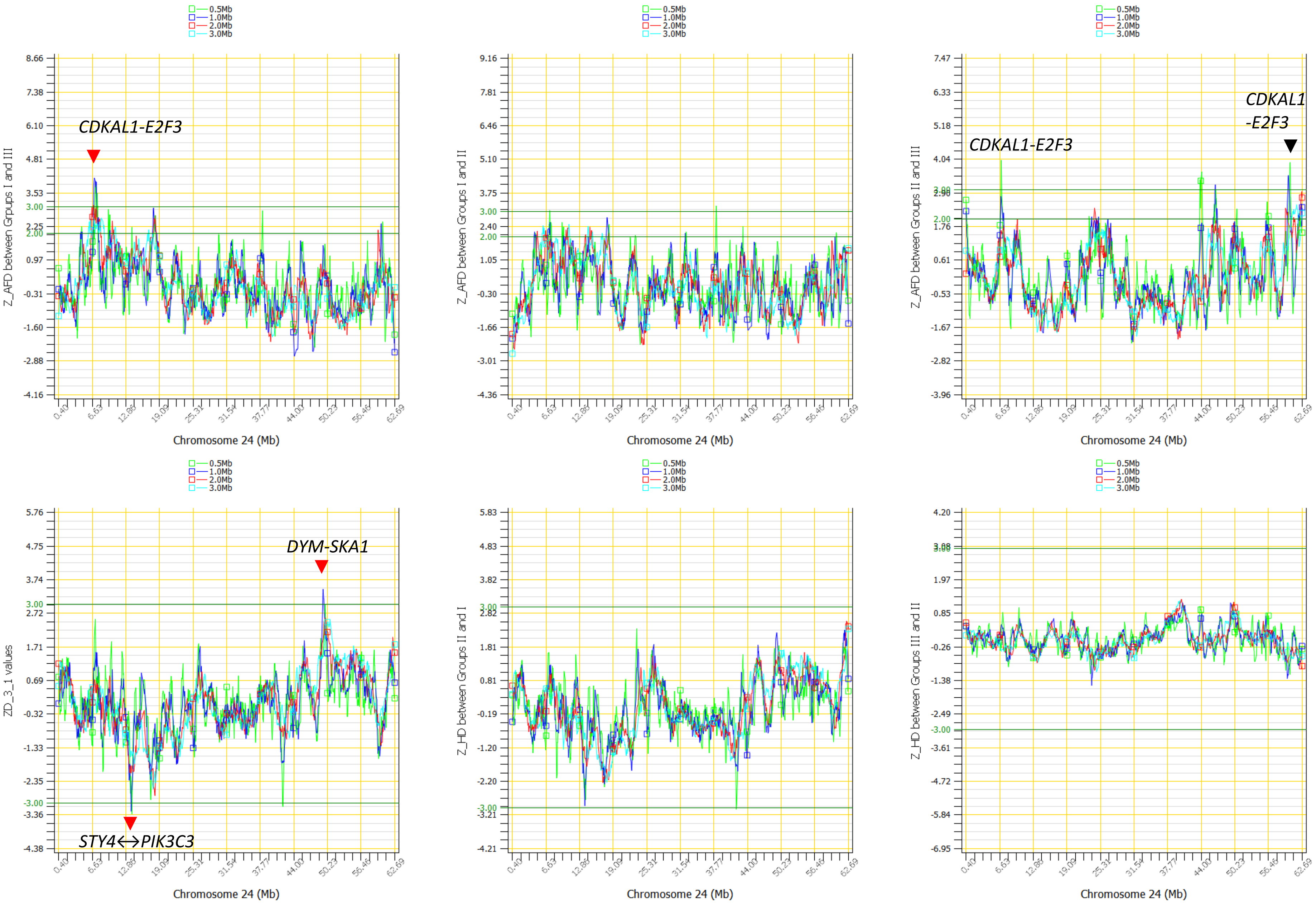
0.5Mb
1.0Mb
2.0Mb
3.0Mb
Z_AFD between Grpups I and III
CDKAL1-E2F3
8.66
7.38
6.10
4.81
3.53
3.00
2.25
2.00
0.97
-0.31
-1.60
-2.88
-4.16
Chromosome 24 (Mb)
0.40
6.63
12.86
19.09
25.31
31.54
37.77
44.00
50.23
56.46
62.69
Z_AFD between Groups I and II
9.16
7.81
6.46
5.10
3.75
3.00
2.40
2.00
1.05
-0.30
-1.66
-3.01
-4.36
Z_AFD between Groups II and III
CDKAL1-E2F3
CDKAL1
-E2F3
7.47
6.33
5.18
4.04
2.00
1.76
0.61
-0.53
-1.67
-2.82
-3.96
ZD_3_1 values
DYM-SKA1
STY4↔PIK3C3
5.76
4.75
3.74
3.00
2.72
1.71
0.69
-0.32
-1.33
-2.35
-3.00
-3.36
-4.38
Z_HD between Groups II and I
5.83
4.83
3.82
3.00
2.82
1.81
0.81
-0.19
-1.20
-2.20
-3.00
-3.21
-4.21
Z_HD between Groups III and II
4.20
3.08
1.97
0.85
-0.26
-1.38
-2.49
-3.00
-3.61
-4.72
-5.84
-6.95

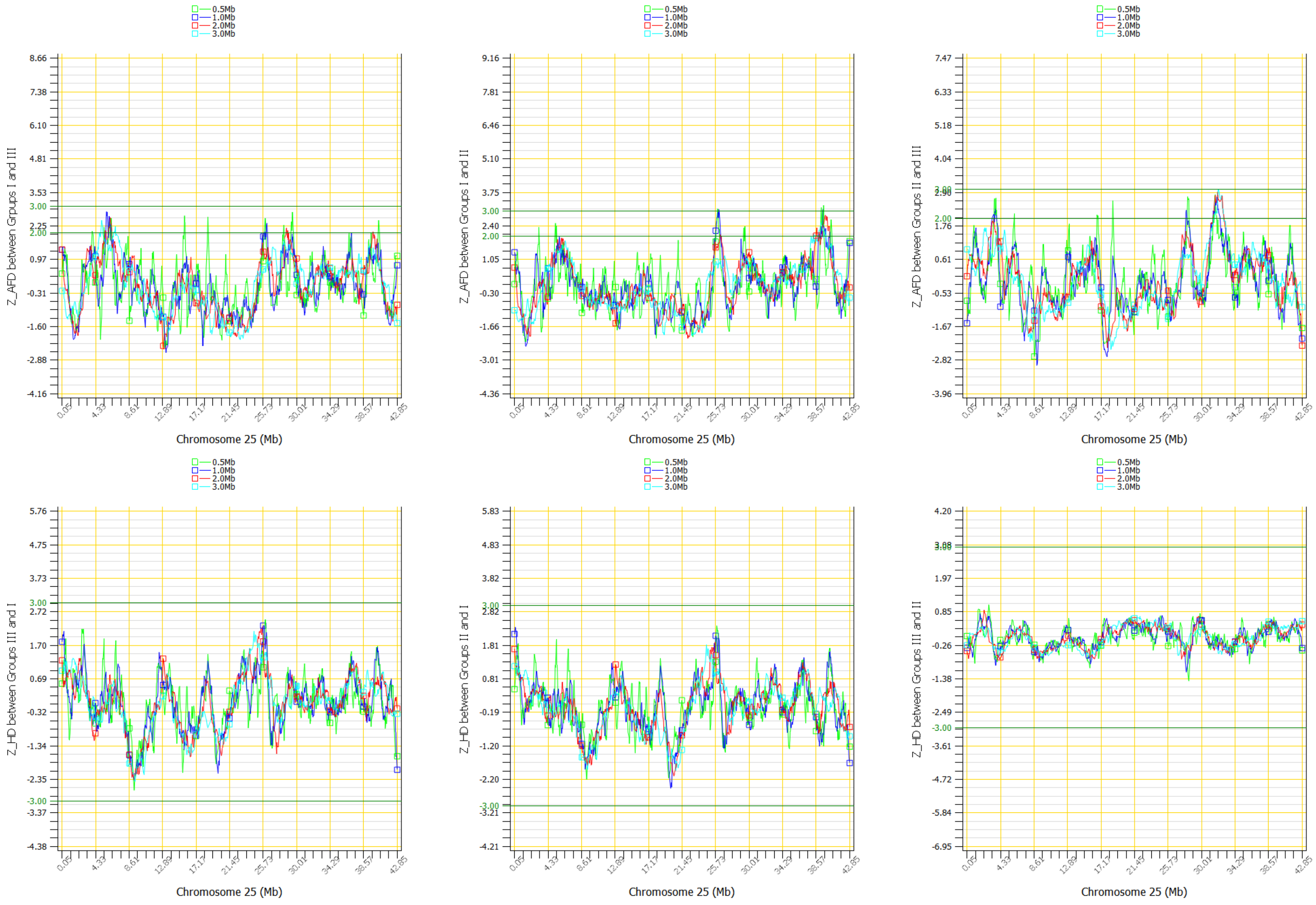
0.5Mb
1.0Mb
2.0Mb
3.0Mb
Z_AFD between Grpups I and III
Chromosome 25 (Mb)
Z_AFD between Groups I and II
Chromosome 25 (Mb)
Z_AFD between Groups II and III
Chromosome 25 (Mb)
Z_HD between Groups III and I
Chromosome 25 (Mb)
Z_HD between Groups II and I
Chromosome 25 (Mb)
Z_HD between Groups III and II
Chromosome 25 (Mb)

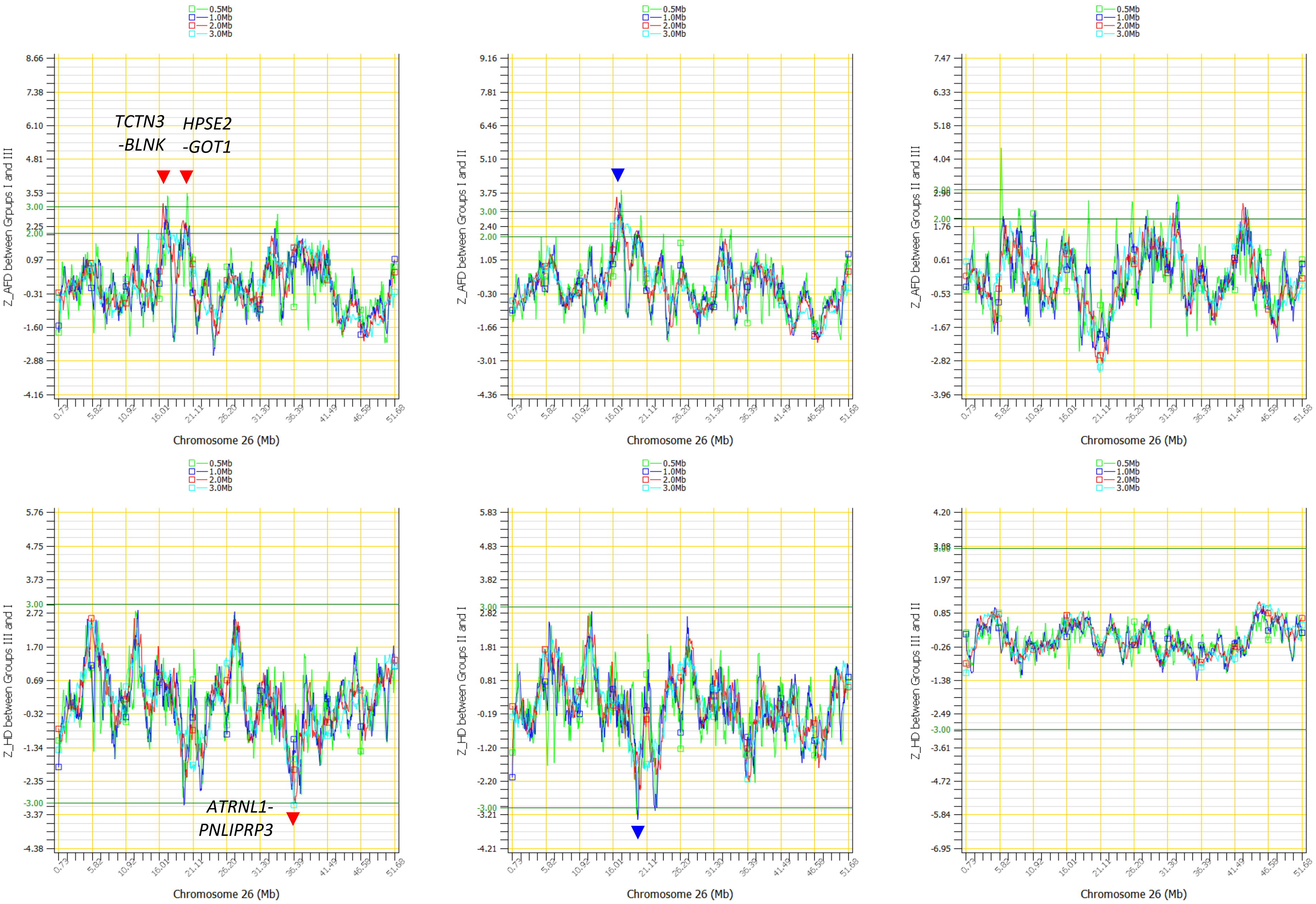
0.5Mb
1.0Mb
2.0Mb
3.0Mb
Z_AFD between Grpups I and III
TCTN3
-BLNK
HPSE2
-GOT1
Chromosome 26 (Mb)
Z_AFD between Groups I and II
Chromosome 26 (Mb)
Z_AFD between Groups II and III
Chromosome 26 (Mb)
Z_HD between Groups III and I
ATRNL1-
PNLIPRP3
Chromosome 26 (Mb)
Z_HD between Groups II and I
Chromosome 26 (Mb)
Z_HD between Groups III and II
Chromosome 26 (Mb)

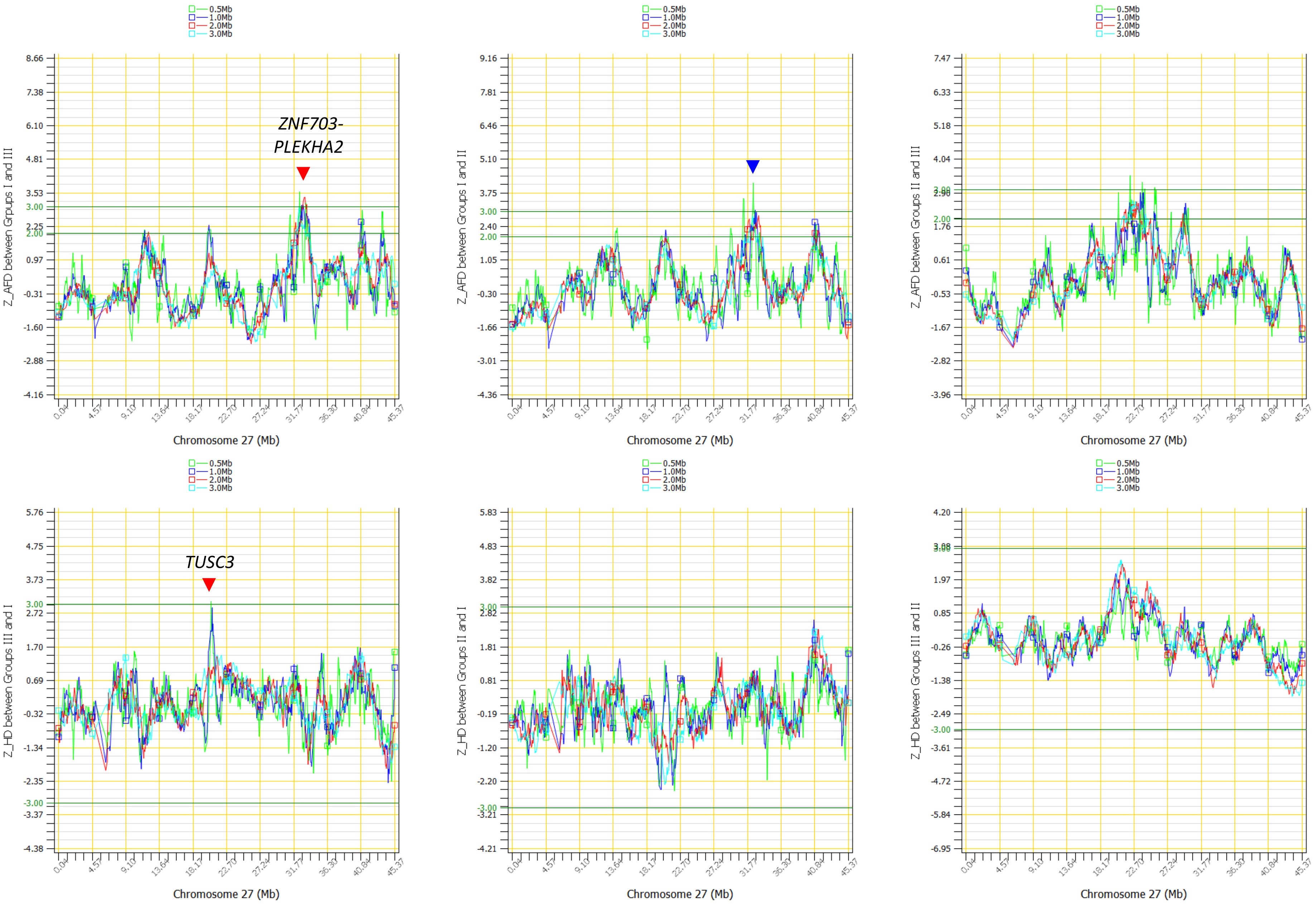

0.5Mb
1.0Mb
2.0Mb
3.0Mb
Z_AFD between Grpups I and III
ZNF703-PLEKHA2
Chromosome 27 (Mb)
Z_AFD between Groups I and II
Chromosome 27 (Mb)
Z_AFD between Groups II and III
Chromosome 27 (Mb)
Z_HD between Groups III and I
TUSC3
Chromosome 27 (Mb)
Z_HD between Groups II and I
Chromosome 27 (Mb)
Z_HD between Groups III and II
Chromosome 27 (Mb)

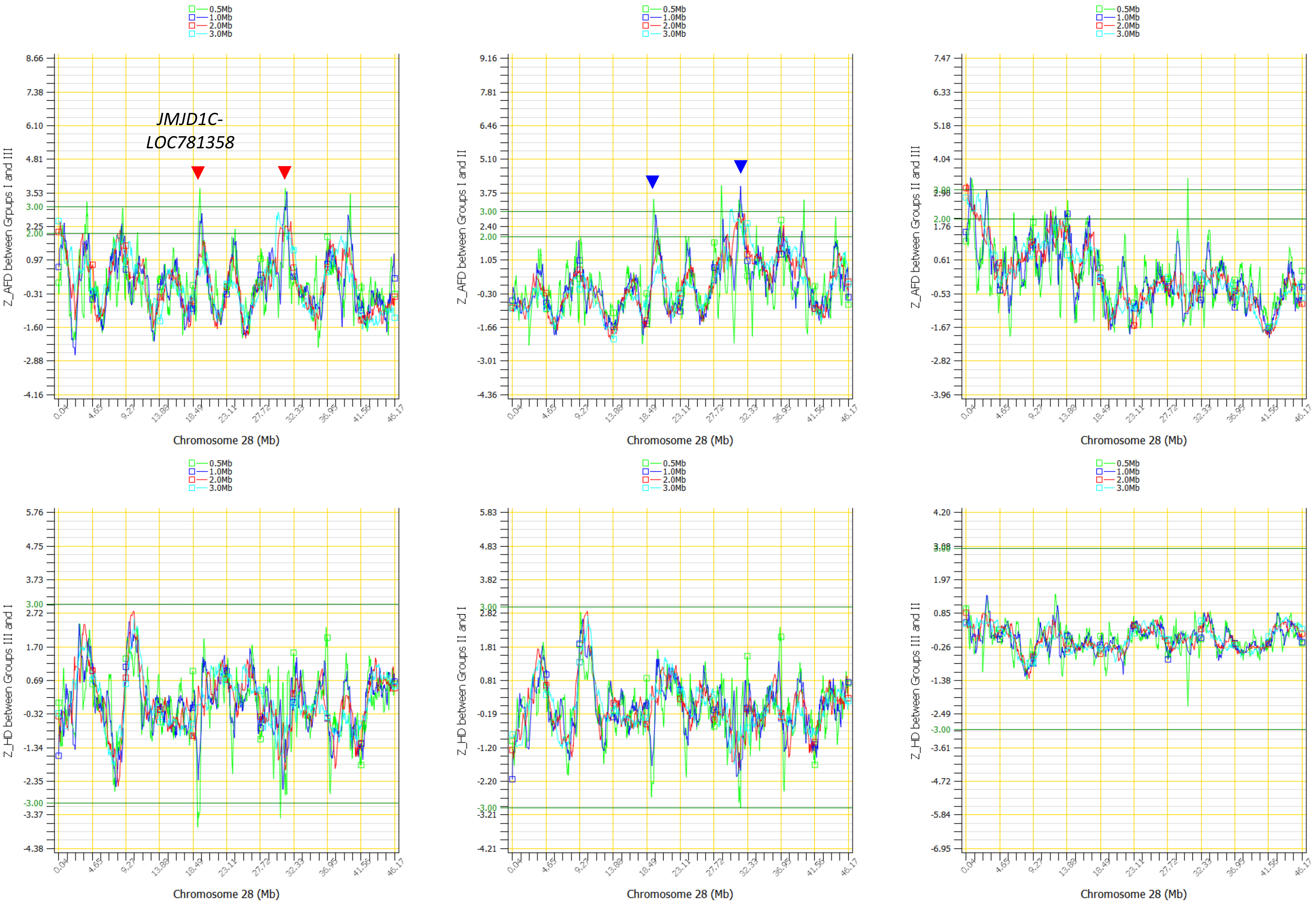
0.5Mb
1.0Mb
2.0Mb
3.0Mb
JMJD1C-
LOC781358
Z_AFD between Groups I and III
Z_AFD between Groups I and II
Z_AFD between Groups II and III
Z_HD between Groups III and I
Z_HD between Groups II and I
Z_HD between Groups III and II
Chromosome 28 (Mb)
0.04
4.65
9.27
13.88
18.49
23.11
27.72
32.33
36.95
41.56
46.17

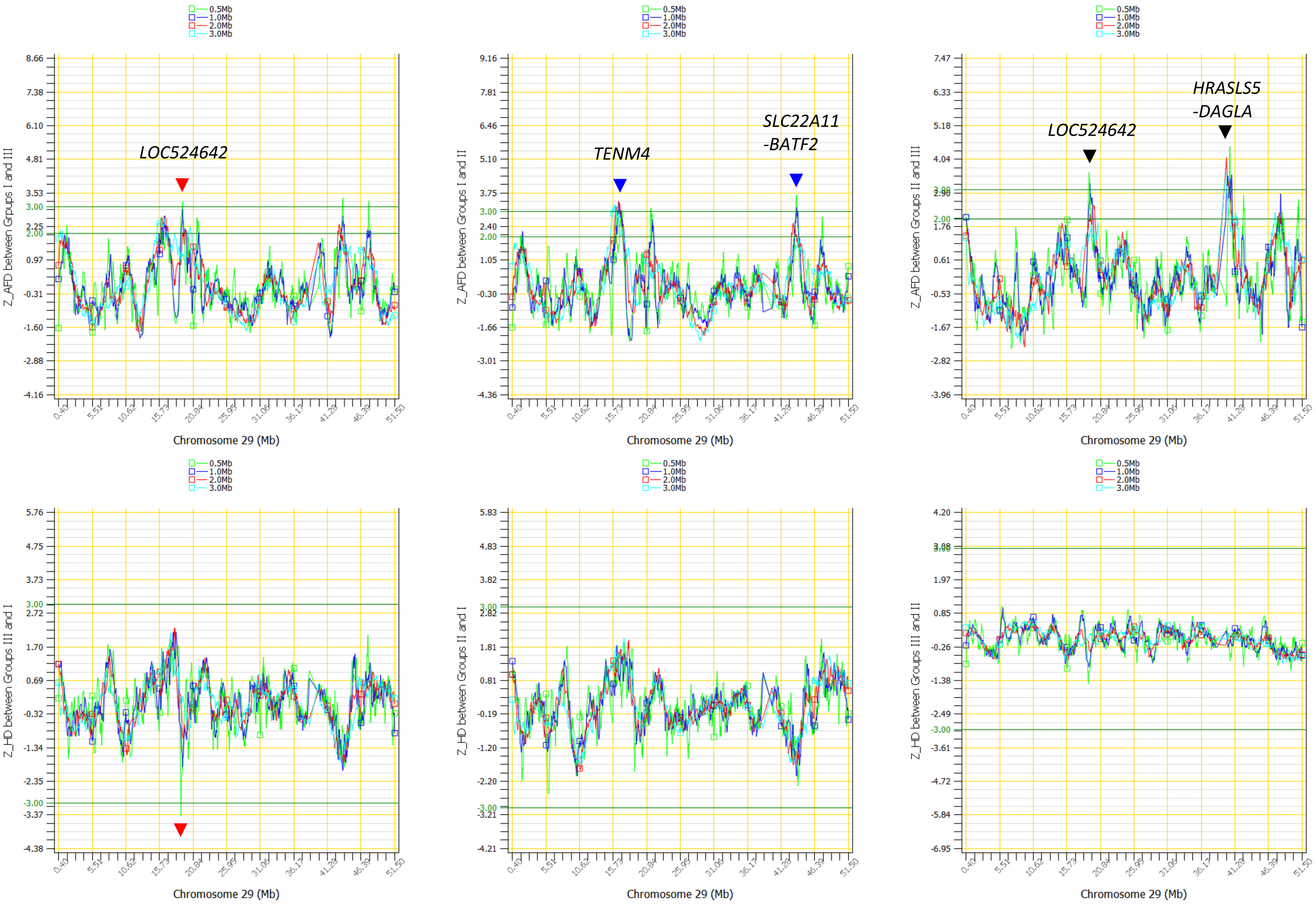

0.5Mb
1.0Mb
2.0Mb
3.0Mb
Z_AFD between Grpups I and III
LOC524642
Chromosome 29 (Mb)
Z_AFD between Groups I and II
TENM4
SLC22A11
-BATF2
Chromosome 29 (Mb)
Z_AFD between Groups II and III
LOC524642
HRASLS5
-DAGLA
Chromosome 29 (Mb)
Z_HD between Groups III and I
Chromosome 29 (Mb)
Z_HD between Groups II and I
Chromosome 29 (Mb)
Z_HD between Groups III and II
Chromosome 29 (Mb)

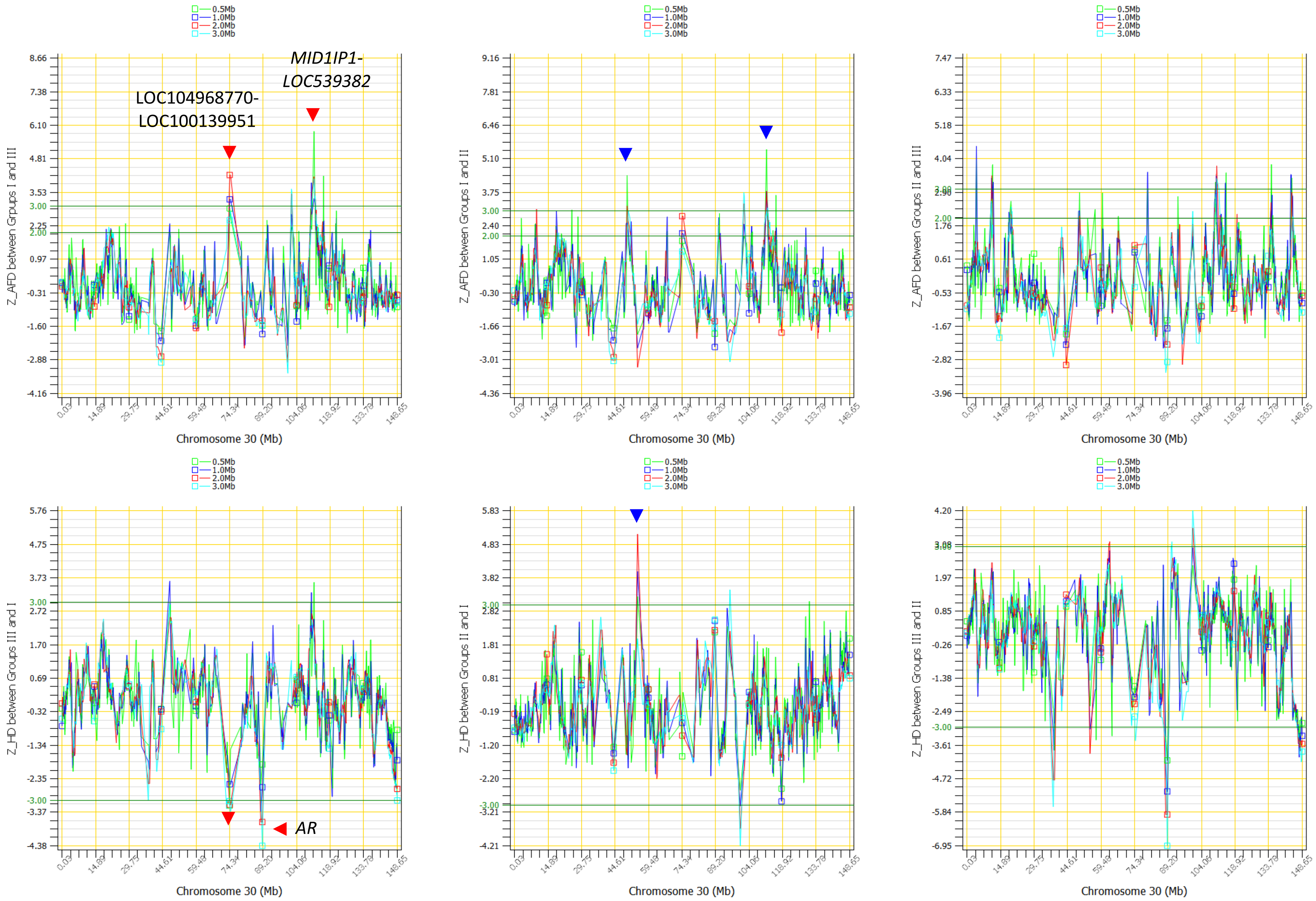

Fig. S6. Long-range differences of allele frequencies and heterozygosity between unselected and selected Holsteins since 1964. Left column: 40 years of selection between Groups I and III. Middle column: the first 20 years of selection between Groups I and II. Right column: the second 20 years of selection between Groups II and III. Chr30 is the X chromosome.
