# Supplementary material for: Genome changes due to artificial selection in U.S. Holstein cattle
Source: BMC Genomics. 2019 Feb 11;20:128. doi: 10.1186/s12864-019-5459-x (PMC6371544; doi:10.1186/s12864-019-5459-x)
Supplement: Supplementary file 7 — Figure S7. Long-range differences of allele frequencies and heterozygosity between an elite group (Group IIIb) and the other groups in the selection signature analysis. Left column: the 40 years of selection between Groups IIIb and I. Middle column: the second 20 years of selection between Groups IIIb and II. Right column: the difference between the elite group and their contemporaries (Groups IIIb and IIIa). Chr30 is the X chromosome. (PDF 19946 kb) [file 12864_2019_5459_MOESM7_ESM.pdf]

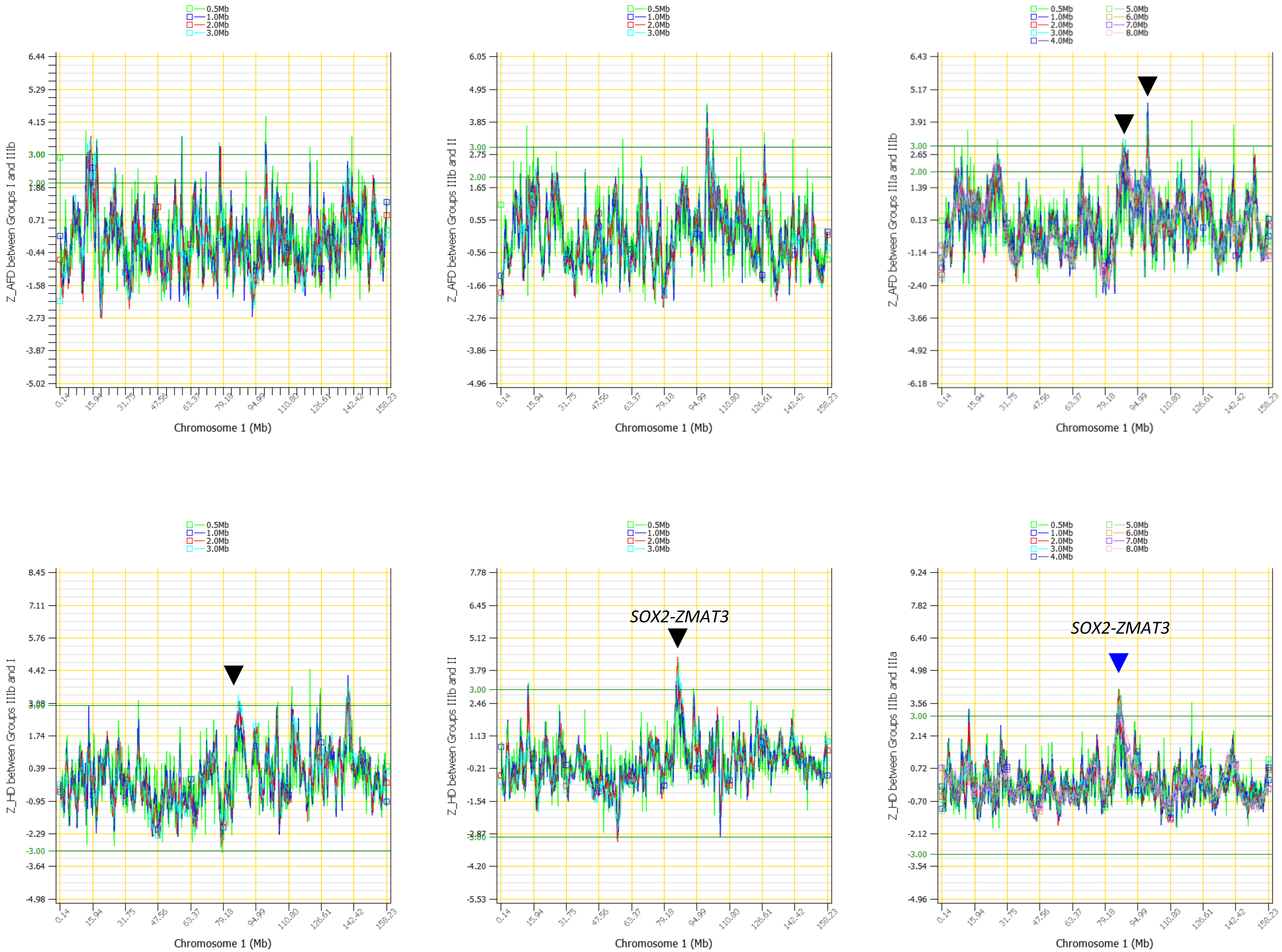

0.5Mb
1.0Mb
2.0Mb
3.0Mb
4.0Mb
5.0Mb
6.0Mb
7.0Mb
8.0Mb
Z_AFD between Groups I and IIIb
Z_AFD between Groups IIIb and II
Z_AFD between Groups IIIa and IIIb
Z_HD between Groups IIIb and I
Z_HD between Groups IIIb and II
Z_HD between Groups IIIb and IIIa
SOX2-ZMAT3
SOX2-ZMAT3
Chromosome 1 (Mb)
0.14
15.94
31.75
47.56
63.37
79.18
94.99
110.80
126.61
142.42
158.23

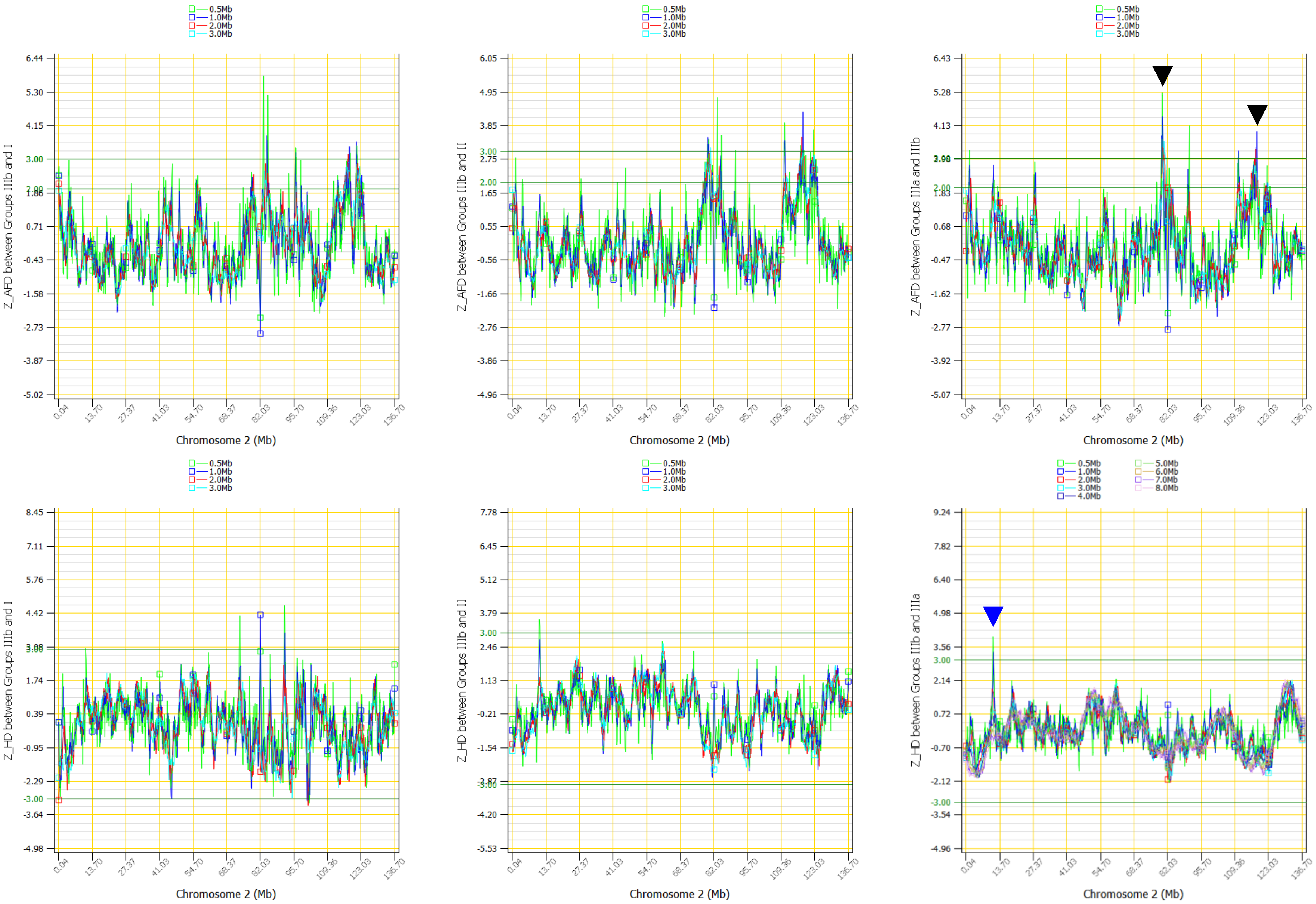

0.5Mb
1.0Mb
2.0Mb
3.0Mb
Z_AFD between Groups IIIb and I
6.44 5.30 4.15 3.00 2.00 1.86 0.71 -0.43 -1.58 -2.73 -3.87 -5.02
Chromosome 2 (Mb)
0.04 13.70 27.37 41.03 54.70 68.37 82.03 95.70 109.36 123.03 136.70
0.5Mb
1.0Mb
2.0Mb
3.0Mb
Z_AFD between Groups IIIb and II
6.05 4.95 3.85 3.00 2.75 2.00 1.65 0.55 -0.56 -1.66 -2.76 -3.86 -4.96
Chromosome 2 (Mb)
0.04 13.70 27.37 41.03 54.70 68.37 82.03 95.70 109.36 123.03 136.70
0.5Mb
1.0Mb
2.0Mb
3.0Mb
Z_AFD between Groups IIIa and IIIb
6.43 5.28 4.13 2.00 1.83 0.68 -0.47 -1.62 -2.77 -3.92 -5.07
Chromosome 2 (Mb)
0.04 13.70 27.37 41.03 54.70 68.37 82.03 95.70 109.36 123.03 136.70
0.5Mb
1.0Mb
2.0Mb
3.0Mb
Z_HD between Groups IIIb and I
8.45 7.11 5.76 4.42 3.08 1.74 0.39 -0.95 -2.29 -3.00 -3.64 -4.98
Chromosome 2 (Mb)
0.04 13.70 27.37 41.03 54.70 68.37 82.03 95.70 109.36 123.03 136.70
0.5Mb
1.0Mb
2.0Mb
3.0Mb
Z_HD between Groups IIIb and II
7.78 6.45 5.12 3.79 3.00 2.46 1.13 -0.21 -1.54 -4.20 -5.53
Chromosome 2 (Mb)
0.04 13.70 27.37 41.03 54.70 68.37 82.03 95.70 109.36 123.03 136.70
0.5Mb
1.0Mb
2.0Mb
3.0Mb
4.0Mb
5.0Mb
6.0Mb
7.0Mb
8.0Mb
Z_HD between Groups IIIb and IIIa
9.24 7.82 6.40 4.98 3.56 3.00 2.14 0.72 -0.70 -2.12 -3.00 -3.54 -4.96
Chromosome 2 (Mb)
0.04 13.70 27.37 41.03 54.70 68.37 82.03 95.70 109.36 123.03 136.70

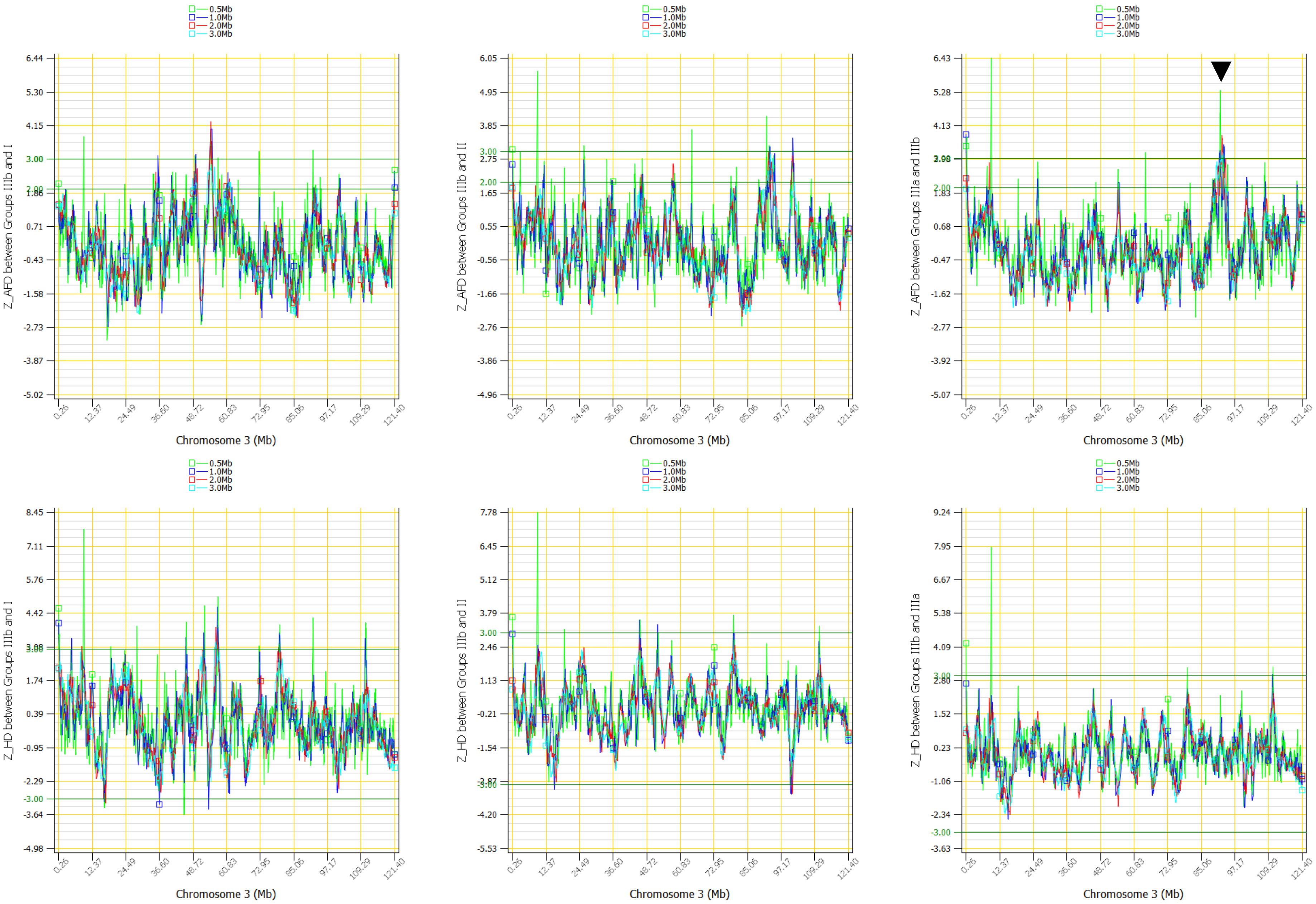

0.5Mb
1.0Mb
2.0Mb
3.0Mb
Z_AFD between Groups IIIb and I
Chromosome 3 (Mb)
Z_AFD between Groups IIIb and II
Chromosome 3 (Mb)
Z_AFD between Groups IIIa and IIIb
Chromosome 3 (Mb)
Z_HD between Groups IIIb and I
Chromosome 3 (Mb)
Z_HD between Groups IIIb and II
Chromosome 3 (Mb)
Z_HD between Groups IIIb and IIIa
Chromosome 3 (Mb)
0.26
12.37
24.49
36.60
48.72
60.83
72.95
85.06
97.17
109.29
121.40

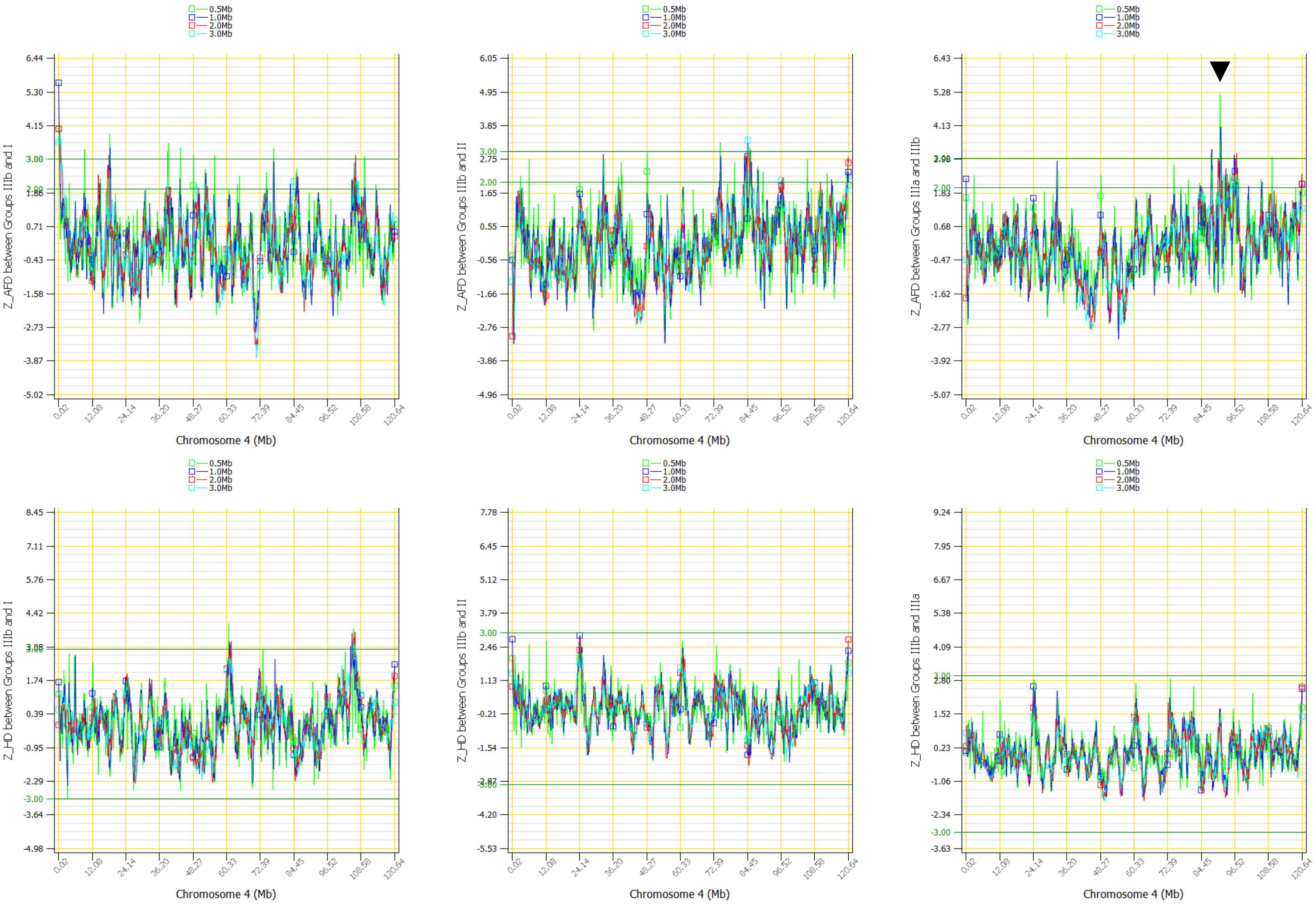

0.5Mb
1.0Mb
2.0Mb
3.0Mb
Z_AFD between Groups IIIb and I
Chromosome 4 (Mb)
Z_AFD between Groups IIIb and II
Chromosome 4 (Mb)
Z_AFD between Groups IIIa and IIIb
Chromosome 4 (Mb)
Z_HD between Groups IIIb and I
Chromosome 4 (Mb)
Z_HD between Groups IIIb and II
Chromosome 4 (Mb)
Z_HD between Groups IIIb and IIIa
Chromosome 4 (Mb)
0.02
12.08
24.14
36.20
48.27
60.33
72.39
84.45
96.52
108.58
120.64

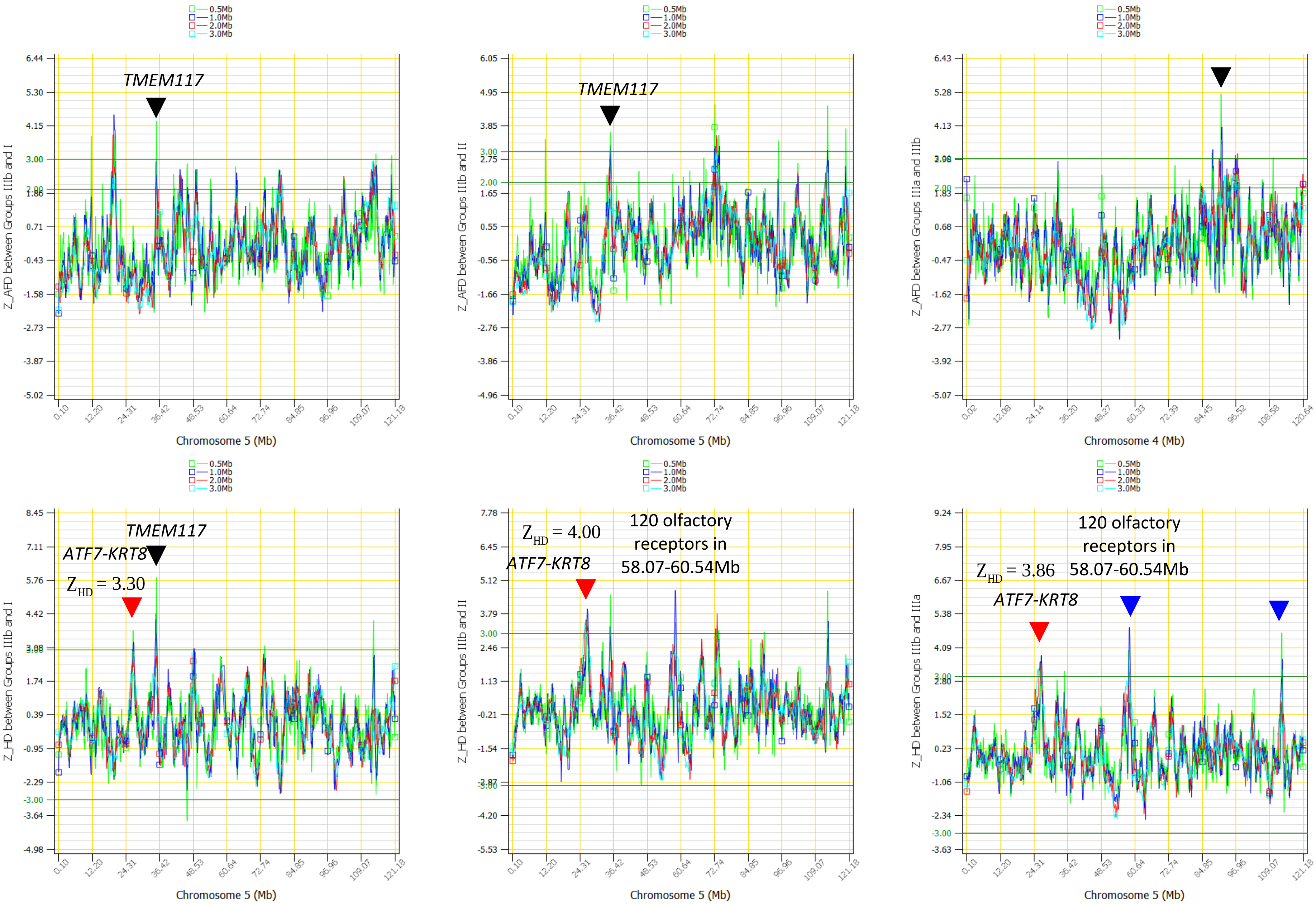
0.5Mb
1.0Mb
2.0Mb
3.0Mb
TMEM117
Z_AFD between Groups IIIb and I
Chromosome 5 (Mb)
TMEM117
Z_AFD between Groups IIIb and II
Chromosome 5 (Mb)
Z_AFD between Groups IIIa and IIIb
Chromosome 4 (Mb)
TMEM117
ATF7-KRT8
Z_{HD} = 3.30
Z_HD between Groups IIIb and I
Chromosome 5 (Mb)
Z_{HD} = 4.00
120 olfactory receptors in 58.07-60.54Mb
ATF7-KRT8
Z_HD between Groups IIIb and II
Chromosome 5 (Mb)
120 olfactory receptors in
Z_{HD} = 3.86 58.07-60.54Mb
ATF7-KRT8
Z_HD between Groups IIIb and IIIa
Chromosome 5 (Mb)

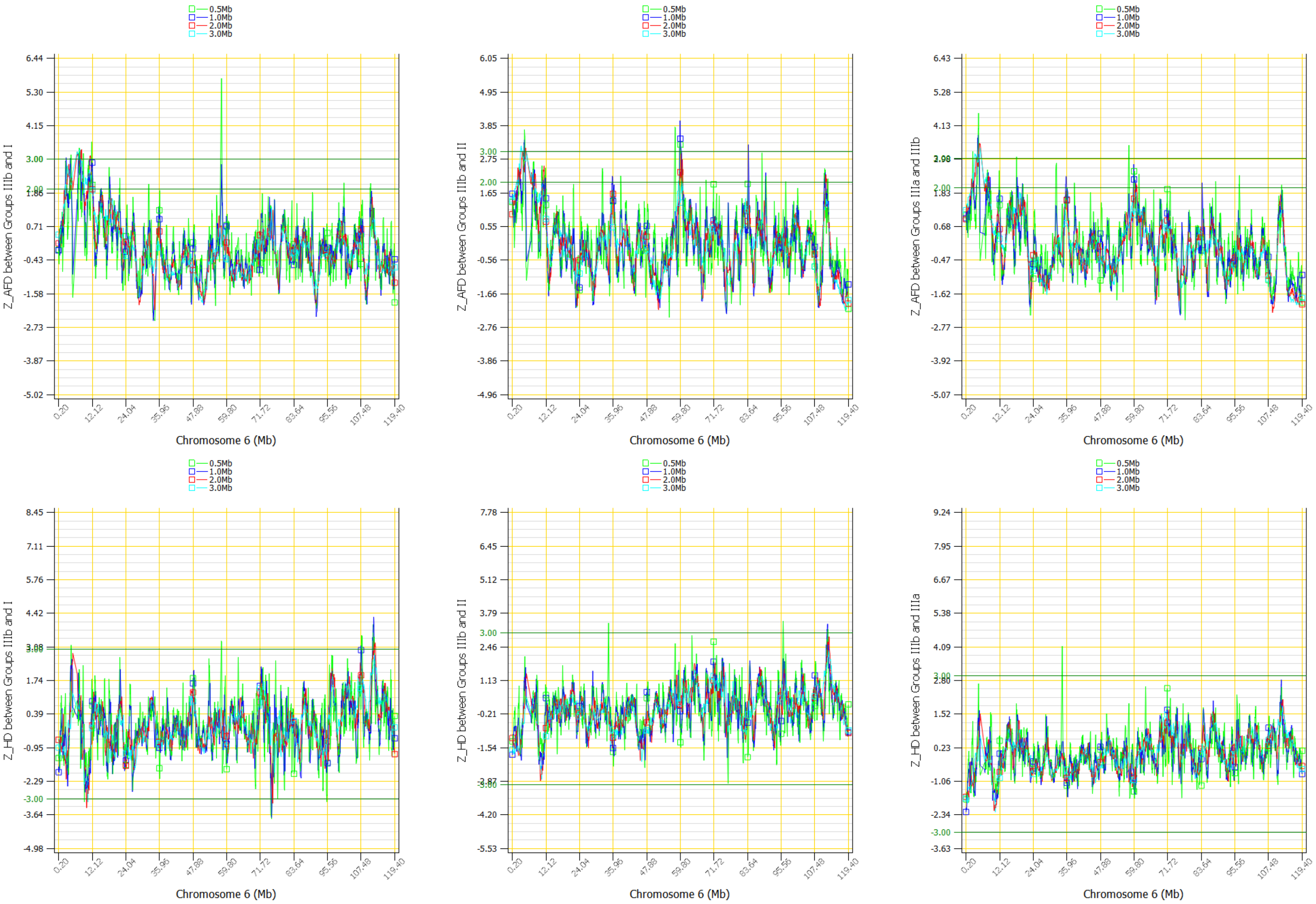

0.5Mb
1.0Mb
2.0Mb
3.0Mb
Z_AFD between Groups IIIb and I
Chromosome 6 (Mb)
Z_AFD between Groups IIIb and II
Chromosome 6 (Mb)
Z_AFD between Groups IIIa and IIIb
Chromosome 6 (Mb)
Z_HD between Groups IIIb and I
Chromosome 6 (Mb)
Z_HD between Groups IIIb and II
Chromosome 6 (Mb)
Z_HD between Groups IIIb and IIIa
Chromosome 6 (Mb)
0.20
12.12
24.04
35.96
47.88
59.80
71.72
83.64
95.56
107.48
119.40

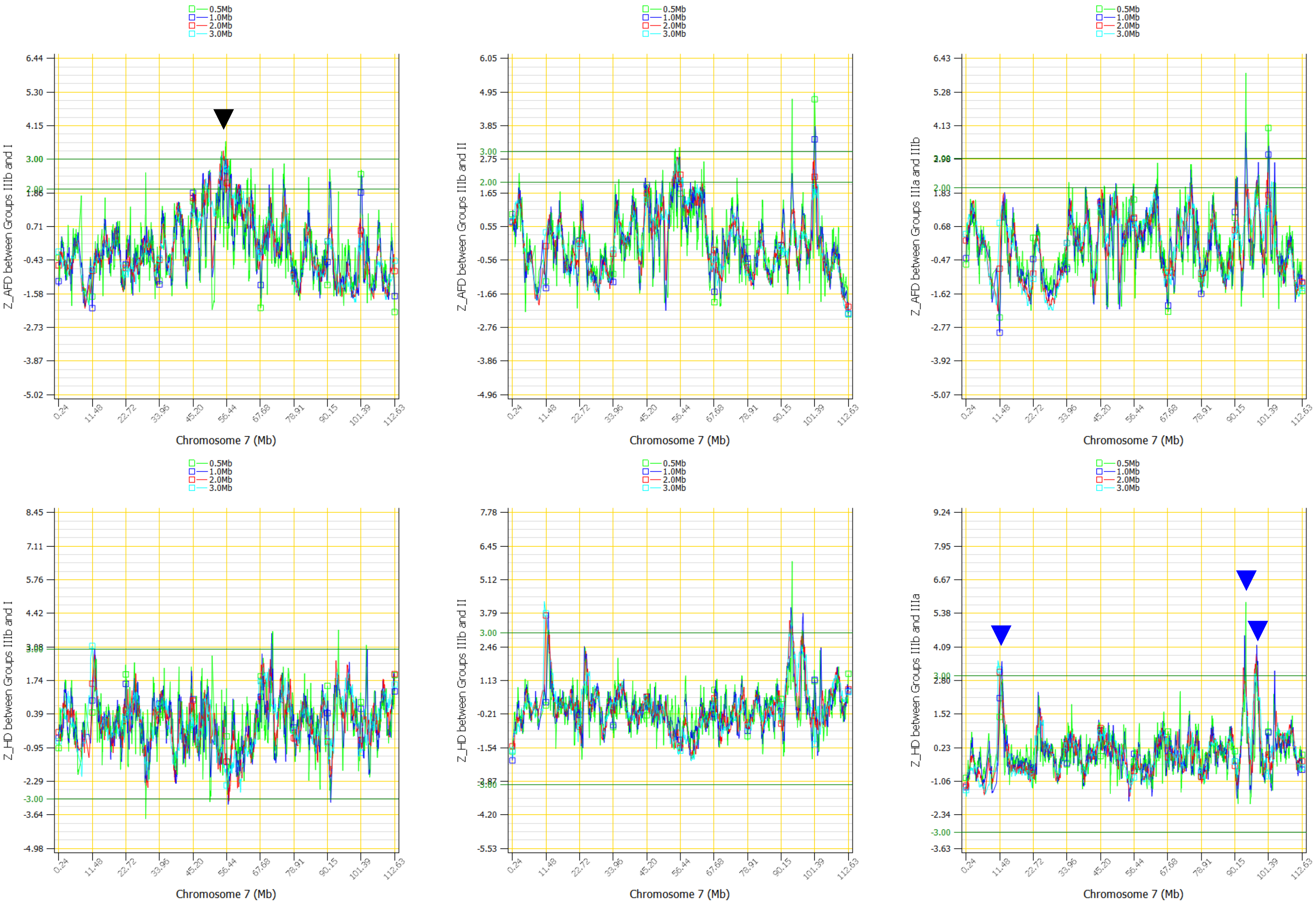

0.5Mb
1.0Mb
2.0Mb
3.0Mb
Z_AFD between Groups IIIb and I
Z_AFD between Groups IIIb and II
Z_AFD between Groups IIIa and IIIb
Z_HD between Groups IIIb and I
Z_HD between Groups IIIb and II
Z_HD between Groups IIIb and IIIa
0.24 11.48 22.72 33.96 45.20 56.44 67.68 78.91 90.15 101.39 112.63
Chromosome 7 (Mb)

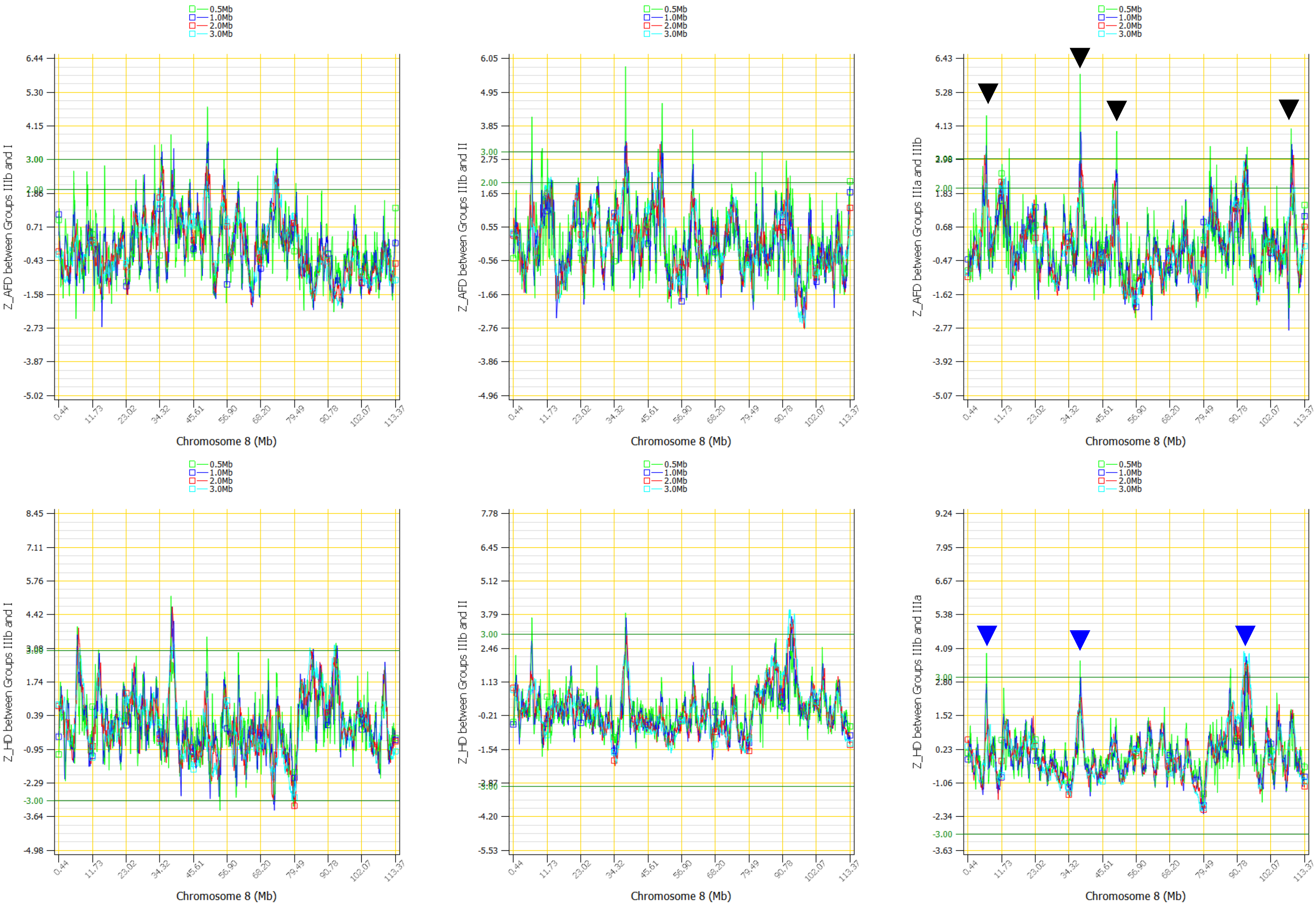

0.5Mb
1.0Mb
2.0Mb
3.0Mb
Z_AFD between Groups IIIb and I
Z_AFD between Groups IIIb and II
Z_AFD between Groups IIIa and IIIb
Z_HD between Groups IIIb and I
Z_HD between Groups IIIb and II
Z_HD between Groups IIIb and IIIa
0.44
11.73
23.02
34.32
45.61
56.90
68.20
79.49
90.78
102.07
113.37
Chromosome 8 (Mb)

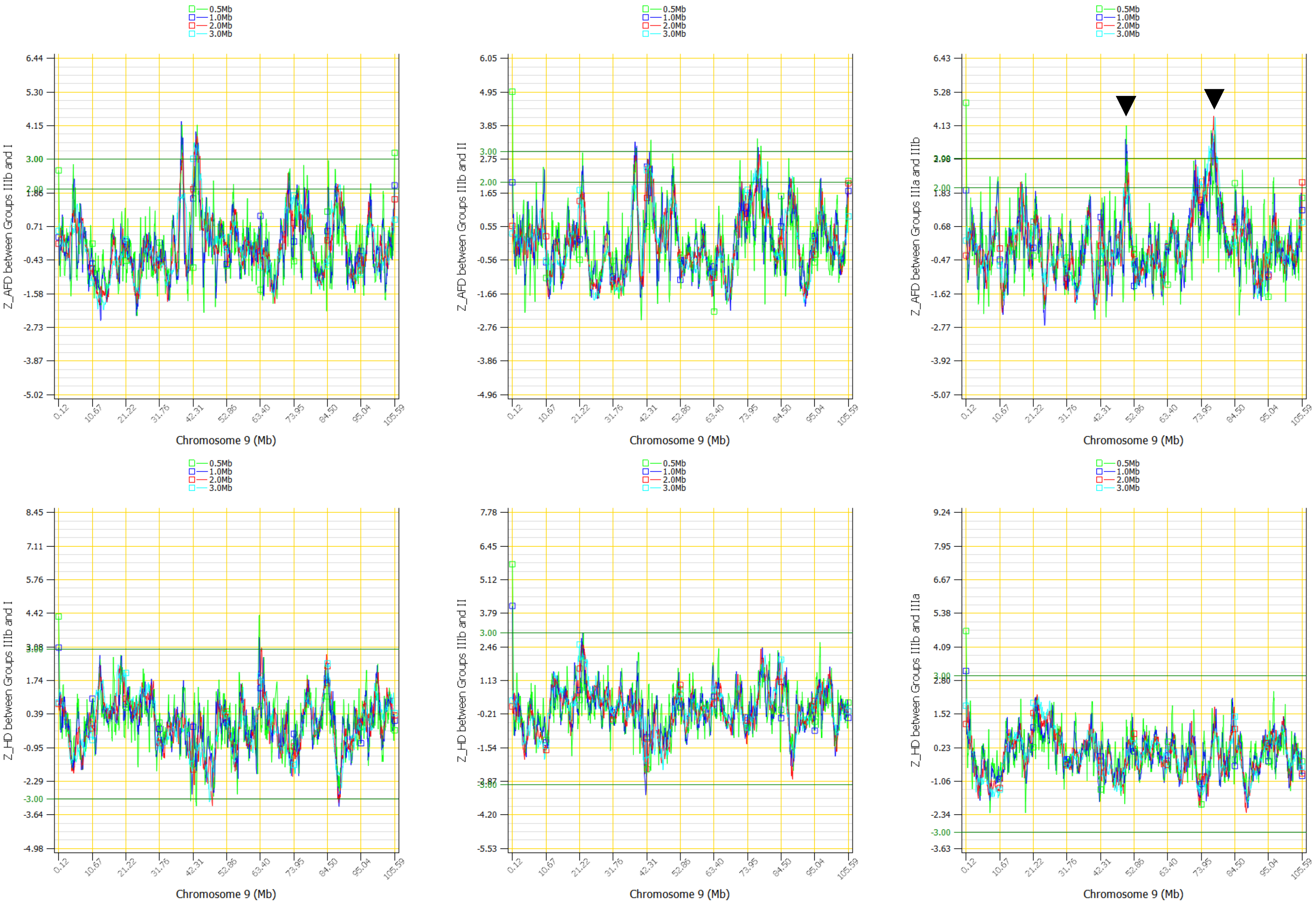

0.5Mb
1.0Mb
2.0Mb
3.0Mb
Z_AFD between Groups IIIb and I
Z_AFD between Groups IIIb and II
Z_AFD between Groups IIIa and IIIb
Z_HD between Groups IIIb and I
Z_HD between Groups IIIb and II
Z_HD between Groups IIIb and IIIa
0.12
10.67
21.22
31.76
42.31
52.86
63.40
73.95
84.50
95.04
105.59
Chromosome 9 (Mb)

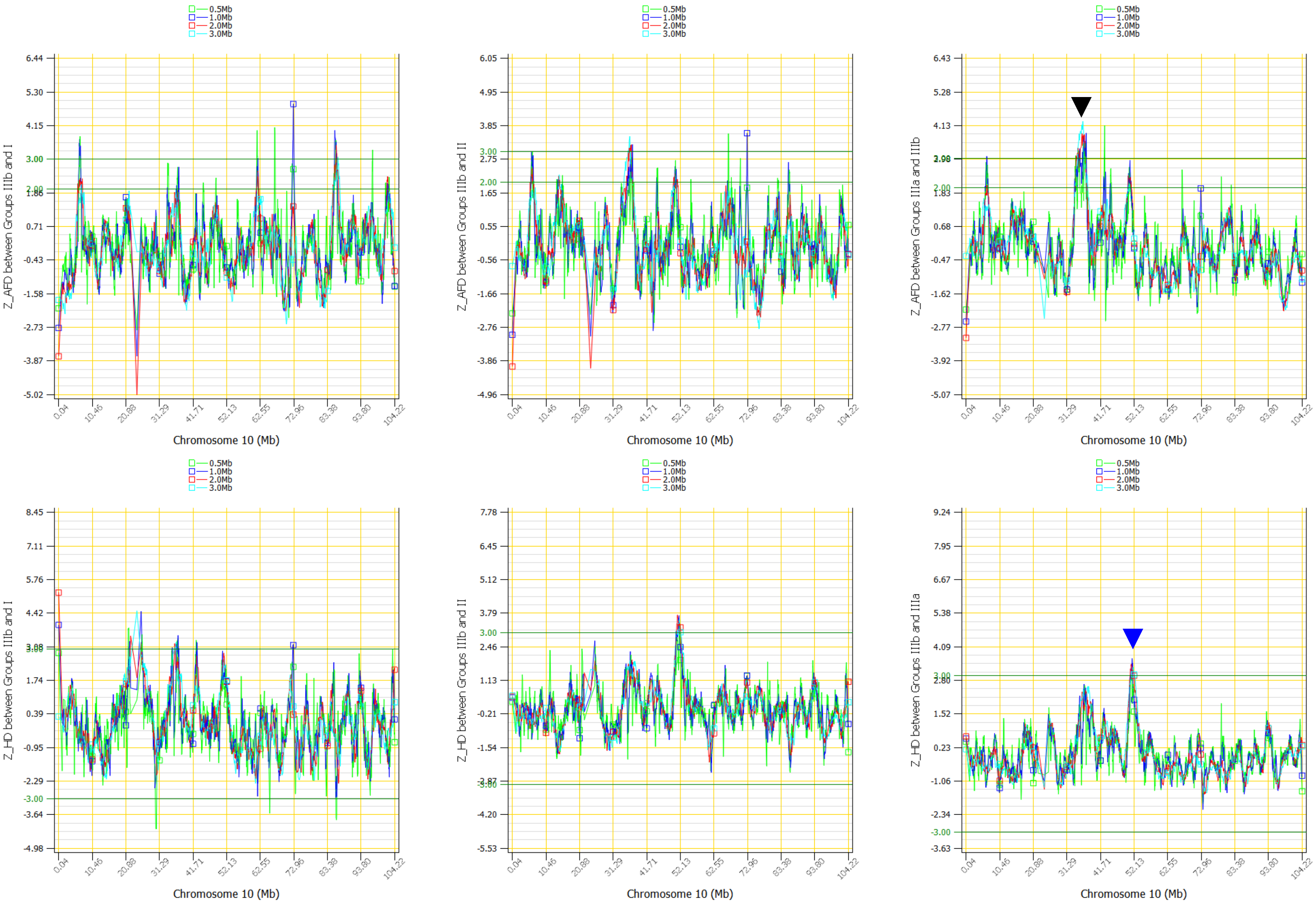

0.5Mb
1.0Mb
2.0Mb
3.0Mb
Z_AFD between Groups IIIb and I
6.44 5.30 4.15 3.00 2.00 1.86 0.71 -0.43 -1.58 -2.73 -3.87 -5.02
Z_AFD between Groups IIIb and II
6.05 4.95 3.85 3.00 2.75 2.00 1.65 0.55 -0.56 -1.66 -2.76 -3.86 -4.96
Z_AFD between Groups IIIa and IIIb
6.43 5.28 4.13 1.83 0.68 -0.47 -1.62 -2.77 -3.92 -5.07
Z_HD between Groups IIIb and I
8.45 7.11 5.76 4.42 3.08 3.00 1.74 0.39 -0.95 -2.29 -3.00 -3.64 -4.98
Z_HD between Groups IIIb and II
7.78 6.45 5.12 3.79 3.00 2.46 1.13 -0.21 -1.54 -2.87 -3.00 -4.20 -5.53
Z_HD between Groups IIIb and IIIa
9.24 7.95 6.67 5.38 4.09 3.00 2.80 1.52 0.23 -1.06 -2.34 -3.00 -3.63
0.04 10.46 20.88 31.29 41.71 52.13 62.55 72.96 83.38 93.80 104.22
Chromosome 10 (Mb)

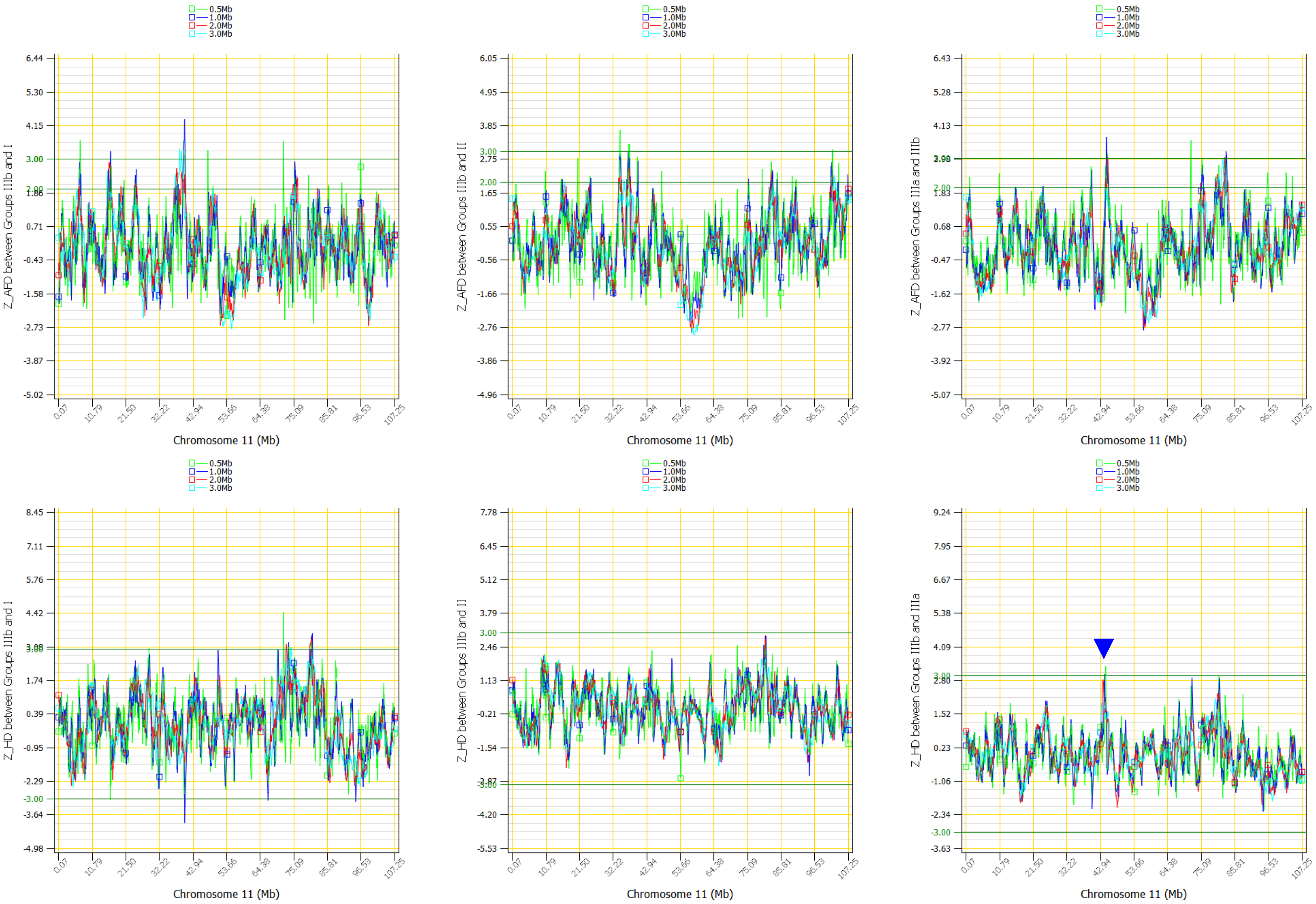
0.5Mb
1.0Mb
2.0Mb
3.0Mb
Z_AFD between Groups IIIb and I
Chromosome 11 (Mb)
Z_AFD between Groups IIIb and II
Chromosome 11 (Mb)
Z_AFD between Groups IIIa and IIIb
Chromosome 11 (Mb)
Z_HD between Groups IIIb and I
Chromosome 11 (Mb)
Z_HD between Groups IIIb and II
Chromosome 11 (Mb)
Z_HD between Groups IIIb and IIIa
Chromosome 11 (Mb)

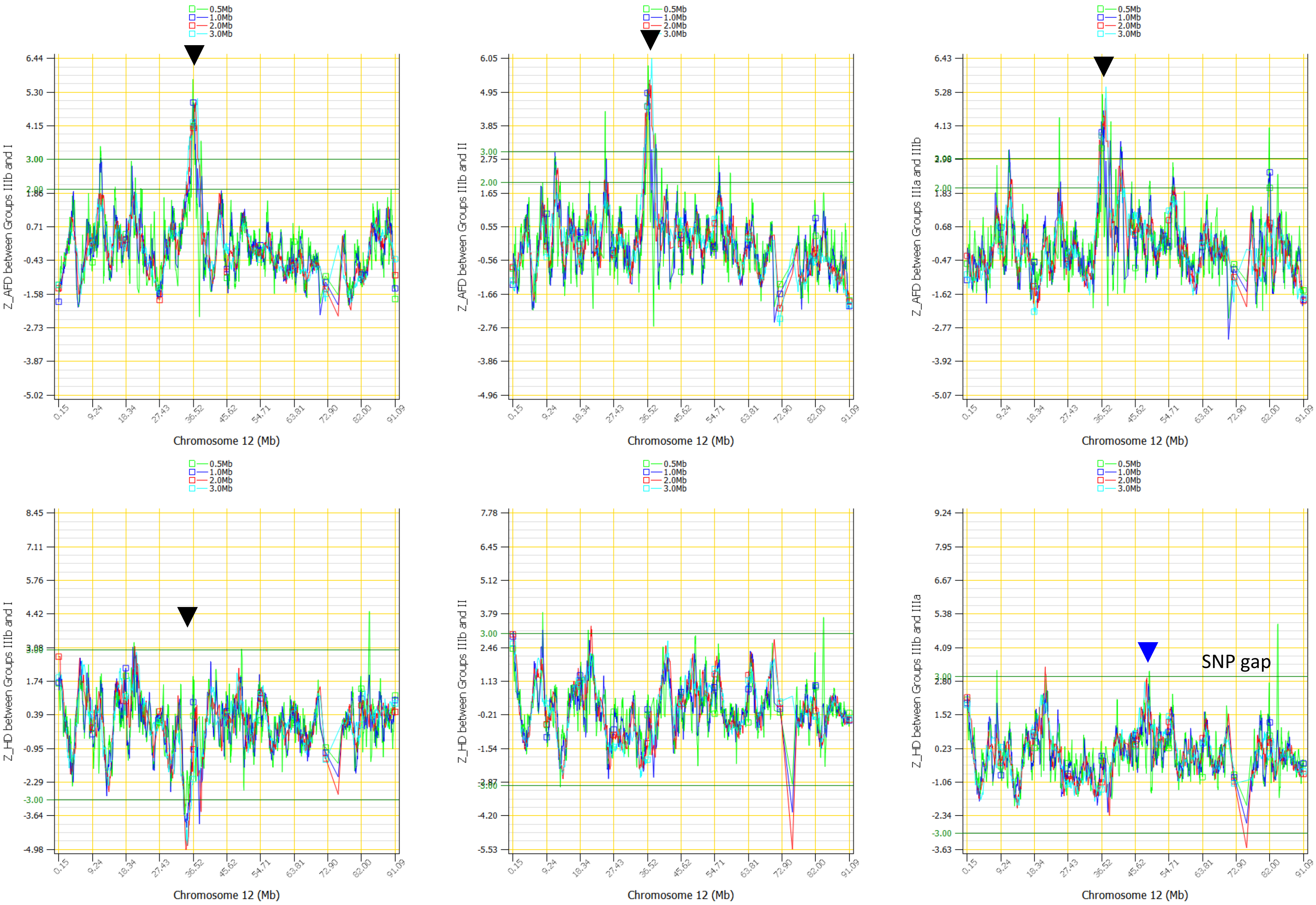
0.5Mb
1.0Mb
2.0Mb
3.0Mb
Z_AFD between Groups IIIb and I
Z_AFD between Groups IIIb and II
Z_AFD between Groups IIIa and IIIb
Z_HD between Groups IIIb and I
Z_HD between Groups IIIb and II
Z_HD between Groups IIIb and IIIa
Chromosome 12 (Mb)
SNP gap

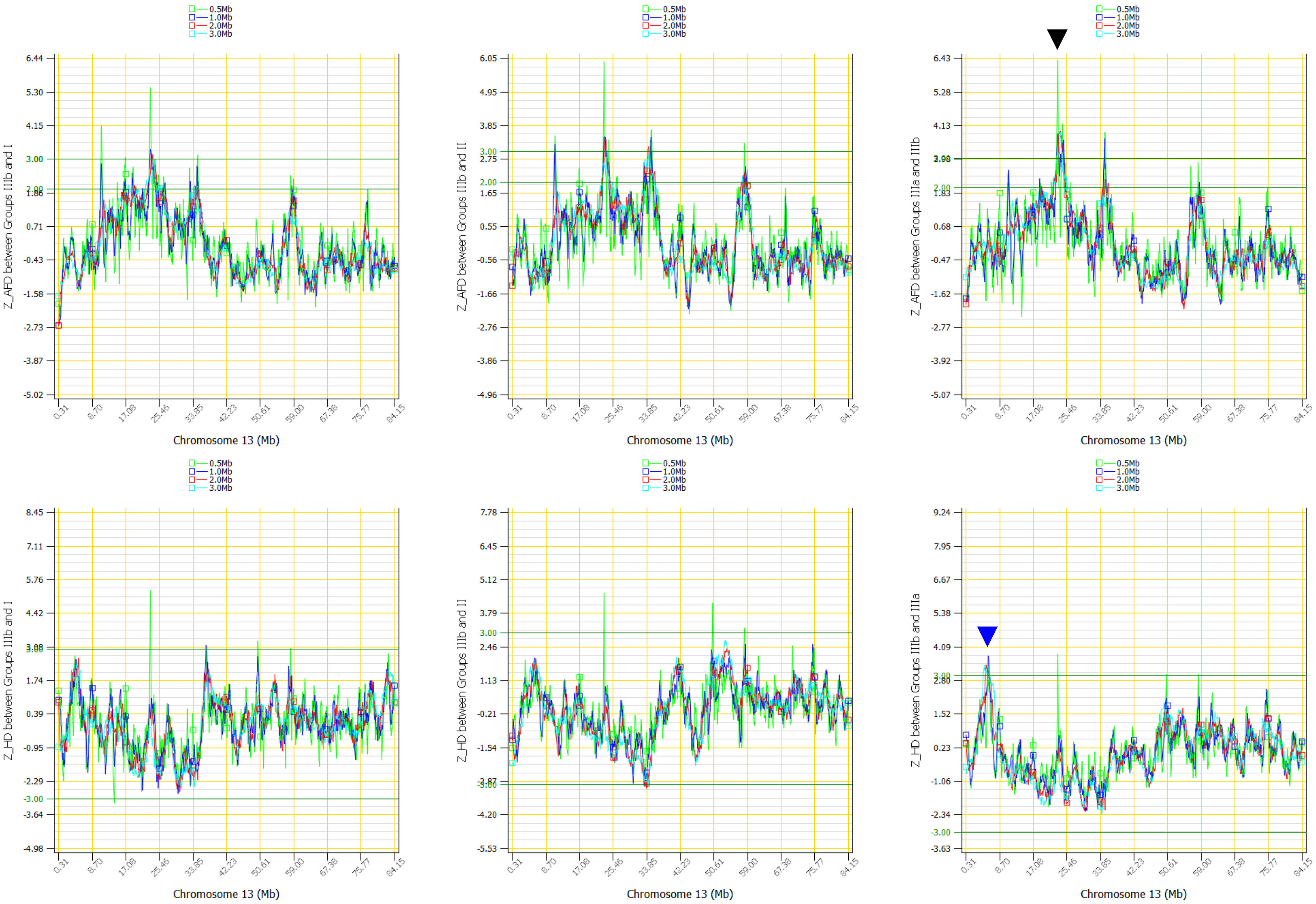
0.5Mb
1.0Mb
2.0Mb
3.0Mb
Z_AFD between Groups IIIb and I
Chromosome 13 (Mb)
Z_AFD between Groups IIIb and II
Chromosome 13 (Mb)
Z_AFD between Groups IIIa and IIIb
Chromosome 13 (Mb)
Z_HD between Groups IIIb and I
Chromosome 13 (Mb)
Z_HD between Groups IIIb and II
Chromosome 13 (Mb)
Z_HD between Groups IIIb and IIIa
Chromosome 13 (Mb)

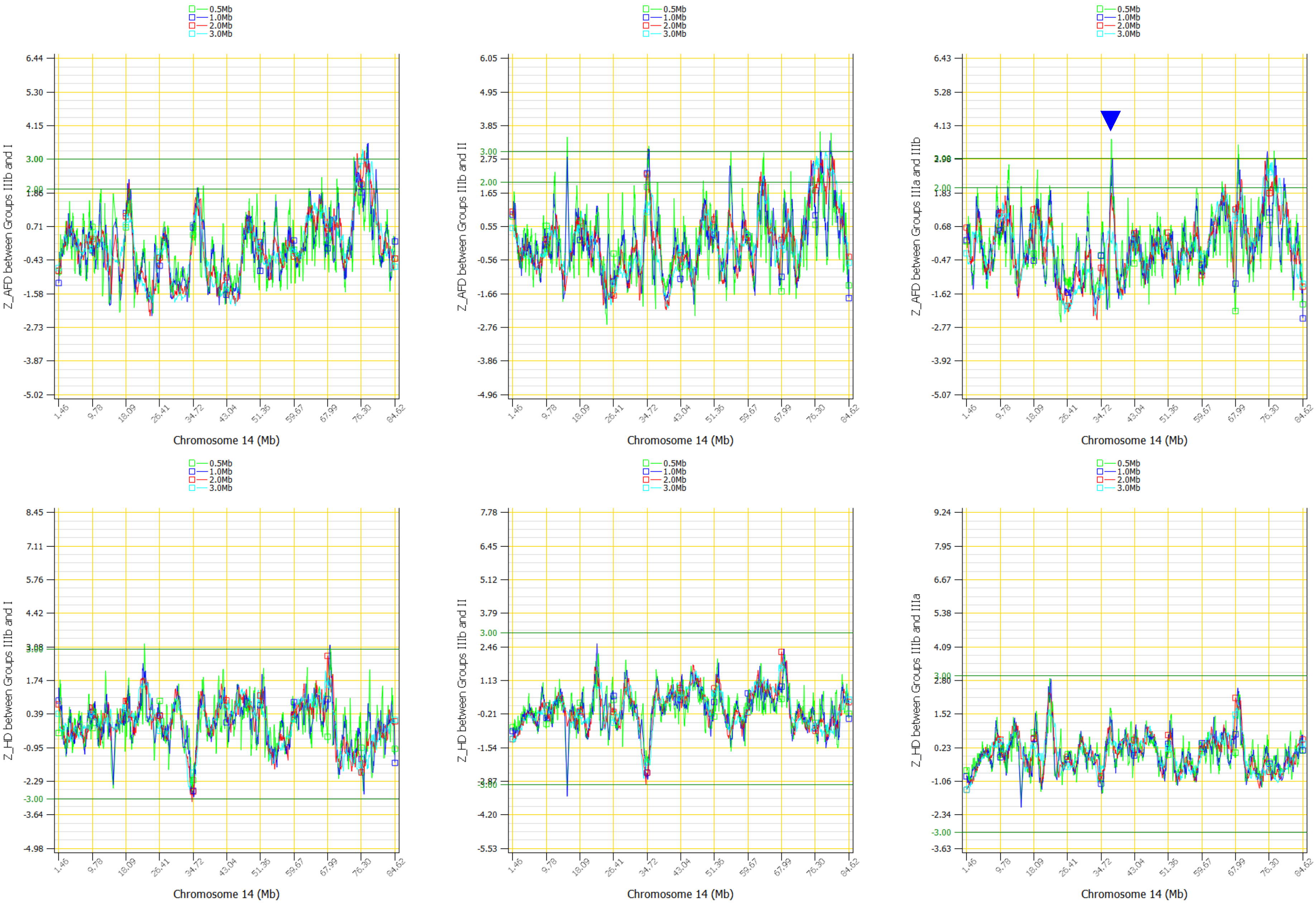
0.5Mb
1.0Mb
2.0Mb
3.0Mb
Z_AFD between Groups IIIb and I
Z_AFD between Groups IIIb and II
Z_AFD between Groups IIIa and IIIb
Z_HD between Groups IIIb and I
Z_HD between Groups IIIb and II
Z_HD between Groups IIIb and IIIa
Chromosome 14 (Mb)
1.46
9.78
18.09
26.41
34.72
43.04
51.36
59.67
67.99
76.30
84.62

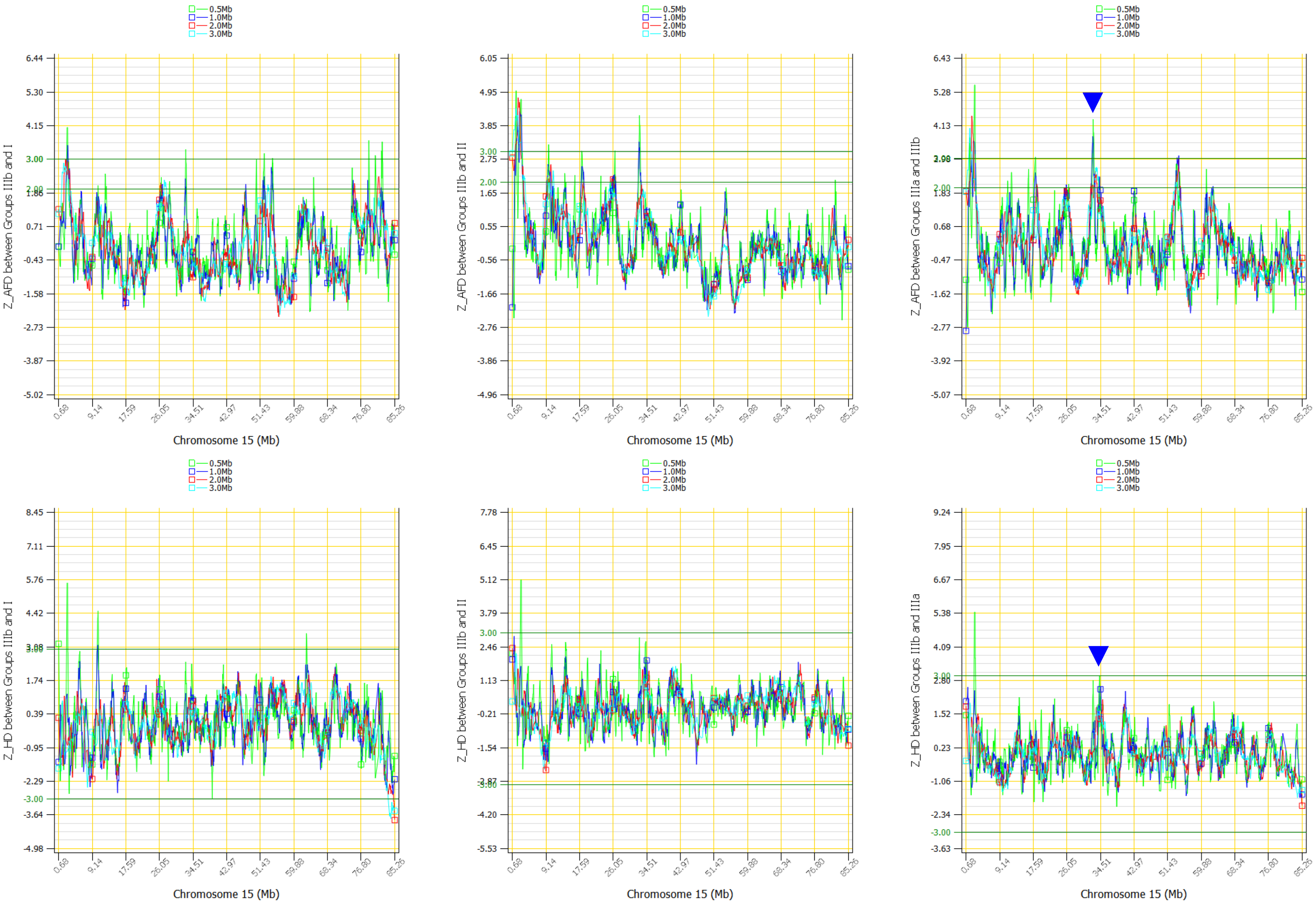

0.5Mb
1.0Mb
2.0Mb
3.0Mb
Z_AFD between Groups IIIb and I
Z_AFD between Groups IIIb and II
Z_AFD between Groups IIIa and IIIb
Z_HD between Groups IIIb and I
Z_HD between Groups IIIb and II
Z_HD between Groups IIIb and IIIa
Chromosome 15 (Mb)
0.68
9.14
17.59
26.05
34.51
42.97
51.43
59.88
68.34
76.80
85.26

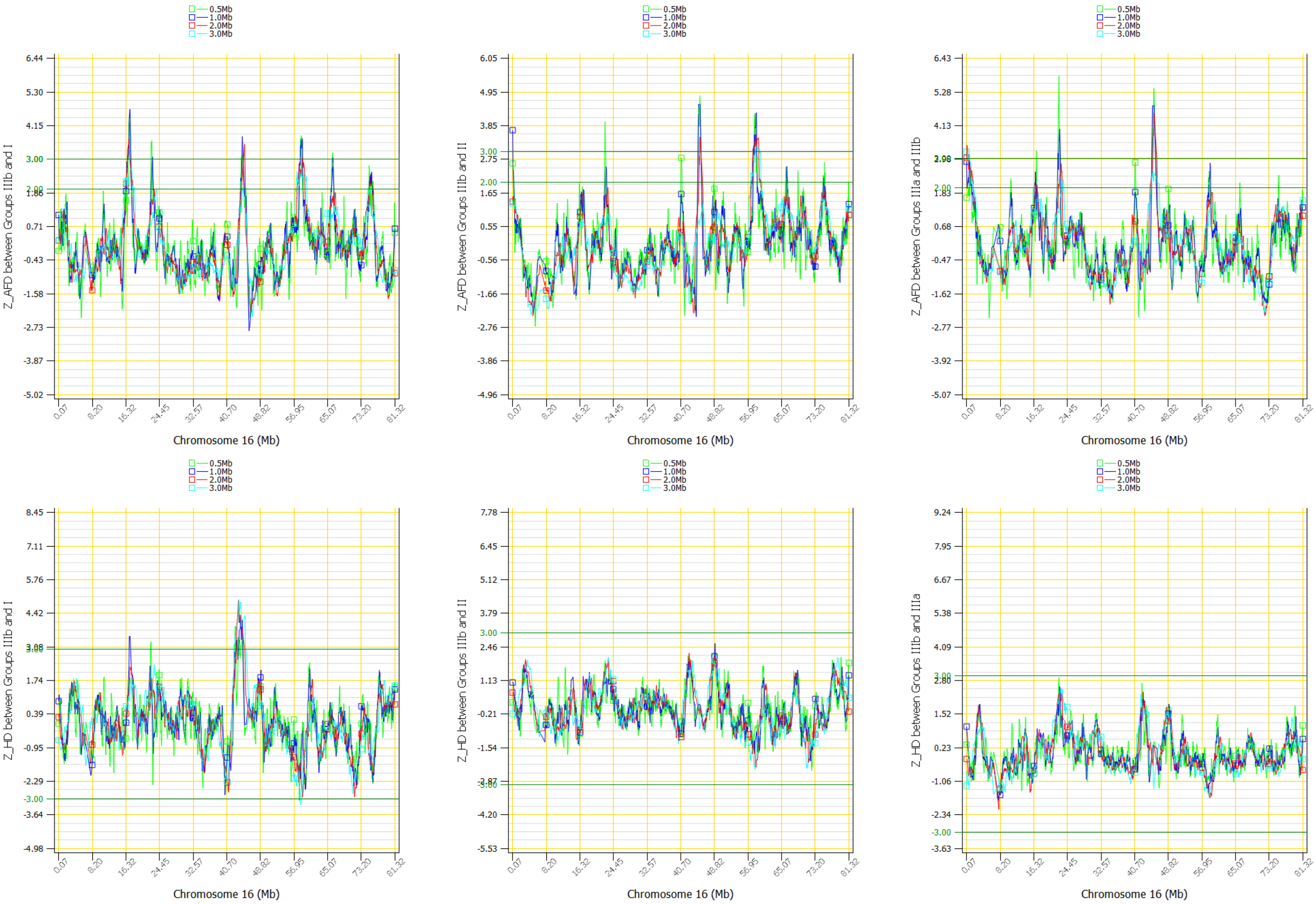
0.5Mb
1.0Mb
2.0Mb
3.0Mb
Z_AFD between Groups IIIb and I
Z_AFD between Groups IIIb and II
Z_AFD between Groups IIIa and IIIb
Z_HD between Groups IIIb and I
Z_HD between Groups IIIb and II
Z_HD between Groups IIIb and IIIa
Chromosome 16 (Mb)
0.07
8.20
16.32
24.45
32.57
40.70
48.82
56.95
65.07
73.20
81.32

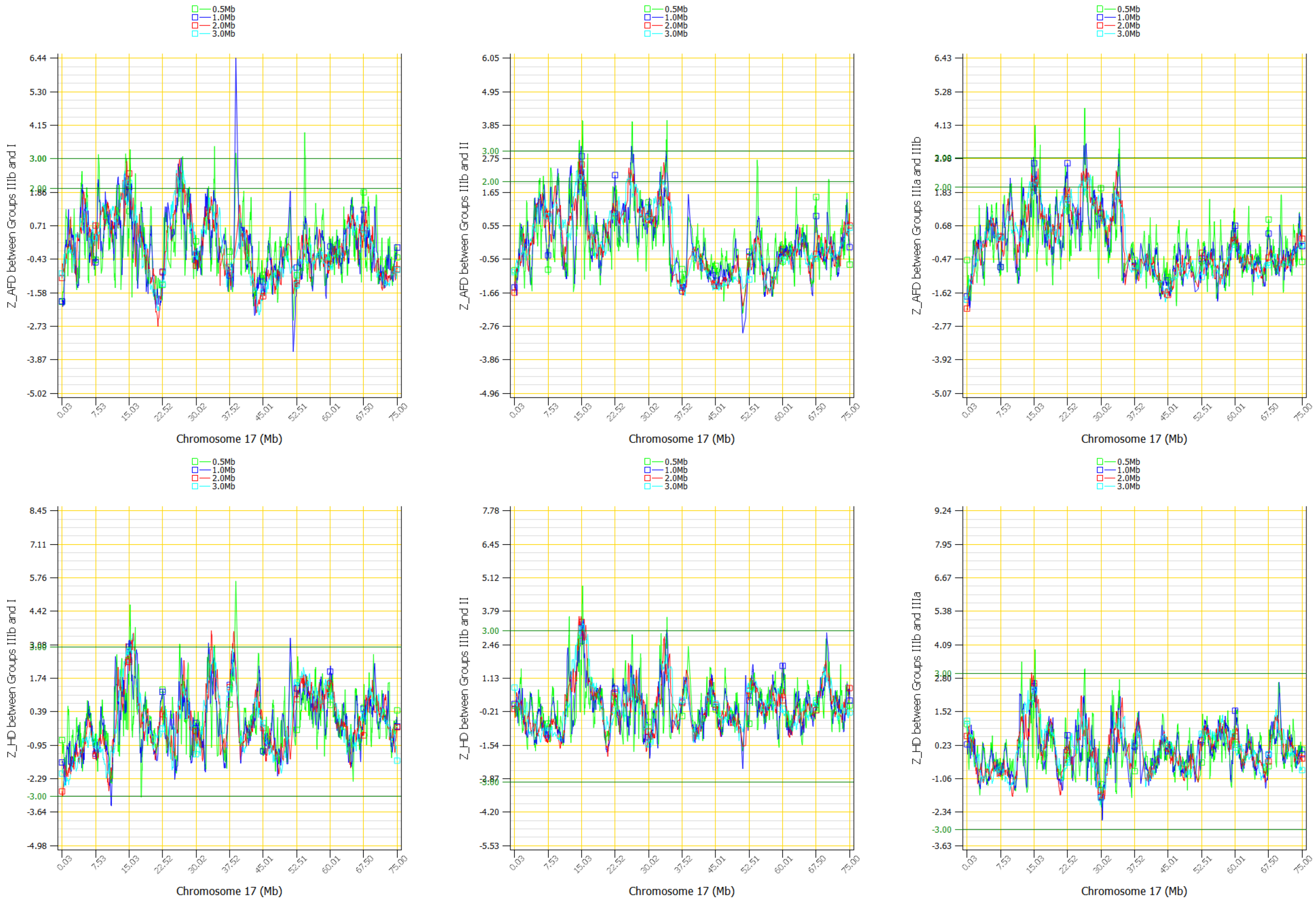
0.5Mb
1.0Mb
2.0Mb
3.0Mb
Z_AFD between Groups IIIb and I
Chromosome 17 (Mb)
Z_AFD between Groups IIIb and II
Chromosome 17 (Mb)
Z_AFD between Groups IIIa and IIIb
Chromosome 17 (Mb)
Z_HD between Groups IIIb and I
Chromosome 17 (Mb)
Z_HD between Groups IIIb and II
Chromosome 17 (Mb)
Z_HD between Groups IIIb and IIIa
Chromosome 17 (Mb)

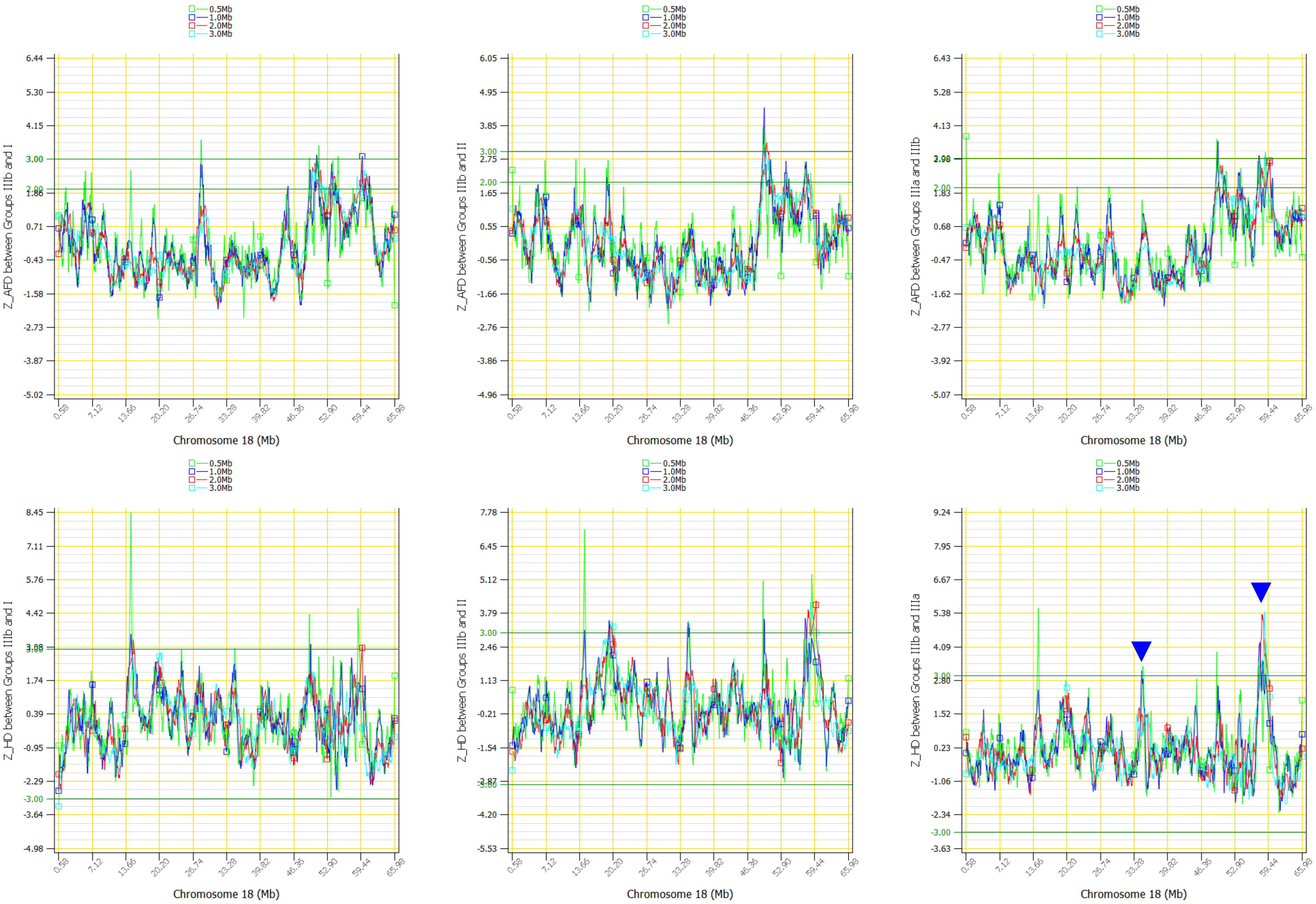
0.5Mb
1.0Mb
2.0Mb
3.0Mb
Z_AFD between Groups IIIb and I
Chromosome 18 (Mb)
Z_AFD between Groups IIIb and II
Chromosome 18 (Mb)
Z_AFD between Groups IIIa and IIIb
Chromosome 18 (Mb)
Z_HD between Groups IIIb and I
Chromosome 18 (Mb)
Z_HD between Groups IIIb and II
Chromosome 18 (Mb)
Z_HD between Groups IIIb and IIIa
Chromosome 18 (Mb)
0.58
7.12
13.66
20.20
26.74
33.28
39.82
46.36
52.90
59.44
65.98

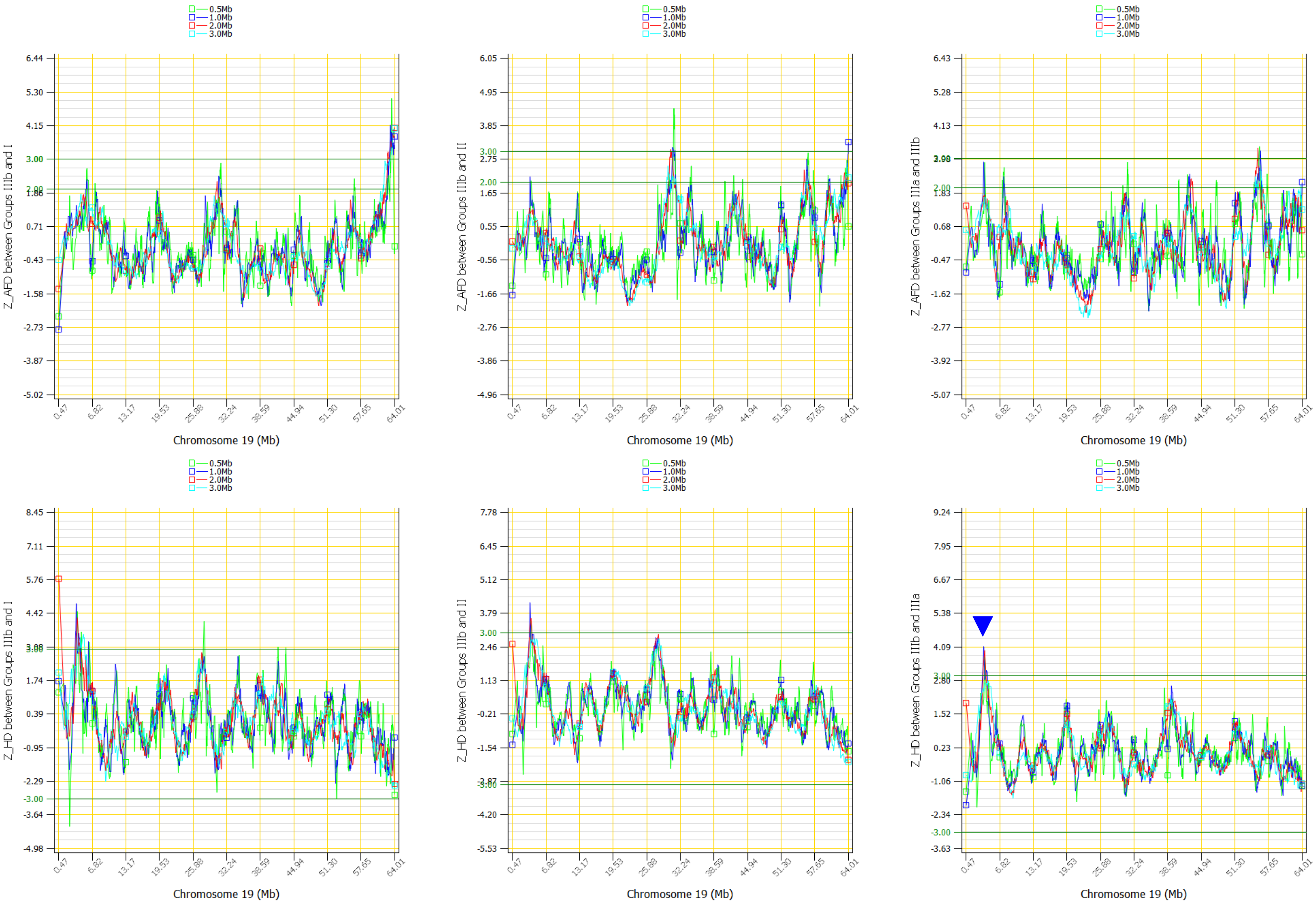
0.5Mb
1.0Mb
2.0Mb
3.0Mb
Z_AFD between Groups IIIb and I
Chromosome 19 (Mb)
Z_AFD between Groups IIIb and II
Chromosome 19 (Mb)
Z_AFD between Groups IIIa and IIIb
Chromosome 19 (Mb)
Z_HD between Groups IIIb and I
Chromosome 19 (Mb)
Z_HD between Groups IIIb and II
Chromosome 19 (Mb)
Z_HD between Groups IIIb and IIIa
Chromosome 19 (Mb)
0.47
6.82
13.17
19.53
25.88
32.24
38.59
44.94
51.30
57.65
64.01

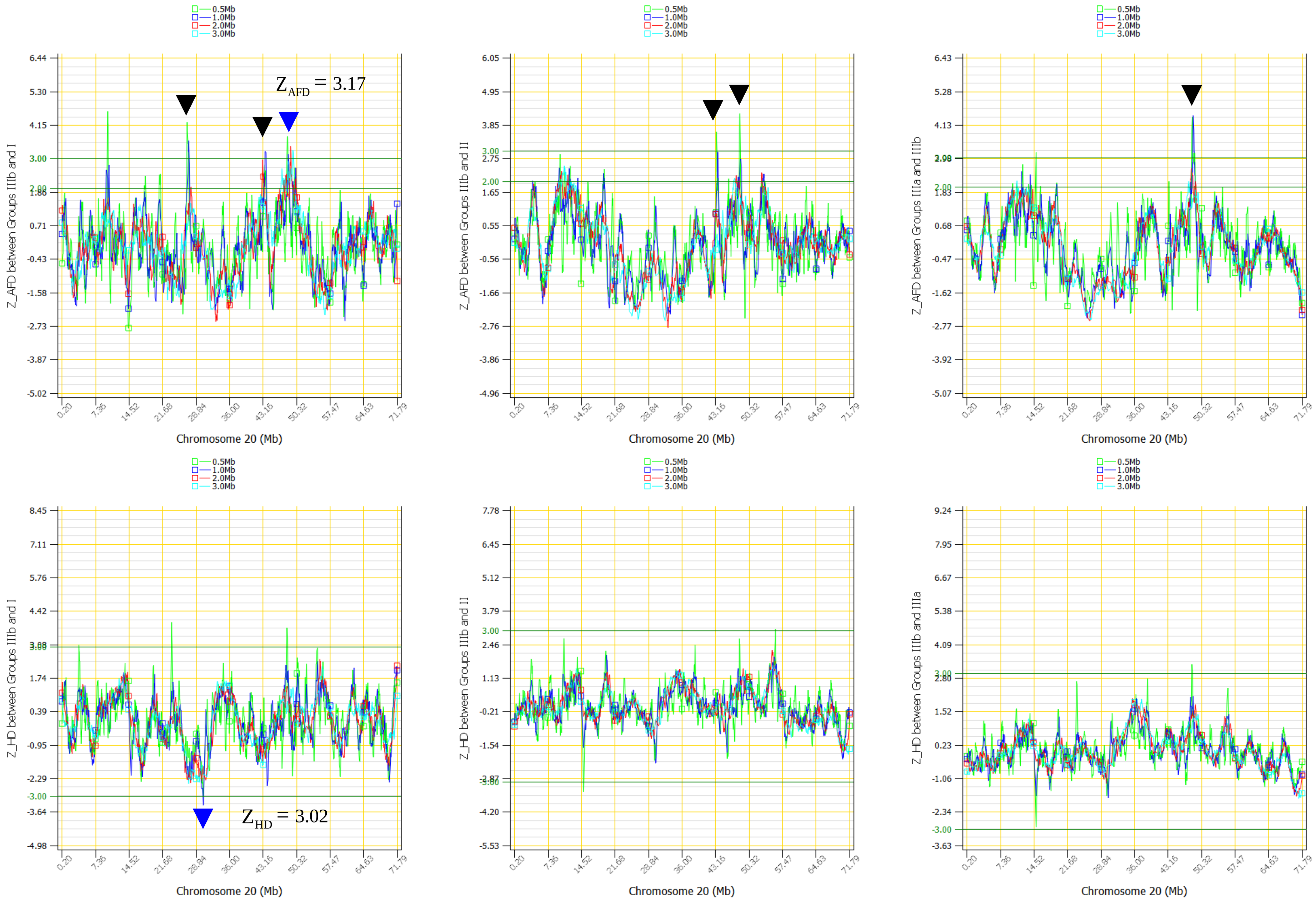
0.5Mb
1.0Mb
2.0Mb
3.0Mb
Z_AFD between Groups IIIb and I
Z_AFD between Groups IIIb and II
Z_AFD between Groups IIIa and IIIb
Z_HD between Groups IIIb and I
Z_HD between Groups IIIb and II
Z_HD between Groups IIIb and IIIa
Chromosome 20 (Mb)
Z_{AFD} = 3.17
Z_{HD} = 3.02

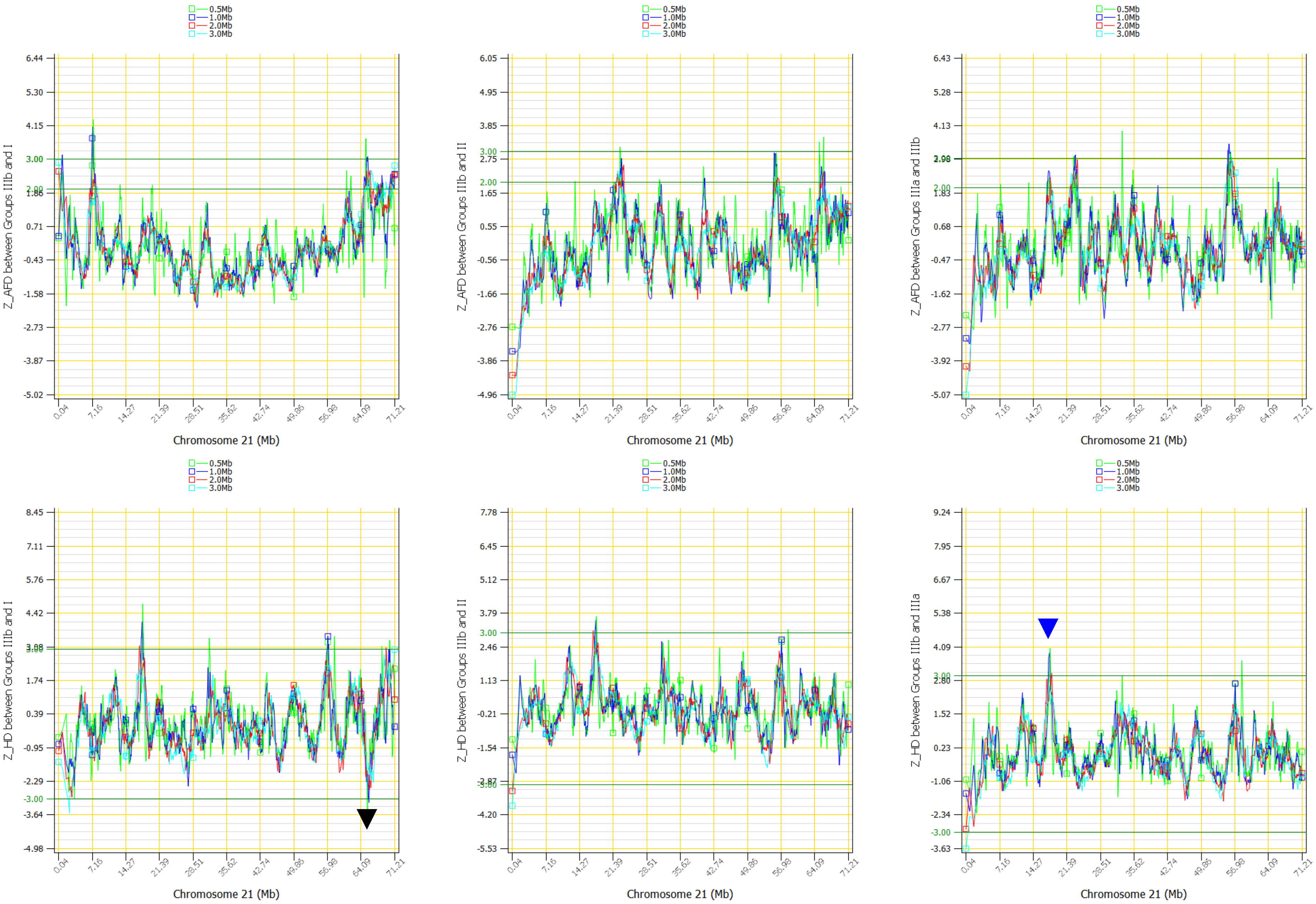
0.5Mb
1.0Mb
2.0Mb
3.0Mb
Z_AFD between Groups IIIb and I
Chromosome 21 (Mb)
Z_AFD between Groups IIIb and II
Chromosome 21 (Mb)
Z_AFD between Groups IIIa and IIIb
Chromosome 21 (Mb)
Z_HD between Groups IIIb and I
Chromosome 21 (Mb)
Z_HD between Groups IIIb and II
Chromosome 21 (Mb)
Z_HD between Groups IIIb and IIIa
Chromosome 21 (Mb)

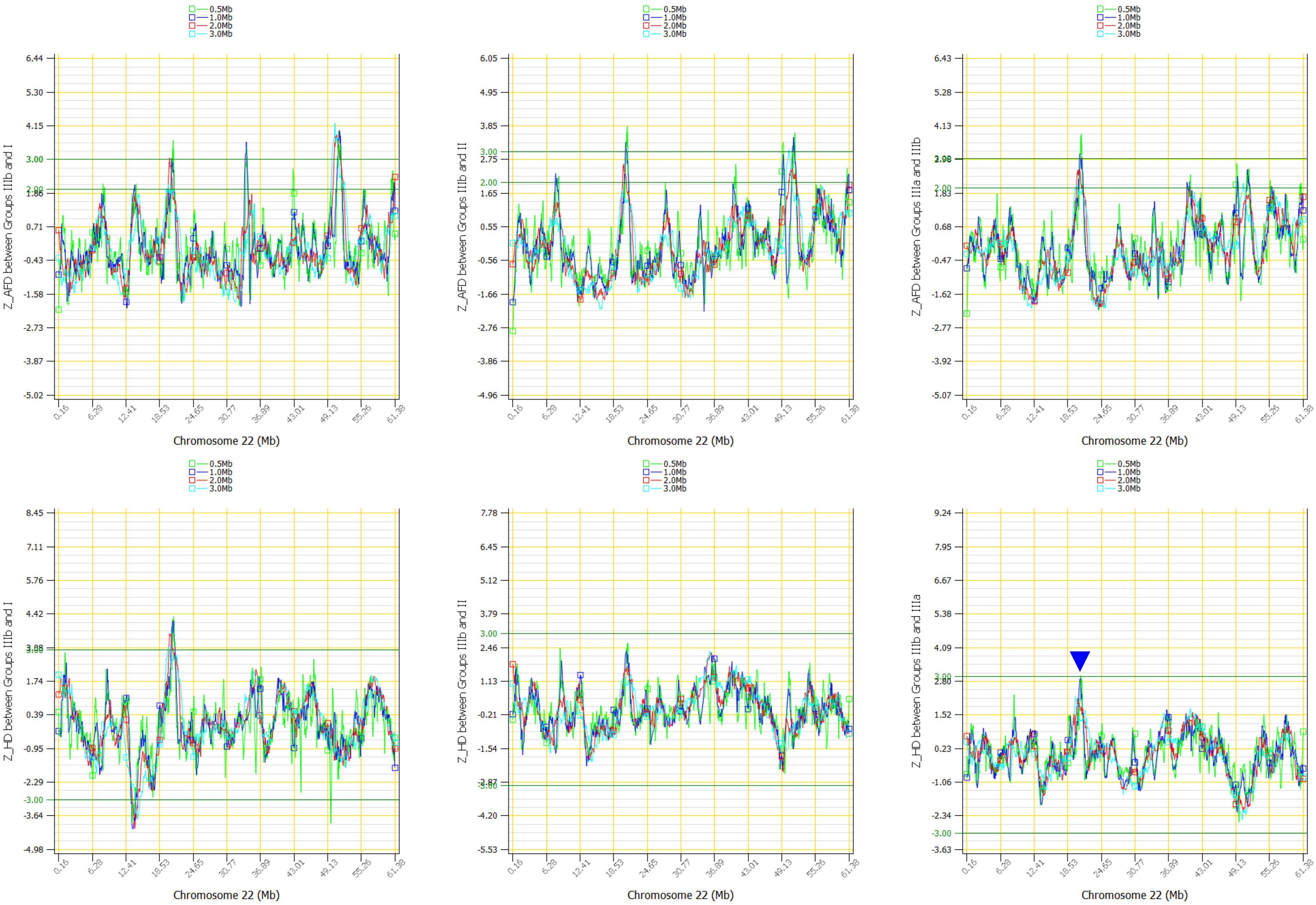

0.5Mb
1.0Mb
2.0Mb
3.0Mb
Z_AFD between Groups IIIb and I
Z_AFD between Groups IIIb and II
Z_AFD between Groups IIIa and IIIb
Z_HD between Groups IIIb and I
Z_HD between Groups IIIb and II
Z_HD between Groups IIIb and IIIa
0.16
6.28
12.41
18.53
24.65
30.77
36.89
43.01
49.13
55.26
61.38
Chromosome 22 (Mb)

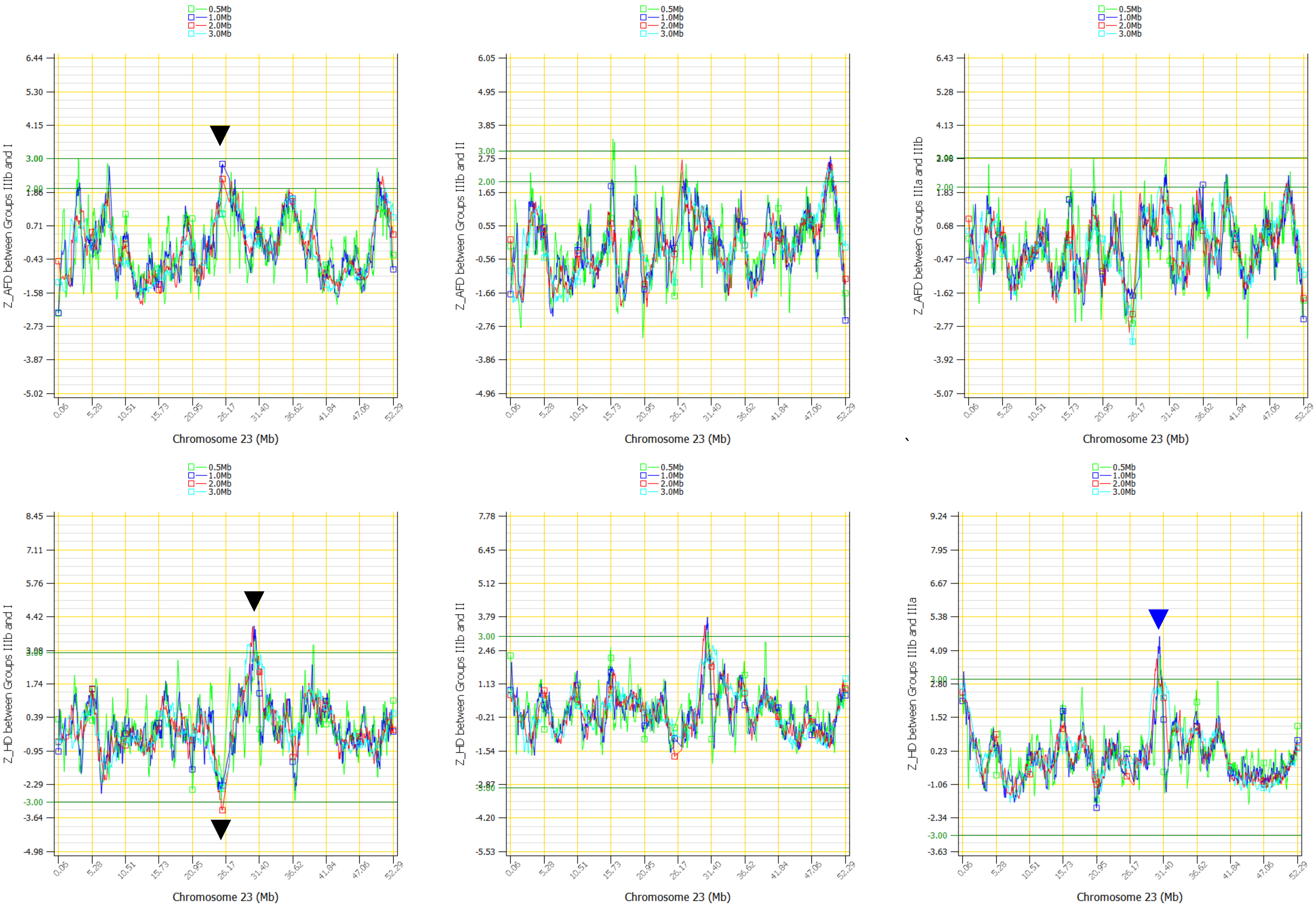
0.5Mb
1.0Mb
2.0Mb
3.0Mb
Z_AFD between Groups IIIb and I
Z_AFD between Groups IIIb and II
Z_AFD between Groups IIIa and IIIb
Z_HD between Groups IIIb and I
Z_HD between Groups IIIb and II
Z_HD between Groups IIIb and IIIa
Chromosome 23 (Mb)
0.06
5.28
10.51
15.73
20.95
26.17
31.40
36.62
41.84
47.06
52.29
3.00
2.00
-3.00

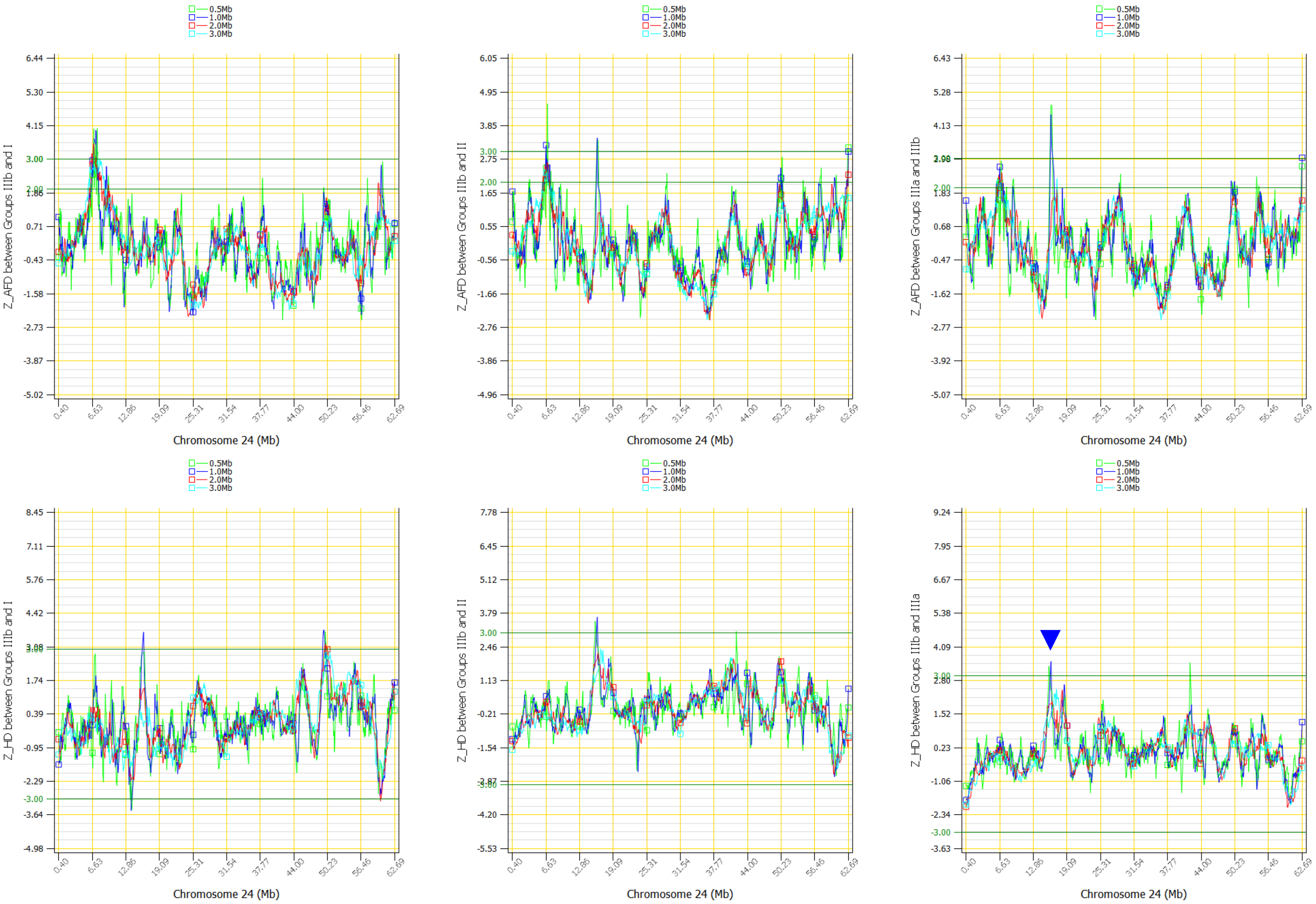
0.5Mb
1.0Mb
2.0Mb
3.0Mb
Z_AFD between Groups IIIb and I
Z_AFD between Groups IIIb and II
Z_AFD between Groups IIIa and IIIb
Z_HD between Groups IIIb and I
Z_HD between Groups IIIb and II
Z_HD between Groups IIIb and IIIa
Chromosome 24 (Mb)

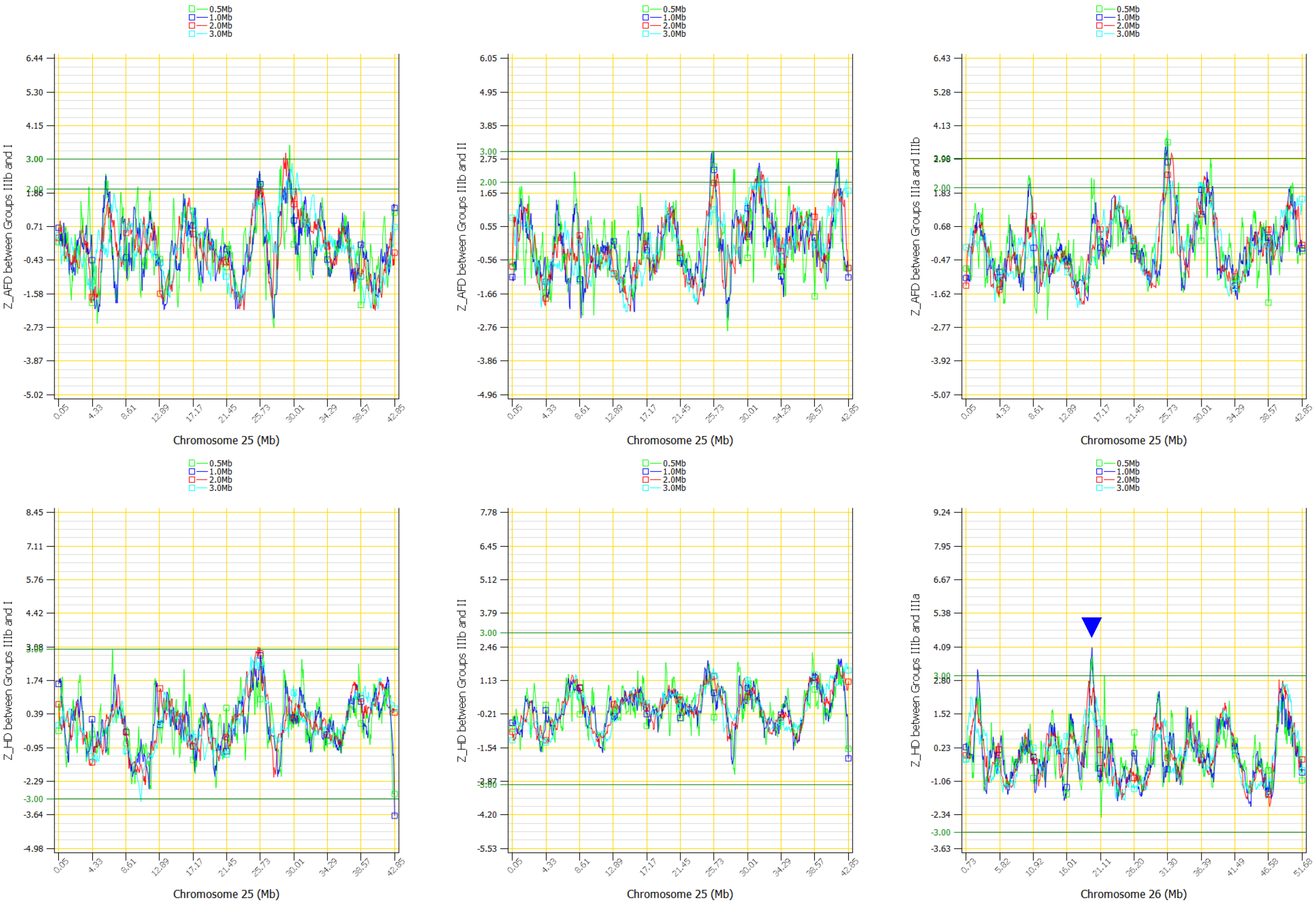

0.5Mb
1.0Mb
2.0Mb
3.0Mb
Z_AFD between Groups IIIb and I
Chromosome 25 (Mb)
Z_AFD between Groups IIIb and II
Chromosome 25 (Mb)
Z_AFD between Groups IIIa and IIIb
Chromosome 25 (Mb)
Z_HD between Groups IIIb and I
Chromosome 25 (Mb)
Z_HD between Groups IIIb and II
Chromosome 25 (Mb)
Z_HD between Groups IIIb and IIIa
Chromosome 26 (Mb)

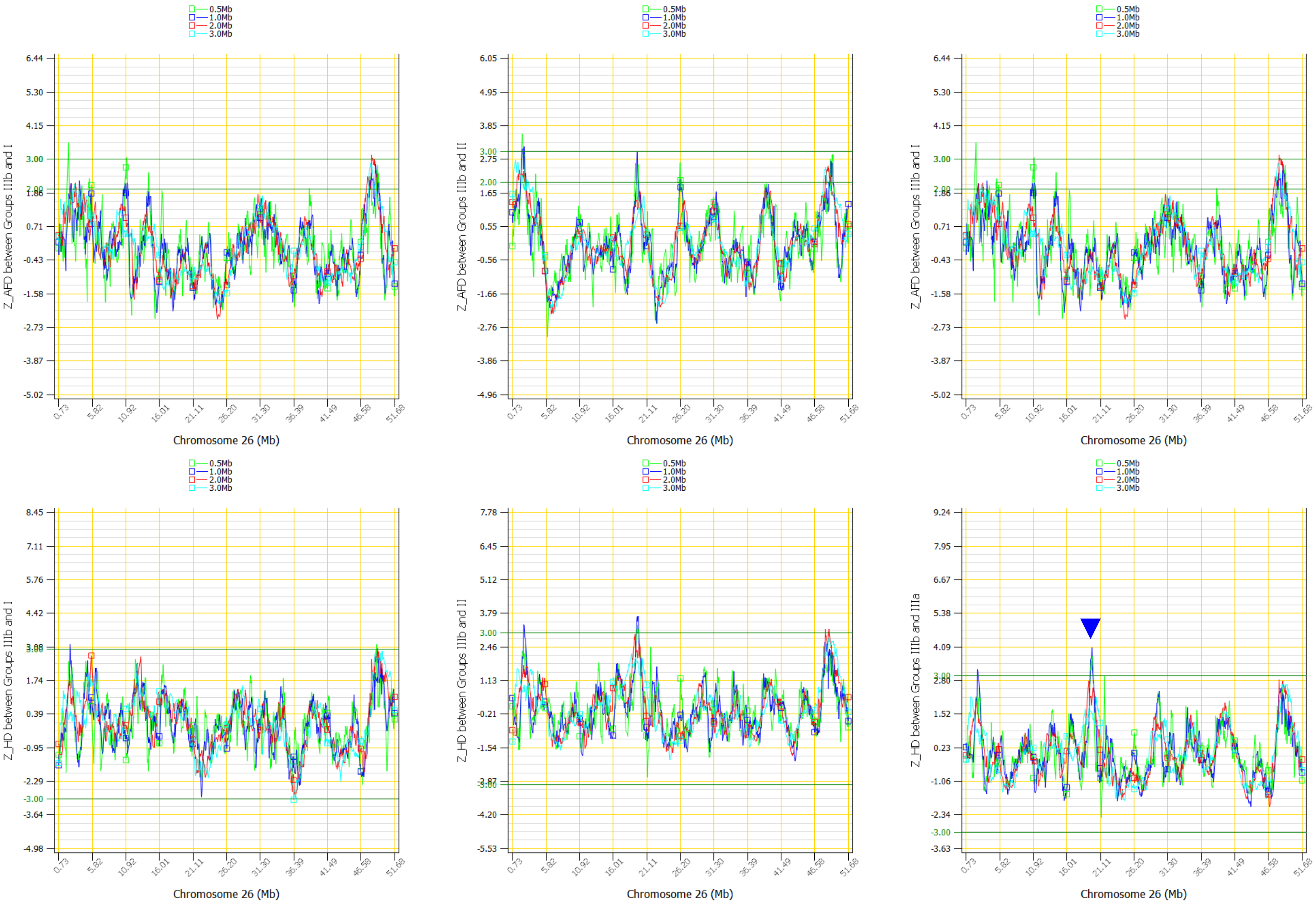

0.5Mb
1.0Mb
2.0Mb
3.0Mb
Z_AFD between Groups IIIb and I
Chromosome 26 (Mb)
Z_AFD between Groups IIIb and II
Chromosome 26 (Mb)
Z_AFD between Groups IIIb and I
Chromosome 26 (Mb)
Z_HD between Groups IIIb and I
Chromosome 26 (Mb)
Z_HD between Groups IIIb and II
Chromosome 26 (Mb)
Z_HD between Groups IIIb and IIIa
Chromosome 26 (Mb)
0.73
5.82
10.92
16.01
21.11
26.20
31.30
36.39
41.49
46.58
51.68

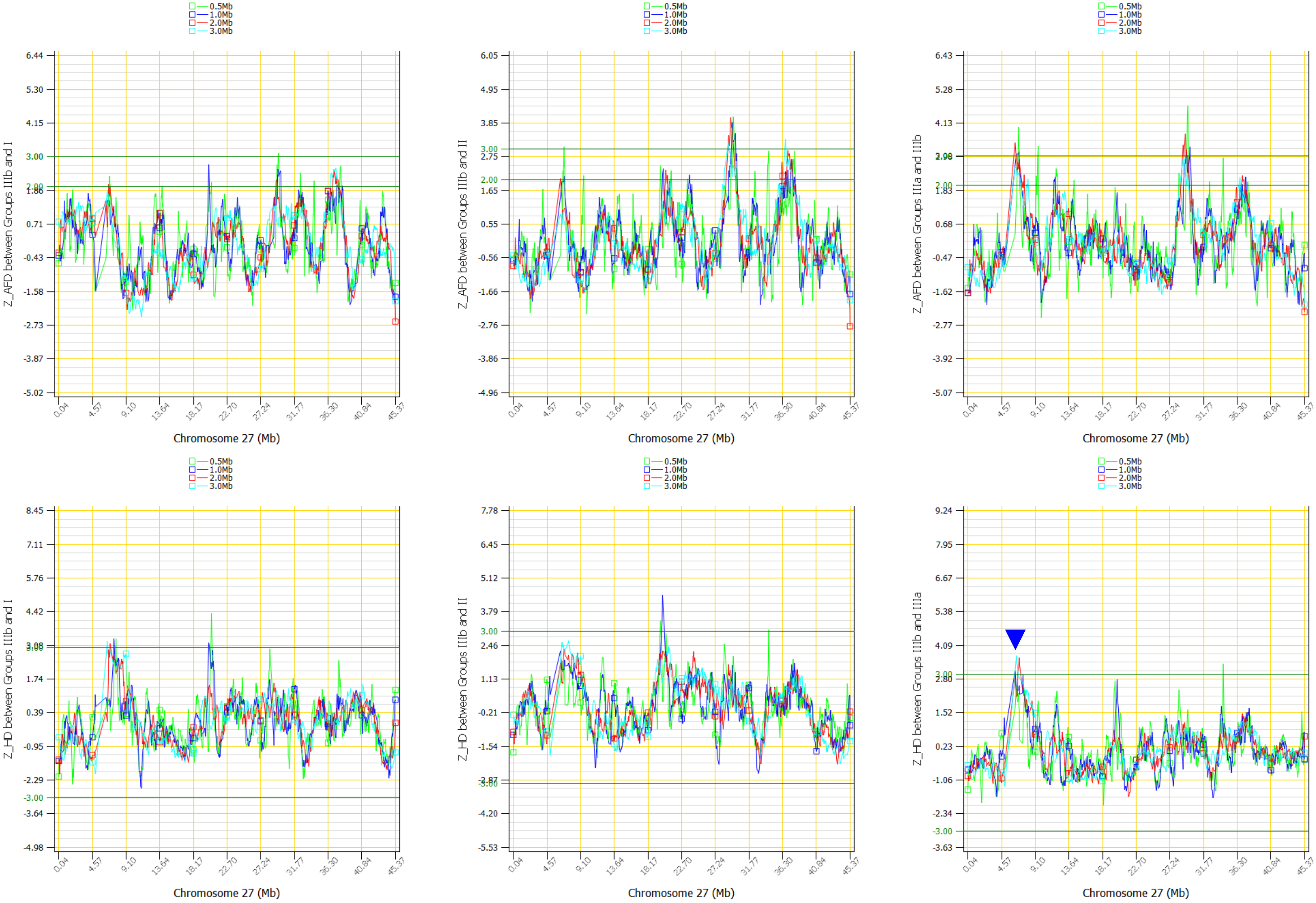
0.5Mb
1.0Mb
2.0Mb
3.0Mb
Z_AFD between Groups IIIb and I
6.44 5.30 4.15 3.00 2.00 1.86 0.71 -0.43 -1.58 -2.73 -3.87 -5.02
0.04 4.57 9.10 13.64 18.17 22.70 27.24 31.77 36.30 40.84 45.37
Chromosome 27 (Mb)
0.5Mb
1.0Mb
2.0Mb
3.0Mb
Z_AFD between Groups IIIb and II
6.05 4.95 3.85 3.00 2.75 2.00 1.65 0.55 -0.56 -1.66 -2.76 -3.86 -4.96
0.04 4.57 9.10 13.64 18.17 22.70 27.24 31.77 36.30 40.84 45.37
Chromosome 27 (Mb)
0.5Mb
1.0Mb
2.0Mb
3.0Mb
Z_AFD between Groups IIIa and IIIb
6.43 5.28 4.13 3.00 2.98 2.00 1.83 0.68 -0.47 -1.62 -2.77 -3.92 -5.07
0.04 4.57 9.10 13.64 18.17 22.70 27.24 31.77 36.30 40.84 45.37
Chromosome 27 (Mb)
0.5Mb
1.0Mb
2.0Mb
3.0Mb
Z_HD between Groups IIIb and I
8.45 7.11 5.76 4.42 3.08 3.00 1.74 0.39 -0.95 -2.29 -3.00 -3.64 -4.98
0.04 4.57 9.10 13.64 18.17 22.70 27.24 31.77 36.30 40.84 45.37
Chromosome 27 (Mb)
0.5Mb
1.0Mb
2.0Mb
3.0Mb
Z_HD between Groups IIIb and II
7.78 6.45 5.12 3.79 3.00 2.46 1.13 -0.21 -1.54 -2.87 -3.00 -4.20 -5.53
0.04 4.57 9.10 13.64 18.17 22.70 27.24 31.77 36.30 40.84 45.37
Chromosome 27 (Mb)
0.5Mb
1.0Mb
2.0Mb
3.0Mb
Z_HD between Groups IIIb and IIIa
9.24 7.95 6.67 5.38 4.09 3.00 2.80 1.52 0.23 -1.06 -2.34 -3.00 -3.63
0.04 4.57 9.10 13.64 18.17 22.70 27.24 31.77 36.30 40.84 45.37
Chromosome 27 (Mb)

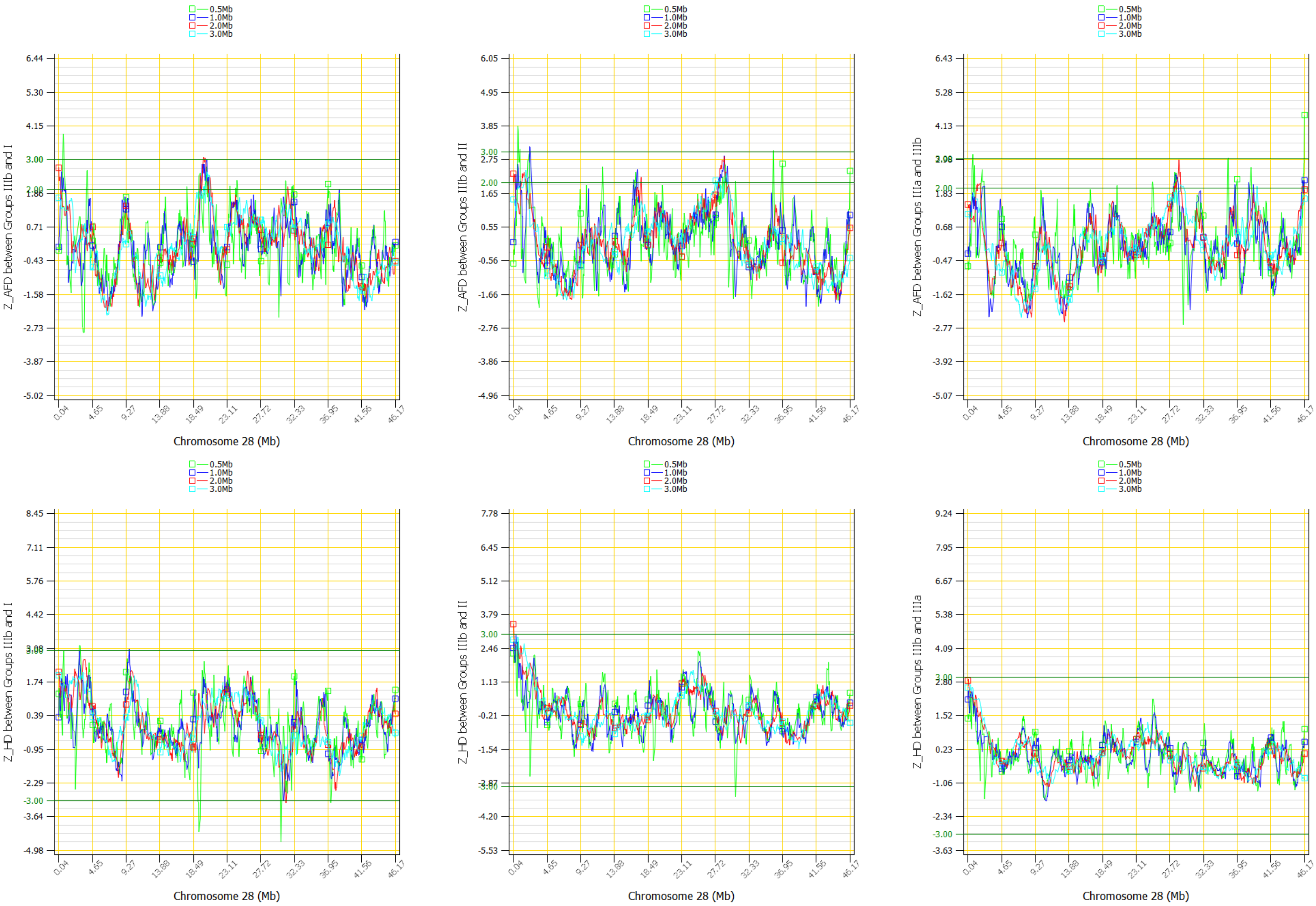
0.5Mb
1.0Mb
2.0Mb
3.0Mb
Z_AFD between Groups IIIb and I
Chromosome 28 (Mb)
Z_AFD between Groups IIIb and II
Chromosome 28 (Mb)
Z_AFD between Groups IIIa and IIIb
Chromosome 28 (Mb)
Z_HD between Groups IIIb and I
Chromosome 28 (Mb)
Z_HD between Groups IIIb and II
Chromosome 28 (Mb)
Z_HD between Groups IIIb and IIIa
Chromosome 28 (Mb)

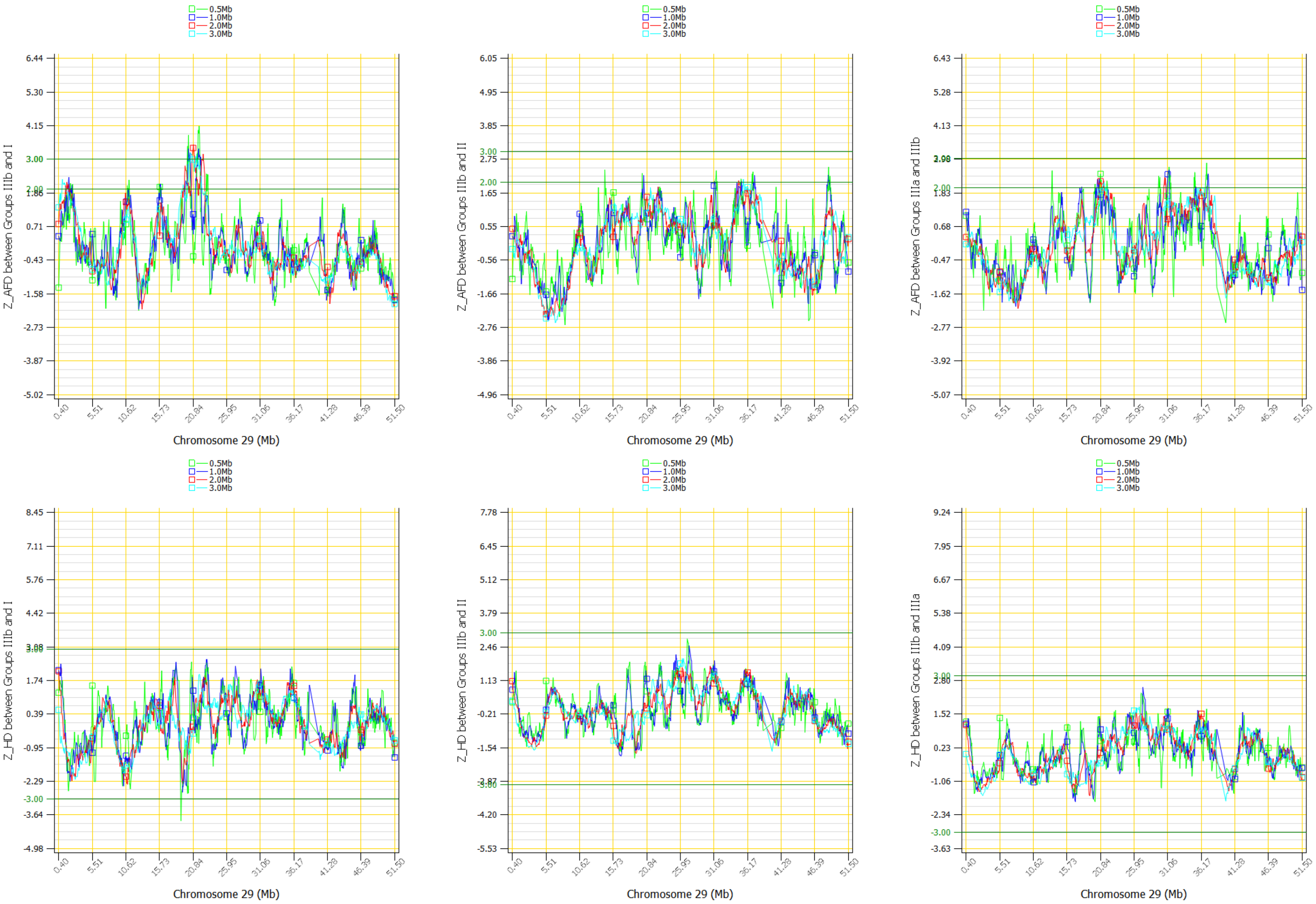
0.5Mb
1.0Mb
2.0Mb
3.0Mb
Z_AFD between Groups IIIb and I
Z_AFD between Groups IIIb and II
Z_AFD between Groups IIIa and IIIb
Z_HD between Groups IIIb and I
Z_HD between Groups IIIb and II
Z_HD between Groups IIIb and IIIa
0.40
5.51
10.62
15.73
20.84
25.95
31.06
36.17
41.28
46.39
51.50
Chromosome 29 (Mb)

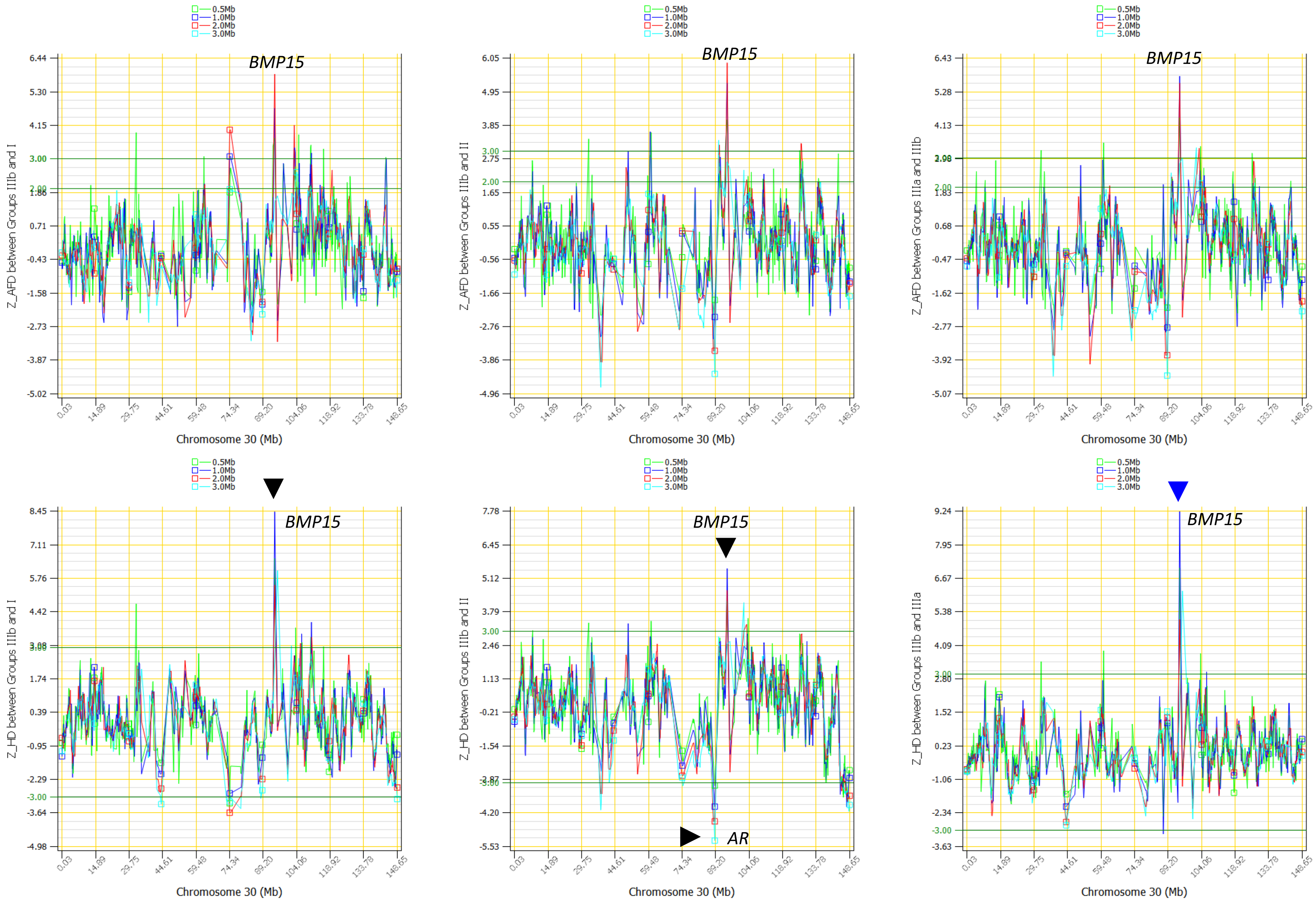

Fig. S7. Long-range differences of allele frequencies and heterozygosity between an elite group (Group IIIb) and the other groups in the selection signature analysis. Left column: the 40 years of selection between Groups IIIb and I. Middle column: the second 20 years of selection between Groups IIIb and II. Right column: the difference between the elite group and their contemporaries (Groups IIIb and IIIa).
