# Supplementary material for: Genome changes due to artificial selection in U.S. Holstein cattle
Source: BMC Genomics. 2019 Feb 11;20:128. doi: 10.1186/s12864-019-5459-x (PMC6371544; doi:10.1186/s12864-019-5459-x)
Supplement: Supplementary file 8 — Table S1. Genome regions with signature of selection detected by long-range frequency differences in 0.5 Mb, 1 Mb, 2 Mb and 3 Mb sliding windows of SNP markers. (PDF 280 kb) [file 12864_2019_5459_MOESM8_ESM.pdf]

Additional file 8: Table S1. Genome regions with signature of selection detected by long-range frequency differences in 0.5Mb, 1Mb, 2Mb and 3Mb sliding windows of SNP markers.

Chr	Position	Size (Kb)	Population comparison	Number of genes	Genes in region (start-end)
1	12964810-17425222 (3Mb sliding windows)	4460.412	2,5,6	0	*NCAM2* at 14.8Mb
1	17790637-18254887	464.25	2	2	*TMPRSS15-CHODL*
1	37862758-38230818	368.06	1,3,4	4	*PROS1-NSUN3*
1	59071436-59337004	265.568	2	4	*ZDHHC23-DRD3*
1	**63951419-65580210**	1628.791	1	29	*IGSF11-GPR156*
1	77485396-78088393	602.997	2,1	9	*CLDN16-TP63*
**1**	**85294656-88698235** (2Mb sliding windows)	3403.579	4,6	42	*SOX2-ZMAT3*
**1**	**94730717-95847141**	1116.424	1	26	*SPATA16-GHSR*, HH2
**1**	**99193415-100200649**	1007.234	2,5	8	*GOLIM4*
1	111997359-112462260	1649.001	3,4	4	*SSR3-KCNAB1*
1	121039462- 121498622	464.901	2	7	*LOC786256-LOC101906885*
1	125943303-126199422	489.63	4	1	*SLC9A9*
1	139381706-140031898	650.192	1,3,4	4	*CPNE4-MRPL3*
1	141205936-141460090	254.154	2	4	*B3GALT5-IGSF5-PCP4*
2	29307365-30011666	704.301	1	3	*LOC100141080-SCN7A*
**2**	**45554413-46010764**	456.351	4	1	*RND3*
2	79257749-80298164	1040.415	5	5	*GPC-MYO1B*
**2**	**84373550-86035295**	1661.745	1,2	13	*SLC39A10-ANKRD44*
**2**	**91221891-92326427**	1104.536	3,4	11	*NOP58-CD28*
2	93505187-93648118	167.381	1	1	*PARD3B*
2	96388684-96612179	223.495	2,1	9	*CCNYL1-CRYGB*
2	98446391-98666598	220.207	2	5	*ACADL-LANCL1*
**2**	**101593916-102214656**	379.362	3,4	3	*IKZF2-SPAG16*
**2**	**116764893-118581255**	1816.362	2,5	9	*DAW1-DNER*
**2**	**120914934-121503634**	588.7	2	18	*ECEL1-AZIN2*

2	128695085-129554709	859.624	1	9	*CLIC4-IL22RA1-PNRC2*
2	133427914-134486603	1058.689	1	23	*RNF186-IGSF21-TAS1R2*
**3**	**8685390-9810337**	1124.947	4,2,5,6	33	*ITLN2-CD244-CD48-CD84-IGSF8-PIGM*, HDR at 9.479Mb
3	11105182-11770282	665.1		26	*OR6K2-CD1B5* (20 olfactory receptors)
**3**	**15357817-15525599**	167.782	1,3	18	*RUSC1-EFNA1*
**3**	**16444546-16881296**	436.75	1,3,4	27	*NUP210L-S100A3*
3	35995151-36440107	444.956	2,4	1	*MGC139448*
3	45405468-46318628	913.16	2	3	*DPYD*
**3**	**52365157-53000242**	635.085	4	2	*LOC100140881-BARHL2*
3	55108725-55537894	463.409	2,4	8	*LOC511531-PKN2*
**3**	**56734548-58105227**	1370.679	4,3,2,1	25	*LMO4-ODF2L*
**3**	**72505537-72743814**	447.474	1,3,2,4	1	*ARPC2*
**3**	**79282515-80265751**	983.236	4	6	*PDE4B-DNAJC6*
**3**	**91402252-92304109**	137.745	4,2	14	*USP24-DHCR24-ACOT11*
**3**	**110766510-111276607**	510.097	4,6	11	*TFAP2E-ZMYM6*
4	18173214-18689580	516.366	1, 2	0	*NXPH1↔PHF14*
4	39012513-39435115	422.602	1, 2	1	*HGF*
**4**	**43600871-43763107**	162.236	1, 2	3	*PHTF2-RSBN1L*
**4**	**56031281-56222505**	191.224	1,2	2	*ZNF277-DOCK4*
**4**	**60917347-61444963**	527.616	4	8	*AOAH-EEPD1*
4	77074551-77249037	174.486	2	4	*RAMP3-NACAD*
**4**	**81624828-81830858**	206.03	1,3	5	*SUGCT-CDK13*
**4**	**91051469-91417417**	365.948	1,5	0	*POT1↔ZNF800*
4	97392045-98338655	946.61	1	2	*CHCHD3-EXOC4*
**4**	**105827564-106051549**	223.985	4,2	12	*WEE2-LOC519161*
5	19531689-20609872	1078.183	1,2	1	*ATP2B1*
5	26661043-27235668	574.625	4,6	30	*ATF7-KRT8* (27.17-27.89Mb, 33 keratin genes)
5	34937478-36388805	189.322	2,4,3,1	1	*TMEM117*
5	46279541-46741275	461.734	3,4,1	2	*DYRK2-CAND1*

5	59665562-59840330	174.768	1,3,6	8	>120 olfactory receptors in 58.07-60.54Mb
5	72646590-72982750	336.16	5	1	*LARGE*
**5**	**104116518-105105448**	988.93	1	29	*PIANP-ANO2*
5	111331877-111380409	416.621	5,6	22	*MEI1-TCF20*
5	114290454-114499520	209.066	2,4	3	*ARFGAP3-TTL1*
6	4049985-4746604	819.612	5	3	*QRFPR-PRDM5*
6	6992427-7812039	819.612	1,2,4	15	*PDE5A-METTL4*
6	10572605-11053007	480.402	1,2,4	0	
6	**40236966-41123393**	886.427	7	0	
6	57947092-57991420	44.328	1,2	0	*DTHD1↔LOC525433*
**6**	**74378643-75426216**	1047.573	1,3,4	0	*IGFBP7↔LPHN3*
**6**	**83919789-84819700**	899.911	7	15	*LOC101908113-**GNRHR**- TMPRSS11E*
6	90075383-90188972	552.763	8	8	*COX18-RASSF6*
6	107576198-107987121	410.923	4	14	*DBDR-ADD1*
7	11486814-12752376	1265.562	6	43	*LOC789580-IL27RA (*9.2-12.2Mb: 139 genes, mostly olfactory receptors and EMR2 receptors)
**7**	**54455147-57844905**	3389.758	1,2,3,4	42	*HDAC3-KCTD16*
**7**	**68627428-71892762**	1088.061	4	20	*SGCD -CLINT1*
**7**	**90900133-91762948**	862.815	4	0	*MEF2C↔MIR3660*
7	93834124-94105221	271.097	5,6	0	*ARRDC3↔ NR2F1*
**8**	**6201599-7068364**	866.765	4,5,6	6	*FBXO8-GLRA3*
8	34584476-35370804	786.328	1,2,5,6	0	
**8**	**37706259-39306605**	1600.346	2,3,4	27	*KDM4C-RIC1*
8	50111844-50553225	441.381	2,4	0	*ANXA1↔RORB*
8	53899633-54627149	727.516	2	7	*GNA14-PSAT1*
8	55505902-56244907	739.005	1,3	1	*TLE4*
**8**	**62359326-62544260**	184.934	1	5	*DCAF10-SHB*
8	73435613-73907982	736.323	2	7	*DOCK5-CDCA2*
**8**	**78701950-80280501**	1578.551	4	12	*SLC28A3-AGTPBP1*

**8**	**85786900-86128908**	342.008	3,4	8	*TSTD2-GABBR2*
**8**	**93276825-94327318**	1050.493	4,5,6	1	*CYLC2*
**8**	108652993-109059409	406.416	5	1	*TLR4*
**9**	**38385371-38992847**	607.476	2	9	*LAMA4-WISP3*
9	43595237-44075848	480.611	1,2,4	5	*QRSL1-AIM1*
9	47959651-48437028	477.377	4	0	*HACE1↔GRIK2*
9	50027556-50832512	804.956	5	4	*ASCC3-MCHR2*
9	54668173-55117119	448.946	1	4	*LOC782007-MANEA*
**9**	**62751655-63116931**	365.276	3,4	1	*SPACA1*
9	77733879-78276718	542.839	5	13	*PERP- LOC100296379*
**9**	**87190233-88964135**	1773.902	4	34	*UST-PLEKHG1*
**10**	**6224953-6871209**	646.256	2	7	*GCNT4-COL4A3BP*
**10**	**21516136-22008344**	492.208	1,3,4	32	*SLC7A8-LOC785963*
					22.1-25.6Mb: 199 genes, mostly T-cell receptor alpha chain V region, Ig alpha, kappa and lambda chains, Ig heavy chains, and olfactory receptors
**10**	**25598535-26289588**	542.719	1,3,4	32	*LOC101904911-BRB*
**10**	**30159278-30367930**	208.652	3,4	4	*ARHGAP11A-ACTC1*
**10**	**36465813-37290812**	824.999	4,5	25	*ZFYVE19-MAPKBP1*
10	42432658-42796747	364.089	1, 4	12	*LOC782862-KLHDC2*
10	51001410-52033595	1032.185	6	13	*MYO1E-LOC101904602*
10	61109072-62309052	1199.98	1, 2	15	*GALK-FBN1*
10	72280968-73487550	1206.582	1,2,3,4	21	*COX7C-SLC38A6*
**10**	**85302011-86511068**	1209.057	1,2,4	40	*C10H14orf169-PROX2*
11	6602495-7047686	445.191	1,2	6	*MAP4K4-IL1R2-IL1R1-IL1RL2, IL1RL1-IL18R1-IL8RAP,*
**11**	**15814741-16759475**	944.734	2	6	*LTBP1-FAN98A*
**11**	**28577854-29495322**	917.468	1,3	19	*EPAS1-CALM2*
11	37876375-38474579	598.204	1,2	6	*CCDC88A-EFEMP1*
**11**	**40183335-40236222**	872	4	0	*CCDC85A↔VRK2*

11	44458785-45346657	887.872	5,6	13	*EDAR- SLC5A7*
**11**	**72844521-73823078**	978.557	3	22	*KCNK3-DTNB*
**11**	**80450475-80982482**	532.007	4	3	*KCNS3-GEN1*
12	11353817-11824890	471.073	2	10	*KBTBD6-VWA8*
12	19676138-20180276	504.138	1,2,4	0	*KCNRG↔RNASEH2B*
**12**	**35886159-37211264**	1325.105	1,2,3,4,5	26	*LATS2-LOC101904113*
12	43551814-43866499	314.685	1	0	*LOC101902172 ↔ KLHL1*
**12**	**49760634-49964419**	203.785	1	0	*KLF12 ↔ TBC1D4*
**13**	**5239814-6282908**	1043.094	6	3	*SRY-ISM1*
**13**	**6396120-6724839**	328.719	1	0	*BTBD3 ↔ SPTLC3*
**13**	**10747684-10966294**	218.61	1, 2	8	*SNRPB2-NINL*
**13**	**16560433-17146206**	585.773	1, 2	6	*SFMBT2-(TRNAF-GAA)*
**13**	**22904772-24416810**	1512.038	2,4,5,6	17	*SKID1-MSRB2*
13	34887980-35263460	375.48	2,5	4	*SVIL-SACS-SGCG*
**13**	**37089930-37429612**	339.682	4	2	*LOC100337001-PTCHD3*
**13**	**49963612-50029350**	65.738	4	0	*BMP2*-(23 ubiquitin-conjugating enzyme pseudogenes)
13	53618441-53809825	191.384	1	2	*PDYN-SIRPA*
**13**	**57876144-58285197**	409.053	4	14	*TUBB1-STX16*
**13**	**76616107-77686707**	1070.6	1	13	*ZMYND8-PREX1*
**14**	**1463676-2138926**	675.25	7	59	*ZNF34-SPATC1*
**14**	**33716508-35052708**	1336.2	1,3,4	7	*CPA6-C14H8orf34*
**14**	**35744766-36092385**	347.619	1,2	2	*PRDM14-NCOA2*
**14**	**66118162-66771994**	653.832	1	9	*RNF19A-VPS13B*
**14**	**68450192-68962221**	512.029	4,5	8	*ERICH5-MTDH-LOC514181*
14	77292340-78092917	800.577	2	5	*SLC2A5-LOC783984*
15	2317117-3550238	1233.121	2,4,5,6	4	*GRIA4-CASP1*
15	12412932-12682016	269.084	1	0	*CNTN5↔LOC100300167*
15	17608371-17812223	203.852	3	2	*SLC35F2-RAB39A*

15	32617589-32861621	244.032	2,5	1	*SORL1*
15	52214348-52660739	446.391	2	25	*LOC407145-CLPB*
15	54365783-54617640	251.857	2	10	*PPME1-LIPT2*
**15**	**63033190-63216353**	183.163	4	2	*IMMP1L-ELP4*
**15**	**74332484-74924833**	592.349	1	9	*TTC17-C15H11orf96*
**15**	**81964881-82260685**	1890.873	2	176	*>150 olfactory receptors in 79-82Mb*
16	16095676-17904939	1809.263	1,2,3,4	1	*BRINP3*
16	22302686-23108285	805.599	2,3,5	3	*LOC529125-TGFB2*
**16**	**43618684-44459269**	840.585	1,2,3,4,5	18	*CASZ1-NMNAT1*
**16**	**58405786-59021630**	615.844	1,2,4	1	*RFWD2*
**16**	**66009531-66543047**	533.516	2	10	*SMG7-GLT25D2*
17	10594226-11231535	637.309	4	3	*PRMT9-EDNRA*
17	13386146-13635843	249.697	1,3	3	*ANAPC10-HHIP*
17	14782322-15372415	516.476	4,2,5,6	2	*USP38-INPP4B*
17	26712567-27127895	415.328	2,4,5,6	0	*PCDH10 ↔ C17H4orf33*
17	32601443-33098572	1213.741	4	1	*FAT4*
**17**	**38467666-39681407**	1050.161	1,2,3,4	0	*LOC512119↔ULBP3*
**17**	**50047172-51097333**	570.443	1,3,4	0	3 testis-specific proteins
**17**	**54081495-54651938**	488.063	2	19	*DNAH10-PITPNM2*
18	5383748-5871811	511.652	1	1	*WWOX*
**18**	**8467054-9606800**	1139.746	1	12	*PLCG2-CDH13*
**18**	**14115136-14626788**	511.652	4,6	18	*CBFA2T3-SPATA33-VPS9D1*, HBR at 14.75Mb
18	27965784-28670292	704.508	2	1	*LOC781392*
18	34794005-35027876	362.316	4	22	*CES4A-PLEKHG4*
18	49396303-49758619	788.291	4,5,6	21	*SNAI3-ANKRD11*
**18**	**50581375-51369666**	322.523	2	41	*CYP2B6-GSK3A*
**18**	**53790007-54112530**	471.93	2	17	*RSPH6A-LOC782346*
**18**	**54556209-55028139**	2725.71	1,2	14	*ZC3H4-GLTSCR1*

**18**	**55807264-56642741**	835.477	8	68	*MAMSTR-MED25*
18	58696066-61421776	212.676	2,3,4,5,6	122	*LOC787057-LOC527385* 57.58-62.53Mb: 249 genes, mostly zinc fingers, cationic amino acid transporters, sialic acid-binding Ig-like, and vomeronasal receptors
19	6241437-6454113	690.751	4,6	8	*TMEM100-PCTP*
**19**	**20878990-21569741**	451.346	1, 3	21	*SLC46A1-COX7C*
**19**	**27643677-28095023**	438.183	4	40	*EIF5A-DNAH2*
**19**	**34229311-34667494**	506.328	4	16	*SPECC1-RNF112*
**19**	**52557638-53063966**	841.393	1,3,4	9	*RPTOR-CARD14*
**19**	**62805137-63646530**	375.519	1,2,4	9	*AXIN2-CACNG4*
**20**	**9919863-10295382**	123.679	1,2	11	*MCCC2-RAD17*
**20**	**23593803-23717482**	585.334	4	1	*PPAP2A*
**20**	**26917749-27503083**	319.185	1,2	0	*PELO↔ ISL1*
**20**	**30079775-30398960**	319.185	4	4	*MRPS30-LOC614882*
**20**	**38453649-38606353**	152.704	1 (EHH)	1	*SPEF2*
**20**	**38920878-39350230**	429.352	1 (EHH)	7	*PRLR-TTC23L*
20	42195463-44040879	1845.416	1,2	4	*CDH6-(TRNAC-GCA)*
20	48114351-49053595	939.244	2,4,5,6	2	*(TRNAF-AAA)-CDH10*
**21**	**7070882-7637712**	566.83	1,2	6	*MEF2A-LRRC28*
**21**	**17531298-18167162**	635.864	4,6	0	*KLHL25↔NTRK3*
**21**	**56348332-57186819**	838.487	4	16	*FRMD5-FBLN5*
**21**	**60851191-61333785**	482.594	1	14	*LOC787624- MIR2284A*
**21**	**64129449-66380363**	2250.914	1,2,3,4	11	*BCL11B-EML1*
21	67366627-67845758	479.131	3,4	90	90 RNA genes
**21**	**69853542-70931906**	1078.364	1, 2	28	*APOPT1-CEP170b,AKT1*
**22**	**13172658-14654094**	1481.436	4	18	*MYRIP-ABHD5*
22	20257375-21431682	1174.307	2,4,5	0	*GRM7↔EDME1*
22	33983201-34416644	433.443	1,2	1	*SUCLG2*

**22**	**50926015-51682391**	756.376	2	51	*MST1R-PRKAR2A*
22	53695239-54103673	408.434	1	8	*CCR3-CCR9*
23	3167662-3239987	452.805	2	0	*BEND6↔DST*
23	7627089-7874236	72.325	1,2	16	*BAK1-MLN*
**23**	**25178791-27992880**	247.147	1,2,3,4	132	*ELOVL5-DDR1*
**23**	**30039692-31222894**	2814.09	4,6	166	(TRNAT-CGU)- ZNF322<br>28.8-31.3Mb: 297 genes, mostly transfer RNA's and olfactory receptors
**23**	**36509046-37321457**	1183.202	3,4	2	*CDKAL1-E2F3*
23	39763063-39896138	812.411	4	6	*CAP2-RBM24*
24	6659975-7838578	133.075	1, 2	8	*SOCS6-DOK6*
**24**	**13210427-13607982**	1178.603	3,4	0	*STY4↔PIK3C3*
**24**	**15692639-16660415**	397.555	4,5,6	0	*PIK3C3↔BRUNOL4*
**24**	**17890979-18369994**	967.776	1	0	*PIK3C3↔ BRUNOL4*
**24**	**49190314-50568228**	479.015	3,4	14	*DYM-SKA1*
**24**	**59564307-61140716**	1377.914	4	14	*MC4R-KIAA1468*
**25**	**23825593-26337016**	1576.409	4,5	37	*LOC104968447-IL4R-IL21R-CD19-IL27*
25	29332965-29665645	2511.423	2	2	*CALN1-WBSCR17*
26	16999066-17465791	332.68	1	8	*TCTN3-BLNK*
**26**	**19507012-20474308**	466.725	1,5	5	*HPSE2-GOT1*
**26**	**36056808-37203584**	967.296	3,4	4	*ATRNL1-PNLIPRP3*
26	48944472-49253877	1146.776	4	1	*MGMT*
27	7381313-8238519	309.405	4,5,6	2	*NEIL3-AGA*
27	20559148-20863121	857.206	2,3,4	1	*TUSC3*
27	29419113-29650426	303.973	2	0	
**27**	**32405436-33749694**	231.313	1	34	*ZNF703-PLEKHA2*
**28**	**3637555-3998395**	1344.258	1,3,4	4	*FAM89A-C28H1orf198*
**28**	**9431460-9827919**	396.459	1,3,4	3	*ACTN2-RYR2*
**28**	**19436357-20480901**	1044.544	1,2,3,4	10	*JMJD1C-LOC781358*
28	31053570-31371629	318.059	1,3,4	10	*DUSP13-C28H10orf11*

28	39700262-39971968	271.706	1	1	*CCSER2*
29	16071248-17402388	1311.140	7	1	*TENM4*
**29**	**19003267-19398242**	394.975	1,3,4	1	*LOC524642*
29	19928179-20312863	531.222	2	1	*MGC157332*
29	21399349-21930571	511.176	2	0	
**29**	**43328607-43839783**	267.93	1	21	*SLC22A11-BATF2*
**29**	**47422720-47668622**	196.567	1	5	*CCND11-FGF4*
**X**	**42024368-43999854**	1975.486	4	7	*LOC786332-LOC100139929*
**X**	**47777883-48186380**	408.497	3,5,6	7	*DIAPH2*
**X**	**73035611-80134199 (2Mb windows)**	7098.588	3,4	51	LOC104968770-LOC100139951
**X**	**88170589-88840217 (2Mb or 3Mb sliding windows)**	669.628	3	1	*AR*
**X**	**93574264-94321737**	747.473	2,4,5,6	8	*SHROOM4-NUDT11*
X	101743604-103244750	1501.146	5	13	*ARHGEF9-LOC104969966*
X	105003475-105267785	264.31	1, 2	3	*NDP-MAOB*
**X**	**109945924-112346433**	2400.509	1,2	39	*MID1IP1-LOC539382*
X	115968607-116335568	366.961	1,5	1	*DMD*
X	143626961-144035042	408.081	2	11	*LOC783713-EIF1AY*

'Chr' and 'Position' in bold face indicate selection signature that contained or were near genes with documented fertility functions. In the column of 'Population comparison', 1 = standardized absolute allele frequency differences (AFD) between Groups I and III, 2= standardized absolute AFD between Groups I and IIIb, 3 = standardized heterozygosity differences (HD) between Groups III and I, 4 = standardized HD between Groups IIIb and I, 5 = standardized absolute allele frequency differences (AFD) between Groups IIIa and IIIb, 6 = standardized HD between Groups IIIb and IIIa, 7 = standardized AFD between Groups I and II, 8 = standardized AFD between Groups II and III.
