# Supplementary material for: Genome changes due to artificial selection in U.S. Holstein cattle
Source: BMC Genomics. 2019 Feb 11;20:128. doi: 10.1186/s12864-019-5459-x (PMC6371544; doi:10.1186/s12864-019-5459-x)
Supplement: Supplementary file 9 — Table S2. Fertility genes in or near chromosome regions subjected to genetic selection since 1964. (PDF 456 kb) [file 12864_2019_5459_MOESM9_ESM.pdf]

Additional file 9: Table S2. Fertility genes in or near chromosome regions subjected to genetic selection since 1964.

Chr	Selection signature position (bp)	Potential fertility gene		Documented fertility function/effect
		Gene symbol	Gene name	
1	63951419-65580210	*COX17*	cytochrome c oxidase copper chaperone	mouse embryos died between embryonic days 8.5 and 10 (1)
1	85959409-87774968	*SOX2*, 85.9 Mb	SRY (sex determining region Y)-box 2	abnormalities in the hypothalamo-pituitary-gonadal axis in mice and humans, including peri-implantation lethality in mice and male fertility problems (2)
		PIK3CA, 88.5 Mb	phosphatidylinositol-4, 5-bisphosphate 3-kinase, catalytic subunit alpha	defective uterine development in mice (3)
1	94730717-95847141 (*GPX5* is within *SPATA16*)	*SPATA16*	spermatogenesis associated 16	a homozygous genotype associated with male infertility in human (4)
		GPX5	glutathione peroxidase 5	sperm DNA integrity in mice (5)
		GHSR	growth hormone secretagogue receptor	association with infertility in women (6)
		HH2, 93.2-98.1 Mb	Holstein lethal haplotype 2	lower conception rate in Holstein cattle (7)
1	99193415-100200649	*MECOM (EVI1),* 99.1 Mb	MDS1 and EVI1 complex	midgestation embryo development and embryonic lethal mutation in mice (8)
2	45554413-46010764	*TSG-6*, 44.9 Mb	tumor necrosis factor, alpha-induced protein 6 (TNFAIP6), mRNA	severe fertility defects in female mice including female infertility (9)
		MMADHC, 46.7 Mb	methylmalonic aciduria (cobalamin deficiency) cblD type, with homocystinuria	required for pre-implantation embryogenesis in mice (10)
2	84373550-86035295	*SLC39A10*	solute carrier family 39 (zinc transporter), member 10	embryonic development (11) and spermatogenesis (12)
		PGAP1	post-GPI attachment to proteins 1	otocephaly and male infertility in mice (13)
2	91221891-92326427	*SUMO1*, 91.2 Mb	SMT3 suppressor of mif two 3 homolog 1	associated with human semen quality (14)
		BMPR2	bone morphogenetic protein receptor,	required for postimplantation uterine function and

		CD28	type II (serine/threonine kinase)<br>cluster of differentiation 28	pregnancy maintenance in mice (15)<br>participates in the process of embryo implantation in mice (16)
2	101835294-102214656	*SPAG16*	sperm associated antigen 16	impaired sperm mobility and male infertility in mice (17)
2	116764893-118581255	*SPATA3*	spermatogenesis associated 3	mouse spermatogenesis (18)
2	120914934-121503634	*AZIN2*<br>*PUM1*, 123.1 Mb	antizyme inhibitor 2<br>pumilio RNA-binding family member 1	spermiogenesis in mice (19)<br>reduced sperm count (20) and reduction in female fertility (21), and suppresses multiple activators of p53 to safeguard spermatogenesis in mice (22)
3	8685390-9810337	*CD48*<br>*VANGL2*<br><br>*ATP1A4*	cluster of differentiation 48<br>vang-like 2<br><br>ATPase, Na+/K+ transporting, alpha 4	local environment of female reproductive track (23)<br>embryo implantation during early pregnancy in mice (24)<br>male fertility in mice (25)
3<br>3	15357817-15525599<br>16444546-16881296	*IL6R,* 16.2 Mb	interleukin 6 receptor	sperm mobility (26) and involvement in recurrent miscarriage (27)
3	52365157-53000242	*HFM1*, 52.2 Mb	ATP-dependent DNA helicase homolog	required for crossover formation and complete synapsis of homologous chromosomes during meiosis in mice (28)
3	56734548-58105227	*LMO4*<br>*SH3GLB1*	LIM domain only 4<br>SH3-domain GRB2-like endophilin B1	perinatal lethality in mice (29)<br>human sperm mobility (30)
3	72296340-72743814	*ARPC2*	actin related protein 2/3 complex, subunit 2, 34kDa	mouse embryo development (31)
3	79282515-80265751	*PDE4B*<br>*INSL5*	phosphodiesterase 4B, cAMP-specific<br>insulin-like 5	growth and fertility in mice (32)<br>impaired fertility in mice (33)
3	91402252-92304109	*DHCR24*	24-dehydrocholesterol reductase	horse spermatogenesis (34)
3	110766510-111276607	*SFPQ*<br>*AGO4,* 110.5 Mb<br>*TEKT2*, 110.3 Mb	splicing factor proline/glutamine-rich<br>argonaute 4<br>tektin 2 (testicular)	expressed in mouse sertoli cells (35)<br>male fertility defects in mice (36)<br>*Tekt2*-null sperm display flagellar bending and

				reduced motility due to a disruption in the dynein inner arm (37, 38)
4	43600871-43763107	*TMEM60*<br>*RSBN1L*	transmembrane protein 60<br>round spermatid basic protein 1-like	premature ovarian failure in women (39)
4	56031281-56222505	*IMMP2L*, 57.1 Mb	inner mitochondrial membrane peptidase-like	mitochondrial function and fertility in male and female mice (40)
4	60917347-61444963	*ELMO1*<br>*SEPT7*, 61.6 Mb<br>*RBX1*, 62.1 Mb<br>*DPY19L2*, 62.3 Mb	engulfment and cell motility 1<br>septin 7<br>ring-box 1, E3 ubiquitin protein ligase<br>dpy-19-like 2	apoptotic germ cell clearance and spermatogenesis (41)<br>sperm morphology (42)<br>infertility (43) and globozoospermia (44, 45) in man and in mice (46)
4	77074551-77249037	*IGFBP1*, 76.7 Mb<br>*IGFBP3*, 76.7 Mb	insulin-like growth factor binding protein 1<br>insulin-like growth factor binding protein 3	multifunctional role in the human female reproductive tract (47)<br>oocyte maturation and development (48)
4	81624828-81830858	*POU6F2*	POU class 6 homeobox 2	mouse kidney embryogenesis (49)
4	91051469-91417417	*GPR37*, 89.8 Mb	G protein-coupled receptor 37	specific modulator of murine testis Dhh mitogenic signaling and SC proliferation and maturation (50)
4	105827564-106051549	*PRSS37*	protease, serine 37	male fertility in mice (51)
5	19931600-20609872	*KITLG*, 18.3 Mb	KIT ligand	female fertility protein (52)
5	34937478-36419523	*NELL2*, 35.7 Mb	neural EGFL like 2	maintenance of the normal female reproductive cycle in mammals (53)
5	103035242-104714350	*ACRBP*<br>*CD9*<br>*FGF23*, 106.2 Mb<br>*CCND2*, 106.3 Mb<br>*PARP11*, 106.6 Mb	acrosin binding protein<br>cluster of differentiation 9<br>fibroblast growth factor 23<br>cyclin D2<br>poly(ADP-ribose) polymerase family member 11	swine semen quality (54)<br>severe effect on female fertility in mice (55-59) and sperm-egg fusion(59)<br>human embryonic development (60)<br>female fertility protein (52)<br>Deletion of the *PARP11* gene results in teratozoospermia and male infertility in mice (61)

6	40236966-41123393	*SLIT2*, 41.2 Mb	slit guidance ligand 2	follicle formation during fetal ovary development (62)
6	74378643-75426216	*IGFBP7*, 74.1 Mb	insulin-like growth factor binding protein 7	regulates uterine receptivity in mice (62)
6	83919789-85743833	*GNRHR*	gonadotropin-releasing hormone receptor	association with the number of corpora lutea in swine (63), protein misfolding and hypogonadotropic hypogonadism in humans, and testis size reduction and ovulation failure in mice (64)
6	89772651-90188972	*AFP*<br><br>*MTHFD2L*	alpha-fetoprotein<br><br>methylenetetrahydrofolate dehydrogenase (NADP+ dependent) 2 like	controls female fertility and prenatal development of the gonadotropin-releasing hormone pathway (65)<br>deletion of Mthfd1l causes embryonic lethality and neural tube and craniofacial defects in mice (66)
7	54455147-57844905	*PCDH12*<br>*FGF1*	protocadherin 12<br>fibroblast growth factor 1	development of mouse placenta (67)<br>embryonic development (68)
7	69305308-71892762	*ADAM19*<br>*SOX30*	ADAM metallopeptidase domain 19<br>SRY (sex determining region Y)-box 30	early pregnancy (69)<br>testis development in mice (70)
7	90900133-91762948	*POLR3G*, 92.4 Mb	polymerase (RNA) III (DNA directed) polypeptide G (32kD)	uniquely expressed among human oocytes (71)
8	6201599-7068364	*HMGB2,* 5.6 Mb	high mobility group box 2	reduced fertility and spermatogenesis defects in mice (72)
8	27710954-29425215	*PSIP1*		Disruption of Ledgf/Psip1 results in perinatal mortality and homeotic skeletal transformations (73)
8	37706259-39306605	*IL33*<br><br>*ERMP1*	interleukin 33<br><br>endoplasmic reticulum metallopeptidase 1	uterine environment in women and pregnancy failure in mice (74)<br>polyovular follicles in the rat ovary (75)
8	62359326-62544260	*SHB*	src homology 2 domain-containing transforming protein B	regulation of mouse oocyte maturation (76)

8	78701950-80280501	*NTRK2*<br><br>*AGTPBP1*	neurotrophic tyrosine kinase, receptor, type 2<br>ATP/GTP binding protein 1	follicle assembly and early follicular development (77) and infertility in mice (78)<br>abnormal sperm development in spermatogenesis (79)
8	85786900-86128908	*FANCC*, 83.0 Mb<br><br>*TSPY*, 83.2 Mb	Fanconi anemia, complementation group C<br>testis specific protein, Y-linked 1	reduced male and female fertility in mice(80)<br><br>human male fertility (81)
8	93276825-94327318	*CYLC2*<br><br><br>*SMC2*, 95.4 Mb	cylicin, basic protein of sperm head cytoskeleton 2<br><br>structural maintenance of chromosomes 2	specifically expressed in testis, part of the cytoskeletal calyx of mammalian sperm heads, role in the morphogenesis of the sperm head (82)<br>causal gene of HH3 (7)
9	38385371-38992847	*REV3L* at 39.4 Mb	REV3-like, polymerase (DNA directed), zeta, catalytic subunit	embryonic lethality in mice (83)
9	50027556-50832512	*MCHR2*	melanin-concentrating hormone receptor 2	inhibits GnRH neurons and blocks kisspeptin activation, linking energy balance to reproduction (84)
9	62751655-63116931	*SPACA1*	sperm acrosome associated 1	abnormally shaped sperm heads reminiscent of globozoospermia (85)
9	87190233-88964135	*LATS1*<br>*ESR1*	large tumor suppressor kinase 1<br>estrogen receptor 1	infertility and growth retardation in mice (86)<br>human male fertility (87) and mouse female fertility (88)
10	6224953-6871209	*HMGCR*	3-hydroxy-3-methylglutaryl-CoA reductase	crucial for early development of the mouse embryos (89)
10	21516136-22008344	*TSSK4*, 20.8 Mb	testis-specific serine kinase 4	essential for maintaining the structural integrity of sperm flagellum (90)
10	25598535-26141254	*NDRG2*<br>*ANG2*	NDRG family member 2<br>angiogenin, ribonuclease A family, member 2	male infertility in men and mice (91)<br>*ANG2* is increased in follicular fluid of polycystic ovarian syndrome women and correlates with number of oocytes retrieved (92)
10	30159278-30367930	*GREM1*	gremlin 1	human embryo development (93) and diminished

				ovarian reserve in women (94)
10	36465813-37290812	*TYRO3*	TYRO3 protein tyrosine kinase 3	essential regulators of mammalian spermatogenesis (95), modulate female reproduction (96)
10	85302011-86511068	*NPC2*<br><br><br>*PGF*<br><br>*EIF2B2*, 86.7 Mb<br><br>*MLH3*, 86.7 Mb<br>*TTLL5*, 88.0 Mb<br><br>*ESRRB*, 88.8 Mb	Niemann-Pick disease, type C2<br><br><br>placental growth factor<br><br>eukaryotic translation initiation factor 2B, subunit 2 beta, 39kDa<br>mutL homolog 3<br>tubulin tyrosine ligase-like family member 5<br>estrogen-related receptor beta	infertile female mice (97), spermatozoa from mice deficient in NPC2 protein have defective cholesterol content and reduced in vitro fertilising ability (98)<br>endometrium, pregnancy and implantation failure in women (99)<br>premature ovarian failure(100)<br><br>MLH3-/- mice are viable but sterile (101)<br>sperm malformation and infertility (102)<br><br>differentially expressed in patients with non-obstructive azoospermia (103)
11	15814741- 16759475	*BIRC6*, 15Mb	baculoviral IAP repeat containing 6	depletion of BIRC6 leads to retarded bovine early embryonic development and blastocyst formation in vitro (104)
11	28577854-29495322	*EPAS1*<br>*LHCGR,* 30.8 Mb<br><br>*FSHR*, 31.1 Mb	endothelial PAS domain protein 1<br>luteinizing hormone/choriogonadotropin receptor<br>follicle stimulating hormone receptor	spermatogenesis in mice (105)<br>fertility in human (106, 107), cattle (108) and mouse(52)<br>pubertal development and fertility in males and females (109)
11	40183335-40236222	*VRK2*, 40.5 Mb	vaccinia related kinase 2	germ cell development in male mice (110)
11	72844521-73823078	*RAB10*	RAB10, member RAS oncogene family	early embryonic lethality in mice (111)
11	80450475-80982482	*SMC6,* 81.0 Mb	DNA repair protein SMC6	completion of meiotic division in mice (112)
12	35886159-37211264	*FGF9*<br>*LATS2*	fibroblast growth factor 9<br>large tumor suppressor kinase 2	testicular development in marsupial (113)<br>modulator of estrogen receptor alpha (114),

		RNF17	ring finger protein 17	embryonic development and genomic integrity (115) spermiogenesis and differentiation of male germ cells (116)
12	49760634-49964419	*UCHL3,* 51.0 Mb	ubiquitin carboxyl-terminal esterase L3	required for normal oocyte maturation and fertilization (117)
13	6396120-6724839	*SRY*, 5.4 Mb<br>*ISM1*, 6.6 Mb<br>*FLRT3*, 8.0 Mb	sex determining region of Chr Y<br>isthmin 1, angiogenesis inhibitor<br>fibronectin leucine rich transmembrane protein 3	XY females with gonadal dysgenesis (Swyer syndrome) and XX male syndrome (118)<br>embryo quality (119), embryo development, growth and pregnancy rate in women (120)<br>cell adhesion and tissue morphogenesis in the developing mouse embryo (121)
13	10747684-10966294	*KIF16B*, 10.2 Mb	kinesin family member 16B	early embryonic development (122)
13	16560433- 17146206	*SFMBT2*	Scm-like with four mbt domains 2	trophoblast maintenance and placenta development in mice (123)
13	22904772-24416810	*SPAG6*	sperm associated antigen 6	male infertility in mice (87)
13	37089930-37429612	*PTCHD3*	patched domain containing 3	male germ cell-specific gene (124)
13	49963612-50029350	*BMP2*	bone morphogenetic protein 2	critical for the murine uterine decidual response (125)
13	57876144-58285197	*RAE1*, 59.3 Mb<br>*SPO11*, 59.4 Mb<br>*BMP7*, 59.4 Mb	ribonucleic acid export 1<br>SPO11 meiotic protein covalently bound to DSB<br>bone morphogenetic protein 7	age-associated infertility in mice (126), drosophila male meiosis and spermatogenesis (127)<br>infertility in male and female mice (128)<br>increases the expression of follicle-stimulating hormone (FSH) receptor in human granulosa cells (129)
13	76616107-77686707	*SULF2*<br>*ARFGEF2*<br>*CSE1L*, 77.9 Mb	sulfatase 2<br>ADP-ribosylation factor guanine nucleotide-exchange factor 2<br>CSE1 chromosome segregation 1-like	mouse neonatal survival (130)<br>early embryonic lethality (131)<br>essential for early embryonic growth and development in mice (132)

14	1463676-2138926	*HSF1*	heat shock transcription factor 1	embryo development beyond the zygotic stage (133)
14	33716508-35052708	*MYBL1*, 32.8 Mb	v-myb avian myeloblastosis viral oncogene homolog-like 1	master regulator of male meiosis (134)
14	35744766-36092385	*SULF1* at 35.5 Mb	sulfatase 1	mouse neonatal survival (130)
14	66118162-66771994	*SPAG1*	sperm associated antigen 1	present in spermatocytes and sperm, may participate in spermatogenesis and fertilization process (135)
14	68450192-68962221	*MTDH*	metadherin	male infertility in mice (136)
15	52214348-52660739	*TRPC2*	transient receptor potential cation channel, subfamily C, member 2	TRPC2-deficient mice exhibit striking behavioral defects in the regulation of sexual and social behaviors (137)
		STIM1, 51.9Mb	stromal interaction molecule 1	calcium signaling during early stages of fertilization (138)
15	74332484-74924833	*HSD17B12*	hydroxysteroid (17-beta) dehydrogenase 12	organogenesis and embryonic survival in mice (139)
		ALKBH2	alkB homolog 2, alpha-ketoglutarate-dependent dioxygenase	protects against lethality and mutation in primary mouse embryonic fibroblasts (140)
15	81964881-82260685	*PGR*	progesterone receptor-like	knockout mice infertile (141)
16	43618684-44459269	*TARDBP*, 43.5 Mb	TAR DNA binding protein	early embryogenesis in mice (142, 143)
		SRM, 43.4 Mb	spermidine synthase	spermine synthesis in mice (144)
		MTOR,43.3 Mb	mechanistic target of rapamycin	embryo implantation in mice (85)
		UBIAD1, 43.3 Mb	UbiA prenyltransferase domain containing 1	embryonic development in mice (71)
		MTHFR, 42.8 Mb	methylenetetrahydrofolate reductase (NAD(P)H)	male fertility (6) and embryo loss (145)
16	58405786-59021630	*PAPPA2* at 59.1 Mb	pappalysin 2	highly expressed in mouse and human placenta (146)
16	66009531-66543047	*SMG7*	SMG7 nonsense mediated mRNA decay factor	embryonic lethality in *Arabidopsis* (147)
		LAMC1, 65.6 Mb	laminin, gamma 1	embryonic lethality in mice (148)
		SHCBP1L, 65.4 Mb	SHC SH2-domain binding protein 1-	spermatogenesis in mammals (149)

			like	
17	38467666-39681407	*RAPGEF2*, 40.5 Mb	Rap guanine nucleotide exchange factor (GEF) 2	essential for embryonic hematopoiesis but dispensable for adult hematopoiesis (76)
17	54081495-54651938	*ATP6V0A2*<br><br>*TMED2*	ATPase, H+ transporting, lysosomal V0 subunit a2	semen quality in men (150) and link between multiple causes of spontaneous abortion in mice (151)<br>morphogenesis of the mouse embryo and placenta (152)
18	8467054-9606800	*HSD17B2*	hydroxysteroid (17-beta) dehydrogenase 2	controls the last step in the formation of all androgens and all estrogens (153), is associated with spermatogenic defect in human (154), and placenta defects and embryonic lethality in mice (139)
18	14115136-14626788	*SPATA33*	spermatogenesis associated 33	spermatogenesis in mice (155)
18	50581375-51369666	*BSP5*, 51.9 Mb	binder of sperm 5	related to bull fertility (86)
18	53790007-54112530	*NANOS2*	nanos homolog 2	suppresses meiosis and promotes male germ cell differentiation (156), is predominantly expressed in male germ cells, and the elimination of this gene results in a complete loss of spermatogonia in mice (157)
18	54556209-55028139	*ELSPBP1*	epididymal sperm binding protein 1	associated with dead semen in Holsteins (158)
18	55807264-56642741	*IZUMO1*<br>*FGF21*<br><br>*TSKS* (*TSKS1*)	izumo sperm-egg fusion 1<br>fibroblast growth factor 21<br><br>testis specific serine kinase substrate	required for sperm to fuse with eggs (159)<br>contributes to neuroendocrine control of female reproduction (160)<br>targeted deletion of Tssk1 and 2 causes male infertility due to haploinsufficiency (161)
19	20878990-21569741	*MYO18B*	myosin XVIIIB	embryonic lethality with cardiac myofibrillar aberrations (162)
19	27643677-28095023	*NEURL4*<br>*TMEM95*<br>*SPEM1*	neuralized E3 ubiquitin protein ligase 4<br>transmembrane protein 95<br>sperm maturation 1	germ cell formation and integrity in Drosophila (163)<br>subfertility in cattle (164)<br>aberrant cytoplasm removal, sperm deformation, and

		SHBG<br>*YBX2*, 27.6 Mb	sex hormone-binding globulin<br>Y box protein 2	male infertility in mice (165)<br>sperm concentration in men (166)<br>abnormal spermatogenesis in men (167)
19	34229311-34667494	*UBB*, 33.9 Mb<br>*LLGL1*, 35.0 Mb<br>*ALKBH5*, 35.0 Mb	ubiquitin B<br>lethal giant larvae homolog 1<br>AlkB family member 5, RNA demethylase	male and female infertility in Ubb-/- mice (167)<br>master regulator of male meiosis (134)<br>proper branching morphogenesis during placental development in mice(164)<br>RNA metabolism and mouse spermatogenesis (165)
19	52557638-53063966	*AATK*, 52.2 Mb	apoptosis-associated tyrosine kinase	spindle morphogenesis in male meiosis (134)
19	62805137-63646530	*PRKAR1A*, 62.3 Mb	protein kinase, cAMP-dependent, regulatory, type I, alpha	fertility defects in male mice and men (168)
20	9919863-10295382	*SMN2*	survival of motor neuron 2, centromeric	rescues embryonic lethality in mice (169)
20	26917749-27503083	*PELO*, 26.3 Mb	pelota homolog	early embryonic lethality and defects in cell cycle progression (170)
20	31393193-32030332	*CCL28*<br>*GHR*, 31.9 Mb	chemokine (C-C motif) ligand 28<br>growth hormone receptor	involvement in human miscarriage (14)<br>defects in reproductive function of female and male mice (171)
20	38453649-38606353	*SPEF2*	sperm flagellar 2	sperm tail development and male fertility (123)
20	38920878-39350230	*PRLR*	prolactin receptor	female fertility (52) and male fertility (172)
21	7070882-7637712	*CERS3,* 6.3 Mb<br>*IGF1R*, 8.0 Mb	ceramide synthase 3<br>insulin-like growth factor 1 receptor	Male fertility (173)<br>mouse testis size and daily sperm production (174)
21	17531298-18167162	*NTRK3*	neurotrophic tyrosine kinase, receptor, type 3	ovarian primordial to primary follicle transition (175)
21	56348332-57186819	*CATSPERB*<br>*CATSPER2*, 55.9 Mb	catsper channel auxiliary subunit beta<br>cation channel, sperm associated 2	part of sperm-specific ion channels (176)<br>human male infertility gene (177)
21	60851191-61333785	*DICER1*	dicer 1, ribonuclease type III	development of female reproductive system (178)
21	64129449-66380363	*VRK1*, 63.3 Mb	vaccinia related kinase 1	male infertility (179) and female infertility (180) in mice

21	69853542-70931906	*AKT1*	v-akt murine thymoma viral oncogene homolog 1	subfertility caused by altered follicular development and oocyte growth in female mice lacking KBalpha/AKT1 (181)
22	13172658-14654094	*CTNNB1*	catenin (cadherin-associated protein), beta 1, 88kDa	seminiferous tubule degeneration and infertility in mice (182)
22	50926015-51682391	*MST1*<br>*RBM5*<br><br><br>*SEMA3F*	macrophage stimulating 1<br>RNA binding motif protein 5<br><br><br>sema domain, immunoglobulin domain (Ig), short basic domain, secreted, (semaphorin) 3F	early embryonic development in mice (183)<br>a male germ cell splicing factor, required for spermatid differentiation and male fertility in mice(184)<br>dysregulation of *SEMA3F* leads to defective GnRH-1 cell migration, abnormal gonadal development and altered fertility (185)
23	25178791-27992880	*FKBP6*<br><br><br>*SKIV2L*<br>*HSPA1A*<br><br>*MSH5*<br><br><br><br><br><br>*POU5F1*	FK506 binding protein 6, 36kDa<br><br><br>superkiller viralicidic activity 2-like<br>heat shock 70kDa protein 1A<br><br>mutS homolog 5<br><br><br><br><br><br>POU domain, class 5, transcription factor 1	plays an essential role in mouse male fertility and homologous chromosome pairing in meiosis (186) and is implicated in sterility in male rats (187)<br>differentially expressed in mouse testis (188)<br>development of cleaved embryos to the blastocyst stage (189)<br>required for proper chromosome synapsis in male and female meiosis and is involved in spermatogenesis in mouse (190), is involved in infertility in Chinese males (191), maybe involved in premature ovarian failure in Caucasian women (192), and male and female mice with *MSH5*$^{-/-}$ genotype are sterile(108);<br>an essential transcription factor involved in regulating early embryonic development and cell differentiation (106), and in rabbit oocyte development (193)
23	30039692-31222894	*NKAPL*	NFKB activating protein-like	mouse spermatogenesis (194)
23	36509046-37321457	*SOX4*	SRY (sex determining region Y)-box 4	partially penetrant perinatal lethality in mice (195)

24	16118203-16306830	*PIK3C3*, 14.2 Mb	phosphatidylinositol 3-kinase, catalytic subunit type 3	early embryogenesis and cell proliferation in mice (196)
24	49190314-50568228	*SMAD4*	SMAD family member 4	ovarian-specific knockout mice are subfertile with decreasing fertility over time and multiple defects in folliculogenesis (197)
24	59564307-61140716	*BCL2,* 62.0 Mb	B-cell CLL/lymphoma 2	decreased numbers of oocytes and primordial follicles established in the post-natal female mouse gonad (198)
25	23825593-26337016	*NUPR1*, 26.3 Mb<br>*MAPK3*, 26.4 Mb<br>*KAT8*, 27.5 Mb	nuclear protein, transcriptional regulator, 1<br>mitogen-activated protein kinase 3<br>K(lysine) acetyltransferase 8	delayed ovarian maturation and testicular development in mice (199)<br>MAPK3/1 (ERK1/2) in ovarian granulosa cells are essential for female fertility (200)<br>essential for progression of embryonic development past the blastocyst stage and required for normal chromatin architecture (201)
26	19507012-20474308	*LOXL4,* 19.2 Mb	lysyl oxidase-like 4	increased risk for endometriosis and endometriosis-related infertility in a Puerto Rican population (202)
26	36056808-37203584	*GFRA1*	glial cell line derived neurotrophic factor family receptor alpha 1	mouse spermatognia (203)
27	32405436-33749694	*ASH2L*<br>*STAR*	ash2 (absent, small, or homeotic)-like<br>steroidogenic acute regulatory protein	required during early embryogenesis (204)<br>steroidogenesis including the ovarian and placental progestogens and estrogens, which regulate reproductive function and secondary sex characteristics in the female, and testicular androgens, which are essential for fertility and secondary sex characteristics in the male (205)
28	3637555-3998395	*FMN2*, 4.1 Mb	formin 2	polyploid embryo formation, recurrent pregnancy loss and sub-fertility (206)
28	9431460-9827919	*ZP4*, 10.7 Mb	zona pellucida glycoprotein 4	mouse folliculogenesis (207)
28	19436357-20480901	*JMJD1C*	jumonji domain containing 1C	required for long-term maintenance of male germ

				cells in mice (208)
29	19928179-20601858	*LUZP2 (MGC157332)*	leucine zipper protein 2	tyrosine phosphorylation in buffalo and cattle spermatozoa (88)
29	43328607-43839783	*EHD1*	EH-domain containing 1	spermatogenesis and male fertility in mice (209)
29	47422720-47690650	*FGF4*	fibroblast growth factor 4	protects male germ cells from apoptosis under heat-stress condition (210)
X	42024368-43999854	*PCDH11X*	protocadherin 11 X-linked	primary ovarian insufficiency in Dutch women (211)
X	47777883-50440169	*DIAPH2*	diaphanous-related formin 2	mutations disrupt oogenesis and spermatogenesis, supporting a role for DIAPH2 in ovarian function (100)
X	73035611-80134199 (2Mb or 3Mb sliding windows)	*DACH2*	dachshund family transcription factor 2	female reproductive tract development is severely disrupted in *DACH1/DACH2* double mutants (212), may have roles in premature ovarian failure in Italian women (213)
		POF1B	premature ovarian failure, 1B	disruption of POF1B binding to nonmuscle actin filaments is associated with premature ovarian failure (214)
		FGF16	fibroblast growth factor 16	required for embryonic heart development (215, 216)
X	88170589-88840217 (2Mb or 3Mb sliding windows)	*AR*	androgen receptor	crucial role in the development of male reproductive organs and needed for puberty, male fertility and male sexual function (217); long polyglutamine tracts in the androgen receptor are associated with reduced trans-activation, impaired sperm production, and male infertility (218); age-dependent defects that resemble the *AR* null phenotype of dysfunctional late follicle development, ovulation and fertility in mice (219)
X	93574264- 94321737	*BMP15*	bone morphogenetic protein 15	hypergonadotropic ovarian failure in women (220), premature ovarian failure in women (221-223), expressed exclusively in the oocyte in humans, rodents and sheep (224), and is associated with

				ovulation rate and infertility (222, 225-227)
X	109945924-112346433	*RPGR*	retinitis pigmentosa GTPase regulator	overexpression of *RPGR* leads to male infertility in mice due to defects in flagellar assembly (188)
		DYNLT3	dynein, light chain, Tctex-type 3	required for chromosome alignment during mouse oocyte meiotic maturation (228)

References

1. Takahashi Y*, et al.* (2002) Mammalian copper chaperone Cox17p has an essential role in activation of cytochrome C oxidase and embryonic development. *Molecular and cellular biology* 22(21):7614-7621.
2. Kelberman D*, et al.* (2006) Mutations within Sox2/SOX2 are associated with abnormalities in the hypothalamo-pituitary-gonadal axis in mice and humans. *Journal of Clinical Investigation* 116(9):2442.
3. Shin H*, et al.* (2014) The role of Pik3ca in uterine gland morphogenesis and fertility in mice. *Cancer Research* 74(19 Supplement):94-94.
4. Dam AH*, et al.* (2007) Homozygous mutation in SPATA16 is associated with male infertility in human globozoospermia. *The American Journal of Human Genetics* 81(4):813-820.
5. Chabory E*, et al.* (2009) Epididymis seleno-independent glutathione peroxidase 5 maintains sperm DNA integrity in mice. *The Journal of clinical investigation* 119(7):2074.
6. Singh K, Singh S, & Raman R (2010) MTHFR A1298C polymorphism and idiopathic male infertility. *Journal of postgraduate medicine* 56(4):267-269.
7. McClure MC*, et al.* (2014) Bovine exome sequence analysis and targeted SNP genotyping of recessive fertility defects BH1, HH2, and HH3 reveal a putative causative mutation in SMC2 for HH3. *PLoS One* 9(3):e92769.
8. Hoyt PR*, et al.* (1997) The Evil proto-oncogene is required at midgestation for neural, heart, and paraxial mesenchyme development. *Mechanisms of development* 65(1):55-70.
9. Wisniewski H-G & Vilček J (2004) Cytokine-induced gene expression at the crossroads of innate immunity, inflammation and fertility: TSG-6 and PTX3/TSG-14. *Cytokine & growth factor reviews* 15(2):129-146.
10. Moreno-Garcia MA, Pupavac M, Rosenblatt DS, Tremblay ML, & Jerome-Majewska LA (2014) The Mmachc gene is required for pre-implantation embryogenesis in the mouse. *Molecular genetics and metabolism* 112(3):198-204.
11. Kong B*, et al.* (2014) Maternally-derived zinc transporters ZIP6 and ZIP10 drive the mammalian oocyte-to-egg transition. *Molecular human reproduction* 20(11):1077-1089.
12. Croxford TP, McCormick NH, & Kelleher SL (2011) Moderate zinc deficiency reduces testicular Zip6 and Zip10 abundance and impairs spermatogenesis in mice. *The Journal of nutrition* 141(3):359-365.
13. Ueda Y*, et al.* (2007) PGAP1 knock-out mice show otocephaly and male infertility. *Journal Of Biological Chemistry* 282(42):30373-30380.
14. Marchiani S*, et al.* (2011) Sumo1-ylation of human spermatozoa and its relationship with semen quality. *International journal of andrology* 34(6pt1):581-593.
15. Nagashima T*, et al.* (2013) BMPR2 is required for postimplantation uterine function and pregnancy maintenance. *The Journal of clinical investigation* 123(6):2539.
16. Liu S*, et al.* (2013) Costimulatory Molecule CD28 Participates in the Process of Embryo Implantation in Mice. *Reproductive Sciences*:1933719113512537.
17. Zhang Z*, et al.* (2006) Deficiency of SPAG16L causes male infertility associated with impaired sperm motility. *Biology of reproduction* 74(4):751-759.
18. Wu Q, Song R, & Yan W (2010) SPATA3 and SPATA6 Interact with KLHL10 and Participate in Spermatogenesis. *Biology of reproduction* 83(1 Supplement):177.

19. López-Contreras AJ, *et al.* (2009) Expression of antizyme inhibitor 2 in male haploid germinal cells suggests a role in spermiogenesis. *The international journal of biochemistry & cell biology* 41(5):1070-1078.
20. Ward WS (2012) Pum 1 sequesters apoptosis during spermatogenesis. *Asian journal of andrology* 14(4):513.
21. Mak W, Chen D, Uyhazi K, & Lin H (2013) The role of Pumilio 1, a translational regulator, in the mammalian female germline. *Fertility and sterility* 100(3):S54.
22. Chen D, *et al.* (2012) Pumilio 1 suppresses multiple activators of p53 to safeguard spermatogenesis. *Current Biology* 22(5):420-425.
23. Tarazona R, *et al.* (2011) Human prostasomes express CD48 and interfere with NK cell function. *Immunobiology* 216(1):41-46.
24. Zhang H, *et al.* (2015) The homologous genes Vangl1 and Vangl2 are required for embryo implantation in the uterus of mice during early pregnancy. *Gene* 555(2):140-149.
25. Jimenez T, McDermott JP, Sánchez G, & Blanco G (2011) Na, K-ATPase α4 isoform is essential for sperm fertility. *Proceedings of the National Academy of Sciences* 108(2):644-649.
26. Wang G, *et al.* (2004) Essential requirement for both hsf1 and hsf2 transcriptional activity in spermatogenesis and male fertility. *Genesis* 38(2):66-80.
27. Korfanty J, *et al.* (2014) Crosstalk between HSF1 and HSF2 during the heat shock response in mouse testes. *The international journal of biochemistry & cell biology* 57:76-83.
28. Guiraldelli MF, Eyster C, Wilkerson JL, Dresser ME, & Pezza RJ (2013) Mouse HFM1/Mer3 is required for crossover formation and complete synapsis of homologous chromosomes during meiosis. *PLoS Genet* 9(3):e1003383.
29. Tse E, *et al.* (2004) Null mutation of the Lmo4 gene or a combined null mutation of the Lmo1/Lmo3 genes causes perinatal lethality, and Lmo4 controls neural tube development in mice. *Molecular and cellular biology* 24(5):2063-2073.
30. Amaral A, *et al.* (2014) Identification of Proteins Involved in Human Sperm Motility Using High-Throughput Differential Proteomics. *Journal of proteome research*.
31. Sun S-C, *et al.* (2013) Actin nucleator Arp2/3 complex is essential for mouse preimplantation embryo development. *Reproduction, Fertility and Development* 25(4):617-623.
32. Jin S-LC, Richard FJ, Kuo W-P, D'Ercole AJ, & Conti M (1999) Impaired growth and fertility of cAMP-specific phosphodiesterase PDE4D-deficient mice. *Proceedings of the National Academy of Sciences* 96(21):11998-12003.
33. Burnicka-Turek O, *et al.* (2012) INSL5-deficient mice display an alteration in glucose homeostasis and an impaired fertility. *Endocrinology* 153(10):4655-4665.
34. Lee A & Ing N (2007) Expression of 24 dehydrocholesterol reductase and exonuclease nef genes in testicular tissues of stallions. *Biology of reproduction* 77(1 Supplement):121.
35. Kuwahara S, *et al.* (2006) PSPC1, NONO, and SFPQ are expressed in mouse Sertoli cells and may function as coregulators of androgen receptor-mediated transcription. *Biology of reproduction* 75(3):352-359.
36. Modzelewski AJ, Holmes RJ, Hilz S, Grimson A, & Cohen PE (2012) AGO4 regulates entry into meiosis and influences silencing of sex chromosomes in the male mouse germline. *Developmental cell* 23(2):251-264.
37. Iguchi N, *et al.* (1999) Molecular cloning of haploid germ cell-specific tektin cDNA and analysis of the protein in mouse testis. *FEBS letters* 456(2):315-321.

38. Tanaka H*, et al.* (2004) Mice deficient in the axonemal protein Tektin-t exhibit male infertility and immotile-cilium syndrome due to impaired inner arm dynein function. *Molecular and cellular biology* 24(18):7958-7964.
39. McGuire MM*, et al.* (2011) Genomic analysis using high-resolution single-nucleotide polymorphism arrays reveals novel microdeletions associated with premature ovarian failure. *Fertility and sterility* 95(5):1595-1600.
40. Lu B*, et al.* (2008) A mutation in the inner mitochondrial membrane peptidase 2-like gene (Immp2l) affects mitochondrial function and impairs fertility in mice. *Biology of reproduction* 78(4):601-610.
41. Elliott MR*, et al.* (2010) Unexpected requirement for ELMO1 in clearance of apoptotic germ cells in vivo. *Nature* 467(7313):333-337.
42. Chao H-CA*, et al.* (2010) The expression pattern of SEPT7 correlates with sperm morphology. *Journal of assisted reproduction and genetics* 27(6):299-307.
43. Harbuz R*, et al.* (2011) A recurrent deletion of DPY19L2 causes infertility in man by blocking sperm head elongation and acrosome formation. *The American Journal of Human Genetics* 88(3):351-361.
44. Koscinski I*, et al.* (2011) DPY19L2 deletion as a major cause of globozoospermia. *The American Journal of Human Genetics* 88(3):344-350.
45. Zhu F, Gong F, Lin G, & Lu G (2013) DPY19L2 gene mutations are a major cause of globozoospermia: identification of three novel point mutations. *Molecular human reproduction*:gat018.
46. Pierre V*, et al.* (2012) Absence of Dpy19l2, a new inner nuclear membrane protein, causes globozoospermia in mice by preventing the anchoring of the acrosome to the nucleus. *Development* 139(16):2955-2965.
47. Fowler D, Nicolaides K, & Miell J (2000) Insulin-like growth factor binding protein-1 (IGFBP-1): a multifunctional role in the human female reproductive tract. *Human reproduction update* 6(5):495-504.
48. Wang T-H*, et al.* (2006) Insulin-like growth factor-II (IGF-II), IGF-binding protein-3 (IGFBP-3), and IGFBP-4 in follicular fluid are associated with oocyte maturation and embryo development. *Fertility and sterility* 86(5):1392-1401.
49. Di Renzo F*, et al.* (2006) The murine Pou6f2 gene is temporally and spatially regulated during kidney embryogenesis and its human homolog is overexpressed in a subset of Wilms tumors. *Journal of pediatric hematology/oncology* 28(12):791-797.
50. La Sala G*, et al.* (2015) Modulation of Dhh signaling and altered Sertoli cell function in mice lacking the GPR37-prosaposin receptor. *The FASEB Journal* 29(5):2059-2069.
51. Shen C*, et al.* (2013) Prss37 is required for male fertility in the mouse. *Biology of reproduction* 88(5):123.
52. Matzuk MM & Lamb DJ (2002) Genetic dissection of mammalian fertility pathways. *Translocations* 45:46XY.
53. Ryu BJ, Kim HR, Jeong JK, & Lee BJ (2011) Regulation of the female rat estrous cycle by a neural cell-specific epidermal growth factor-like repeat domain containing protein, NELL2. *Molecules and cells* 32(2):203-207.
54. Vilagran I*, et al.* (2013) Acrosin-binding protein (ACRBP) and triosephosphate isomerase (TPI) are good markers to predict boar sperm freezing capacity. *Theriogenology* 80(5):443-450.
55. Kaji K*, et al.* (2000) The gamete fusion process is defective in eggs of Cd9-deficient mice. *Nature genetics* 24(3):279-282.

56. Kaji K, Oda S, Miyazaki S, & Kudo A (2002) Infertility of CD9-deficient mouse eggs is reversed by mouse CD9, human CD9, or mouse CD81; polyadenylated mRNA injection developed for molecular analysis of sperm–egg fusion. *Developmental biology* 247(2):327-334.
57. Le Naour F, Rubinstein E, Jasmin C, Prenant M, & Boucheix C (2000) Severely reduced female fertility in CD9-deficient mice. *Science* 287(5451):319-321.
58. Miller BJ, Georges-Labouesse E, Primakoff P, & Myles DG (2000) Normal fertilization occurs with eggs lacking the integrin α6β1 and is CD9-dependent. *The Journal of cell biology* 149(6):1289-1296.
59. Miyado K*, et al.* (2000) Requirement of CD9 on the egg plasma membrane for fertilization. *Science* 287(5451):321-324.
60. Cormier S, Leroy C, Delezoide A-L, & Silve C (2005) Expression of fibroblast growth factors 18 and 23 during human embryonic and fetal development. *Gene expression patterns* 5(4):569-573.
61. Meyer-Ficca ML*, et al.* (2015) Spermatid head elongation with normal nuclear shaping requires ADP-ribosyltransferase PARP11 (ARTD11) in mice. *Biology of reproduction* 92(3):80, 81-13.
62. Dickinson RE*, et al.* (2010) Involvement of the SLIT/ROBO pathway in follicle development in the fetal ovary. *Reproduction* 139(2):395-407.
63. Jiang Z, Gibson JP, Archibald AL, & Haley CS (2001) The porcine gonadotropin-releasing hormone receptor gene (GNRHR): genomic organization, polymorphisms, and association with the number of corpora lutea. *Genome* 44(1):7-12.
64. Stewart MD*, et al.* (2012) Mice harboring Gnrhr E90K, a mutation that causes protein misfolding and hypogonadotropic hypogonadism in humans, exhibit testis size reduction and ovulation failure. *Molecular Endocrinology* 26(11):1847-1856.
65. De Mees C*, et al.* (2006) Alpha-fetoprotein controls female fertility and prenatal development of the gonadotropin-releasing hormone pathway through an antiestrogenic action. *Molecular and cellular biology* 26(5):2012-2018.
66. Momb J*, et al.* (2013) Deletion of Mthfd1l causes embryonic lethality and neural tube and craniofacial defects in mice. *Proceedings of the National Academy of Sciences* 110(2):549-554.
67. Philibert C, Bouillot S, Huber P, & Faury G (2012) Protocadherin-12 deficiency leads to modifications in the structure and function of arteries in mice. *Pathologie Biologie* 60(1):34-40.
68. Anonymous (FGF1. http://www.ncbi.nlm.nih.gov/gene/2246.
69. Wang H-X*, et al.* (2005) Expression of adamalysin 19/ADAM19 in the endometrium and placenta of rhesus monkey (Macaca mulatta) during early pregnancy. *Molecular human reproduction* 11(6):429-435.
70. Han F*, et al.* (2014) Epigenetic regulation of Sox30 is associated with testis development in mice.
71. Nakagawa K*, et al.* (2014) Vitamin K2 Biosynthetic Enzyme, UBIAD1 Is Essential for Embryonic Development of Mice. *PloS one* 9(8):e104078.
72. Ronfani L*, et al.* (2001) Reduced fertility and spermatogenesis defects in mice lacking chromosomal protein Hmgb2. *Development* 128(8):1265-1273.
73. Sutherland HG*, et al.* (2006) Disruption of Ledgf/Psip1 results in perinatal mortality and homeotic skeletal transformations. *Molecular and cellular biology* 26(19):7201-7210.

74. Salker MS, *et al.* (2012) Disordered IL-33/ST2 activation in decidualizing stromal cells prolongs uterine receptivity in women with recurrent pregnancy loss. *PloS one* 7(12):e52252.
75. Garcia-Rudaz C, *et al.* (2007) Fxna, a novel gene differentially expressed in the rat ovary at the time of folliculogenesis, is required for normal ovarian histogenesis. *Development* 134(5):945-957.
76. Calounova G, *et al.* (2010) The Src homology 2 domain-containing adapter protein B (SHB) regulates mouse oocyte maturation.
77. Kerr B, Garcia-Rudaz C, Dorfman M, Paredes A, & Ojeda SR (2009) NTRK1 and NTRK2 receptors facilitate follicle assembly and early follicular development in the mouse ovary. *Reproduction* 138(1):131-140.
78. Dorfman MD, *et al.* (2014) Loss of Ntrk2/Kiss1r signaling in oocytes causes premature ovarian failure.
79. Kim N, *et al.* (2011) Abnormal sperm development in pcd 3J-/-mice: the importance of Agtpbp1 in spermatogenesis. *Molecules and cells* 31(1):39-48.
80. Koomen M, *et al.* (2002) Reduced fertility and hypersensitivity to mitomycin C characterize Fancg/Xrcc9 null mice. *Human molecular genetics* 11(3):273-281.
81. Svacinova V, *et al.* (2011) Sequence recombination in exon 1 of the TSPY gene in men with impaired fertility. *Biomedical Papers* 155(3):287-298.
82. Anonymous (2015) CYLC2. *http://www.ncbi.nlm.nih.gov/gene/1539*.
83. Wittschieben J, *et al.* (2000) Disruption of the developmentally regulated Rev3l gene causes embryonic lethality. *Current Biology* 10(19):1217-1220.
84. Wu M, Dumalska I, Morozova E, van den Pol A, & Alreja M (2009) Melanin-concentrating hormone directly inhibits GnRH neurons and blocks kisspeptin activation, linking energy balance to reproduction. *Proceedings of the National Academy of Sciences* 106(40):17217-17222.
85. Chen X, *et al.* (2009) The role of MTOR in mouse uterus during embryo implantation. *Reproduction* 138(2):351-356.
86. Mishra C, Palai TK, Sarangi LN, Prusty BR, & Maharana BR (2013) Candidate gene markers for sperm quality and fertility in bulls. *Veterinary World* 6(11):905-910.
87. Sapiro R, *et al.* (2002) Male infertility, impaired sperm motility, and hydrocephalus in mice deficient in sperm-associated antigen 6. *Molecular and cellular biology* 22(17):6298-6305.
88. Byrne K, Leahy T, McCulloch R, Colgrave ML, & Holland MK (2012) Comprehensive mapping of the bull sperm surface proteome. *Proteomics* 12(23-24):3559-3579.
89. Ohashi K, *et al.* (2003) Early embryonic lethality caused by targeted disruption of the 3-hydroxy-3-methylglutaryl-CoA reductase gene. *Journal Of Biological Chemistry* 278(44):42936-42941.
90. Wang X, *et al.* (2014) Tssk4 is essential for maintaining the structural integrity of sperm flagellum. *Molecular human reproduction*:gau097.
91. Hou W, *et al.* (2010) Altered expression of NDRG2 in the testes of experimental rat model of cryptorchidism. *Urology* 75(4):985-991.
92. Tal R, Seifer D, Shohat-Tal A, Malter H, & Grazi R (2013) Angiopoietin-2 is increased in follicular fluid of polycystic ovarian syndrome (PCOS) women during controlled ovarian stimulation and correlates with number of oocytes retrieved. *Fertility and sterility* 100(3):S360.

93. McKenzie L*, et al.* (2004) Human cumulus granulosa cell gene expression: a predictor of fertilization and embryo selection in women undergoing IVF. *Human Reproduction* 19(12):2869-2874.

94. Jindal S, Greenseid K, Berger D, Santoro N, & Pal L (2012) Impaired Gremlin 1 (GREM1) expression in cumulus cells in young women with diminished ovarian reserve (DOR). *Journal of assisted reproduction and genetics* 29(2):159-162.

95. Lu Q*, et al.* (1999) Tyro-3 family receptors are essential regulators of mammalian spermatogenesis. *Nature* 398(6729):723-728.

96. Pierce A*, et al.* (2008) Axl and Tyro3 modulate female reproduction by influencing gonadotropin-releasing hormone neuron survival and migration. *Molecular Endocrinology* 22(11):2481-2495.

97. Busso D, Onate-Alvarado M, Balboa E, Zanlungo S, & Moreno R (2010) Female infertility due to anovulation and defective steroidogenesis in NPC2 deficient mice. *Molecular and cellular endocrinology* 315(1):299-307.

98. Busso D*, et al.* (2014) Spermatozoa from mice deficient in Niemann-Pick disease type C2 (NPC2) protein have defective cholesterol content and reduced in vitro fertilising ability. *Reproduction, Fertility and Development* 26(4):609-621.

99. Santi A*, et al.* (2011) Increased endometrial placenta growth factor (PLGF) gene expression in women with successful implantation. *Fertility and sterility* 96(3):663-668.

100. Welt CK (2008) Primary ovarian insufficiency: a more accurate term for premature ovarian failure. *Clinical endocrinology* 68(4):499-509.

101. Lipkin SM*, et al.* (2002) Meiotic arrest and aneuploidy in MLH3-deficient mice. *Nature genetics* 31(4):385-390.

102. Lee G-S*, et al.* (2013) Disruption of Ttll5/stamp gene (tubulin tyrosine ligase-like protein 5/SRC-1 and TIF2-associated modulatory protein gene) in male mice causes sperm malformation and infertility. *Journal Of Biological Chemistry* 288(21):15167-15180.

103. Yu J*, et al.* (2015) Identification of seven genes essential for male fertility through a genome-wide association study of non-obstructive azoospermia and RNA interference-mediated large-scale functional screening in Drosophila. *Human molecular genetics* 24(5):1493-1503.

104. Salilew-Wondim D*, et al.* (2010) Depletion of BIRC6 leads to retarded bovine early embryonic development and blastocyst formation in vitro. *Reproduction, Fertility and Development* 22(3):564-579.

105. Gruber M, Mathew LK, Runge AC, Garcia JA, & Simon MC (2010) EPAS1 is required for spermatogenesis in the postnatal mouse testis. *Biology of reproduction* 82(6):1227-1236.

106. Arnhold IJ, Lofrano-Porto A, & Latronico AC (2008) Inactivating mutations of luteinizing hormone beta-subunit or luteinizing hormone receptor cause oligo-amenorrhea and infertility in women. *Hormone research* 71(2):75-82.

107. Simoni M*, et al.* (2008) Polymorphisms of the luteinizing hormone/chorionic gonadotropin receptor gene: association with maldescended testes and male infertility. *Pharmacogenetics and genomics* 18(3):193-200.

108. Hastings N, Donn S, Derecka K, Flint A, & Woolliams J (2006) Polymorphisms within the coding region of the bovine luteinizing hormone receptor gene and their association with fertility traits. *Animal genetics* 37(6):583-585.

109. Layman LC (1999) Mutations in the follicle-stimulating hormone-beta (FSH beta) and FSH receptor genes in mice and humans. *Seminars in reproductive medicine*, pp 5-10.

110. Lu B & Bishop CE (2003) Late onset of spermatogenesis and gain of fertility in POG-deficient mice indicate that POG is not necessary for the proliferation of spermatogonia. *Biology of reproduction* 69(1):161-168.
111. Lv P*, et al.* (2015) Targeted disruption of Rab10 causes early embryonic lethality. *Protein & cell* 6(6):463.
112. Verver D, van Pelt AM, Repping S, & Hamer G (2013) Role for rodent Smc6 in pericentromeric heterochromatin domains during spermatogonial differentiation and meiosis. *Cell death & disease* 4(8):e749.
113. Chung J, Pask A, Yu H, & Renfree M (2011) Fibroblast growth factor-9 in marsupial testicular development. *Sexual Development* 5(3):131-140.
114. Lit LC*, et al.* (2013) LATS2 is a modulator of estrogen receptor alpha. *Anticancer research* 33(1):53-63.
115. McPherson JP*, et al.* (2004) Lats2/Kpm is required for embryonic development, proliferation control and genomic integrity. *The EMBO journal* 23(18):3677-3688.
116. Pan J*, et al.* (2005) RNF17, a component of the mammalian germ cell nuage, is essential for spermiogenesis. *Development* 132(18):4029-4039.
117. Mtango NR*, et al.* (2012) Essential role of maternal UCHL1 and UCHL3 in fertilization and preimplantation embryo development. *Journal of cellular physiology* 227(4):1592-1603.
118. Anonymous (The National Center for Biotechnology Information. https://www.ncbi.nlm.nih.gov/gene/6736.
119. Hassani F*, et al.* (2013) The effects of ISM1 medium on embryo quality and outcomes of IVF/ICSI cycles. *International journal of fertility & sterility* 7(2):108.
120. Xella S*, et al.* (2010) Embryo quality and implantation rate in two different culture media: ISM1 versus Universal IVF Medium. *Fertility and sterility* 93(6):1859-1863.
121. Maretto S*, et al.* (2008) Ventral closure, headfold fusion and definitive endoderm migration defects in mouse embryos lacking the fibronectin leucine-rich transmembrane protein FLRT3. *Developmental biology* 318(1):184-193.
122. Ueno H, Huang X, Tanaka Y, & Hirokawa N (2011) KIF16B/Rab14 molecular motor complex is critical for early embryonic development by transporting FGF receptor. *Developmental cell* 20(1):60-71.
123. Sironen A*, et al.* (2010) Expression of SPEF2 during mouse spermatogenesis and identification of IFT20 as an interacting protein. *Biology of reproduction* 82(3):580-590.
124. Fan J*, et al.* (2007) Male germ cell-specific expression of a novel Patched-domain containing gene Ptchd3. *Biochemical and biophysical research communications* 363(3):757-761.
125. Lee KY*, et al.* (2007) Bmp2 is critical for the murine uterine decidual response. *Molecular and cellular biology* 27(15):5468-5478.
126. Baker DJ*, et al.* (2004) BubR1 insufficiency causes early onset of aging-associated phenotypes and infertility in mice. *Nature genetics* 36(7):744-749.
127. Volpi S, Bongiorni S, Fabbretti F, Wakimoto BT, & Prantera G (2013) Drosophila rae1 is required for male meiosis and spermatogenesis. *Journal of cell science* 126(16):3541-3551.
128. Romanienko PJ & Camerini-Otero RD (2000) The mouse Spo11 gene is required for meiotic chromosome synapsis. *Molecular cell* 6(5):975-987.

129. Shi J*, et al.* (2010) Bone morphogenetic protein 7 (BMP-7) increases the expression of follicle-stimulating hormone (FSH) receptor in human granulosa cells. *Fertility and sterility* 93(4):1273-1279.
130. Holst CR*, et al.* (2007) Secreted sulfatases Sulf1 and Sulf2 have overlapping yet essential roles in mouse neonatal survival. *PloS one* 2(6):e575-e575.
131. Grzmil P*, et al.* (2010) Early embryonic lethality in gene trap mice with disruption of the Arfgef2 gene. *International Journal of Developmental Biology* 54(8):1259.
132. Bera TK, Bera J, Brinkmann U, Tessarollo L, & Pastan I (2001) Cse1l is essential for early embryonic growth and development. *Molecular and cellular biology* 21(20):7020-7024.
133. Christians E, Davis A, Thomas S, & Benjamin I (2000) Embryonic development: maternal effect of Hsf1 on reproductive success. *Nature* 407(6805):693-694.
134. Bolcun-Filas E*, et al.* (2011) A-MYB (MYBL1) transcription factor is a master regulator of male meiosis. *Development* 138(15):3319-3330.
135. Liu N*, et al.* (2005) A sperm component, HSD-3.8 (SPAG1), interacts with G-protein beta 1 subunit and activates extracellular signal-regulated kinases (ERK). *Frontiers in bioscience: a journal and virtual library* 11:1679-1689.
136. Shang P*, et al.* (2010) Functional transformation of the chromatoid body in mouse spermatids requires testis-specific serine/threonine kinases. *Journal of cell science* 123(3):331-339.
137. Zufall F (2005) The TRPC2 ion channel and pheromone sensing in the accessory olfactory system. *Naunyn-Schmiedeberg's archives of pharmacology* 371(4):245-250.
138. Gómez-Fernández C*, et al.* (2009) Relocalization of STIM1 in mouse oocytes at fertilization: early involvement of store-operated calcium entry. *Reproduction* 138(2):211-221.
139. Rantakari P*, et al.* (2008) Placenta Defects and Embryonic Lethality Resulting from Disruption of Mouse Hydroxysteroid (17-β) Dehydrogenase 2 Gene. *Molecular Endocrinology* 22(3):665-675.
140. Nay SL, Lee D-H, Bates SE, & O'Connor TR (2012) Alkbh2 protects against lethality and mutation in primary mouse embryonic fibroblasts. *DNA repair* 11(5):502-510.
141. Robker R*, et al.* (2000) Progesterone-regulated genes in the ovulation process: ADAMTS-1 and cathepsin L proteases. *Proceedings of the National Academy of Sciences* 97(9):4689-4694.
142. Wu LS*, et al.* (2010) TDP-43, a neuro-pathosignature factor, is essential for early mouse embryogenesis. *Genesis* 48(1):56-62.
143. Sephton CF*, et al.* (2010) TDP-43 is a developmentally regulated protein essential for early embryonic development. *Journal Of Biological Chemistry* 285(9):6826-6834.
144. Lorenz B*, et al.* (1998) Spermine deficiency in Gy mice caused by deletion of the spermine synthase gene. *Human molecular genetics* 7(3):541-547.
145. Callejón G*, et al.* (2007) Genotypes of the C677T and A1298C polymorphisms of the MTHFR gene as a cause of human spontaneous embryo loss. *Human Reproduction* 22(12):3249-3254.
146. Wang J*, et al.* (2009) Expression of pregnancy-associated plasma protein A2 during pregnancy in human and mouse. *Journal of Endocrinology* 202(3):337-345.
147. Riehs N*, et al.* (2008) Arabidopsis SMG7 protein is required for exit from meiosis. *Journal of cell science* 121(13):2208-2216.

148. Smyth N, *et al.* (1999) Absence of basement membranes after targeting the LAMC1 gene results in embryonic lethality due to failure of endoderm differentiation. *The Journal of cell biology* 144(1):151-160.
149. Liu M, *et al.* (2014) SHCBP1L, a conserved protein in mammals, is predominantly expressed in male germ cells and maintains spindle stability during meiosis in testis. *Molecular human reproduction* 20(6):463-475.
150. Ota K, *et al.* (2013) Expression of a2 vacuolar ATPase in spermatozoa is associated with semen quality and chemokine-cytokine profiles in infertile men. *PloS one* 8(7):e70470.
151. Jaiswal MK, Gilman-Sachs A, Chaouat G, & Beaman KD (2011) Placental ATPase expression is a link between multiple causes of spontaneous abortion in mice. *Biology of reproduction* 85(3):626-634.
152. Jerome-Majewska LA, Achkar T, Luo L, Lupu F, & Lacy E (2010) The trafficking protein Tmed2/p24β 1 is required for morphogenesis of the mouse embryo and placenta. *Developmental biology* 341(1):154-166.
153. Labrie F, *et al.* (1997) The key role of 17β-hydroxysteroid dehydrogenases in sex steroid biology. *Steroids* 62(1):148-158.
154. Hurst BS, *et al.* (2014) Molecular evaluation of proliferative-phase endometrium may provide insight about the underlying causes of infertility in women with endometriosis. *Archives of gynecology and obstetrics* 289(5):1119-1124.
155. Chen H, Yi M, Sheng Y, Cheng H, & Zhou R (2013) A Novel Testis-Enriched Gene Spata33 Is Expressed during Spermatogenesis.
156. Suzuki A & Saga Y (2008) Nanos2 suppresses meiosis and promotes male germ cell differentiation. *Genes & development* 22(4):430-435.
157. Tsuda M, *et al.* (2003) Conserved role of nanos proteins in germ cell development. *Science* 301(5637):1239-1241.
158. D'Amours O, *et al.* (2012) Binder of sperm 1 and epididymal sperm binding protein 1 are associated with different bull sperm subpopulations. *Reproduction* 143(6):759-771.
159. Inoue N, Ikawa M, Isotani A, & Okabe M (2005) The immunoglobulin superfamily protein Izumo is required for sperm to fuse with eggs. *Nature* 434(7030):234.
160. Owen BM, *et al.* (2013) FGF21 contributes to neuroendocrine control of female reproduction. *Nature medicine* 19(9):1153.
161. Xu B, *et al.* (2008) Targeted deletion of Tssk1 and 2 causes male infertility due to haploinsufficiency. *Developmental biology* 319(2):211-222.
162. Ajima R, *et al.* (2008) Deficiency of Myo18B in mice results in embryonic lethality with cardiac myofibrillar aberrations. *Genes to cells* 13(10):987-999.
163. Nagarkatti-Gude DR (2012) SPAG16 is a Bifunctional Gene Regulating Male Fertility.
164. Sripathy S, Lee M, & Vasioukhin V (2011) Mammalian Llgl2 is necessary for proper branching morphogenesis during placental development. *Molecular and cellular biology* 31(14):2920-2933.
165. Zheng G, *et al.* (2013) ALKBH5 is a mammalian RNA demethylase that impacts RNA metabolism and mouse fertility. *Molecular cell* 49(1):18-29.
166. Sinnar SA, *et al.* (2011) Altered testicular gene expression patterns in mice lacking the polyubiquitin gene Ubb. *Molecular reproduction and development* 78(6):415-425.
167. Ryu K-Y, *et al.* (2008) The mouse polyubiquitin gene Ubb is essential for meiotic progression. *Molecular and cellular biology* 28(3):1136-1146.
168. Burton KA, *et al.* (2006) Haploinsufficiency at the Protein Kinase A RIα gene locus leads to fertility defects in male mice and men. *Molecular Endocrinology* 20(10):2504-2513.

169. Monani UR*, et al.* (2000) The human centromeric survival motor neuron gene (SMN2) rescues embryonic lethality in Smn–/–mice and results in a mouse with spinal muscular atrophy. *Human molecular genetics* 9(3):333-339.
170. Adham IM*, et al.* (2003) Disruption of the pelota gene causes early embryonic lethality and defects in cell cycle progression. *Molecular and cellular biology* 23(4):1470-1476.
171. List EO*, et al.* (2010) Endocrine parameters and phenotypes of the growth hormone receptor gene disrupted (GHR−/−) mouse. *Endocrine reviews* 32(3):356-386.
172. Lin C*, et al.* (2006) Candidate gene markers for sperm quality and fertility of boar. *Animal reproduction science* 92(3):349-363.
173. Rabionet M*, et al.* (2015) Male meiotic cytokinesis requires ceramide synthase 3-dependent sphingolipids with unique membrane anchors. *Human molecular genetics* 24(17):4792-4808.
174. Pitetti J-L*, et al.* (2013) An essential role for insulin and IGF1 receptors in regulating sertoli cell proliferation, testis size, and FSH action in mice. *Molecular Endocrinology* 27(5):814-827.
175. Nilsson E, Dole G, & Skinner MK (2009) Neurotrophin NT3 promotes ovarian primordial to primary follicle transition. *Reproduction* 138(4):697-707.
176. Wang H, Liu J, Cho K-H, & Ren D (2009) A novel, single, transmembrane protein CATSPERG is associated with CATSPER1 channel protein. *Biology of reproduction* 81(3):539-544.
177. Avidan N*, et al.* (2003) CATSPER2, a human autosomal nonsyndromic male infertility gene. *European journal of human genetics* 11(7):497-502.
178. Hong X, Luense LJ, McGinnis LK, Nothnick WB, & Christenson LK (2008) Dicer1 is essential for female fertility and normal development of the female reproductive system. *Endocrinology* 149(12):6207-6212.
179. Wiebe MS, Nichols RJ, Molitor TP, Lindgren JK, & Traktman P (2010) Mice deficient in the serine/threonine protein kinase VRK1 are infertile due to a progressive loss of spermatogonia. *Biology of reproduction* 82(1):182-193.
180. Schober CS, Aydiner F, Booth CJ, Seli E, & Reinke V (2011) The kinase VRK1 is required for normal meiotic progression in mammalian oogenesis. *Mechanisms of development* 128(3):178-190.
181. Brown C*, et al.* (2010) Subfertility caused by altered follicular development and oocyte growth in female mice lacking PKBalpha/Akt1. *Biology of reproduction* 82(2):246-256.
182. Boyer A, Hermo L, Paquet M, Robaire B, & Boerboom D (2008) Seminiferous tubule degeneration and infertility in mice with sustained activation of WNT/CTNNB1 signaling in sertoli cells. *Biology of reproduction* 79(3):475-485.
183. Oh S*, et al.* (2009) Crucial role for Mst1 and Mst2 kinases in early embryonic development of the mouse. *Molecular and cellular biology* 29(23):6309-6320.
184. O'Bryan MK*, et al.* (2013) RBM5 is a male germ cell splicing factor and is required for spermatid differentiation and male fertility. *PLoS Genet* 9(7):e1003628.
185. Messina A*, et al.* (2011) Dysregulation of Semaphorin7A/β1-integrin signaling leads to defective GnRH-1 cell migration, abnormal gonadal development and altered fertility. *Human molecular genetics* 20(24):4759-4774.
186. Crackower MA*, et al.* (2003) Essential role of Fkbp6 in male fertility and homologous chromosome pairing in meiosis. *Science* 300(5623):1291-1295.

187. Guerrero-Bosagna C & Skinner MK (2014) Environmentally induced epigenetic transgenerational inheritance of male infertility. *Current opinion in genetics & development* 26:79-88.
188. Zhang H*, et al.* (2014) Proteomic analysis of mouse testis reveals perfluorooctanoic acid-induced reproductive dysfunction via direct disturbance of testicular steroidogenic machinery. *Journal of proteome research* 13(7):3370-3385.
189. Cochran SD, Cole JB, Null DJ, & Hansen PJ (2013) Single nucleotide polymorphisms in candidate genes associated with fertilizing ability of sperm and subsequent embryonic development in cattle. *Biology of reproduction*:biolreprod. 113.111260.
190. de Vries SS*, et al.* (1999) Mouse MutS-like protein Msh5 is required for proper chromosome synapsis in male and female meiosis. *Genes & development* 13(5):523-531.
191. Xu K, Lu T, Zhou H, Bai L, & Xiang Y (2010) The role of MSH5 C85T and MLH3 C2531T polymorphisms in the risk of male infertility with azoospermia or severe oligozoospermia. *Clinica Chimica Acta* 411(1):49-52.
192. Mandon-Pépin B*, et al.* (2008) Genetic investigation of four meiotic genes in women with premature ovarian failure. *European Journal of Endocrinology* 158(1):107-115.
193. Mamo S, Gal AB, Polgar Z, & Dinnyes A (2008) Expression profiles of the pluripotency marker gene POU5F1 and validation of reference genes in rabbit oocytes and preimplantation stage embryos. *BMC molecular biology* 9(1):67.
194. Okuda H*, et al.* (2015) A Novel Transcriptional Factor Nkapl Is a Germ Cell-Specific Suppressor of Notch Signaling and Is Indispensable for Spermatogenesis.
195. Wiles WG*, et al.* (2014) Mutation of Murine Sox4 Untranslated Regions Results in Partially Penetrant Perinatal Lethality. *In Vivo* 28(5):709-718.
196. Zhou X, Takatoh J, & Wang F (2011) The mammalian class 3 PI3K (PIK3C3) is required for early embryogenesis and cell proliferation. *PloS one* 6(1):e16358.
197. Pangas SA, Li X, Robertson EJ, & Matzuk MM (2006) Premature luteinization and cumulus cell defects in ovarian-specific Smad4 knockout mice. *Molecular Endocrinology* 20(6):1406-1422.
198. Ratts V, Flaws J, Kolp R, Sorenson C, & Tilly J (1995) Ablation of bcl-2 gene expression decreases the numbers of oocytes and primordial follicles established in the post-natal female mouse gonad. *Endocrinology* 136(8):3665-3668.
199. Passe CMM*, et al.* (2008) Loss of the protein NUPR1 (p8) leads to delayed LHB expression, delayed ovarian maturation, and testicular development of a sertoli-cell-only syndrome-like phenotype in mice. *Biology of reproduction* 79(4):598-607.
200. Fan H-Y*, et al.* (2009) MAPK3/1 (ERK1/2) in ovarian granulosa cells are essential for female fertility. *Science* 324(5929):938-941.
201. Thomas T, Dixon MP, Kueh AJ, & Voss AK (2008) Mof (MYST1 or KAT8) is essential for progression of embryonic development past the blastocyst stage and required for normal chromatin architecture. *Molecular and cellular biology* 28(16):5093-5105.
202. Ruiz LA*, et al.* (2011) Single-nucleotide polymorphisms in the lysyl oxidase-like protein 4 and complement component 3 genes are associated with increased risk for endometriosis and endometriosis-associated infertility. *Fertility and sterility* 96(2):512-515.
203. Grasso M*, et al.* (2012) Distribution of GFRA1-expressing spermatogonia in adult mouse testis. *Reproduction* 143(3):325-332.
204. Stoller JZ*, et al.* (2010) Ash2l interacts with Tbx1 and is required during early embryogenesis. *Experimental Biology and Medicine* 235(5):569-576.

205. Stocco DM (2001) StAR protein and the regulation of steroid hormone biosynthesis. *Annual Review of Physiology* 63(1):193-213.
206. Leader B*, et al.* (2002) Formin-2, polyploidy, hypofertility and positioning of the meiotic spindle in mouse oocytes. *Nature cell biology* 4(12):921-928.
207. Rankin TL*, et al.* (2001) Defective zonae pellucidae in Zp2-null mice disrupt folliculogenesis, fertility and development. *Development* 128(7):1119-1126.
208. Kuroki S*, et al.* (2013) JMJD1C, a JmjC domain-containing protein, is required for long-term maintenance of male germ cells in mice. *Biology of reproduction* 89(4):93.
209. Rainey MA*, et al.* (2010) The endocytic recycling regulator EHD1 is essential for spermatogenesis and male fertility in mice. *BMC developmental biology* 10(1):37.
210. Hirai K*, et al.* (2004) HST-1/FGF-4 protects male germ cells from apoptosis under heat-stress condition. *Experimental cell research* 294(1):77-85.
211. Knauff EA*, et al.* (2011) Copy number variants on the X chromosome in women with primary ovarian insufficiency. *Fertility and sterility* 95(5):1584-1588. e1581.
212. Davis RJ, Harding M, Moayedi Y, & Mardon G (2008) Mouse Dach1 and Dach2 are redundantly required for Müllerian duct development. *Genesis* 46(4):205-213.
213. Bione S*, et al.* (2004) Mutation analysis of two candidate genes for premature ovarian failure, DACH2 and POF1B. *Human Reproduction* 19(12):2759-2766.
214. Lacombe A*, et al.* (2006) Disruption of POF1B binding to nonmuscle actin filaments is associated with premature ovarian failure. *The American Journal of Human Genetics* 79(1):113-119.
215. Lu SY*, et al.* (2008) FGF-16 is required for embryonic heart development. *Biochemical and biophysical research communications* 373(2):270-274.
216. Hotta Y*, et al.* (2008) Fgf16 is required for cardiomyocyte proliferation in the mouse embryonic heart. *Developmental dynamics* 237(10):2947-2954.
217. Dohle G, Smit M, & Weber R (2003) Androgens and male fertility. *World journal of urology* 21(5):341-345.
218. Tut TG, Ghadessy FJ, Trifiro M, Pinsky L, & Yong E (1997) Long Polyglutamine Tracts in the Androgen Receptor Are Associated with Reduced Trans-Activation, Impaired Sperm Production, and Male Infertility 1. *The Journal of Clinical Endocrinology & Metabolism* 82(11):3777-3782.
219. Walters K*, et al.* (2007) Female mice haploinsufficient for an inactivated androgen receptor (AR) exhibit age-dependent defects that resemble the AR null phenotype of dysfunctional late follicle development, ovulation, and fertility. *Endocrinology* 148(8):3674-3684.
220. Di Pasquale E, Beck-Peccoz P, & Persani L (2004) Hypergonadotropic ovarian failure associated with an inherited mutation of human bone morphogenetic protein-15 (BMP15) gene. *The American Journal of Human Genetics* 75(1):106-111.
221. Di Pasquale E*, et al.* (2013) Identification of new variants of human BMP15 gene in a large cohort of women with premature ovarian failure. *The Journal of Clinical Endocrinology & Metabolism*.
222. Dixit H*, et al.* (2006) Missense mutations in the BMP15 gene are associated with ovarian failure. *Human genetics* 119(4):408-415.
223. Tiotiu D*, et al.* (2010) Variants of the BMP15 gene in a cohort of patients with premature ovarian failure. *Human Reproduction*:deq073.
224. Galloway S*, et al.* (2002) Bmp15 mutations and ovarian function. *Molecular and cellular endocrinology* 191(1):15-18.

225. Galloway SM*, et al.* (2000) Mutations in an oocyte-derived growth factor gene (BMP15) cause increased ovulation rate and infertility in a dosage-sensitive manner. *Nature genetics* 25(3):279-283.
226. Hanrahan JP*, et al.* (2004) Mutations in the genes for oocyte-derived growth factors GDF9 and BMP15 are associated with both increased ovulation rate and sterility in Cambridge and Belclare sheep (Ovis aries). *Biology of reproduction* 70(4):900-909.
227. Di Pasquale E*, et al.* (2006) Identification of new variants of human BMP15 gene in a large cohort of women with premature ovarian failure. *The Journal of Clinical Endocrinology & Metabolism* 91(5):1976-1979.
228. Huang X*, et al.* (2011) DYNLT3 is required for chromosome alignment during mouse oocyte meiotic maturation. *Reproductive Sciences* 18(10):983-989.
