# Supplementary material for: Genome changes due to artificial selection in U.S. Holstein cattle
Source: BMC Genomics. 2019 Feb 11;20:128. doi: 10.1186/s12864-019-5459-x (PMC6371544; doi:10.1186/s12864-019-5459-x)
Supplement: Supplementary file 10 — Table S3. Genes with documented fertility functions in or near genome regions with signature of selection. (Summarized from Additional file 9: Table S2). (PDF 93 kb) [file 12864_2019_5459_MOESM10_ESM.pdf]

Additional file 10: Table 3. Genes with documented fertility functions in or near genome regions with signature of selection. (Summarized from Table S2)

Male fertility (76 genes)	*AATK, ACRBP, AGO4, AGTPBP1, ATP1A4, AZIN2, BIRC6, BSP5, CATSPER2, CATSPERB, CTNNB1, CYLC2, DHCR24, DPY19L2, EHD1, ELMO1, ELSPBP1, EPAS1, ESRRB, FGF1, FGF4, FGF9, FKBP6, GFRA1, GPR37, GPX5, HMGB2, IGF1R, JMJD1C, LUZP2, MAATS1, MLH3, MTDH, MYBL1, NANOS2, NDRG2, NKAPL, PARP11, PGAP1, PRKAR1A, PRSS37, PTCHD3, RAE1, RBM5, RNF17, RPGR, SEMA3F, SEPT7, SFPQ, SH3GLB1, SHBG, SHCBP1L, SKIV2L, SMC6, SOX30, SPACA1, SPAG16, SPAG6, SPATA16, SPATA3, SPATA33, SPEF2, SPEM1, SRM, SRY, SUMO1, TEKT2, TMEM95, TRPC2, TSKS, TSPY, TSSK4, TTLL5, VRK1, VRK2, YBX2*
Female fertility (99 genes)	*ADAM19, AFP, AKT1, ALKBH3, ANG2, AR, ARFGEF2, ARPC2, ASH2L, BCL2, BMP2, BMP7, BMP15, CCL28, CCND2, CD9, CD48, COX17,CSE1L, DACH2, DIAPH2, DICER1, DYNLT3, EIF2B2, ERMP1, ESR1, FGF16, FGF21, FGF23, FLRT3, FMN2, GHR, GREM1, HMGCR, HSF1, HSPA1A, IGFBP1, IGFBP3, IGFBP7, IL33, INSL5, ISM1, KAT8, KIF16B, KITLG, LAMC1, LATS1, LATS2, LLGL1, LMO4, MAPK3, MECOM, MMADHC, MST1, MTHFD2L, MTOR, MYO18B, NELL2, NEURL4, NTRK2, NTRK3, NUPR1, PAPPA2, PCDH11X, PCDH12, PDE4B, PELO, PGF, PGR, PIK3C3, PIK3CA, POF1B, POLR3G, POU5F1, POU6F2, RAB10, RAPGEF2, RBX1, REV3L, RSBN1L, SFMBT2, SHB, SMAD4, SMC2, SMG7, SMN2, SOX4, SPAG1, STIM1, SULF1, SULF2, TARDBP, TMED2, TMEM60, TSG6, UBIAD1, UCHL3, VANGL2, ZP4*
Fertility in both males and females (23 genes)	*ATHFR, ATP6V0A2, FANCC, FSHR, GHSR, HMGB2, HSD17B12, IL6R, IMMP2L, IZUMO1, LHCGR, LOXL4, MSH5, NPC2, PRLR, PUM1, SLC39A10, SOX2, SPO11, STAR, TYRO3, UBB*
