# Supplementary material for: Genome changes due to artificial selection in U.S. Holstein cattle
Source: BMC Genomics. 2019 Feb 11;20:128. doi: 10.1186/s12864-019-5459-x (PMC6371544; doi:10.1186/s12864-019-5459-x)
Supplement: Supplementary file 11 — Table S4. Immunity genes in or near chromosome regions subjected to genetic selection since 1964. (PDF 255 kb) [file 12864_2019_5459_MOESM11_ESM.pdf]

Additional file 11: Table S4. Immunity genes in or near chromosome regions subjected to genetic selection since 1964.

Chr	Selection signature	Gene symbol	Gene name	Documented immunity function
1	12964810-17425222 (3Mb sliding windows)	*NCAM*	neural cell adhesion molecule 1	affects the innate immune system in the lung [1]
1	**63951419-65580210**	*IGSF11*	immunoglobulin superfamily member 11	a novel target for cancer immunotherapy of gastrointestinal and hepatocellular carcinomas [2]
		CD80	cluster of differentiation 80	costimulatory signals for T cell proliferation, cytokine production, and generation of CTL[3]
		GSK3B	glycogen synthase kinase-3 beta	negatively regulate IFN-ß Production by TLR4-stimulated innate immune cells [4]
**1**	**85294656-88698235** (2Mb sliding windows)	*SOX2*	SRY-box 2	a sequence-specific DNA sensor in neutrophils to initiate innate immunity against microbial infection [5]
		USP13	ubiquitin specific peptidase 13	negatively regulates antiviral responses by deubiquitinating STING [6]
2	79257749-80298164	*STAT1*	signal transducer and activator of transcription 1	targeted disruption of the mouse STAT1 gene results in compromised innate immunity to viral disease [7]
		STAT4	signal transducer and activator of transcription 4	mediated immune mechanisms in protection against plague [8]
**2**	**84373550-86035295**	*SLC39A10*	solute carrier family 39 member 10	controls humoral immunity by modulating B-cell receptor signal strength [9]
**2**	**91221891-92326427**	*CD28*	cluster of differentiation 28	promote T cell survival [10]
**2**	**120914934-121503634**	*TRIM64*	tripartite motif containing 64	Trim62-deficient mice had increased susceptibility to fungal infection [11]
2	128695085-129554709	*GRHL3*	grainyhead like transcription factor 3	A GRHL3-regulated repair pathway suppresses immune-mediated epidermal hyperplasia [12]
		IFNLR1	interferon lambda receptor 1 interleukin 22 receptor	expression of Ifnlr1 on intestinal epithelial cells is critical to the antiviral effects of interferon lambda against norovirus and reovirus [13]
		IL22RA1	subunit alpha 1	critical role of IL-22/IL22-RA1 signaling in pneumococcal pneumonia [14]

**3**	**8685390-9810337**	*CD244*<br>*CD48*<br><br>*CD84*	cluster of differentiation 244<br>cluster of differentiation 48<br><br>cluster of differentiation 84	long noncoding RNA derived from CD244 signaling epigenetically controls CD8+ T-cell immune responses in tuberculosis infection [15]<br>CD48-deficient mice have a pronounced defect in CD4+ T cell activation [16]
3	11105182-11770282	*IGSF8*	immunoglobulin superfamily member 8	This gene encodes a member the EWI subfamily of the immunoglobulin protein superfamily. Members of this family contain a single transmembrane domain, an EWI (Glu-Trp-Ile)-motif and a variable number of immunoglobulin domains. This protein interacts with the tetraspanins CD81 and CD9 and may regulate their role in certain cellular functions including cell migration and viral infection. [17]
**3**	**16444546-16881296**	*CRTC2*	CREB regulated transcription coactivator 2	the CREB/CRTC2 pathway modulates autoimmune disease by promoting Th17 differentiation [18]
**3**	**79282515-80265751**	*PDE4B*	phosphodiesterase 4B	PDE4B gene activation by LPS constitutes a feedback regulation essential for an efficient immune response [19]
4	39012513-39435115	*HGF*	hepatocyte growth factor	key regulator of dendritic cell migration in skin immunity [20]
**5**	**104116518-105105448**	*CD27*<br><br>*CD9*	cluster of differentiation 27<br><br>cluster of differentiation 9	CD27 is required for generation and long-term maintenance of T cell immunity [21]<br>CD9 tetraspanin is not required for the development of peripheral B cells or for humoral immunity [22]
6	90075383-90188972	*ANKRD17*	ankyrin repeat domain 17	Ankrd17 positively regulates RIG-I-like receptor (RLR)-mediated immune signaling [23]
7	11486814-12752376	*IL-27Ra*	interleukin 27 receptor subunit alpha	IL-27Ra-deficient mice were hypersusceptible to experimental autoimmune encephalomyelitis and generated more IL-17-producing T helper cells [24]
**7**	**54455147-57844905**	*RNF14*<br><br>*NDFIP1*	ring finger protein 14<br><br>Nedd4 family interacting protein 1	RNF14 is a regulator of mitochondrial and immune function in muscle [25]<br>mediates peripheral tolerance to self and exogenous antigen by inducing cell cycle exit in responding

				CD4+ T cells [26]
**8**	**37706259-39306605**	*IL33*	interleukin 33	a crucial amplifier of innate rather than acquired immunity [27]
**8**	108652993-109059409	*TLR4*	toll like receptor 4	links innate immunity and fatty acid–induced insulin resistance [28]
**9**	**87190233-88964135**	*LATS1*	large tumor suppressor kinase 1	The Hippo pathway kinases LATS1/2 suppress cancer immunity [29]
**10**	**21516136-22008344** (the gene clusters are at 22.1-25.5 Mb)	135 genes<br>4 genes<br>3 genes<br>3 genes<br>1 gene<br>1 gene<br>18 genes<br>2 genes<br>1 gene	T-cell receptor alpha, T-cell receptor alpha chain V region and C region<br>Ig lambda chain V-I region<br>Ig lambda chain V-II region<br>Ig lambda chain V-III region<br>Ig lambda chain V-VI region<br>Ig kappa chain V-I region<br>Ig kappa chain V-III region<br>Ig kappa chain V-VI region<br>Ig heavy chain V region	T cell receptors recognize foreign antigens and bound to MHC molecules at the surface of antigen presenting cells [30]<br>Immunoglobulins recognize foreign antigens and initiate immune responses such as phagocytosis and the complement system [31]
**10**	**25598535-26289588**	*NDRG2*	NDRG2	one of novel intrinsic factors for regulation of IL-10 production in human myeloid cell [32]
**10**	**36465813-37290812**	*TYRO3*	TYRO3 protein tyrosine kinase	a negative regulator of type 2 immunity [33]
10	51001410-52033595	*ADAM10*	ADAM metallopeptidase domain 10	regulates the production of multiple secreted factors that contribute to autoimmune reactions [34]
11	6602495-7047686	*MAP4K4*<br>*IL1R2*<br>*IL1R1*<br>*IL1RL2*<br>*IL1RL1*<br>*IL18R*<br>*IL18RAP*	mitogen-activated protein kinase kinase kinase kinase 4<br>interleukin 1 receptor type 2<br>interleukin 1 receptor type 1<br>interleukin 1 receptor like 2<br>interleukin 1 receptor like 1<br>interleukin 18 receptor 1<br>interleukin 18 receptor accessory protein	promotes vascular inflammation and atherosclerosis [35]<br>The IL-1 family: regulators of immunity [36]<br>The IL-1 family: regulators of immunity [36]<br>The IL-1 family: regulators of immunity [36]<br>The IL-1 family: regulators of immunity [36]<br>The IL-1 family: regulators of immunity [36]<br>The IL-1 family: regulators of immunity [36]

**11**	**15814741-16759475**	*RASGRP3*	RAS, guanyl releasing protein 3	limits inflammatory response [37]
**11**	**28577854-29495322**	*EPAS1*	endothelial PAS domain protein 1	links DOCK8 deficiency to atopic skin inflammation via IL-31 induction [38]
**12**	**35886159-37211264**	*LATS2*	large tumor suppressor kinase 2	The Hippo pathway kinases LATS1/2 suppress cancer immunity [29]
18	34794005-35027876	*TRADD*	TNFRSF1A associated via death domain	an essential component of the RIG-like helicase antiviral pathway [39]
**18**	**50581375-51369666**	*AXL*	AXL receptor tyrosine kinase	required for T cell priming and antiviral immunity [40]
		CEACAM1	carcinoembryonic antigen related cell adhesion molecule 1	a regulatory co-receptor for both lymphoid and myeloid cell types [41]
18	58696066-61421776	33 genes	cationic amino acid transporters	play key roles in the survival and transmission of apicomplexan parasites [42]
		9 genes	sialic acid-binding Ig-like lectin	Sialic acid-binding immunoglobulin-like lectins (SIGLECs) are a family of cell surface proteins belonging to the immunoglobulin superfamily. They mediate protein-carbohydrate interactions by selectively binding to different sialic acid moieties present on glycolipids and glycoproteins. [43]
**20**	**9919863-10295382**	*NAIP*	NLR family apoptosis inhibitory protein	recognizes bacterial type III secretion needle protein for inflammasome activation [44]
**21**	**64129449-66380363**	*BCL11B*	B-cell CLL/lymphoma 11B	required for differentiation and survival of αβ T lymphocytes [45]
22	53695239-54103673	*CCR1*	C-C motif chemokine receptor 9	interaction between CCR1 and CCL5 modulates the innate immune response during sepsis [46]
		XCR1	X-C motif chemokine receptor 1	an integral component in the development of efficient cytotoxic immunity in vivo [47]
		CXCR6	C-X-C motif chemokine receptor 6	adaptive immunity to structurally diverse antigens [48]
			C-C motif chemokine	an essential role for CCR9 in the homing of pDC to

		CCR9	receptor 9	the intestine under homeostatic and inflammatory conditions [49]
23	7627089-7874236	*BAK1*	BCL2 antagonist/killer 1	The receptor-like kinase SERK3/BAK1 is a central regulator of innate immunity in plants [50]
**23**	**25178791-27992880**	MHC	major histocompatibility complex	associated with many diseases [51]
		BTNL2	butyrophilin-like 2 (MHC class II associated)	Inhibit T Cell Activation [52]
		ABHD16A	abhydrolase domain containing 16A	immunomodulatory lysophosphatidylserines are regulated by ABHD16A and ABHD12 interplay [53]
		NFKBIL1	NFKB inhibitor like 1	confers resistance to experimental autoimmune arthritis through the regulation of dendritic cell functions [54]
24	6659975-7838578	*CD226*	cluster of differentiation 226	regulates human T cell function [55]
**25**	**23825593-26337016**	*IL4R*	interleukin 4 receptor	regulate increased lymphocyte proliferation and survival [56]
		IL21R	interleukin 21 receptor	critical for sustained functionality and control of chronic viral infection [57, 58]
		IL27	interleukin 27	expression of IL-27 in murine carcinoma cells produces antitumor effects and induces protective immunity in inoculated host animals [59]
		LAT	linker for activation of T-cells	essential role in T cell development [60]
		CD19	cluster of differentiation 19	regulates signal transduction thresholds governing humoral immunity and autoimmunity [61]
26	16999066-17465791	*BLNK*	B-cell linker	an essential role in human B cell development [62]
**29**	**43328607-43839783**	*BATF2*	basic leucine zipper ATF-like transcription factor 2	Batf2/Irf1 induces inflammatory responses in classically activated macrophages, lipopolysaccharides, and mycobacterial infection [63]

References

1. Ulm C, Saffarzadeh M, Mahavadi P, Müller S, Prem G, Saboor F, et al. Soluble polysialylated NCAM: a novel player of the innate immune system in the lung. Cellular and molecular life sciences. 2013;70(19):3695-708.
2. Watanabe T, Suda T, Tsunoda T, Uchida N, Ura K, Kato T, et al. Identification of immunoglobulin superfamily 11 (IGSF11) as a novel target for cancer immunotherapy of gastrointestinal and hepatocellular carcinomas. Cancer science. 2005;96(8):498-506.
3. Lanier LL, O'Fallon S, Somoza C, Phillips JH, Linsley PS, Okumura K, et al. CD80 (B7) and CD86 (B70) provide similar costimulatory signals for T cell proliferation, cytokine production, and generation of CTL. The Journal of Immunology. 1995;154(1):97-105.
4. Beurel E, Michalek SM, Jope RS. Innate and adaptive immune responses regulated by glycogen synthase kinase-3 (GSK3). Trends in immunology. 2010;31(1):24-31.
5. Xia P, Wang S, Ye B, Du Y, Huang G, Zhu P, et al. Sox2 functions as a sequence-specific DNA sensor in neutrophils to initiate innate immunity against microbial infection. Nature immunology. 2015;16(4):366-75.
6. Sun H, Zhang Q, Jing Y-Y, Zhang M, Wang H-Y, Cai Z, et al. USP13 negatively regulates antiviral responses by deubiquitinating STING. Nature Communications. 2017;8:ncomms15534.
7. Durbin JE, Hackenmiller R, Simon MC, Levy DE. Targeted disruption of the mouse Stat1 gene results in compromised innate immunity to viral disease. Cell. 1996;84(3):443-50.
8. Elvin SJ, Williamson ED. Stat 4 but not Stat 6 mediated immune mechanisms are essential in protection against plague. Microbial pathogenesis. 2004;37(4):177-84.
9. Hojyo S, Miyai T, Fujishiro H, Kawamura M, Yasuda T, Hijikata A, et al. Zinc transporter SLC39A10/ZIP10 controls humoral immunity by modulating B-cell receptor signal strength. Proceedings of the National Academy of Sciences. 2014;111(32):11786-91.
10. Boise LH, Minn AJ, Noel PJ, June CH, Accavitti MA, Lindsten T, et al. CD28 costimulation can promote T cell survival by enhancing the expression of Bcl-x L. Immunity. 1995;3(1):87-98.
11. Cao Z, Conway KL, Heath RJ, Rush JS, Leshchiner ES, Ramirez-Ortiz ZG, et al. Ubiquitin ligase TRIM62 regulates CARD9-mediated anti-fungal immunity and intestinal inflammation. Immunity. 2015;43(4):715-26.
12. Gordon WM, Zeller MD, Klein RH, Swindell WR, Ho H, Espetia F, et al. A GRHL3-regulated repair pathway suppresses immune-mediated epidermal hyperplasia. The Journal of clinical investigation. 2014;124(12):5205.
13. Baldridge MT, Lee S, Brown JJ, McAllister N, Urbanek K, Dermody TS, et al. Expression of Ifnlr1 on intestinal epithelial cells is critical to the antiviral effects of interferon lambda against norovirus and reovirus. Journal of virology. 2017;91(7):e02079-16.

14. Trevejo-Nunez G, Elsegeiny W, Conboy P, Chen K, Kolls JK. Critical Role of IL-22/IL22-RA1 Signaling in Pneumococcal Pneumonia. The Journal of Immunology. 2016;197(5):1877-83.
15. Wang Y, Zhong H, Xie X, Chen CY, Huang D, Shen L, et al. Long noncoding RNA derived from CD244 signaling epigenetically controls CD8+ T-cell immune responses in tuberculosis infection. Proceedings of the National Academy of Sciences. 2015;112(29):E3883-E92.
16. González-Cabrero J, Wise CJ, Latchman Y, Freeman GJ, Sharpe AH, Reiser H. CD48-deficient mice have a pronounced defect in CD4+ T cell activation. Proceedings of the National Academy of Sciences. 1999;96(3):1019-23.
17. The National Center for Biotechnology Information. https://www.ncbi.nlm.nih.gov/gene/93185.
18. Hernandez JB, Chang C, LeBlanc M, Grimm D, Le Lay J, Kaestner KH, et al. The CREB/CRTC2 pathway modulates autoimmune disease by promoting Th17 differentiation. Nature communications. 2015;6:7216.
19. Jin S-LC, Conti M. Induction of the cyclic nucleotide phosphodiesterase PDE4B is essential for LPS-activated TNF-α responses. Proceedings of the National Academy of Sciences. 2002;99(11):7628-33.
20. Baek J-H, Birchmeier C, Zenke M, Hieronymus T. The HGF receptor/Met tyrosine kinase is a key regulator of dendritic cell migration in skin immunity. The Journal of Immunology. 2012;189(4):1699-707.
21. Hendriks J, Gravestein LA, Tesselaar K, van Lier RA, Schumacher TN, Borst J. CD27 is required for generation and long-term maintenance of T cell immunity. Nature immunology. 2000;1(5):433-40.
22. Cariappa A, Shoham T, Liu H, Levy S, Boucheix C, Pillai S. The CD9 tetraspanin is not required for the development of peripheral B cells or for humoral immunity. The Journal of Immunology. 2005;175(5):2925-30.
23. Wang Y, Tong X, Li G, Li J, Deng M, Ye X. Ankrd17 positively regulates RIG-I-like receptor (RLR)-mediated immune signaling. European journal of immunology. 2012;42(5):1304-15.
24. Batten M, Li J, Yi S, Kljavin NM, Danilenko DM, Lucas S, et al. Interleukin 27 limits autoimmune encephalomyelitis by suppressing the development of interleukin 17–producing T cells. Nature immunology. 2006;7(9):929-36.
25. Ingham AB, Osborne SA, Menzies M, Briscoe S, Chen W, Kongsuwan K, et al. RNF14 is a regulator of mitochondrial and immune function in muscle. BMC systems biology. 2014;8(1):10.
26. Altin JA, Daley SR, Howitt J, Rickards HJ, Batkin AK, Horikawa K, et al. Ndfip1 mediates peripheral tolerance to self and exogenous antigen by inducing cell cycle exit in responding CD4+ T cells. Proceedings of the National Academy of Sciences. 2014;111(6):2067-74.
27. Oboki K, Ohno T, Kajiwara N, Arae K, Morita H, Ishii A, et al. IL-33 is a crucial amplifier of innate rather than acquired immunity. Proceedings of the National Academy of Sciences. 2010;107(43):18581-6.
28. Shi H, Kokoeva MV, Inouye K, Tzameli I, Yin H, Flier JS. TLR4 links innate immunity and fatty acid–induced insulin resistance. Journal of Clinical Investigation. 2006;116(11):3015.
29. Moroishi T, Hayashi T, Pan W-W, Fujita Y, Holt MV, Qin J, et al. The Hippo pathway kinases LATS1/2 suppress cancer immunity. Cell. 2016;167(6):1525-39. e17.

30. The National Center for Biotechnology Information. https://www.ncbi.nlm.nih.gov/gene/21473.
31. The National Center for Biotechnology Information. https://www.ncbi.nlm.nih.gov/gene/243469.
32. Choi S-C, Kim KD, Kim J-T, Oh S-S, Yoon SY, Song EY, et al. NDRG2 is one of novel intrinsic factors for regulation of IL-10 production in human myeloid cell. Biochemical and biophysical research communications. 2010;396(3):684-90.
33. Chan PY, Silva EAC, De Kouchkovsky D, Joannas LD, Hao L, Hu D, et al. The TAM family receptor tyrosine kinase TYRO3 is a negative regulator of type 2 immunity. Science. 2016;352(6281):99-103.
34. Gibb DR, Saleem SJ, Chaimowitz NS, Mathews J, Conrad DH. The emergence of ADAM10 as a regulator of lymphocyte development and autoimmunity. Molecular immunology. 2011;48(11):1319-27.
35. Flach RJR, Skoura A, Matevossian A, Danai LV, Zheng W, Cortes C, et al. Endothelial protein kinase MAP4K4 promotes vascular inflammation and atherosclerosis. Nature communications. 2015;6.
36. Sims JE, Smith DE. The IL-1 family: regulators of immunity. Nature Reviews Immunology. 2010;10(2):89-102.
37. Tang S, Chen T, Yu Z, Zhu X, Yang M, Xie B, et al. RasGRP3 limits Toll-like receptor-triggered inflammatory response in macrophages by activating Rap1 small GTPase. Nature communications. 2014;5.
38. Yamamura K, Uruno T, Shiraishi A, Tanaka Y, Ushijima M, Nakahara T, et al. The transcription factor EPAS1 links DOCK8 deficiency to atopic skin inflammation via IL-31 induction. Nature communications. 2017;8.
39. Michallet M-C, Meylan E, Ermolaeva MA, Vazquez J, Rebsamen M, Curran J, et al. TRADD protein is an essential component of the RIG-like helicase antiviral pathway. Immunity. 2008;28(5):651-61.
40. Schmid ET, Pang IK, Silva EAC, Bosurgi L, Miner JJ, Diamond MS, et al. AXL receptor tyrosine kinase is required for T cell priming and antiviral immunity. Elife. 2016;5:e12414.
41. Gray-Owen SD, Blumberg RS. CEACAM1: contact-dependent control of immunity. Nature Reviews Immunology. 2006;6(6):433-46.
42. Rajendran E, Hapuarachchi SV, Miller CM, Fairweather SJ, Cai Y, Smith NC, et al. Cationic amino acid transporters play key roles in the survival and transmission of apicomplexan parasites. Nature Communications. 2017;8.
43. The National Center for Biotechnology Information. https://www.ncbi.nlm.nih.gov/gene/89858.
44. Yang J, Zhao Y, Shi J, Shao F. Human NAIP and mouse NAIP1 recognize bacterial type III secretion needle protein for inflammasome activation. Proceedings of the National Academy of Sciences. 2013;110(35):14408-13.
45. Wakabayashi Y, Watanabe H, Inoue J, Takeda N, Sakata J, Mishima Y, et al. Bcl11b is required for differentiation and survival of αβ T lymphocytes. Nature immunology. 2003;4(6):533-9.
46. Ness TL, Carpenter KJ, Ewing JL, Gerard CJ, Hogaboam CM, Kunkel SL. CCR1 and CC chemokine ligand 5 interactions exacerbate innate immune responses during sepsis. The Journal of Immunology. 2004;173(11):6938-48.
47. Dorner BG, Dorner MB, Zhou X, Opitz C, Mora A, Güttler S, et al. Selective expression of the chemokine receptor XCR1 on cross-presenting dendritic cells determines cooperation with CD8+ T cells. Immunity. 2009;31(5):823-33.

48. Paust S, Gill HS, Wang B-Z, Flynn MP, Moseman EA, Senman B, et al. Critical role for the chemokine receptor CXCR6 in NK cell-mediated antigen-specific memory of haptens and viruses. Nature immunology. 2010;11(12):1127-35.
49. Wendland M, Czeloth N, Mach N, Malissen B, Kremmer E, Pabst O, et al. CCR9 is a homing receptor for plasmacytoid dendritic cells to the small intestine. Proceedings of the National Academy of Sciences. 2007;104(15):6347-52.
50. Heese A, Hann DR, Gimenez-Ibanez S, Jones AM, He K, Li J, et al. The receptor-like kinase SERK3/BAK1 is a central regulator of innate immunity in plants. Proceedings of the National Academy of Sciences. 2007;104(29):12217-22.
51. Kumánovics A, Takada T, Lindahl KF. Genomic organization of the mammalian MHC. Annual Review of Immunology. 2003;21(1):629-57.
52. Nguyen T, Liu XK, Zhang Y, Dong C. BTNL2, a butyrophilin-like molecule that functions to inhibit T cell activation. The Journal of Immunology. 2006;176(12):7354-60.
53. Kamat SS, Camara K, Parsons WH, Chen D-H, Dix MM, Bird TD, et al. Immunomodulatory lysophosphatidylserines are regulated by ABHD16A and ABHD12 interplay. Nature chemical biology. 2015;11(2):164-71.
54. Chiba T, Matsuzaka Y, Warita T, Sugoh T, Miyashita K, Tajima A, et al. NFKBIL1 confers resistance to experimental autoimmune arthritis through the regulation of dendritic cell functions. Scandinavian journal of immunology. 2011;73(5):478-85.
55. Lozano E, Dominguez-Villar M, Kuchroo V, Hafler DA. The TIGIT/CD226 axis regulates human T cell function. The Journal of Immunology. 2012;188(8):3869-75.
56. Venmar KT, Fingleton B. Lessons from immunology: IL4R directly promotes mammary tumor metastasis. Oncoimmunology. 2014;3(9):e955373.
57. Fröhlich A, Kisielow J, Schmitz I, Freigang S, Shamshiev AT, Weber J, et al. IL-21R on T cells is critical for sustained functionality and control of chronic viral infection. Science. 2009;324(5934):1576-80.
58. John SY, Du M, Zajac AJ. A vital role for interleukin-21 in the control of a chronic viral infection. Science. 2009;324(5934):1572-6.
59. Chiyo M, Shimozato O, Yu L, Kawamura K, Iizasa T, Fujisawa T, et al. Expression of IL-27 in murine carcinoma cells produces antitumor effects and induces protective immunity in inoculated host animals. International journal of cancer. 2005;115(3):437-42.
60. Zhang W, Sommers CL, Burshtyn DN, Stebbins CC, DeJarnette JB, Trible RP, et al. Essential role of LAT in T cell development. Immunity. 1999;10(3):323-32.
61. Tedder TF, Inaoki M, Sato S. The CD19–CD21 complex regulates signal transduction thresholds governing humoral immunity and autoimmunity. Immunity. 1997;6(2):107-18.
62. Minegishi Y, Rohrer J, Coustan-Smith E, Lederman HM, Pappu R, Campana D, et al. An essential role for BLNK in human B cell development. Science. 1999;286(5446):1954-7.

63. Roy S, Guler R, Parihar SP, Schmeier S, Kaczkowski B, Nishimura H, et al. Batf2/Irf1 induces inflammatory responses in classically activated macrophages, lipopolysaccharides, and mycobacterial infection. The Journal of Immunology. 2015;194(12):6035-44.
