# Supplementary material for: Genome changes due to artificial selection in U.S. Holstein cattle
Source: BMC Genomics. 2019 Feb 11;20:128. doi: 10.1186/s12864-019-5459-x (PMC6371544; doi:10.1186/s12864-019-5459-x)
Supplement: Supplementary file 12 — Data Set1. SNP and pedigree data of the University of Minnesota Holstein control line unselected since 1964. (ZIP 5309 kb) [file 12864_2019_5459_MOESM12_ESM.zip › control_line_data/readme_control_line_data.pdf]

Additional file 12: Data Set1. SNP and pedigree data of the University of Minnesota Holstein control line unselected since 1964.

This additional file has four files:

1. SNP_control_line.zip. This file has 31 chromosome files, where 1-29 are the 29 autosomes, '30' is the X chromosome, and '99' contains SNPs with unknown chromosome locations. Column 1 is the sample-ID coded at AGIL/USDA, and Column 2 is the Holstein ID. The remaining columns are the SNP data with '5' denoting missing genotype, '1' for the heterozygous genotype, and '0' and '2' for the two homozygous genotypes of each SNP. The first row of each file contains SNP names. This file also contains SNP_map.dat, which contains SNP names and the UMD 3.1 chromosome positions.
2. control_line_pedigree.xlsx. This is the historical pedigree of the control line complied from multiple sources including USDA Holstein records and the control line record books by Yang Da, George Wiggans, Harvey Blackburn and Brian Crooker. Majority of the animal IDs should be correct but ID errors in early generations could exist. The animal IDs are the Holstein ID's after removing 'H' and leading 0's.
3. control_line_pedigree_graph.pdf. This is the graph of the control line historical pedigree, produced using the Pedigraph program developed by John Garbe and Yang Da (https://animalgene.umn.edu/pedigraph).
4. readme_control_line_data.pdf. This readme file.
