# Supplementary figures and images for: Genome changes due to artificial selection in U.S. Holstein cattle
Source: BMC Genomics. 2019 Feb 11;20:128. doi: 10.1186/s12864-019-5459-x (PMC6371544; doi:10.1186/s12864-019-5459-x)

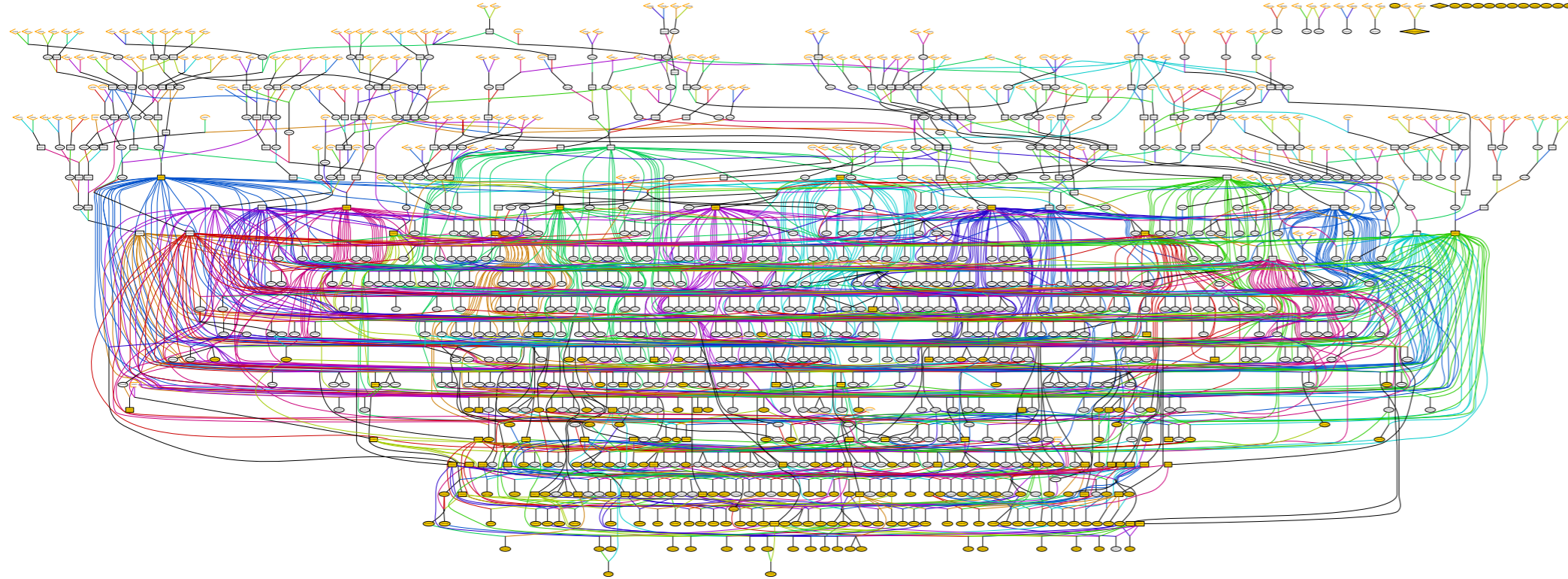

Supplement: Supplementary file 12 — Data Set1. SNP and pedigree data of the University of Minnesota Holstein control line unselected since 1964. (ZIP 5309 kb) [file 12864_2019_5459_MOESM12_ESM.zip › control_line_data/control_line_pedigree_graph.pdf]
